# Clinical guidelines for the management of treatment-resistant depression: French recommendations from experts, the French Association for Biological Psychiatry and Neuropsychopharmacology and the fondation FondaMental

**DOI:** 10.1186/s12888-019-2237-x

**Published:** 2019-08-28

**Authors:** D. Bennabi, T. Charpeaud, A. Yrondi, J.-B. Genty, S. Destouches, S. Lancrenon, N. Alaïli, F. Bellivier, T. Bougerol, V. Camus, J.-M. Dorey, O. Doumy, F. Haesebaert, J. Holtzmann, C. Lançon, M. Lefebvre, F. Moliere, I. Nieto, C. Rabu, R. Richieri, L. Schmitt, F. Stephan, G. Vaiva, M. Walter, M. Leboyer, W. El-Hage, P.-M. Llorca, P. Courtet, B. Aouizerate, E. Haffen

**Affiliations:** 1 Service de Psychiatrie clinique, Centre Expert Dépression Résistante FondaMental, Centre Investigation Clinique 1431-INSERM, EA 481 Neurosciences, Université de Bourgogne Franche Comté, Besançon, France; 2 Service de Psychiatrie de l’adulte B, Centre Expert Dépression Résistante FondaMental, CHU de Clermont-Ferrand, Clermont-Ferrand, France; 3 Service de Psychiatrie et de Psychologie Médicale de l’adulte, Centre Expert Dépression Résistante FondaMental, CHRU de Toulouse, Hôpital Purpan, ToNIC, Toulouse NeuroImaging Center Université de Toulouse, Inserm, UPS, Toulouse, France; 4 SYLIA-STAT, 10, boulevard du Maréchal-Joffre, 92340 Bourg-la-Reine, France; 5 Service de Psychiatrie adulte, Centre Expert Dépression Résistante FondaMental, Hôpital Fernand-Widal, Paris, France; 6 Service de Psychiatrie de l’adulte, CS 10217, Centre Expert Dépression Résistante FondaMental, CHU de Grenoble, Hôpital Nord, Grenoble, France; 7 Clinique Psychiatrique Universitaire, Centre Expert Dépression Résistante FondaMental, CHRU de Tours, Université de Tours, Inserm U1253 imaging and Brain : iBrain, Tours, France; 8 Old Age Psychiatry Unit, pôle EST, Centre Hospitalier le Vinatier, Bron, France; 9 Brain Dynamics and Cognition, Lyon Neuroscience Research Center, INSERM U1028, CNRS UMR 5292, Lyon, France; 10 Geriatrics Unit, CM2R, Hospices civils de Lyon, Hôpital des Charpennes, Villeurbanne, France; 11 Pôle de Psychiatrie Générale et Universitaire, Centre Expert Dépression Résistante FondaMental, CH Charles Perrens, UMR INRA 1286, NutriNeuro, Université de Bordeaux, Bordeaux, France; 12 Service universitaire des pathologies psychiatriques résistantes, Centre expert FondaMental, PSYR2 Team, Lyon Neuroscience Research Center, INSERM U1028, CNRS UMR5292, Centre Hospitalier Le Vinatier, University Lyon 1, Bron, France; 13 Pôle Psychiatrie, Centre Expert Dépression Résistante FondaMental, CHU La Conception, Marseille, France; 14 Département des Urgences et Post-Urgences Psychiatriques, Centre Expert Dépression Résistante FondaMental, CHU Montpellier, University of Montpellier, Montpellier, France; 15 DHU PePSY, Pole de psychiatrie et d’addictologie des Hôpitaux Universitaires Henri Mondor, Université Paris Est Créteil, Créteil, France; 16 Service hospitalo-universitaire de psychiatrie d’adultes et de psychiatrie de liaison - secteur 1, Centre Expert Dépression Résistante Fondamental, CHRU Brest, hôpital de Bohars, Bohars, France; 17 Service de Psychiatrie adulte, Centre Expert Dépression Résistante FondaMental, CHU de Lille, Hôpital Fontan 1, Lille, France; 18 Department of Clinical Psychiatry, 25030 Besançon University Hospital, 25030 Besançon, France

**Keywords:** Treatment resistant depression, Antidepressants, Expert consensus guidelines, Pharmacotherapy, Major depressive disorder

## Abstract

**Background:**

Clear guidance for successive antidepressant pharmacological treatments for non-responders in major depression is not well established.

**Method:**

Based on the RAND/UCLA Appropriateness Method, the French Association for Biological Psychiatry and Neuropsychopharmacology and the fondation FondaMental developed expert consensus guidelines for the management of treatment-resistant depression. The expert guidelines combine scientific evidence and expert clinicians’ opinions to produce recommendations for treatment-resistant depression. A written survey comprising 118 questions related to highly-detailed clinical presentations was completed on a risk-benefit scale ranging from 0 to 9 by 36 psychiatrist experts in the field of major depression and its treatments. Key-recommendations are provided by the scientific committee after data analysis and interpretation of the results of the survey.

**Results:**

The scope of these guidelines encompasses the assessment of pharmacological resistance and situations at risk of resistance, as well as the pharmacological and psychological strategies in major depression.

**Conclusion:**

The expert consensus guidelines will contribute to facilitate treatment decisions for clinicians involved in the daily assessment and management of treatment-resistant depression across a number of common and complex clinical situations.

**Electronic supplementary material:**

The online version of this article (10.1186/s12888-019-2237-x) contains supplementary material, which is available to authorized users.

## Background

Depressive disorders are one of the most pressing public health problems, directly accounting for 4.4% of disease burden worldwide and 7.2% in the European Union [1, 2]. Major depressive disorder (MDD) is a predominantly recurrent disorder, as 50–80% of patients who have received psychiatric care for an episode of major depression have at least one further episode and a median of four episodes in a lifetime. Moreover, approximately 20–30% of patients with MDD develop a chronic course of their disease resulting in a decreased quality of life and increased care utilisation and costs [3, 4]. Pharmacologic strategies remain the cornerstone of treatment, but response rates to the first-line antidepressant (ADT) are moderate (40–60%), and remission is achieved in a minority of patients (from 30 to 45%) [5, 6]. Several risk factors have been related to a poor response to ADT treatment, including psychosocial factors, clinical characteristics of the current depressive episode, psychiatric and somatic comorbidities or biological factors [7–9]. In clinical practice, the availability of novel ADT agents, combined with psychotherapy or brain stimulation techniques, offer a wide array of strategies, but raise questions regarding the selection of the most appropriate therapy for a given patient Several guidelines have already been established by professional societies to assist clinical decision-making at different stages of the treatment. They were developed after a critical analysis of scientific data, which were selected and ranked according to their level of evidence, so that data issued from randomised, double-blind, controlled trials (RCT) covering large study samples were considered to provide the highest level of evidence. Despite this rigorous approach at both the scientific and methodological levels, the guideline-based data raise critical issues regarding the insufficiency of evidence beyond the second-line treatment. Moreover, the use of restrictive criteria in RCT (i.e. the exclusion of populations with psychiatric or organic comorbidities, high suicide risks, or high levels of pharmacological resistance) precludes clear statements in those specific clinical situations. These serious limitations justify the relevance of the Formal Consensus method to specify a prescription framework for specific populations or clinical situations for which evidence are scarce or debated. From methodological considerations, the Formal Consensus method primarily refers to individual clinical expertise of a panel of experts coupled with available external clinical evidence. The French National Health agency recommends the Formal Consensus method when the following conditions are met:

- No or insufficient levels of evidence addressing the question.
- Possibility to decline the topic in easily identifiable clinical situations.
- Need to identify and select the strategies deemed appropriate by an independent panel of experts from amongst several alternative therapeutic options.

In the field of treatment-resistant depression (TRD), in which empirical literature is lacking for specific iterative medications, the formal consensus method appears to be particularly appropriate. The expert guidelines provide useful insights into the treatment practices of clinician experts in clinical situations that require the thoughtful application of evidence-based knowledge. By focusing on « real world » prescribing habits of experienced clinicians, this methodology helps to fill the gap between empirical literature and clinical practice. As part of a process to improve the quality of care, the French Association for Biological Psychiatry and Neuropsychopharmacology (AFPBN) and the fondation FondaMental (www.fondation-fondamental.org) have developed Formal Consensus Guidelines that are expected to provide clear guidance regarding treatment options for non-responders and partial responders in major depression.

Before application of these guidelines, a diagnostic re-evaluation is essential to the proper management of these patients. In particular, exclusion of “pseudo-resistance” is a crucial step of identification of patients with high risk of treatment resistance. Causes of pseudo-resistance include inadequate dose and treatment duration of the antidepressant, insufficient plasma levels, non-compliance of the patient regarding medication intake or relevant psychiatric and/or somatic comorbidities. We provide a synthesis of the deliberations of the French experts’ panel, thereby enabling major recommendations in TRD to be formulated*.* We will then discuss the interests and conditions of the use of these recommendations in light of the evidence-based guidelines (EBG).

## Methods

Expert recommendations were determined using the RAND/UCLA Appropriateness Method (for a full description of the organisation, expert panel, questionnaire development, and data analysis, see Additional file 1 and 2). This method has been previously described and uses a comprehensive review and analysis of the literature in combination with a structured, quantitative technique for incorporating the judgment of expert clinicians to produce appropriateness assessments for several highly detailed and illustrative clinical presentations [10]. The limitations of a purely evidence-based approach are recognised, and the collective judgment of experts is integrated into the process. However, unlike the Delphi method, the process is meant to detect agreement among experts without trying to promote consensus and potentially reduce the real differences of clinical opinion. For these guidelines, a consensus survey of expert opinion on the pharmacologic treatment of TRD was undertaken by the AFBPN and FondaMental foundation. A panel of 36 psychiatrist experts in the field of MDD rated the appropriateness of treatment options for different clinical scenarios using a modified version of the RAND 9-point scale. Key-recommendations for lines of treatment were provided by the scientific committee after data analysis and interpretation of the results of the survey. This method has been previously used to measure the appropriateness of a wide variety of medical and surgical interventions [11, 12].

## Results

### Expert panel: description

Based on the selected criteria, 36 experts constituted the panel. Their socio-demographic characteristics and professional activities are presented in Additional file 5.

### Reading rules

- Therapeutic strategies are organised around successive lines of treatment (from first to sixth) and, for each line, two levels of recommendations are provided, as first and second intentions, respectively
- Definitions of response, complete and partial remission, chronic depression, relapse and recurrence are provided in Additional file 3
- A classification of ADT is provided in Additional file 4
- Optimisation consists of increasing the dose of the treatment up to the maximally tolerated dose as recommended by the summary of the characteristics of the product.
- Potentiation consists of adding an originally non-ADT pharmacological agent in association with the ADT drug over a given period of time in order to obtain pharmacological synergy that may improve the therapeutic properties
- Association consists of combining two ADTs with distinct and complementary pharmacological profiles over a given period of time

### Definition of resistant depression and at-risk situations

#### Based on clinical expert consensus, the definition of treatment-resistant depression adopted is the failure of two ADT of adequate duration and dose. The optimal duration is 4 to 6 weeks when the targeted dose is obtained

A history of an unresponsive form of depression is considered the main predictive factor of treatment-resistance and should be meticulously considered. Other potentially predictive indicators are considered, including:

- Comorbid anxiety disorder
- Comorbid substance abuse
- Comorbid personality disorders
- Comorbid non-psychiatric chronic and organic disease

The duration of the untreated episode and early or late age at onset of the first depressive episode as well as the illness severity or onset of depression during the peri-menopausal period are recognised as increasing the risk for treatment resistance. Of note, childhood adversity was not explored in our questionnaire, despite it is a well-established prognostic factor for TRD.

Comorbid neurodegenerative, neurovascular or autoimmune diseases are systematically considered to negatively impact the treatment response. Coronary, endocrine and pulmonary diseases, migraines and cancers could eventually limit clinical alleviation.

Among the medications that may interfere with clinical improvement, only interferon therapy was firmly considered among the risk factors. Corticoid treatments, isotretinoin or first-generation antipsychotics must be carefully considered. No clear consensus emerged regarding the risk of non-response associated with second-generation antipsychotics, valproate, carbamazepine, gabapentin or topiramate.

### Assessments of treatment-resistant depression

The expert panel recommended systematically performing a comprehensive assessment of the depressive episode using the following clinical instruments:

- Clinician-rated and self-rated scales of depression severity
- Hypomania rating scale
- Suicide rating scale

A mood diagram, a structured diagnostic interview as well as a specific questionnaire exploring anxiety disorders can be non-systematically administered.

In cases of depression unresponsive to at least two previous ADT, experts recommended systematically performing the following paraclinical examinations:

- Complete blood count, blood electrolytes, liver and renal functions
- Lipid profile (cholesterol, triglyceride) and glucose levels
- Thyroid-Stimulating Hormone levels (TSHus)
- Plasma levels of ADT
- Electrocardiogram
- Brain MRI

Paraclinical examinations could be proposed depending on the clinical state of the patient:

- Pharmacogenetic testing for CYP enzymes
- Urinary and blood toxicological analysis
- Plasma cortisol determination
- Polysomnographic sleep assessment
- Electroencephalography
- Plasma levels of Vitamin D
- Sexual hormone levels
- Carbohydrate deficient transferrin determination
- C-reactive protein measurement

Close monitoring of blood pressure, abdominal circumference, weight, suicide risk, mood-switching, lipid profile and glucose levels is systematically recommended when ADT are prescribed.

### Principles of clinical and pharmacological management

#### Indications for hospitalisation

Hospitalisation is systematically recommended in cases of:

- High suicidal risk
- Presence of psychotic symptoms
- Severe forms of MDD
- Failure of three unsuccessful attempts of ADT
- Need for electroconvulsive therapy

Hospitalisation can be considered in cases of:

- Risk of poor adherence to treatment
- Failure of two previous ADT
- Comorbidity with a severe medical condition
- Co-occurrence with other psychiatric disorders
- Lack of adequate familial support
- Intolerance to current medication
- Need for benzodiazepines withdrawal
- Need for monoamine oxidase inhibitors, transcranial magnetic stimulation or transcranial direct current stimulation

The need to introduce a tricyclic ADT, lithium, pramipexole or second-generation antipsychotic is not considered as an indication for hospitalisation.

#### Adjuvant treatments

For patients with anxious features, the adjunctive use of benzodiazepines or hydroxyzine is systematically recommended. The use of buspirone, pregabalin or an ADT belonging to a different pharmacological class is possible in this indication.

The use of an ADT from the same pharmacological class is not recommended.

For patients with sleep disorders, the adjunctive use of hypnotic (zolpidem or zopiclone) is systematically recommended. The use of hydroxyzine, benzodiazepines or an ADT with a different pharmacological profile is possible as an alternative therapeutic option.

For patients with a high risk of self-harm injury, no clear consensus has emerged regarding the use of adjunctive treatment. The experts have suggested several possible options based on the prescription of hydroxyzine, benzodiazepines, second-generation antipsychotics or lithium.

#### Treatments with an ADT action

*The following classes or medications are recognised as having antidepressant properties:*

- Selective serotonin reuptake inhibitors (SSRIs)
- Dual serotonin and norepinephrine reuptake inhibitors (SNRIs)
- Tricyclic ADT
- Irreversible, non-selective monoamine oxidase inhibitors (IMAOs)
- α2-antagonists
- Agomelatine
- Tianeptine

Several treatments are considered as having an antidepressant action:

- In monotherapy: bupropion, selective and reversible IMAO-A, quetiapine
- In combination with an ADT: lithium, lamotrigine and second-generation antipsychotics

Some clinical characteristics are seen as quality evidence to guide the choice of an ADT, as follows:

- For Major Depressive Disorder with a significant aboulia, anhedonia, psychomotor retardation or fatigue: SNRI
- For Major Depressive Disorder with a significant weight loss or significant sleep disturbances: α2-antagonist
- For Major Depressive Disorder with a marked depressed mood: tricyclic ADT

The experts were questioned on tolerance profile of the ADT (Additional file 6).

#### Minimal duration of ADT

The experts recommended that patients should be maintained on the ongoing treatment with ADT for 6 months after achieving clinical remission. A longer-term treatment is recommended in cases with:

- The presence of a comorbid psychiatric disorder.
- The presence of psychotic symptoms.
- The presence of high suicidal risk.
- Resistance to at least one ADT at adequate duration and dose.
- A history of early relapse after the treatment is discontinued.
- A long period before reaching remission.
- A history of at least 2 previous episodes of depression.

### Pharmacological strategies in treatment-resistant depression

#### Switching strategies

Once the decision is made to switch from one ADT to another one, the clinician should consider how this strategy can be implemented. There are three major types of a ADT switch strategies that can be envisaged [13]:

- Concurrent switch: changes in the dose of both medications are implemented simultaneously. The new medication is gradually titrated upward while the current agent is gradually tapered downward.
- Overlapping switch: dose changes are only implemented for one medication at time, while holding the original medication constant at the original dose until the second medication has reached its optimal dose.
- Sequential switch: the dose of the current medication is titrated downward until the interruption. Then, the new medication is introduced.

The concurrent switch is recommended, except when the patient is currently receiving a monoamine oxidase inhibitor (MAOI) medication. In that case, the sequential approach is required during the switching process.

Switching strategies are recommended in the following indications:

- No response to the initial treatment
- Poor tolerance to the initial treatment
- Previous response to the newly introduced treatment

In the first-line, inter-class switch is recommended. The different molecules proposed according to the initial treatment are represented in Table 1.

**Table 1:**
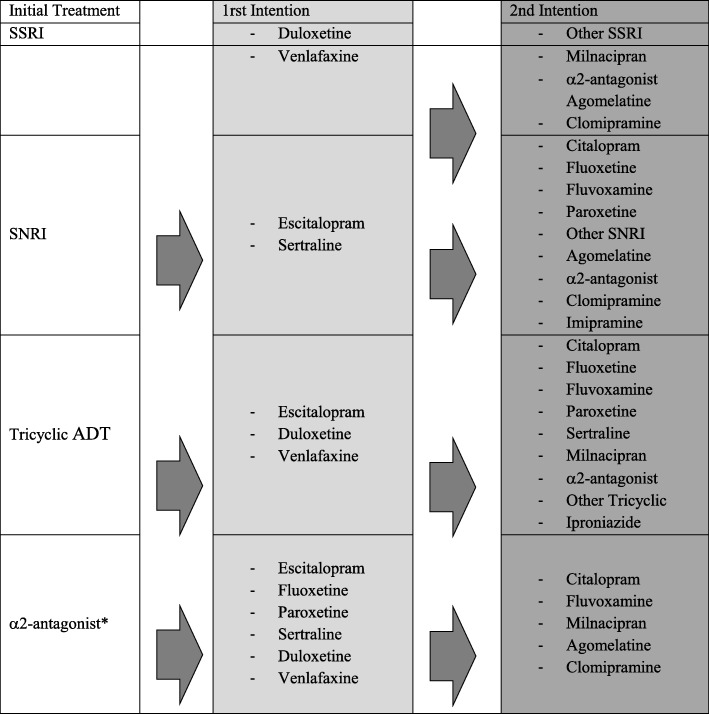
Recommendations for Switching ADT

* Switching from an α2-antagonist to another one is not recommended

#### Combination strategies

The combination strategy (i.e. adding another ADT to an existing one) is only recommended in cases of partial response, after 4 to 6 weeks of adequate treatment. In the first-line treatment, the recommended strategies consist of the following combinations:

- SSRI + α2 antagonist
- SNRI + α2 antagonist
- Tricyclic ADT + α2-antagonist

The recommended doses range from 15 to 30 mg per day for mirtazapine and 30 to 60 mg per day for mianserine. In the second-line treatment, an association between SSRIs, SNRIs or tricyclic ADT and agomelatine can be proposed. The maintenance of the combined ADT is recommended for a period of six months once clinical remission is obtained. A period of one year is not justified, except in specific indications.

#### Add-on strategies

The potentiation strategy is only recommended in cases of partial response, after 4 to 6 weeks of adequate treatment. Adding lithium or quetiapine to the ongoing ADT is recommended to enhance ADT efficacy (Table 2). The use of thyroid hormones or aripiprazole is proposed as a second intention. In this indication, second-generation antipsychotics such as risperidone, olanzapine, clozapine, amisulpride) or anticonvulsants (apart from lamotrigine) are not recommended.

**Table 2:** Recommendations for treatment potentiation

Potentiation Treatment	Target level:
1rst Intention	
•Lithium	0,5 à 0,8 mmol/L^a^
•Quetiapine	50 à 150 mg/day^b^
2nd Intention	
•Aripiprazole	2,5 à 10 mg/day^c^
•Tri-iodothyronine	25 à 50 μg/day^d^
•Lamotrigine	200 à 400 mg/day

^a^: A plasma level of less than 0, 4 mmol/L is not recommended in this indication

^b^: A dosage greater than 300 mg/day is not recommended in this indication

^c^: A dosage greater than 15 mg day is not recommended in this indication

^d^: Numeric trend (no consensus)

Thyroid hormone supplementation is recommended in combination with SNRIs or tricyclic ADT, and eventually with SSRIs or α2-antagonists. No consensus could be reached regarding the targeted doses, but the dose typically used for this purpose is 25 to 50 μg/day of L-T3. A pre-therapeutic assessment is recommended and includes:

- Physical examination
- Electrocardiogram
- Determination of Thyroid-Stimulating Hormone levels

In the absence of a consensus, the targeted dose should allow TSH levels ranging from 0.1 to 1 μg/L to be achieved.

### Strategies to prevent relapse

Treatment to prevent relapse is recommended from the first episode, and a preventive treatment of recurrences is proposed beyond the third episode. The presence of residual symptoms should be carefully considered leading to the prescription of a treatment in order to prevent recurrences from the first depressive episode.

Strategies considered to be effective for preventing recurrences are maintained with electroconvulsive therapy and lithium. In the second-line treatment, the experts proposed lamotrigine or quetiapine.

When full remission is obtained, the experts recommend:

- Regularly assessing treatment adherence
- Regularly assessing insertion and social functioning
- Regularly assessing the quality of life,
- Investigating possible illicit drug use.

The promotion of a regular physical activity and satisfactory food hygiene can be proposed as complementary alternatives for the prevention of recurrences.

### Organisation of sequenced treatment

#### First-line strategy

Two main criteria were proposed to the experts that could guide the selection of the appropriate therapeutic strategy: the intensity of the current depressive episode and associated clinical features.

##### Recommendations for mild, moderate and severe depression

- SSRIs and SNRIs are considered a first-line treatment, regardless of the clinical severity, without distinguishing between these types of ADT.
- For *severe* depression, psychotherapies are only recommended in combination with ADT, whereas they can be proposed in monotherapy in mild to moderate major depressive episodes.
- Tricyclic ADT, α2-antagonists or agomelatine can be proposed as a second-line treatment in severe depression.

##### Recommendations for clinical dimensions of major depressive disorder (Table 3)

No clinical features support the use of different ADT in combination in first-line.

**Table 3:** Recommendations for clinical dimensions of major depressive disorder

Dimension	First Intention	Second Intention
With marked anhedonia	SSRI or SNRI	α2-antagonist or agomelatine
With marked psychomotor retardation	SNRI. SSRI	Tricyclic or α2 antagonists
With marked sleep disturbances	SSRI or SNRI or α2-antagonist or agomelatine	Tricyclic ADT
With atypical features (hyperphagia, hypersomnia)	SSRI or SNRI	Tricyclic or agomelatine
With psychotic features	SNRI in monotherapy or SSRI in combination with an atypical antipsychotic	SSRI, tricyclic ADT or α2-antagonist, in monotherapy or in combination with AAP
With anxious features	SSRI or SNRI or α2 antagonist	Tricyclic ADT
With high suicidal risk	SSRI or SNRI or α2 antagonist	Tricyclic ADT or potentiation strategies with lithium or AAP

*AAP* Atypical Antipsychotic, *SNRI* Dual serotonin and norepinephrine reuptake inhibitors, *SSRI* Selective serotonin reuptake inhibitors

#### Second-, third-, fourth-, fifth- and sixth-line strategies

In the second-line treatment, in cases of partial response or non-response to ADT treatment, optimising the dose of the initial ADT is systematically recommended with a high priority level.

- *If a patient has a partial response to the first-line ADT*: association with an α2-antagonist is recommended irrespective of the class of the initial ADT (except for mianserine and mirtazapine)
- *If a patient has non-response to the first-line ADT*: switching strategies is recommended. Association of two ADT is not recommended in first intention.

Strategies recommended in the second-, third-, fourth-, fifth- and sixth-line treatments, which rely on the previous line of treatment, are summarised in Additional file 6: Figures S1 to S7).

### Psychotherapy

In the case of unipolar depression, psychological treatments intend to:

- Provide psychological support
- Inform the patient about the disease and its overall characteristics and management
- Help the patient to gain a greater understanding of their own psychopathology
- Increase therapeutic alliance and adherence to pharmacotherapy
- Develop coping strategies for stressful situations
- Improve psychosocial functioning and quality of life
- Manage psychiatric comorbidities, in particular addiction and anxiety disorders
- Teach the patient to evaluate mood and recognise evidence for the occurrence of early symptoms or clinical worsening

In the acute phase of MDD of mild to moderate intensity, the experts recommended (eventually in monotherapy):

- Cognitive behaviour therapy
- Supportive therapy
- Psychoeducational intervention

In moderate to severe MDD, only supportive therapy and psychoeducational intervention are recommended, systematically in combination with an ADT, regardless of the line of treatment.

When remission is obtained, it is necessary to continue or propose:

- Cognitive behaviour therapy
- Psychoeducational intervention
- Mindfulness therapy.

### Brain stimulation techniques

Among the available and effective brain stimulation techniques for the management of MDD, the scientific committee has preferentially chosen electroconvulsive therapy (ECT) and repetitive transcranial magnetic stimulation (rTMS), in monotherapy or in combination with the current ADT. To date, the applications of Vague Nerve Stimulation, Deep Brain stimulation or transcranial direct current stimulation are not sufficiently supported in the field of major depression.

#### ECT is proposed as an effective treatment in monotherapy or in combination to prevent the risk of relapse, while rTMS is not considered as having preventive properties

ECT is never recommended as a first-line treatment for the initial major depressive episode, irrespective of the clinical severity or clinical features; the same is true for rTMS.

Brain stimulation techniques should be reserved for situations of treatment resistance and recommended in the first intention, although only from the fourth line of treatment (after the failure of three adequately conducted ADT).

## Discussion

Despite the large variety of treatment options currently available for the management of MDD, many patients do not achieve a satisfactory improvement with adequate doses of ADTs given for sufficient duration, and are eventually classified as experiencing treatment resistance.

Besides the basic strategies for detecting, diagnosing, and treating TRD, the French recommendations in this guideline outline the potential for advanced strategies to be guided by the preceding lines of treatment and emphasise the significance of the systematic and rigorous evaluation of previous clinical responses prior to any treatment decision made in resistant/refractory cases.

Comparison between evidence-based and expert guidelines is complex, reflecting differences in methodology, in weight placed on the available evidence and, to some extent, in cultural traditions in treatment, attitudes towards or availability of particular pharmacological agents. The differences mainly concern the advantage given to one pharmacological option over the others and the hierarchical treatment preferences, although strategies (i.e. optimisation, switching, potentiation or combination) are similar.

Experts’ support for using optimisation as a first step following failure to respond to treatment is consistent with most EBGs and with studies on dose increase strategies documenting the efficacy of dose escalation [14–17]. So far, only the NICE guidelines have displayed some reserve in the general recommendations about dose-escalation [18]. The dose-response relationship varies between pharmacological classes, with beneficial effects reported with TCAs [19, 20], venlafaxine [19] and the IMAO tranylcypromine [19–21]. Evidence supporting the efficacy of dose increase for SSRIs is inconclusive [22–26]. However, optimisation may be a reasonable step, especially due to the inter-individual variability in the plasma concentration of ADT and associated uncertainty about the identification of patients that could probably benefit from high-dose medication.

A switch of the currently administered ADT within or across ADT classes is valuable at each step of the treatment, and our expert panel prioritises a switch across classes for an ADT with evidence of superior efficacy. This strategy is often employed in EBGs in cases of non-response, even though it is not yet fully substantiated by RCTs. Moreover, there is no clear proven advantage of one switch option over the others, even though there may be slightly higher remission rates for between-class than within-class switches [27]. The response rates after switching ADT, including to the same class, shows significant variation between studies (12–70%). A meta-analysis representing 1496 participants compared the switch from an SSRI with either a switch to another class of ADT or a second course of an SSRI, and found slight but significantly higher remission rates for the latter strategy (28% for the across-class switch versus 23.5% for the within-class switch [27]. Similarly, the results of the STAR*D level II study, which enrolled large numbers of patients in “real-world” clinical settings, have shown that citalopram non-responders achieved remission rates between 17.6 and 24.8% after switching to bupropion, sertraline or venlafaxine without any significant differences between the different agents [5]. However, in a large European multicentre study, Souery et al. [28] found no differences between across-class and within-class switches when analysing response and remission rates. At this point, switching to an ADT with some evidence of higher efficacy is recommended by the CANMAT, BAP, and WFSBP, especially in cases of non-response [14–17].

The combination of ADTs recommended by the experts is a commonly used strategy in daily clinical practice [29]. However, it should be considered carefully that the evidence for this option in TRD is limited. The literature has focused mainly on the augmentation of SRRIs with TCAs, mirtazapine or mianserin [30, 31], leading several EBGs to recommend concurrent medication with SSRIs or SNRIs and mirtazapine or mianserin. The combination of a TCA with an α2-antagonist recommended by the experts is not documented in the literature and deserves further study. Adding lithium to the ongoing ADT is recommended by the experts as a second intention after a partial response to the first-line treatment, whether it is an SSRI, SNRI or TCA, and in the second intention after non-response to a tricyclic ADT. Its use is consistently supported by treatment guidelines in TRD, and positioned by BAP in the first intention following the failure of the first ADT and in the second intention by the CANMAT after the failure of the first ADT, especially in cases of partial response [14, 15]. These differences are probably the result of a less common use of augmentation with lithium than AAP in clinical routine care, underlined by the need for continuous plasma level determinations and the long-term risks of thyroid, cardiovascular and renal adverse effects [29]. Overall, lithium effectively augments TCAs, although more evidence is needed before such definitive claims about its activity in combination with SSRIs or other first-line ADTs can be confirmed [32, 33].

AAPs were recognised as a second-line strategy in the second intention in partial responders, and as a fourth-line consideration in cases of non-response. The efficacy of the augmentation of ADT with AAP has been the focus of several RCTs and meta-analyses [34, 35]. However, experts’ recommendations were limited to quetiapine in the first intention and aripiprazole in the second intention, despite a high degree of perceived efficacy of other AAPs in several EBGs [14–17, 36]. Of note, quetiapine is the only AAP to have been previously studied under trial conditions in a head to head comparison with lithium, meaning at least comparable short-term effectiveness between the two treatments [37]. A network meta-analysis of 48 RCTs examined the comparative effects of 11 augmentation agents (aripiprazole, bupropion, buspirone, lamotrigine, lithium, methylphenidate, olanzapine, pindolol, quetiapine, risperidone, and thyroid hormone with each other and with placebo. While only aripiprazole, lithium, quetiapine, and T3 were more effective than placebo, quetiapine and aripiprazole appear to be the most robust evidence-based options [38]. Risperidone has been found to improve ADT responses in two, relatively short, RCT [39, 40] and in a meta-analysis [41], but has not yet received approval for that indication from the US Food and Drug Administration (FDA). It should also be noted that rather than strictly being an augmentation therapy, it is the proprietary combination of olanzapine and fluoxetine (referred to as OFC) that has been studied as a treatment for TRD and it has not been shown that olanzapine augments other ADTs.

T3 augmentation has not been extensively studied, despite no significant differences with regard to the response rates in comparison to lithium in the STAR*D study [42]. Add-on treatment with lamotrigine proposed by the experts is support by one retrospective chart review suggesting that this strategy could be efficacious and well tolerated [43]. Furthermore it could be for a subset of patients suffering from very severe depressive symptoms [44].

It is important to note that for most people with TRD, a combination of pharmacological and psychological approaches may be the most effective treatment both in terms of acute response and relapse prevention. Several guidelines propose to consider Cognitive Behavioral Therapy, Interpersonal therapy or Mindfulness-based cognitive therapy [18, 45] and Behavioral activation [18] in combination or as an alternative to pharmacotherapy in cases of non- or partial response. However, high-quality studies that specifically sought to examine the effectiveness of psychotherapeutic treatments for TRD are scarce. In a recent meta-analysis investigating the effectiveness of psychotherapy for TRD, van Bronswijk and colleagues [46] found no evidence to conclude that there is a significant benefit of psychotherapy as compared with treatment as usual (TAU) (i.e. pharmacotherapy). However, they reported a moderate general effect size of 0.42 (95% CI 0.29–0.54) in favor of psychotherapy plus TAU. Moreover, there analysis revealed no significant differences in the efficacy between the most frequently investigated psychological interventions (i.e. cognitive behavior therapy, mindfulness-based cognitive therapy, cognitive behavioral analysis system of psychotherapy, and interpersonal psychotherapy). This meta-analysis also provided evidence for a positive association between baseline severity as well as group versus individual therapy format with the treatment effect. Comparisons of different psychological treatments is complex and the choice of a specific type of psychological treatment should notably consider availability and patient preference [17].

Since little comparative data between these strategies exist, it is important to consider side-effect burden, partial response, and previous medication history when deciding between strategies. According to the British Association for Psychopharmacology and the CANMAT, the decision between switching and adjunctive strategies should be individualised based on clinical factors including the tolerance of the current ADT, the number of previously failed treatment, the severity of the illness, patient preferences and partial/insufficient response on the current ADT [14, 15]. The experts incorporate an additional dimension with consideration of the previous line of treatment. Although their recommendations can meet the clinical needs for most patients, they cannot replace clinical judgement, and tailored choices about care must carefully be considered; the overall characteristics of each individual patient should also be incorporated. Importantly, the availability of ADTs and other compounds investigated as potentiators of ADT varies across countries (i.e., not all agents are benefited from worldwide approval for the treatment of MDD), leading to some discrepancies in daily availability and use patterns.

## Conclusion

By integrating the more updated scientific knowledge with everyday clinical practice and patient-specific factors, EBG and CBG contribute to significantly facilitate and guide treatment decisions and choices for those clinicians involved actively in the assessment and management of TRD. While there are numerous first-line treatment strategies for depressed patients, there is, in contrast, a paucity of information regarding the best approaches to adopt when the first-line treatment is unsuccessful. Therefore, CBG methodologies allow the identification of strategies in areas in which EBG recommendations are generally nonspecific due to their particular reliance on evidence bases. Experts’ behaviours are important to assess inasmuch as they may help to identify optimal successive treatment steps and to tailor individualised treatment recommendations, a shortcoming for all of the established guidelines.

Well-designed clinical trials based on monotherapy and adjunctive strategies with other pharmacological agents and psychotherapy are still required in order to better identify the most appropriate strategies in TRD. Complementary researches are needed to determine specific markers and develop quantifiable measures to assess biobehavioural factors associated with TRD. The implementation of such variables in clinical practice could assist in guiding optimal care targeting specific vulnerable subgroups and planning short- and long-term treatments through relevant staging models. In the area of personalised medicine, the complementary use of EBGs and CGBs should be able to provide scientific guidance that is helpful for clinicians in routine clinical practice.

## Additional files

Additional file 1: organisation, expert panel, questionnaire development and data analysis. (DOCX 21 kb)

Additional file 2: Questionnaire. (DOCX 1090 kb)

Additional file 3: Definitions (*According to the American College of Neuropsychopharmacology*) [47]. (DOCX 14 kb)

Additional file 4: Antidepressant treatments classified according to the pharmacological profile. (DOCX 14 kb)

Additional file 5: Expert Panel: description. (DOCX 14 kb)

Additional file 6: Tolerance profile of the antidepressants. (DOCX 147 kb)

## Data Availability

All data generated or analyzed during this study are included in this published article and its supplementary information files.

## Abbreviations

AAP: Atypical Antipsychotic
ADT: Antidepressant
AFBPN: Association for Biological Psychiatry and Neuropsychopharmacology
CBG: Clinical-based guidelines
EBG: Evidence-based guidelines
ECT: Electroconvulsive therapy
MAO: Monoamine oxidase inhibitors
MDD: Major depressive disorder
RCT: Randomized controlled trials
rTMS: repetitive transcranial magnetic stimulation
SNRI: dual serotonin and norepinephrine reuptake inhibitors
SSRI: Selective serotonin reuptake inhibitors
TRD: Treatment-resistant depression
