# Supplementary material for: Clinical guidelines for the management of treatment-resistant depression: French recommendations from experts, the French Association for Biological Psychiatry and Neuropsychopharmacology and the fondation FondaMental
Source: BMC Psychiatry. 2019 Aug 28;19:262. doi: 10.1186/s12888-019-2237-x (PMC6712810; doi:10.1186/s12888-019-2237-x)
Supplement: Supplementary file 1 — organisation, expert panel, questionnaire development and data analysis. (DOCX 21 kb) [file 12888_2019_2237_MOESM1_ESM.docx]

**Additional file 1: organisation, expert panel, questionnaire development and data analysis**

**Organization**

The organization committee initiated the development of French Recommendations in treatment-resistant depression. It comprises a scientific committee in charge of expert’s selection, work schedule implementation, and clinical focus identification. The scientific committee was in charge of data analysis and interpretation of the results of the survey, and development and promotion of recommendations. At the same time, the organization committee mandated two independent committees responsible for guaranteeing the methodological rigor regarding questionnaire construction and recommendations development, respectively.

| **Organization committee** |
| --- |
| Professor AOUIZERATE Bruno (Bordeaux)  Professor COURTET Philippe (Montpellier)  Professor HAFFEN Emmanuel (Besançon)  Professor LEBOYER Marion (Créteil)  Professor LLORCA Pierre-Michel (Clermont-Ferrand) |
| **Scientific committee** |
| Coordination: Doctor CHARPEAUD Thomas (Clermont-Ferrand)  Professor AOUIZERATE Bruno (Bordeaux)  Professor COURTET Philippe (Montpellier)  Professor EL-HAGE Wissam (Tours)  Doctor GENTY Jean-Baptiste (Clermont-Ferrand)  Professor HAFFEN Emmanuel (Besançon)  Professor LLORCA Pierre-Michel (Clermont-Ferrand)  Doctor YRONDI Antoine (Clermont-Ferrand) |
| **Independent** **committee in charge of** **proofreading the questionnaire** |
| Doctor BELZEAUX Raoul (Marseille)  Doctor ETAIN Bruno (Créteil)  Doctor GEOFFROY Pierre-Alexis (Paris)  Doctor OLIE Emilie (Montpellier) |
| **Independent committee in charge of proofreading the recommendations** |
| Professor FAKRA Eric (Saint-Etienne)  Professor FOSSATI Philippe (Paris)  Professor ROUILLON Fréderic (Paris)  Professor THOMAS Pierre (Lille) |

**Questionnaire development**

At an early stage, the scientific committee conducted data synthesis and analysis concerning the definition, assessment and treatment of resistant depression (CT, AB, CP, EW, GJB, HE, LPM, YA). A literature search using the keywords « « major depressive disorder », « treatment resistant depression » and « guideline » was performed in Pubmed to identify all relevant studies.

Based on this approach, a questionnaire consisting of 118 questions was drafted, then proofread and corrected by an independent scientific committee (BR, BE, GPA, OE). The 118 questions were regrouped into 3 main areas that were judged as essential:

- Assessment of pharmacological resistance and situations at risk of resistance (questions 1 to 10)
- Pharmacological and psychological strategies (questions 11 to 89)
- Specific populations/clinical situations: strategies in elderly patients, subjects with comorbid anxiety disorders, substance use disorders or personality disorders (questions 90 to 118)

This questionnaire was completed by an experts' panel who could express the level of agreement or disagreement for each formulated question. Each expert answered each question with a graduated scale from 0 to 9 derived from a variation of the “Nominal Group” method, developed by the Rand Corporation and the University of California in the USA (“RAND/UCLA appropriateness rating method”), 0 meaning a “total disagreement” or “a formal contraindication” and 9 indicating a “total agreement” or “a formal indication”.

For example, for pharmacotherapy or therapeutic strategies:

- **9 :** **Extremely appropriate**: treatment or strategy of choice
- **7-8 : Usually appropriate**: first-line treatment or strategy, frequently used
- **4-5-6**: Second-line treatment or strategy, sometimes used (preference of the patient or family, ineffective or unsuitable first-line treatment or strategy)
- **1-2-3 : Usually inappropriate**: rarely used treatment or strategy
- **0 : Totally inappropriate**: never used treatment or strategy
- **NA :** **Not Applicable,** **possible answer when the question did not correspond to the current practice**

**Expert panel: Selection**

Since 2015, the Scientific Committee has invited 126 French psychiatrists to participate in this study. To identify them, the national network of expert centers for treatment resistant depression (CEDR) of the FondaMental Foundation, covering the whole French territory, was mandated to preselect potential experts who did not belong to the network themselves.

The selection criteria defined by the Scientific Committee were:

- Number of years of practice ≥ 5
- Number of patients currently seen for unipolar depression ≥ 30 per month in average or representing more than 30% of the daily clinical activity in psychiatry
- Participation in research projects and/or publication(s) and/ or communication(s) of research work in the field of unipolar depression over the last five years

Based on these criteria, 57 experts were identified. Among them, 36 completed the questionnaire between April 2015 and February 2016, representing 63% of those psychiatrists contacted.

**Data analysis**

The responses to the different questions or propositions were qualitatively and quantitatively analyzed (number of answers, median, mean, standard deviation, minimum, maximum) (Table 1).

- First intention treatment/strategy was defined if at least 50% of the answers to the question were in the zone 7–9 and less than 20% were in the zone 0.
- Second intention treatment/strategy was defined if less than 50% of the answers to the question were in the zone 7–9, at least 50% were in the cumulated zones 7–9 and 4–6, and less than 20% were in the zone 0.
- Third intention treatment/strategy was defined if less than 50% of the answers to the question were in the cumulated zones 7–9 and 4–6, and less than 20% were in the zone 0.
- In all cases, if the percentage of answers in the zone 0 was ≥20% and < 50%, the question was left pending.
- Contraindication was defined if at least 50% of the answers were in the zone 0.
- For all other cases the question was considered as non-consensual and the results were interpreted by the scientific committee and confronted with the literature, leading to the development of the recommendations:
  - If 50% of the answers to the question were in the zone 7–9 and 50% in the zone 4–6
  - If 50% of the answers to the question were in the zone 7–9 and 4–6 and 50% in the zone 1–3
  - For all other cases

| **Percentage of answers in the zones** | | | | |
| --- | --- | --- | --- | --- |
| **[0]** | **[1 - 3]** | **[4 - 6]** | **[7 - 9]** |  |
| < 20% | **-** | < 50% | **≥ 50%** | 🡺 First line |
| < 20% | < 50% | **≥ 50%** (< 50% in the zone [7 to 9] ) | | 🡺 Second-line |
| < 20% | **-** | < 50% | | 🡺 Third-line |
| **≥ 50 %** | **-** | **-** | **-** | 🡺 Contraindication |
| **-** | **-** | 50% | 50% | 🡺 Discussion in the Committee |
| **-** | 50% | 50% | |  |
| Other cases | | | |  |

**Data Analysis.** *For all other cases (−), the question was considered as non-consensual. The following rules were used to conclude the analyzed therapeutic strategy*
