# Supplementary material for: Clinical guidelines for the management of treatment-resistant depression: French recommendations from experts, the French Association for Biological Psychiatry and Neuropsychopharmacology and the fondation FondaMental
Source: BMC Psychiatry. 2019 Aug 28;19:262. doi: 10.1186/s12888-019-2237-x (PMC6712810; doi:10.1186/s12888-019-2237-x)
Supplement: Supplementary file 2 — Questionnaire. (DOCX 1090 kb) [file 12888_2019_2237_MOESM2_ESM.docx]

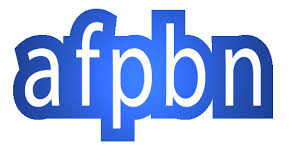
­­­­­
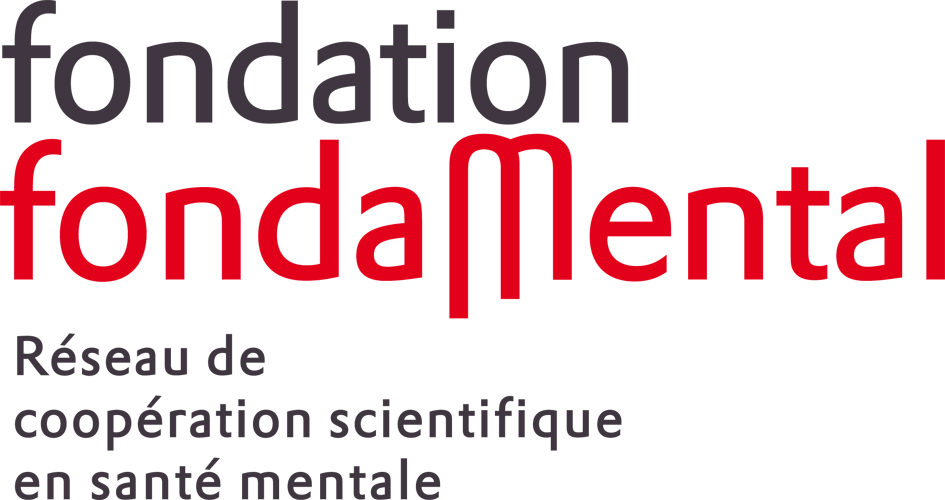


**Recommendations from experts**

**Management of treatment-resistant depression**

**EXPERT QUESTIONNARY**

Expert centers for treatment-resistant depression **(fondation FondaMental)**

- 1. **Assessments of treatment-resistant depression and at-risk situations**

# I. Évaluation de la résistance et des situations à risque de résistance

**Question 1**

0 means that this tool is never useful in daily practice

1, 2, 3 means that this tool is not very useful and should only be used in case of clinical orientation

4, 5, 6 means that this tool may be useful and may be used in daily practice

7, 8, 9 means that this tool is necessary and must be used in daily practice

NA means that you have no opinion and/or experience with this tool

| 1 | Among the following tools, which ones do you recommend in the daily practice for the management of patients under the age of 65 years of age, suffering from major depressive episodes?  *Circle the number corresponding to your choice* |
| --- | --- |

| 1A | A mood diagram | 0 | 1 | 2 | 3 | 4 | 5 | 6 | 7 | 8 | 9 | NA |
| --- | --- | --- | --- | --- | --- | --- | --- | --- | --- | --- | --- | --- |
| 1B | A self-rated scale of depression severity (BDI, QIDS-SR) | 0 | 1 | 2 | 3 | 4 | 5 | 6 | 7 | 8 | 9 | NA |
| 1C | A clinician-rated scale of depression severity (MADRS, Hamilton-D, QIDS…) | 0 | 1 | 2 | 3 | 4 | 5 | 6 | 7 | 8 | 9 | NA |
| 1D | A hypomania rating scale (Angst, MDQ, HCL…) | 0 | 1 | 2 | 3 | 4 | 5 | 6 | 7 | 8 | 9 | NA |
| 1E | A structured diagnostic interview (MINI…) | 0 | 1 | 2 | 3 | 4 | 5 | 6 | 7 | 8 | 9 | NA |
| 1F | A specific questionnaire exploring self-esteem (Rosenberg) | 0 | 1 | 2 | 3 | 4 | 5 | 6 | 7 | 8 | 9 | NA |
| 1G | A specific questionnaire exploring anxiety disorders (Hamilton A, COVI…) | 0 | 1 | 2 | 3 | 4 | 5 | 6 | 7 | 8 | 9 | NA |
| 1H | A scale to measure dimensions of personality (NEO PI-R, Big-Five Inventory) | 0 | 1 | 2 | 3 | 4 | 5 | 6 | 7 | 8 | 9 | NA |
| 1I | A suicide rating scale (C-SSRS, Beck Suicide Ideator Scale…) | 0 | 1 | 2 | 3 | 4 | 5 | 6 | 7 | 8 | 9 | NA |
| 1J | A specific scale exploring sleep disorders (Epworth, PSQI…) | 0 | 1 | 2 | 3 | 4 | 5 | 6 | 7 | 8 | 9 | NA |
| 1K | A specific scale exploring mood reativity (MAThyS…) | 0 | 1 | 2 | 3 | 4 | 5 | 6 | 7 | 8 | 9 | NA |
| 1L | Another tool : …………………………………………………………… | 0 | 1 | 2 | 3 | 4 | 5 | 6 | 7 | 8 | 9 | NA |
| 1M | None | 0 | 1 | 2 | 3 | 4 | 5 | 6 | 7 | 8 | 9 | NA |

| 2 | In your opinion, what the optimal minimum duration of the antidepressant treatment, once the target dose has been obtained, to judge the clinical efficacy in the outpatient treatment of unipolar depressive episode in an adult under 65 years of age?  *Circle the number corresponding to your choice (only one possible answer)* |
| --- | --- |

| 2A | 1 week |  |
| --- | --- | --- |
| 2B | 2 weeks |  |
| 2C | 3 weeks |  |
| 2D | 4 weeks |  |
| 2E | 5 weeks |  |
| 2F | 6 weeks |  |
| 2G | 7 weeks |  |
| 2H | 8 weeks |  |
| 2I | 3 months |  |
| 2J | 6 months |  |
| 2K | 9 months |  |
| 2L | 12 months |  |

**Question n 3**

0 means that this criterion is never a criterion for hospitalization.

1, 2, 3 means that this criterion can rarely be a criterion for hospitalization.

4, 5, 6 means that this criterion is most often a criterion for hospitalization.

7, 8, 9 means that this criterion is always a criterion for hospitalization.

NA means that you have no opinion on this criterion.

| 3 | In your opinion, what are the criteria for hospitalization in the case of unipolar depressive episode in an adult under the age of 65 years of age?  *Circle the number corresponding to your choice* |
| --- | --- |

| 3A | A high suicidal risk | 0 | 1 | 2 | 3 | 4 | 5 | 6 | 7 | 8 | 9 | NA |
| --- | --- | --- | --- | --- | --- | --- | --- | --- | --- | --- | --- | --- |
| 3B | The presence of psychotic symptoms | 0 | 1 | 2 | 3 | 4 | 5 | 6 | 7 | 8 | 9 | NA |
| 3C | The risk of poor adherence to treatment | 0 | 1 | 2 | 3 | 4 | 5 | 6 | 7 | 8 | 9 | NA |
| 3D | The failure of one antidepressant treatment | 0 | 1 | 2 | 3 | 4 | 5 | 6 | 7 | 8 | 9 | NA |
| 3E | The failure of two antidepressant treatments | 0 | 1 | 2 | 3 | 4 | 5 | 6 | 7 | 8 | 9 | NA |
| 3F | The failure of three antidepressant treatments | 0 | 1 | 2 | 3 | 4 | 5 | 6 | 7 | 8 | 9 | NA |
| 3G | The comorbidity with a severe medical condition (diabetes, COPD…) | 0 | 1 | 2 | 3 | 4 | 5 | 6 | 7 | 8 | 9 | NA |
| 3H | The intolerance to current medication | 0 | 1 | 2 | 3 | 4 | 5 | 6 | 7 | 8 | 9 | NA |
| 3I | The co-occurrence with other psychiatric disorders (anxiety disorders, substance abuse, personality disorder) | 0 | 1 | 2 | 3 | 4 | 5 | 6 | 7 | 8 | 9 | NA |
| 3J | The lack of adequate familial support | 0 | 1 | 2 | 3 | 4 | 5 | 6 | 7 | 8 | 9 | NA |
| 3K | The need for electroconvulsive therapy | 0 | 1 | 2 | 3 | 4 | 5 | 6 | 7 | 8 | 9 | NA |
| 3L | The need for transcranial magnetic stimulation | 0 | 1 | 2 | 3 | 4 | 5 | 6 | 7 | 8 | 9 | NA |
| 3M | The need for transcranial direct current stimulation | 0 | 1 | 2 | 3 | 4 | 5 | 6 | 7 | 8 | 9 | NA |
| 3N | The need for a tricyclic antidepressant | 0 | 1 | 2 | 3 | 4 | 5 | 6 | 7 | 8 | 9 | NA |
| 3O | The need for non-selective monoamine oxidase inhibitors | 0 | 1 | 2 | 3 | 4 | 5 | 6 | 7 | 8 | 9 | NA |
| 3P | The need for lithium | 0 | 1 | 2 | 3 | 4 | 5 | 6 | 7 | 8 | 9 | NA |
| 3Q | The need for thyroid hormones | 0 | 1 | 2 | 3 | 4 | 5 | 6 | 7 | 8 | 9 | NA |
| 3R | The need for a combination strategy | 0 | 1 | 2 | 3 | 4 | 5 | 6 | 7 | 8 | 9 | NA |
| 3S | The need for pramipexole | 0 | 1 | 2 | 3 | 4 | 5 | 6 | 7 | 8 | 9 | NA |
| 3T | The need for second generation antipsychotic | 0 | 1 | 2 | 3 | 4 | 5 | 6 | 7 | 8 | 9 | NA |
| 3U | The need for benzodiazepines withdrawal | 0 | 1 | 2 | 3 | 4 | 5 | 6 | 7 | 8 | 9 | NA |
| 3V | Other reasons :  ………………………………………………………………………………… | 0 | 1 | 2 | 3 | 4 | 5 | 6 | 7 | 8 | 9 | NA |

**Question 4**

This question aims to determine the place of treatments, including non-pharmacological treatments (psychotherapy, brain stimulation) in the definition of resistant depression.

| 4 | Which of the following proposals do you think best corresponds to the definition of a treatment resistant depressive episode?  *Circle the number corresponding to your choice (only one possible answer)* |
| --- | --- |

| 4A | The failure of one well-conducted line of treatment including non-pharmacological treatment |  |
| --- | --- | --- |
| 4B | The failure of one well-conducted line of treatment including non-pharmacological treatment |  |
| 4C | The failure of one well-conducted line of treatment including non-pharmacological treatment |  |
| 4D | The failure of one pharmacological treatment of adequate duration and dose |  |
| 4E | The failure of two pharmacological treatments of adequate duration and dose |  |
| 4F | The failure of three pharmacological treatments of adequate duration and dose |  |
| 4G | The failure of one tricyclic treatment of adequate duration and dose |  |
| 4H | The failure of one non-selective irreversible MAOI of adequate duration and dose |  |
| 4I | The failure of ECT |  |

**Question 5**

0 means that this feature is never predictive of a risk of resistance to pharmacological treatment.

1, 2, 3 means that this feature is rarely predictive of a risk of resistance to pharmacological treatment.

4, 5, 6 means that this feature is often predictive of a risk of resistance to pharmacological treatment.

7, 8, 9 means that this feature is always predictive of a risk of resistance to pharmacological treatment.

NA means that you have no opinion and/or experience with this feature

| 5 | Which of the following “lifetime” features are predictive of a treatment resistance risk for in an adult patient under 65 years of age suffering from unipolar depressive episode?  *Circle the number corresponding to your choice* |
| --- | --- |

| 5A | Comorbid substance abuse | 0 | 1 | 2 | 3 | 4 | 5 | 6 | 7 | 8 | 9 | NA |
| --- | --- | --- | --- | --- | --- | --- | --- | --- | --- | --- | --- | --- |
| 5B | Comorbid anxiety disorder | 0 | 1 | 2 | 3 | 4 | 5 | 6 | 7 | 8 | 9 | NA |
| 5C | Comorbid non-psychiatric chronic and organic disease | 0 | 1 | 2 | 3 | 4 | 5 | 6 | 7 | 8 | 9 | NA |
| 5D | Comorbid personality disorders (DSM cluster A: paranoid, schizoid, schizotypal)) | 0 | 1 | 2 | 3 | 4 | 5 | 6 | 7 | 8 | 9 | NA |
| 5E | Comorbid personality disorders (DSM cluster B: antisocial, borderline, histrionic, narcissistic) | 0 | 1 | 2 | 3 | 4 | 5 | 6 | 7 | 8 | 9 | NA |
| 5F | Comorbid personality disorders DSM (cluster C: avoidant, dependent, obsessive-compulsive) | 0 | 1 | 2 | 3 | 4 | 5 | 6 | 7 | 8 | 9 | NA |
| 5G | A history of one depressive episode | 0 | 1 | 2 | 3 | 4 | 5 | 6 | 7 | 8 | 9 | NA |
| 5H | A history of two depressive episode | 0 | 1 | 2 | 3 | 4 | 5 | 6 | 7 | 8 | 9 | NA |
| 5I | A history of one treatment resistant depressive episode | 0 | 1 | 2 | 3 | 4 | 5 | 6 | 7 | 8 | 9 | NA |
| 5J | Family history of depressive episodes | 0 | 1 | 2 | 3 | 4 | 5 | 6 | 7 | 8 | 9 | NA |
| 5K | A history of psychological trauma | 0 | 1 | 2 | 3 | 4 | 5 | 6 | 7 | 8 | 9 | NA |
| 5L | Separated or divorced status | 0 | 1 | 2 | 3 | 4 | 5 | 6 | 7 | 8 | 9 | NA |
| 5M | A low socioeconomic level | 0 | 1 | 2 | 3 | 4 | 5 | 6 | 7 | 8 | 9 | NA |

**Question 6**

0 means that this feature is never predictive of a risk of resistance to pharmacological treatment.

1, 2, 3 means that this feature is rarely predictive of a risk of resistance to pharmacological treatment..

4, 5, 6 means that this feature is often predictive of a risk of resistance to pharmacological treatment.

7, 8, 9 means that this feature is always predictive of a risk of resistance to pharmacological treatment.

NA means that you have no opinion and/or experience with this feature

| 6 | In your opinion, which of the features of the current depressive episode are predictive of a risk of treatment resistance in an adult under 65 years of age?  *Circle the number corresponding to your choice* |
| --- | --- |

| 6A | The duration of the episode | 0 | 1 | 2 | 3 | 4 | 5 | 6 | 7 | 8 | 9 | NA |
| --- | --- | --- | --- | --- | --- | --- | --- | --- | --- | --- | --- | --- |
| 6B | The duration of the untreated episode | 0 | 1 | 2 | 3 | 4 | 5 | 6 | 7 | 8 | 9 | NA |
| 6C | The early onset (before 20 ans) | 0 | 1 | 2 | 3 | 4 | 5 | 6 | 7 | 8 | 9 | NA |
| 6D | The late onset (after 60 ans) | 0 | 1 | 2 | 3 | 4 | 5 | 6 | 7 | 8 | 9 | NA |
| 6E | The illness severity | 0 | 1 | 2 | 3 | 4 | 5 | 6 | 7 | 8 | 9 | NA |
| 6F | The onset during the peri-menopausal period | 0 | 1 | 2 | 3 | 4 | 5 | 6 | 7 | 8 | 9 | NA |
| 6G | The occurrence in response to a stressful event | 0 | 1 | 2 | 3 | 4 | 5 | 6 | 7 | 8 | 9 | NA |
| 6H | The apparent absence of precipitating factor | 0 | 1 | 2 | 3 | 4 | 5 | 6 | 7 | 8 | 9 | NA |
| 6I | The presence of marked anxious symptoms | 0 | 1 | 2 | 3 | 4 | 5 | 6 | 7 | 8 | 9 | NA |
| 6J | The presence of marked atypical symptoms (hypersomnia, hyperphagia) | 0 | 1 | 2 | 3 | 4 | 5 | 6 | 7 | 8 | 9 | NA |
| 6K | The presence of psychotic symptoms | 0 | 1 | 2 | 3 | 4 | 5 | 6 | 7 | 8 | 9 | NA |
| 6L | The presence of marked psychomotor retardation | 0 | 1 | 2 | 3 | 4 | 5 | 6 | 7 | 8 | 9 | NA |
| 6M | The occurrence in the autumn / winter period | 0 | 1 | 2 | 3 | 4 | 5 | 6 | 7 | 8 | 9 | NA |

**Question 7**

0 means that this disease never interferes with clinical improvement.

1, 2, 3 means that this disease interferes with clinical improvement in rare cases.

4, 5, 6 means that this disease often interferes with clinical improvement.

7, 8, 9 means that this disease always interferes with clinical improvement.

NA means that you have no opinion/experience with this disease.

| 7 | Which of the following organic diseases may interfere with the clinical improvement of a patient under 65 years of age with unipolar depressive episode treated with an antidepressant?  *Circle the number corresponding to your choice* |
| --- | --- |

| 7A | Neurodegenerative disease (multiple sclerosis, Parkinson disease…) | 0 | 1 | 2 | 3 | 4 | 5 | 6 | 7 | 8 | 9 | NA |
| --- | --- | --- | --- | --- | --- | --- | --- | --- | --- | --- | --- | --- |
| 7B | Migraines | 0 | 1 | 2 | 3 | 4 | 5 | 6 | 7 | 8 | 9 | NA |
| 7C | Neurovascular disease | 0 | 1 | 2 | 3 | 4 | 5 | 6 | 7 | 8 | 9 | NA |
| 7D | Coronary disease | 0 | 1 | 2 | 3 | 4 | 5 | 6 | 7 | 8 | 9 | NA |
| 7E | Hypertensive disease | 0 | 1 | 2 | 3 | 4 | 5 | 6 | 7 | 8 | 9 | NA |
| 7F | Venous thromboembolic disease | 0 | 1 | 2 | 3 | 4 | 5 | 6 | 7 | 8 | 9 | NA |
| 7G | Pulmonary disease (COBP, asthma…) | 0 | 1 | 2 | 3 | 4 | 5 | 6 | 7 | 8 | 9 | NA |
| 7H | Metabolic disease (diabetes, obesity…) | 0 | 1 | 2 | 3 | 4 | 5 | 6 | 7 | 8 | 9 | NA |
| 7I | Endocrine disease (thyroid, adrenal…) | 0 | 1 | 2 | 3 | 4 | 5 | 6 | 7 | 8 | 9 | NA |
| 7J | Cancer | 0 | 1 | 2 | 3 | 4 | 5 | 6 | 7 | 8 | 9 | NA |
| 7K | Auto-immune disease (lupus, rheumatoid arthritis, Crohn’s disease …) | 0 | 1 | 2 | 3 | 4 | 5 | 6 | 7 | 8 | 9 | NA |
| 7L | Dermatological disease (psoriasis, eczema…) | 0 | 1 | 2 | 3 | 4 | 5 | 6 | 7 | 8 | 9 | NA |
| 7M | Other : ……………………………………………………… | 0 | 1 | 2 | 3 | 4 | 5 | 6 | 7 | 8 | 9 | NA |

**Question 8**

0 means that this medication never interferes with clinical improvement.

1, 2, 3 means that this medication interferes with clinical improvement in rare cases.

4, 5, 6 means that this medication often interferes with clinical improvement.

7, 8, 9 means that this medication always interferes with clinical improvement.

NA means that you have no opinion/experience with this medication.

| 8 | Which of the following medications may interfere with the clinical improvement of a patient under the age of 65 years of age suffering from unipolar depressive episode treated with an antidepressant treatment?  *Circle the number corresponding to your choice* |
| --- | --- |

| 8A | Corticoid treatment | 0 | 1 | 2 | 3 | 4 | 5 | 6 | 7 | 8 | 9 | NA |
| --- | --- | --- | --- | --- | --- | --- | --- | --- | --- | --- | --- | --- |
| 8B | Treatment with calcium-channel blocker | 0 | 1 | 2 | 3 | 4 | 5 | 6 | 7 | 8 | 9 | NA |
| 8C | Angiotensin-converting enzyme inhibitors | 0 | 1 | 2 | 3 | 4 | 5 | 6 | 7 | 8 | 9 | NA |
| 8D | Beta-blocker | 0 | 1 | 2 | 3 | 4 | 5 | 6 | 7 | 8 | 9 | NA |
| 8E | Interferon therapy | 0 | 1 | 2 | 3 | 4 | 5 | 6 | 7 | 8 | 9 | NA |
| 8F | First generation antipsychotic (e.g Cyamemazine, Haloperidol) | 0 | 1 | 2 | 3 | 4 | 5 | 6 | 7 | 8 | 9 | NA |
| 8G | Second generation antipsychotic (e.g Risperidone; Olanzapine) | 0 | 1 | 2 | 3 | 4 | 5 | 6 | 7 | 8 | 9 | NA |
| 8H | Valproic acid derivatives (e.g Divalproex sodium) | 0 | 1 | 2 | 3 | 4 | 5 | 6 | 7 | 8 | 9 | NA |
| 8I | Carbamazepine | 0 | 1 | 2 | 3 | 4 | 5 | 6 | 7 | 8 | 9 | NA |
| 8J | Topiramate | 0 | 1 | 2 | 3 | 4 | 5 | 6 | 7 | 8 | 9 | NA |
| 8K | Gabapentin | 0 | 1 | 2 | 3 | 4 | 5 | 6 | 7 | 8 | 9 | NA |
| 8L | Isotretinoin | 0 | 1 | 2 | 3 | 4 | 5 | 6 | 7 | 8 | 9 | NA |
| 8M | Lithium | 0 | 1 | 2 | 3 | 4 | 5 | 6 | 7 | 8 | 9 | NA |
| 8N | Lamotrigine | 0 | 1 | 2 | 3 | 4 | 5 | 6 | 7 | 8 | 9 | NA |
| 8O | Continuous treatment with benzodiazepines | 0 | 1 | 2 | 3 | 4 | 5 | 6 | 7 | 8 | 9 | NA |
| 8P | Other : ………………………………………………………… | 0 | 1 | 2 | 3 | 4 | 5 | 6 | 7 | 8 | 9 | NA |

**Question 9**

0 means that this paraclinical examination is never indicate in daily practice.

1, 2, 3 means that this paraclinical examination is rarely indicate in daily practice.

4, 5, 6 means tthat his paraclinical examination is often indicate in daily practice.

7, 8, 9 means tthat his paraclinical examination is always indicate in daily practice.

NA means you have no opinion/experience concerning this paraclinical examination.

| 9 | A patient with no significant history, under 65 years of age, suffers from a depressive episode, with a failure of two antidepressants treatment at adequate dosage and duration.  Do you plan further examinations?  Yes  No  If so, which ones will you perform in daily clinical practice among those listed below?  *Circle the number corresponding to your choice* |
| --- | --- |

| 9A | Brain scan | 0 | 1 | 2 | 3 | 4 | 5 | 6 | 7 | 8 | 9 | NA |
| --- | --- | --- | --- | --- | --- | --- | --- | --- | --- | --- | --- | --- |
| 9B | Brain MRI | 0 | 1 | 2 | 3 | 4 | 5 | 6 | 7 | 8 | 9 | NA |
| 9C | Electroencephalography | 0 | 1 | 2 | 3 | 4 | 5 | 6 | 7 | 8 | 9 | NA |
| 9D | Complete blood count, blood electrolytes, liver and renal functions | 0 | 1 | 2 | 3 | 4 | 5 | 6 | 7 | 8 | 9 | NA |
| 9E | Lipid profile (cholesterol, triglyceride) and glucose levels | 0 | 1 | 2 | 3 | 4 | 5 | 6 | 7 | 8 | 9 | NA |
| 9F | Thyroid-Stimulating Hormone levels (TSHus) | 0 | 1 | 2 | 3 | 4 | 5 | 6 | 7 | 8 | 9 | NA |
| 9G | Free T3 and T4 levels | 0 | 1 | 2 | 3 | 4 | 5 | 6 | 7 | 8 | 9 | NA |
| 9H | Carbohydrate Deficient Transferrin levels | 0 | 1 | 2 | 3 | 4 | 5 | 6 | 7 | 8 | 9 | NA |
| 9I | Pharmacogenetic testing for CYP enzymes | 0 | 1 | 2 | 3 | 4 | 5 | 6 | 7 | 8 | 9 | NA |
| 9J | Blood toxicological analysis | 0 | 1 | 2 | 3 | 4 | 5 | 6 | 7 | 8 | 9 | NA |
| 9K | Urinary and blood toxicological analysis | 0 | 1 | 2 | 3 | 4 | 5 | 6 | 7 | 8 | 9 | NA |
| 9L | Polysomnographic sleep assessment | 0 | 1 | 2 | 3 | 4 | 5 | 6 | 7 | 8 | 9 | NA |
| 9M | Sexual hormone levels | 0 | 1 | 2 | 3 | 4 | 5 | 6 | 7 | 8 | 9 | NA |
| 9N | Plasma levels of psychotropic medications | 0 | 1 | 2 | 3 | 4 | 5 | 6 | 7 | 8 | 9 | NA |
| 9O | C-reactive protein measurement | 0 | 1 | 2 | 3 | 4 | 5 | 6 | 7 | 8 | 9 | NA |
| 9P | Plasma cortisol determination | 0 | 1 | 2 | 3 | 4 | 5 | 6 | 7 | 8 | 9 | NA |
| 9Q | Plasma levels of Vitamin D | 0 | 1 | 2 | 3 | 4 | 5 | 6 | 7 | 8 | 9 | NA |
| 9R | Others : ………………………………………………… | 0 | 1 | 2 | 3 | 4 | 5 | 6 | 7 | 8 | 9 | NA |
| 9S | None | 0 | 1 | 2 | 3 | 4 | 5 | 6 | 7 | 8 | 9 | NA |

**Question 10**

0 means that this modality is never useful to confirm a good adherence to medication.

1, 2, 3 means that this modality is rarely useful to confirm a good adherence to medication.

4, 5, 6 means that this modality is often useful to confirm a good adherence to medication.

7, 8, 9 means that this modality is alwaysuseful to confirm a good adherence to medication.

NA means that you have no opinion/experience with this modality.

| 10 | You are taking care of an outpatient under 65 years of age suffering from unipolar depression.  What modalities do you recommend to confirm a good adherence to medication?  *Circle the number corresponding to your choice* |
| --- | --- |

| 10A | Systematic evaluation during the individual consultation | 0 | 1 | 2 | 3 | 4 | 5 | 6 | 7 | 8 | 9 | NA |
| --- | --- | --- | --- | --- | --- | --- | --- | --- | --- | --- | --- | --- |
| 10B | Evaluation during a consultation with the patient and one of his relatives | 0 | 1 | 2 | 3 | 4 | 5 | 6 | 7 | 8 | 9 | NA |
| 10C | Proposition of supervision of the observance by the surroundings | 0 | 1 | 2 | 3 | 4 | 5 | 6 | 7 | 8 | 9 | NA |
| 10D | Performing a plasmatic dosage of the treatment | 0 | 1 | 2 | 3 | 4 | 5 | 6 | 7 | 8 | 9 | NA |
| 10E | Participation in a therapeutic education/ psychoeducation program | 0 | 1 | 2 | 3 | 4 | 5 | 6 | 7 | 8 | 9 | NA |
| 10F | Information on potential adverse reactions | 0 | 1 | 2 | 3 | 4 | 5 | 6 | 7 | 8 | 9 | NA |
| 10G | Using a tool for shared medical decision making | 0 | 1 | 2 | 3 | 4 | 5 | 6 | 7 | 8 | 9 | NA |
| 10H | Reducing the number of treatments | 0 | 1 | 2 | 3 | 4 | 5 | 6 | 7 | 8 | 9 | NA |
| 10I | Reducing the number of every day medications | 0 | 1 | 2 | 3 | 4 | 5 | 6 | 7 | 8 | 9 | NA |
| 10J | Using specific tools for treatment adherence | 0 | 1 | 2 | 3 | 4 | 5 | 6 | 7 | 8 | 9 | NA |
| 10K | Treatment of psychiatric comorbidities | 0 | 1 | 2 | 3 | 4 | 5 | 6 | 7 | 8 | 9 | NA |
| 10L | Research and management of adverse effects | 0 | 1 | 2 | 3 | 4 | 5 | 6 | 7 | 8 | 9 | NA |
| 10M | Implementation of a pillbox | 0 | 1 | 2 | 3 | 4 | 5 | 6 | 7 | 8 | 9 | NA |
| 10N | Proposal for partial hospital care | 0 | 1 | 2 | 3 | 4 | 5 | 6 | 7 | 8 | 9 | NA |
| 10O | Proposal for a home-based nurse to distribute the treatments | 0 | 1 | 2 | 3 | 4 | 5 | 6 | 7 | 8 | 9 | NA |

**II. thérapeutic strategies**

# II. Stratégies thérapeutiques

Section n°1 : **Pharmacological strategies**

Section n°2 : **Psychotherapic strategies**

Section n°3 : **Organisation of sequenced treatment**

**II. THERAPEUTIC STRATEGIES**

**Section 1

pharmacological management**

**Section n°1**

## *Stratégies pharmacologiques*

1. **Adjuvant Treatments**
2. **Antidepressant treatment**
3. **Switching strategies**
4. **Combination strategies**
5. **Potentiation strategies**
6. **Strategies to prevent relapse and recurrences**

**Question 11**

0 means that this treatment must never be used in this indication

1, 2, 3 means that this treatment must rarely (3^rd^ intention) be used in this indication

4, 5, 6 means that this treatment may be used (2nd intention) in this indication

7, 8, 9 means that this treatment must be used (1^st^ intention) in this indication

NA means that you have no opinion and/or experience for this treatment

### A. adjuvant TREATMENTS

| 11 | As part of the outpatient treatment of a major depressive episode in an adult under 65 years, what pharmacological treatment do you recommend from the outset in combination with the antidepressant to reduce anxious symptoms?  *Circle the number corresponding to your choice* |
| --- | --- |

| 11A | Another antidepressant from the same pharmacological class | 0 | 1 | 2 | 3 | 4 | 5 | 6 | 7 | 8 | 9 | NA |
| --- | --- | --- | --- | --- | --- | --- | --- | --- | --- | --- | --- | --- |
| 11B | Another antidepressant from a different pharmacological class | 0 | 1 | 2 | 3 | 4 | 5 | 6 | 7 | 8 | 9 | NA |
| 11C | Hydroxyzine | 0 | 1 | 2 | 3 | 4 | 5 | 6 | 7 | 8 | 9 | NA |
| 11D | Benzodiazepine with short half-life | 0 | 1 | 2 | 3 | 4 | 5 | 6 | 7 | 8 | 9 | NA |
| 11E | Benzodiazepine with intermediary half-life | 0 | 1 | 2 | 3 | 4 | 5 | 6 | 7 | 8 | 9 | NA |
| 11F | Benzodiazepine with long half-life | 0 | 1 | 2 | 3 | 4 | 5 | 6 | 7 | 8 | 9 | NA |
| 11G | Hypnotic (zolpidem or zopiclone) | 0 | 1 | 2 | 3 | 4 | 5 | 6 | 7 | 8 | 9 | NA |
| 11H | Etifoxine | 0 | 1 | 2 | 3 | 4 | 5 | 6 | 7 | 8 | 9 | NA |
| 11I | Buspirone | 0 | 1 | 2 | 3 | 4 | 5 | 6 | 7 | 8 | 9 | NA |
| 11J | First generation antipsychotic  (e.g Haloperidol) | 0 | 1 | 2 | 3 | 4 | 5 | 6 | 7 | 8 | 9 | NA |
| 11K | Second generation antipsychotic (e.g Olanzapine) | 0 | 1 | 2 | 3 | 4 | 5 | 6 | 7 | 8 | 9 | NA |
| 11L | Valproic acide derivatives (e.g Divalproex sodium) | 0 | 1 | 2 | 3 | 4 | 5 | 6 | 7 | 8 | 9 | NA |
| 11M | Carbamazepine | 0 | 1 | 2 | 3 | 4 | 5 | 6 | 7 | 8 | 9 | NA |
| 11N | Oxcarbazepine | 0 | 1 | 2 | 3 | 4 | 5 | 6 | 7 | 8 | 9 | NA |
| 11O | Pregabalin | 0 | 1 | 2 | 3 | 4 | 5 | 6 | 7 | 8 | 9 | NA |
| 11P | Topiramate | 0 | 1 | 2 | 3 | 4 | 5 | 6 | 7 | 8 | 9 | NA |
| 11Q | Lithium | 0 | 1 | 2 | 3 | 4 | 5 | 6 | 7 | 8 | 9 | NA |
| 11R | Phytotherapy | 0 | 1 | 2 | 3 | 4 | 5 | 6 | 7 | 8 | 9 | NA |
| 11S | Vitaminotherapy | 0 | 1 | 2 | 3 | 4 | 5 | 6 | 7 | 8 | 9 | NA |
| 11T | Other …………………………………………………………………… | 0 | 1 | 2 | 3 | 4 | 5 | 6 | 7 | 8 | 9 | NA |
| 11U | I don't recommend adjuvant treatment in this indication | 0 | 1 | 2 | 3 | 4 | 5 | 6 | 7 | 8 | 9 | NA |

**Question 12**

0 means that this treatment must never be used in this indication

1, 2, 3 means that this treatment must rarely (3^rd^ intention) be used in this indication

4, 5, 6 means that this treatment may be used (2nd intention) in this indication

7, 8, 9 means that this treatment must be used (1^st^ intention) in this indication

NA means you do not have an opinion and/or experience with treatment

| 12 | As part of the outpatient treatment of a major depressive episode in an adult under 65 years, what pharmacological treatment do you recommend from the outset in combination with the antidepressant to reduce sleep disorders?  *Circle the number corresponding to your choice* |
| --- | --- |

| 12A | Another antidepressant from the same pharmacological class | 0 | 1 | 2 | 3 | 4 | 5 | 6 | 7 | 8 | 9 | NA |
| --- | --- | --- | --- | --- | --- | --- | --- | --- | --- | --- | --- | --- |
| 12B | Another antidepressant from a different pharmacological class | 0 | 1 | 2 | 3 | 4 | 5 | 6 | 7 | 8 | 9 | NA |
| 12C | Hydroxyzine | 0 | 1 | 2 | 3 | 4 | 5 | 6 | 7 | 8 | 9 | NA |
| 12D | Benzodiazepine with short half-life | 0 | 1 | 2 | 3 | 4 | 5 | 6 | 7 | 8 | 9 | NA |
| 12E | Benzodiazepine with intermediary half-life | 0 | 1 | 2 | 3 | 4 | 5 | 6 | 7 | 8 | 9 | NA |
| 12F | Benzodiazepine with long half-life | 0 | 1 | 2 | 3 | 4 | 5 | 6 | 7 | 8 | 9 | NA |
| 12G | Hypnotic (zolpidem or zopiclone) | 0 | 1 | 2 | 3 | 4 | 5 | 6 | 7 | 8 | 9 | NA |
| 12H | Etifoxine | 0 | 1 | 2 | 3 | 4 | 5 | 6 | 7 | 8 | 9 | NA |
| 12I | Buspirone | 0 | 1 | 2 | 3 | 4 | 5 | 6 | 7 | 8 | 9 | NA |
| 12J | First generation antipsychotic  (e.g Haloperidol) | 0 | 1 | 2 | 3 | 4 | 5 | 6 | 7 | 8 | 9 | NA |
| 12K | Second generation antipsychotic (e.g Olanzapine) | 0 | 1 | 2 | 3 | 4 | 5 | 6 | 7 | 8 | 9 | NA |
| 12L | Valproic acide derivatives (e.g Divalproex sodium) | 0 | 1 | 2 | 3 | 4 | 5 | 6 | 7 | 8 | 9 | NA |
| 12M | Carbamazepine | 0 | 1 | 2 | 3 | 4 | 5 | 6 | 7 | 8 | 9 | NA |
| 12N | Oxcarbazepine | 0 | 1 | 2 | 3 | 4 | 5 | 6 | 7 | 8 | 9 | NA |
| 12O | Pregabalin | 0 | 1 | 2 | 3 | 4 | 5 | 6 | 7 | 8 | 9 | NA |
| 12P | Topiramate | 0 | 1 | 2 | 3 | 4 | 5 | 6 | 7 | 8 | 9 | NA |
| 12Q | Lithium | 0 | 1 | 2 | 3 | 4 | 5 | 6 | 7 | 8 | 9 | NA |
| 12R | Phytotherapy | 0 | 1 | 2 | 3 | 4 | 5 | 6 | 7 | 8 | 9 | NA |
| 12S | Vitaminotherapy | 0 | 1 | 2 | 3 | 4 | 5 | 6 | 7 | 8 | 9 | NA |
| 12T | Other …………………………………………………………………… | 0 | 1 | 2 | 3 | 4 | 5 | 6 | 7 | 8 | 9 | NA |
| 12U | I do not recommend adjuvant treatment in this indication | 0 | 1 | 2 | 3 | 4 | 5 | 6 | 7 | 8 | 9 | NA |

**Question 13**

0 means that this treatment must never be used in this indication

1, 2, 3 means that this treatment must rarely (3^rd^ intention) be used in this indication

4, 5, 6 means that this treatment may be used (2nd intention) in this indication

7, 8, 9 means that this treatment must be used (1^st^ intention) in this indication

NA means you do not have an opinion and/or experience with this treatment

| 13 | As part of the outpatient treatment of a major depressive episode in an adult under 65 years, what pharmacological treatment do you recommend from the outset in combination with the antidepressant to reduce the risk of self-harm injury?  *Circle the number corresponding to your choice* |
| --- | --- |

| 13A | Another antidepressant from the same pharmacological class | 0 | 1 | 2 | 3 | 4 | 5 | 6 | 7 | 8 | 9 | NA |
| --- | --- | --- | --- | --- | --- | --- | --- | --- | --- | --- | --- | --- |
| 13B | Another antidepressant from a different pharmacological class | 0 | 1 | 2 | 3 | 4 | 5 | 6 | 7 | 8 | 9 | NA |
| 13C | Hydroxyzine | 0 | 1 | 2 | 3 | 4 | 5 | 6 | 7 | 8 | 9 | NA |
| 13D | Benzodiazepine with short half-life | 0 | 1 | 2 | 3 | 4 | 5 | 6 | 7 | 8 | 9 | NA |
| 13E | Benzodiazepine with intermediary half-life | 0 | 1 | 2 | 3 | 4 | 5 | 6 | 7 | 8 | 9 | NA |
| 13F | Benzodiazepine with long half-life | 0 | 1 | 2 | 3 | 4 | 5 | 6 | 7 | 8 | 9 | NA |
| 13G | Hypnotic (zolpidem or zopiclone) | 0 | 1 | 2 | 3 | 4 | 5 | 6 | 7 | 8 | 9 | NA |
| 13H | Etifoxine | 0 | 1 | 2 | 3 | 4 | 5 | 6 | 7 | 8 | 9 | NA |
| 13I | Buspirone | 0 | 1 | 2 | 3 | 4 | 5 | 6 | 7 | 8 | 9 | NA |
| 13J | First generation antipsychotic  (e.g Haloperidol) | 0 | 1 | 2 | 3 | 4 | 5 | 6 | 7 | 8 | 9 | NA |
| 13K | Second generation antipsychotic (e.g Olanzapine) | 0 | 1 | 2 | 3 | 4 | 5 | 6 | 7 | 8 | 9 | NA |
| 13L | Valproic acide derivatives (e.g Divalproex sodium) | 0 | 1 | 2 | 3 | 4 | 5 | 6 | 7 | 8 | 9 | NA |
| 13M | Carbamazepine | 0 | 1 | 2 | 3 | 4 | 5 | 6 | 7 | 8 | 9 | NA |
| 13N | Oxcarbazepine | 0 | 1 | 2 | 3 | 4 | 5 | 6 | 7 | 8 | 9 | NA |
| 13O | Pregabalin | 0 | 1 | 2 | 3 | 4 | 5 | 6 | 7 | 8 | 9 | NA |
| 13P | Topiramate | 0 | 1 | 2 | 3 | 4 | 5 | 6 | 7 | 8 | 9 | NA |
| 13Q | Lithium | 0 | 1 | 2 | 3 | 4 | 5 | 6 | 7 | 8 | 9 | NA |
| 13R | Phytotherapy | 0 | 1 | 2 | 3 | 4 | 5 | 6 | 7 | 8 | 9 | NA |
| 13S | Vitaminotherapy | 0 | 1 | 2 | 3 | 4 | 5 | 6 | 7 | 8 | 9 | NA |
| 13T | Other …………………………………………………………………… | 0 | 1 | 2 | 3 | 4 | 5 | 6 | 7 | 8 | 9 | NA |
| 13U | I do not recommend adjuvant treatment in this indication | 0 | 1 | 2 | 3 | 4 | 5 | 6 | 7 | 8 | 9 | NA |

**Question 14**

0 means that this treatment must never be used in this indication

1, 2, 3 means that this treatment must rarely (3^rd^ intention) be used in this indication

4, 5, 6 means that this treatment may be used (2nd intention) in this indication

7, 8, 9 means that this treatment must be used (1^st^ intention) in this indication

NA means you do not have an opinion and/or experience with this treatment

| 14 | As part of the outpatient treatment of a major depressive episode in an adult under 65 years, do you recommend the immediate prescription of an adjuvant treatment to provide an immediate therapeutic response until the action of the antidepressant?  Yes  No  If so, what treatment do you recommend?  *Circle the number corresponding to your choice* |
| --- | --- |

| 14A | Another antidepressant from the same pharmacological class | 0 | 1 | 2 | 3 | 4 | 5 | 6 | 7 | 8 | 9 | NA |
| --- | --- | --- | --- | --- | --- | --- | --- | --- | --- | --- | --- | --- |
| 14B | Another antidepressant from a different pharmacological class | 0 | 1 | 2 | 3 | 4 | 5 | 6 | 7 | 8 | 9 | NA |
| 14C | Hydroxyzine | 0 | 1 | 2 | 3 | 4 | 5 | 6 | 7 | 8 | 9 | NA |
| 14D | Benzodiazepine with short half-life | 0 | 1 | 2 | 3 | 4 | 5 | 6 | 7 | 8 | 9 | NA |
| 14E | Benzodiazepine with intermediary half-life | 0 | 1 | 2 | 3 | 4 | 5 | 6 | 7 | 8 | 9 | NA |
| 14F | Benzodiazepine with long half-life | 0 | 1 | 2 | 3 | 4 | 5 | 6 | 7 | 8 | 9 | NA |
| 14G | Hypnotic (zolpidem or zopiclone) | 0 | 1 | 2 | 3 | 4 | 5 | 6 | 7 | 8 | 9 | NA |
| 14H | Etifoxine | 0 | 1 | 2 | 3 | 4 | 5 | 6 | 7 | 8 | 9 | NA |
| 14I | Buspirone | 0 | 1 | 2 | 3 | 4 | 5 | 6 | 7 | 8 | 9 | NA |
| 14J | First generation antipsychotic  (e.g Haloperidol) | 0 | 1 | 2 | 3 | 4 | 5 | 6 | 7 | 8 | 9 | NA |
| 14K | Second generation antipsychotic (e.g Olanzapine) | 0 | 1 | 2 | 3 | 4 | 5 | 6 | 7 | 8 | 9 | NA |
| 14L | Valproic acide derivatives (e.g Divalproex sodium) | 0 | 1 | 2 | 3 | 4 | 5 | 6 | 7 | 8 | 9 | NA |
| 14M | Carbamazepine | 0 | 1 | 2 | 3 | 4 | 5 | 6 | 7 | 8 | 9 | NA |
| 14N | Oxcarbazepine | 0 | 1 | 2 | 3 | 4 | 5 | 6 | 7 | 8 | 9 | NA |
| 14O | Pregabalin | 0 | 1 | 2 | 3 | 4 | 5 | 6 | 7 | 8 | 9 | NA |
| 14P | Topiramate | 0 | 1 | 2 | 3 | 4 | 5 | 6 | 7 | 8 | 9 | NA |
| 14Q | Lithium | 0 | 1 | 2 | 3 | 4 | 5 | 6 | 7 | 8 | 9 | NA |
| 14R | Phytotherapy | 0 | 1 | 2 | 3 | 4 | 5 | 6 | 7 | 8 | 9 | NA |
| 14S | Vitaminotherapy | 0 | 1 | 2 | 3 | 4 | 5 | 6 | 7 | 8 | 9 | NA |
| 14T | Other …………………………………………………………………… | 0 | 1 | 2 | 3 | 4 | 5 | 6 | 7 | 8 | 9 | NA |
| 14U | I do not recommend adjuvant treatment in this indication | 0 | 1 | 2 | 3 | 4 | 5 | 6 | 7 | 8 | 9 | NA |

**Question 15**

0 means that this molecule never has an antidepressant action

1, 2, 3 means that this molecule never has an antidepressant action in rare case

4, 5, 6 means that this molecule may have (in the 2nd intention) an antidepressant action

7, 8, 9 means that this molecule always has an antidepressant action

NA means that you have no opinion/experience concerning this molecule

### B. antidepressant treatments

| 15 | Among the following medications, which ones do you think have antidepressant properties, in monotherapy, in the outpatient treatment of a major depressive episode in an adult under 65 years?  *Circle the number corresponding to your choice* |
| --- | --- |

| 15A | Selective serotonin reuptake inhibitors (e.g Citalopram) | 0 | 1 | 2 | 3 | 4 | 5 | 6 | 7 | 8 | 9 | NA |
| --- | --- | --- | --- | --- | --- | --- | --- | --- | --- | --- | --- | --- |
| 15B | Dual serotonin and norepinephrine reuptake inhibitors (e.g Venlafaxine) | 0 | 1 | 2 | 3 | 4 | 5 | 6 | 7 | 8 | 9 | NA |
| 15C | Tricyclic antidepressants (e.g Clomipramine) | 0 | 1 | 2 | 3 | 4 | 5 | 6 | 7 | 8 | 9 | NA |
| 15D | Bupropion | 0 | 1 | 2 | 3 | 4 | 5 | 6 | 7 | 8 | 9 | NA |
| 15E | Selective monoamine oxidase inhibitors (Moclobemide) | 0 | 1 | 2 | 3 | 4 | 5 | 6 | 7 | 8 | 9 | NA |
| 15F | Non-selective monoamine oxidase inhibitors (Iproniazide) | 0 | 1 | 2 | 3 | 4 | 5 | 6 | 7 | 8 | 9 | NA |
| 15G | Benzodiazepine | 0 | 1 | 2 | 3 | 4 | 5 | 6 | 7 | 8 | 9 | NA |
| 15H | Hydroxyzine | 0 | 1 | 2 | 3 | 4 | 5 | 6 | 7 | 8 | 9 | NA |
| 15I | First generation antipsychotic  (e.g Haloperidol) | 0 | 1 | 2 | 3 | 4 | 5 | 6 | 7 | 8 | 9 | NA |
| 15J | Second generation antipsychotic (e.g Olanzapine) | 0 | 1 | 2 | 3 | 4 | 5 | 6 | 7 | 8 | 9 | NA |
| 15K | Etifoxine | 0 | 1 | 2 | 3 | 4 | 5 | 6 | 7 | 8 | 9 | NA |
| 15L | Lithium | 0 | 1 | 2 | 3 | 4 | 5 | 6 | 7 | 8 | 9 | NA |
| 15M | Valproic acide derivatives (e.g Divalproex sodium) | 0 | 1 | 2 | 3 | 4 | 5 | 6 | 7 | 8 | 9 | NA |
| 15N | Carbamazepine | 0 | 1 | 2 | 3 | 4 | 5 | 6 | 7 | 8 | 9 | NA |
| 15O | Oxcarbazepine | 0 | 1 | 2 | 3 | 4 | 5 | 6 | 7 | 8 | 9 | NA |
| 15P | Lamotrigine | 0 | 1 | 2 | 3 | 4 | 5 | 6 | 7 | 8 | 9 | NA |
| 15Q | Pregabalin | 0 | 1 | 2 | 3 | 4 | 5 | 6 | 7 | 8 | 9 | NA |
| 15R | Gabapentine | 0 | 1 | 2 | 3 | 4 | 5 | 6 | 7 | 8 | 9 | NA |
| 15S | Topiramate | 0 | 1 | 2 | 3 | 4 | 5 | 6 | 7 | 8 | 9 | NA |
| 15T | Thyroid hormons | 0 | 1 | 2 | 3 | 4 | 5 | 6 | 7 | 8 | 9 | NA |

| 15 (Continuated) | Among the following medications, which ones do you think have antidepressant properties, in monotherapy, in the outpatient treatment of a major depressive episode in an adult under 65 years?  *Circle the number corresponding to your choice* |
| --- | --- |

| 15U | Pramipexole | 0 | 1 | 2 | 3 | 4 | 5 | 6 | 7 | 8 | 9 | NA |
| --- | --- | --- | --- | --- | --- | --- | --- | --- | --- | --- | --- | --- |
| 15V | L-Dopa | 0 | 1 | 2 | 3 | 4 | 5 | 6 | 7 | 8 | 9 | NA |
| 15W | Methylphenidate | 0 | 1 | 2 | 3 | 4 | 5 | 6 | 7 | 8 | 9 | NA |
| 15X | Amantadine | 0 | 1 | 2 | 3 | 4 | 5 | 6 | 7 | 8 | 9 | NA |
| 15Y | Modafinil | 0 | 1 | 2 | 3 | 4 | 5 | 6 | 7 | 8 | 9 | NA |
| 15Z | Millepertuis | 0 | 1 | 2 | 3 | 4 | 5 | 6 | 7 | 8 | 9 | NA |

**Question 16**

0 means that there is no need to monitor this element during the prescription of antidepressant treatment.

1, 2, 3 means that this element may be monitor during the prescription of antidepressant treatment in rare cases.

4, 5, 6 means that it this often useful to monitor this element during the prescription of antidepressant treatment in rare cases.

7, 8, 9 means that this element must always be monitor during the prescription of antidepressant treatment

NA means that you have no opinion/experience concerning the monitoring of this element during the prescription of antidepressant treatment

| 16 | Which of the following do you think should be systematically monitored in daily clinical practice, when prescribing any antidepressant treatment, in the management of a depressive unipolar episode, in an adult under the age of 65?  *Circle the number corresponding to your choice* |
| --- | --- |

| 16A | Weight | 0 | 1 | 2 | 3 | 4 | 5 | 6 | 7 | 8 | 9 | NA |
| --- | --- | --- | --- | --- | --- | --- | --- | --- | --- | --- | --- | --- |
| 16B | Blood pressure | 0 | 1 | 2 | 3 | 4 | 5 | 6 | 7 | 8 | 9 | NA |
| 16C | Abdominal circumference | 0 | 1 | 2 | 3 | 4 | 5 | 6 | 7 | 8 | 9 | NA |
| 16D | Electrocardiogram | 0 | 1 | 2 | 3 | 4 | 5 | 6 | 7 | 8 | 9 | NA |
| 16E | Electroencephalography | 0 | 1 | 2 | 3 | 4 | 5 | 6 | 7 | 8 | 9 | NA |
| 16F | Blood count | 0 | 1 | 2 | 3 | 4 | 5 | 6 | 7 | 8 | 9 | NA |
| 16G | Blood electrolytes | 0 | 1 | 2 | 3 | 4 | 5 | 6 | 7 | 8 | 9 | NA |
| 16H | Liver function | 0 | 1 | 2 | 3 | 4 | 5 | 6 | 7 | 8 | 9 | NA |
| 16I | Renal function | 0 | 1 | 2 | 3 | 4 | 5 | 6 | 7 | 8 | 9 | NA |
| 16J | Lipid profile (cholesterol, triglyceride) and glucose levels | 0 | 1 | 2 | 3 | 4 | 5 | 6 | 7 | 8 | 9 | NA |
| 16K | C-reactive protein measurement | 0 | 1 | 2 | 3 | 4 | 5 | 6 | 7 | 8 | 9 | NA |
| 16L | Mood-switching | 0 | 1 | 2 | 3 | 4 | 5 | 6 | 7 | 8 | 9 | NA |
| 16M | Suicide risk | 0 | 1 | 2 | 3 | 4 | 5 | 6 | 7 | 8 | 9 | NA |
| 16N | Other : ……………………………………………………………… | 0 | 1 | 2 | 3 | 4 | 5 | 6 | 7 | 8 | 9 | NA |

**Question 17**

0 means that this characteristic does not influences you in any way towards the prescription of this treatment.

1, 2, 3 means that this characteristic may influences you towards the prescription of this treatment in rare cases.

4, 5, 6 means that this characteristic often influences you towards the prescription of this treatment.

7, 8, 9 means that this characteristic always influences you towards the prescription of this treatment.

NA means that you have no opinion/experience concerning this characteristic.

**SSRI**

**SNRI**

**Mianserine / Mirtazapine**

**Tianeptine**

**Agomelatine**

**Bupropion**

**Tricyclic antidepressant**

**Non selectif irreversible MAOI**

**Selectif reversible MAOI**

| 17 | Do the following clinical features lead you to a particular class of antidepressant treatment due to a better efficacy in the treatment of major depressive episodes in adult under 65 years?  *Pour chaque caractéristique clinique et classe de traitement antidépresseur, notez de 0 (ne m’influence pas du tout vers la prescription) à 9 (m’influence fortement vers la prescription).* |
| --- | --- |

| 17A | Marked moral pain |  |  |  |  |  |  |  |  |  |
| --- | --- | --- | --- | --- | --- | --- | --- | --- | --- | --- |
| 17B | Marked anhedonia |  |  |  |  |  |  |  |  |  |
| 17C | Marked abulia |  |  |  |  |  |  |  |  |  |
| 17D | Marked psychomotor retardation |  |  |  |  |  |  |  |  |  |
| 17E | Marked psychomotor agitation |  |  |  |  |  |  |  |  |  |
| 17F | High suicidal risk |  |  |  |  |  |  |  |  |  |
| 17G | Marked asthenia |  |  |  |  |  |  |  |  |  |
| 17H | Marked weight loss |  |  |  |  |  |  |  |  |  |
| 17I | Atypical features (hyperphagia, hypersomnia) |  |  |  |  |  |  |  |  |  |
| 17J | Marked cognitive impairments |  |  |  |  |  |  |  |  |  |
| 17K | Marked sleep disturbances |  |  |  |  |  |  |  |  |  |
| 17L | Marked sexual dysfunctions |  |  |  |  |  |  |  |  |  |
| 17M | Marked anxious features |  |  |  |  |  |  |  |  |  |
| 17N | Marked psychotic features |  |  |  |  |  |  |  |  |  |
| 17O | Other clinical features :  ……………………………………………… |  |  |  |  |  |  |  |  |  |

**Question 18**

For each treatment and for each type of undesirable effect, note from 0 to 9 with:

- 0: for an extremely bad tolerance for this class of adverse effects,
- 9: for an optimal level of tolerance for this class of adverse effects.

| 18 | What is, according to your experience, the tolerance profile of the following antidepressants used in the treatment of a depressive unipolar episode in an adult under 65 years?  *For each antidepressant treatment, note 0 (very poorly tolerated) to 9 (very well tolerated).*  **Cardiac tolerability**  **Orthostatic hypotension**  **Metabolic tolerability**  **Weight Gain**  **Hepatic tolerability**  **Digestive tolerability**  **Neurological tolerability**  **Sexual tolerability**  **Ocular tolerability** |
| --- | --- |

| 18A1 | Citalopram |  |  |  |  |  |  |  |  |  |
| --- | --- | --- | --- | --- | --- | --- | --- | --- | --- | --- |
| 18A2 | Escitalopram |  |  |  |  |  |  |  |  |  |
| 18A3 | Fluoxetine |  |  |  |  |  |  |  |  |  |
| 18A4 | Fluvoxamine |  |  |  |  |  |  |  |  |  |
| 18A5 | Paroxetine |  |  |  |  |  |  |  |  |  |
| 18A6 | Sertraline |  |  |  |  |  |  |  |  |  |
| 18B1 | Duloxetine |  |  |  |  |  |  |  |  |  |
| 18B2 | Milnacipran |  |  |  |  |  |  |  |  |  |
| 18B3 | Venlafaxine |  |  |  |  |  |  |  |  |  |
| 18C1 | Mianserine |  |  |  |  |  |  |  |  |  |
| 18C2 | Mirtazapine |  |  |  |  |  |  |  |  |  |
| 18D1 | Tianeptine |  |  |  |  |  |  |  |  |  |
| 18D2 | Agomelatine |  |  |  |  |  |  |  |  |  |
| 18D3 | Bupropion |  |  |  |  |  |  |  |  |  |

| 18 (Continuated) | What is, according to your experience, the tolerance profile of the following antidepressants used in the treatment of a depressive unipolar episode in an adult under 65 years?  *For each antidepressant treatment, note 0 (very poorly tolerated) to 9 (very well tolerated).*  **Cardiac tolerability**  **Orthostatic hypotension**  **Metabolic tolerability**  **Weight Gain**  **Hepatic tolerability**  **Digestive tolerability**  **Neurological tolerability**  **Sexual tolerability**  **Ocular tolerability** |
| --- | --- |

| 18E1 | Amoxapine |  |  |  |  |  |  |  |  |  |
| --- | --- | --- | --- | --- | --- | --- | --- | --- | --- | --- |
| 18E2 | Clomipramine |  |  |  |  |  |  |  |  |  |
| 18E3 | Dosulepine |  |  |  |  |  |  |  |  |  |
| 18E4 | Doxepine |  |  |  |  |  |  |  |  |  |
| 18E5 | Imipramine |  |  |  |  |  |  |  |  |  |
| 18E6 | Maprotiline |  |  |  |  |  |  |  |  |  |
| 18E7 | Trimipramine |  |  |  |  |  |  |  |  |  |
| 18F1 | Moclobemide |  |  |  |  |  |  |  |  |  |
| 18F2 | Iproniazide |  |  |  |  |  |  |  |  |  |

| 19 | Quelle est, selon vous, la durée minimale de traitement antidépresseur classiquement préconisée dans le cadre de la prise en charge d’un premier épisode dépressif caractérisé unipolaire d’un adulte de moins de 65 ans ?  In your opinion, how long the ongoing antidepressant should be maintained after achieving clinical remission in an adult patient under the age of 65 suffering from a unipolar depressive episode.  *Circle the number corresponding to your choice (only one possible answer)* |
| --- | --- |

| 19A | Less than 3 months |  |
| --- | --- | --- |
| 19B | 3 months |  |
| 19C | Between 3 and 6 months |  |
| 19D | 6 months |  |
| 19E | Between 6 and 9 months |  |
| 19F | 9 months |  |
| 19G | Between 9 and 12 months |  |
| 19H | 12 months |  |
| 19I | Between 12 and 15 months |  |
| 19J | 15 months |  |
| 19K | Between 15 and 18 months |  |
| 19L | 18 months |  |
| 19M | More than 18 months |  |

**Question 20**

0 means that this feature never guides you toward a longer-term treatment.

1, 2, 3 means that this feature guides you toward a longer-term treatment in rare cases.

4, 5, 6 means that this feature often guides you toward a longer-term treatment.

7, 8, 9 means that this feature always guides you toward a longer-term treatment.

NA means that you have no opinion concerning this strategy.

| 20 | In an adult patient under the age of 65 suffering from a depressive episode treated with an antidepressant, which situations lead you to choose a longer-term treatment?  *Circle the number corresponding to your choice* |
| --- | --- |

| 20A | A history of one previous episode of depression | 0 | 1 | 2 | 3 | 4 | 5 | 6 | 7 | 8 | 9 | NA |
| --- | --- | --- | --- | --- | --- | --- | --- | --- | --- | --- | --- | --- |
| 20B | A history of 2 previous episodes of depression | 0 | 1 | 2 | 3 | 4 | 5 | 6 | 7 | 8 | 9 | NA |
| 20C | A history of at least 3 previous episodes of depression | 0 | 1 | 2 | 3 | 4 | 5 | 6 | 7 | 8 | 9 | NA |
| 20D | The presence of psychotic symptoms | 0 | 1 | 2 | 3 | 4 | 5 | 6 | 7 | 8 | 9 | NA |
| 20E | The presence of marked anxious symptoms | 0 | 1 | 2 | 3 | 4 | 5 | 6 | 7 | 8 | 9 | NA |
| 20F | The presence of high suicidal risk | 0 | 1 | 2 | 3 | 4 | 5 | 6 | 7 | 8 | 9 | NA |
| 20G | The presence of marked atypical symptoms (hypersomnia, hyperphagia) | 0 | 1 | 2 | 3 | 4 | 5 | 6 | 7 | 8 | 9 | NA |
| 20H | Resistance to one antidepressant treatment at adequate duration and dose | 0 | 1 | 2 | 3 | 4 | 5 | 6 | 7 | 8 | 9 | NA |
| 20I | Resistance to two antidepressant treatment at adequate duration and dose | 0 | 1 | 2 | 3 | 4 | 5 | 6 | 7 | 8 | 9 | NA |
| 20J | Resistance to at least three antidepressant treatment at adequate duration and dose | 0 | 1 | 2 | 3 | 4 | 5 | 6 | 7 | 8 | 9 | NA |
| 20K | A long period before reaching remission (> 8 weeks) | 0 | 1 | 2 | 3 | 4 | 5 | 6 | 7 | 8 | 9 | NA |
| 20L | Other : ………………………………………………………… | 0 | 1 | 2 | 3 | 4 | 5 | 6 | 7 | 8 | 9 | NA |

**Question 21**

0 means that you never use this strategy.

1, 2, 3 means that this strategy can be used in rare cases.

4, 5, 6 means that this strategy can often be used.

7, 8, 9 means that this strategy can always be used.

NA means that you have no opinion/experience concerning this strategy.

### C. switching strategies

| 21 | An adult patient under the age of 65 suffering from a first depressive unipolar episode is treated with an antidepressant without clinical improvement. You choose to switch from this antidepressant to another one.  What strategy do you adopt preferentially?  *Circle the number corresponding to your choice* |
| --- | --- |

| 21A | **Concurrent switch:** changes in the dose of both medications are implemented simultaneously. The new medication is gradually titrated upward while the current agent is gradually tapered downward. | 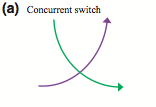 | 0 | 1 | 2 | 3 | 4 | 5 | 6 | 7 | 8 | 9 | NA |
| --- | --- | --- | --- | --- | --- | --- | --- | --- | --- | --- | --- | --- | --- |
| 21B | **Overlapping switch:** dose changes are only implemented for one medication at time, while holding the original medication constant at the original dose until the second medication has reached its optimal dose. | 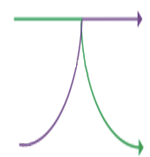 | 0 | 1 | 2 | 3 | 4 | 5 | 6 | 7 | 8 | 9 | NA |
| 21C | **Sequential switch:**  the dose of the current medication is titrated downward until the interruption. Then, the new medication is introduced**.** | 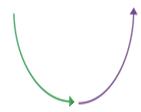 | 0 | 1 | 2 | 3 | 4 | 5 | 6 | 7 | 8 | 9 | NA |

**Question 22**

0 means that this feature never guides you toward this strategy

1, 2, 3 means that this feature guides you toward the switching strategy in rare cases

4, 5, 6 means that this feature often guides you toward the switching strategy

7, 8, 9 means that this feature always guides you toward the switching strategy

NA means that you have no opinion concerning this strategy

| 22 | In an adult patient under the age of 65 suffering from a depressive unipolar episode treated with a first antidepressant agent you choose to switch to a second antidepressant.  Which situations guide your choice to a switching strategy?    *Circle the number corresponding to your choice* |
| --- | --- |

| 22A | Partial response to the first antidepressant | 0 | 1 | 2 | 3 | 4 | 5 | 6 | 7 | 8 | 9 | NA |
| --- | --- | --- | --- | --- | --- | --- | --- | --- | --- | --- | --- | --- |
| 22B | No response to the first treatment | 0 | 1 | 2 | 3 | 4 | 5 | 6 | 7 | 8 | 9 | NA |
| 22C | Poor tolerance to the first treatment | 0 | 1 | 2 | 3 | 4 | 5 | 6 | 7 | 8 | 9 | NA |
| 22D | Fear of triggering antidepressant discontinuation syndrome | 0 | 1 | 2 | 3 | 4 | 5 | 6 | 7 | 8 | 9 | NA |
| 22E | Previous response to the newly introduced treatment | 0 | 1 | 2 | 3 | 4 | 5 | 6 | 7 | 8 | 9 | NA |
| 22F | The typology of patient’s symptoms likely to respond to new antidepressant treatment | 0 | 1 | 2 | 3 | 4 | 5 | 6 | 7 | 8 | 9 | NA |
| 22G | Previous response to the newly introduced treatment in a first-degree parent | 0 | 1 | 2 | 3 | 4 | 5 | 6 | 7 | 8 | 9 | NA |
| 22H | Other : …………………………………………………………… | 0 | 1 | 2 | 3 | 4 | 5 | 6 | 7 | 8 | 9 | NA |

**Question 23**

0 means that this treatment must never be used in this indication

1, 2, 3 means that this treatment must be used in third intention in this indication

4, 5, 6 means that this treatment must be used in second intention in this indication.

7, 8, 9 means that this treatment must be used in first intention in this indication.

NA means that you have no opinion/experience concerning this treatment in this indication.

| 23 | An adult patient under the age of 65 suffering from a first depressive unipolar episode, is treated with a SSRI (e.g citalopram) without clinical improvement.  What treatment do you recommend?  *Circle the number corresponding to your choice* |
| --- | --- |

| 23A | Other SSRI | 0 | 1 | 2 | 3 | 4 | 5 | 6 | 7 | 8 | 9 | NA |
| --- | --- | --- | --- | --- | --- | --- | --- | --- | --- | --- | --- | --- |
| 23B1 | Duloxetine | 0 | 1 | 2 | 3 | 4 | 5 | 6 | 7 | 8 | 9 | NA |
| 23B2 | Milnacipran | 0 | 1 | 2 | 3 | 4 | 5 | 6 | 7 | 8 | 9 | NA |
| 23B3 | Venlafaxine | 0 | 1 | 2 | 3 | 4 | 5 | 6 | 7 | 8 | 9 | NA |
| 23C1 | Mianserine | 0 | 1 | 2 | 3 | 4 | 5 | 6 | 7 | 8 | 9 | NA |
| 23C2 | Mirtazapine | 0 | 1 | 2 | 3 | 4 | 5 | 6 | 7 | 8 | 9 | NA |
| 23D1 | Tianeptine | 0 | 1 | 2 | 3 | 4 | 5 | 6 | 7 | 8 | 9 | NA |
| 23D2 | Agomelatine | 0 | 1 | 2 | 3 | 4 | 5 | 6 | 7 | 8 | 9 | NA |
| 23D3 | Bupropion | 0 | 1 | 2 | 3 | 4 | 5 | 6 | 7 | 8 | 9 | NA |
| 23E1 | Amoxapine | 0 | 1 | 2 | 3 | 4 | 5 | 6 | 7 | 8 | 9 | NA |
| 23E2 | Clomipramine | 0 | 1 | 2 | 3 | 4 | 5 | 6 | 7 | 8 | 9 | NA |
| 23E3 | Dosulepine | 0 | 1 | 2 | 3 | 4 | 5 | 6 | 7 | 8 | 9 | NA |
| 23E4 | Doxepine | 0 | 1 | 2 | 3 | 4 | 5 | 6 | 7 | 8 | 9 | NA |
| 23E5 | Imipramine | 0 | 1 | 2 | 3 | 4 | 5 | 6 | 7 | 8 | 9 | NA |
| 23E6 | Maprotiline | 0 | 1 | 2 | 3 | 4 | 5 | 6 | 7 | 8 | 9 | NA |
| 23E7 | Trimipramine | 0 | 1 | 2 | 3 | 4 | 5 | 6 | 7 | 8 | 9 | NA |
| 23F1 | Moclobemide | 0 | 1 | 2 | 3 | 4 | 5 | 6 | 7 | 8 | 9 | NA |
| 23F2 | Iproniazide | 0 | 1 | 2 | 3 | 4 | 5 | 6 | 7 | 8 | 9 | NA |

**Question 24**

0 means that this treatment must never be used in this indication

1, 2, 3 means that this treatment must be used in third intention in this indication

4, 5, 6 means that this treatment must be used in second intention in this indication.

7, 8, 9 means that this treatment must be used in first intention in this indication.

NA means that you have no opinion/experience concerning this treatment in this indication.

| 24 | An adult patient under the age of 65 suffering from a first depressive unipolar episode, is treated with a SNRI (e.g venlafaxine) without clinical improvement.  What treatment do you recommend?  *Circle the number corresponding to your choice* |
| --- | --- |

| 24A1 | Citalopram | 0 | 1 | 2 | 3 | 4 | 5 | 6 | 7 | 8 | 9 | NA |
| --- | --- | --- | --- | --- | --- | --- | --- | --- | --- | --- | --- | --- |
| 24A2 | Escitalopram | 0 | 1 | 2 | 3 | 4 | 5 | 6 | 7 | 8 | 9 | NA |
| 24A3 | Fluoxetine | 0 | 1 | 2 | 3 | 4 | 5 | 6 | 7 | 8 | 9 | NA |
| 24A4 | Fluvoxamine | 0 | 1 | 2 | 3 | 4 | 5 | 6 | 7 | 8 | 9 | NA |
| 24A5 | Paroxetine | 0 | 1 | 2 | 3 | 4 | 5 | 6 | 7 | 8 | 9 | NA |
| 24A6 | Sertraline | 0 | 1 | 2 | 3 | 4 | 5 | 6 | 7 | 8 | 9 | NA |
| 24B | Other SNRI | 0 | 1 | 2 | 3 | 4 | 5 | 6 | 7 | 8 | 9 | NA |
| 24C1 | Mianserine | 0 | 1 | 2 | 3 | 4 | 5 | 6 | 7 | 8 | 9 | NA |
| 24C2 | Mirtazapine | 0 | 1 | 2 | 3 | 4 | 5 | 6 | 7 | 8 | 9 | NA |
| 24D1 | Tianeptine | 0 | 1 | 2 | 3 | 4 | 5 | 6 | 7 | 8 | 9 | NA |
| 24D2 | Agomelatine | 0 | 1 | 2 | 3 | 4 | 5 | 6 | 7 | 8 | 9 | NA |
| 24D3 | Bupropion | 0 | 1 | 2 | 3 | 4 | 5 | 6 | 7 | 8 | 9 | NA |
| 24E1 | Amoxapine | 0 | 1 | 2 | 3 | 4 | 5 | 6 | 7 | 8 | 9 | NA |
| 24E2 | Clomipramine | 0 | 1 | 2 | 3 | 4 | 5 | 6 | 7 | 8 | 9 | NA |
| 24E3 | Dosulepine | 0 | 1 | 2 | 3 | 4 | 5 | 6 | 7 | 8 | 9 | NA |
| 24E4 | Doxepine | 0 | 1 | 2 | 3 | 4 | 5 | 6 | 7 | 8 | 9 | NA |
| 24E5 | Imipramine | 0 | 1 | 2 | 3 | 4 | 5 | 6 | 7 | 8 | 9 | NA |
| 24E6 | Maprotiline | 0 | 1 | 2 | 3 | 4 | 5 | 6 | 7 | 8 | 9 | NA |
| 24E7 | Trimipramine | 0 | 1 | 2 | 3 | 4 | 5 | 6 | 7 | 8 | 9 | NA |
| 24F1 | Moclobemide | 0 | 1 | 2 | 3 | 4 | 5 | 6 | 7 | 8 | 9 | NA |
| 24F2 | Iproniazide | 0 | 1 | 2 | 3 | 4 | 5 | 6 | 7 | 8 | 9 | NA |

**Question 25**

0 means that this treatment must never be used in this indication

1, 2, 3 means that this treatment must be used in third intention in this indication

4, 5, 6 means that this treatment must be used in second intention in this indication.

7, 8, 9 means that this treatment must be used in first intention in this indication.

NA means that you have no opinion/experience concerning this treatment in this indication.

| 25 | An adult patient under the age of 65 suffering from a first depressive unipolar episode, is treated with a tricyclic antidepressant (e.g clomipramine) without clinical improvement.  What treatment do you recommend?  *Circle the number corresponding to your choice* |
| --- | --- |

| 25A1 | Citalopram | 0 | 1 | 2 | 3 | 4 | 5 | 6 | 7 | 8 | 9 | NA |
| --- | --- | --- | --- | --- | --- | --- | --- | --- | --- | --- | --- | --- |
| 25A2 | Escitalopram | 0 | 1 | 2 | 3 | 4 | 5 | 6 | 7 | 8 | 9 | NA |
| 25A3 | Fluoxetine | 0 | 1 | 2 | 3 | 4 | 5 | 6 | 7 | 8 | 9 | NA |
| 25A4 | Fluvoxamine | 0 | 1 | 2 | 3 | 4 | 5 | 6 | 7 | 8 | 9 | NA |
| 25A5 | Paroxetine | 0 | 1 | 2 | 3 | 4 | 5 | 6 | 7 | 8 | 9 | NA |
| 25A6 | Sertraline | 0 | 1 | 2 | 3 | 4 | 5 | 6 | 7 | 8 | 9 | NA |
| 25B1 | Duloxetine | 0 | 1 | 2 | 3 | 4 | 5 | 6 | 7 | 8 | 9 | NA |
| 25B2 | Milnacipran | 0 | 1 | 2 | 3 | 4 | 5 | 6 | 7 | 8 | 9 | NA |
| 25B3 | Venlafaxine | 0 | 1 | 2 | 3 | 4 | 5 | 6 | 7 | 8 | 9 | NA |
| 25C1 | Mianserine | 0 | 1 | 2 | 3 | 4 | 5 | 6 | 7 | 8 | 9 | NA |
| 25C2 | Mirtazapine | 0 | 1 | 2 | 3 | 4 | 5 | 6 | 7 | 8 | 9 | NA |
| 25D1 | Tianeptine | 0 | 1 | 2 | 3 | 4 | 5 | 6 | 7 | 8 | 9 | NA |
| 25D2 | Agomelatine | 0 | 1 | 2 | 3 | 4 | 5 | 6 | 7 | 8 | 9 | NA |
| 25D3 | Bupropion | 0 | 1 | 2 | 3 | 4 | 5 | 6 | 7 | 8 | 9 | NA |
| 25E | Other tricyclic antidepressant | 0 | 1 | 2 | 3 | 4 | 5 | 6 | 7 | 8 | 9 | NA |
| 25F1 | Moclobemide | 0 | 1 | 2 | 3 | 4 | 5 | 6 | 7 | 8 | 9 | NA |
| 25F2 | Iproniazide | 0 | 1 | 2 | 3 | 4 | 5 | 6 | 7 | 8 | 9 | NA |

**Question 26**

0 means that this treatment must never be used in this indication

1, 2, 3 means that this treatment must be used in third intention in this indication

4, 5, 6 means that this treatment must be used in second intention in this indication.

7, 8, 9 means that this treatment must be used in first intention in this indication.

NA means that you have no opinion/experience concerning this treatment in this indication.

| 26 | An adult patient under the age of 65 suffering from a first depressive unipolar episode, is treated with Mirtazapine or Mianserine without clinical improvement.  What treatment do you recommend?  *Circle the number corresponding to your choice* |
| --- | --- |

| 26A1 | Citalopram | 0 | 1 | 2 | 3 | 4 | 5 | 6 | 7 | 8 | 9 | NA |
| --- | --- | --- | --- | --- | --- | --- | --- | --- | --- | --- | --- | --- |
| 26A2 | Escitalopram | 0 | 1 | 2 | 3 | 4 | 5 | 6 | 7 | 8 | 9 | NA |
| 26A3 | Fluoxetine | 0 | 1 | 2 | 3 | 4 | 5 | 6 | 7 | 8 | 9 | NA |
| 26A4 | Fluvoxamine | 0 | 1 | 2 | 3 | 4 | 5 | 6 | 7 | 8 | 9 | NA |
| 26A5 | Paroxetine | 0 | 1 | 2 | 3 | 4 | 5 | 6 | 7 | 8 | 9 | NA |
| 26A6 | Sertraline | 0 | 1 | 2 | 3 | 4 | 5 | 6 | 7 | 8 | 9 | NA |
| 26B1 | Duloxetine | 0 | 1 | 2 | 3 | 4 | 5 | 6 | 7 | 8 | 9 | NA |
| 26B2 | Milnacipran | 0 | 1 | 2 | 3 | 4 | 5 | 6 | 7 | 8 | 9 | NA |
| 26B3 | Venlafaxine | 0 | 1 | 2 | 3 | 4 | 5 | 6 | 7 | 8 | 9 | NA |
| 26C | Other α2 antagonist | 0 | 1 | 2 | 3 | 4 | 5 | 6 | 7 | 8 | 9 | NA |
| 26D1 | Tianeptine | 0 | 1 | 2 | 3 | 4 | 5 | 6 | 7 | 8 | 9 | NA |
| 26D2 | Agomelatine | 0 | 1 | 2 | 3 | 4 | 5 | 6 | 7 | 8 | 9 | NA |
| 26D3 | Bupropion | 0 | 1 | 2 | 3 | 4 | 5 | 6 | 7 | 8 | 9 | NA |
| 26E1 | Amoxapine | 0 | 1 | 2 | 3 | 4 | 5 | 6 | 7 | 8 | 9 | NA |
| 26E2 | Clomipramine | 0 | 1 | 2 | 3 | 4 | 5 | 6 | 7 | 8 | 9 | NA |
| 26E3 | Dosulepine | 0 | 1 | 2 | 3 | 4 | 5 | 6 | 7 | 8 | 9 | NA |
| 26E4 | Doxepine | 0 | 1 | 2 | 3 | 4 | 5 | 6 | 7 | 8 | 9 | NA |
| 26E5 | Imipramine | 0 | 1 | 2 | 3 | 4 | 5 | 6 | 7 | 8 | 9 | NA |
| 26E6 | Maprotiline | 0 | 1 | 2 | 3 | 4 | 5 | 6 | 7 | 8 | 9 | NA |
| 26E7 | Trimipramine | 0 | 1 | 2 | 3 | 4 | 5 | 6 | 7 | 8 | 9 | NA |
| 26F1 | Moclobemide | 0 | 1 | 2 | 3 | 4 | 5 | 6 | 7 | 8 | 9 | NA |
| 26F2 | Iproniazide | 0 | 1 | 2 | 3 | 4 | 5 | 6 | 7 | 8 | 9 | NA |

**Question 27**

0 means that this situation never leads you to choose a combination of two antidepressants.

1, 2, 3 means that this situation may leads you to choose a combination of two antidepressants in rare cases.

4, 5, 6 means that this situation often leads you to choose a combination of two antidepressants.

7, 8, 9 means that this situation always leads you to choose a combination of two antidepressants.

NA means that you have no opinion/experience with this situation.

### D. combination of antidepressants

| 27 | In an adult patient under the age of 65 suffering from a major depressive episode treated with a first antidepressant agent you choose to add a second antidepressant.  *Circle the number corresponding to your choice* |
| --- | --- |

| 27A | Partial response to the first antidepressant | 0 | 1 | 2 | 3 | 4 | 5 | 6 | 7 | 8 | 9 | NA |
| --- | --- | --- | --- | --- | --- | --- | --- | --- | --- | --- | --- | --- |
| 27B | Good tolerance to the first treatment | 0 | 1 | 2 | 3 | 4 | 5 | 6 | 7 | 8 | 9 | NA |
| 27C | Adverse event with the first treatment which could be offset by the new one | 0 | 1 | 2 | 3 | 4 | 5 | 6 | 7 | 8 | 9 | NA |
| 27D | Fear of triggering antidepressant discontinuation syndrome | 0 | 1 | 2 | 3 | 4 | 5 | 6 | 7 | 8 | 9 | NA |
| 27E | Previous response to the newly introduced treatment | 0 | 1 | 2 | 3 | 4 | 5 | 6 | 7 | 8 | 9 | NA |
| 27F | The typology of patient’s symptoms likely to respond to new antidepressant treatment | 0 | 1 | 2 | 3 | 4 | 5 | 6 | 7 | 8 | 9 | NA |
| 27G | Other : ……………………………………………………………… | 0 | 1 | 2 | 3 | 4 | 5 | 6 | 7 | 8 | 9 | NA |

**Question 28**

0 means that this treatment must never be used in this indication

1, 2, 3 means that this treatment must be used in third intention in this indication

4, 5, 6 means that this treatment must be used in second intention in this indication.

7, 8, 9 means that this treatment must be used in first intention in this indication.

NA means that you have no opinion/experience concerning this treatment in this indication.

| 28 | In an adult patient under the age of 65 suffering from a major depressive episode with a partial response after a treatment with SSRI (e.g sertraline), you choose to add a second antidepressant in combination.  What strategy(s) do you recommend?  *Circle the number corresponding to your choice* |
| --- | --- |

| 28A | Other SSRI (e.g. Citalopram) | 0 | 1 | 2 | 3 | 4 | 5 | 6 | 7 | 8 | 9 | NA |
| --- | --- | --- | --- | --- | --- | --- | --- | --- | --- | --- | --- | --- |
| 28B | Tricyclic antidepressant (e.g. Amitriptyline) | 0 | 1 | 2 | 3 | 4 | 5 | 6 | 7 | 8 | 9 | NA |
| 28C | SNRI (e.g. Venlafaxine) | 0 | 1 | 2 | 3 | 4 | 5 | 6 | 7 | 8 | 9 | NA |
| 28D | Mianserine | 0 | 1 | 2 | 3 | 4 | 5 | 6 | 7 | 8 | 9 | NA |
| 28E | Mirtazapine | 0 | 1 | 2 | 3 | 4 | 5 | 6 | 7 | 8 | 9 | NA |
| 28F | Agomelatine | 0 | 1 | 2 | 3 | 4 | 5 | 6 | 7 | 8 | 9 | NA |
| 28G | Tianeptine | 0 | 1 | 2 | 3 | 4 | 5 | 6 | 7 | 8 | 9 | NA |
| 28H | Bupropion | 0 | 1 | 2 | 3 | 4 | 5 | 6 | 7 | 8 | 9 | NA |
| 28I | Reversible, non-selectif MAOI | 0 | 1 | 2 | 3 | 4 | 5 | 6 | 7 | 8 | 9 | NA |
| 28J | Irreversible, non-selectif MAOI | 0 | 1 | 2 | 3 | 4 | 5 | 6 | 7 | 8 | 9 | NA |
| 28K | Other : ……………………………………………………………… | 0 | 1 | 2 | 3 | 4 | 5 | 6 | 7 | 8 | 9 | NA |

**Question 29**

0 means that this treatment must never be used in this indication.

1, 2, 3 means that this treatment must be used in third intention in this indication.

4, 5, 6 means that this treatment must be used in second intention in this indication.

7, 8, 9 means that this treatment must be used in first intention in this indication.

NA means that you have no opinion/experience concerning this treatment in this indication.

| 29 | In an adult patient under the age of 65 suffering from a major depressive episode with a partial response after a treatment with SNRI (e.g venlafaxine), you choose to add a second antidepressant in combination.  What strategy(s) do you recommend?  *Circle the number corresponding to your choice* |
| --- | --- |

| 29A | Other SNRI | 0 | 1 | 2 | 3 | 4 | 5 | 6 | 7 | 8 | 9 | NA |
| --- | --- | --- | --- | --- | --- | --- | --- | --- | --- | --- | --- | --- |
| 29B | SSRI (e.g. Citalopram) | 0 | 1 | 2 | 3 | 4 | 5 | 6 | 7 | 8 | 9 | NA |
| 29C | Tricyclic antidepressant (e.g. Amitriptyline) | 0 | 1 | 2 | 3 | 4 | 5 | 6 | 7 | 8 | 9 | NA |
| 29D | Mianserine | 0 | 1 | 2 | 3 | 4 | 5 | 6 | 7 | 8 | 9 | NA |
| 29E | Mirtazapine | 0 | 1 | 2 | 3 | 4 | 5 | 6 | 7 | 8 | 9 | NA |
| 29F | Agomelatine | 0 | 1 | 2 | 3 | 4 | 5 | 6 | 7 | 8 | 9 | NA |
| 29G | Tianeptine | 0 | 1 | 2 | 3 | 4 | 5 | 6 | 7 | 8 | 9 | NA |
| 29H | Bupropion | 0 | 1 | 2 | 3 | 4 | 5 | 6 | 7 | 8 | 9 | NA |
| 29I | Reversible, non-selectif MAOI | 0 | 1 | 2 | 3 | 4 | 5 | 6 | 7 | 8 | 9 | NA |
| 29J | Irreversible, non-selectif MAOI | 0 | 1 | 2 | 3 | 4 | 5 | 6 | 7 | 8 | 9 | NA |
| 29K | Other : ……………………………………………………………… | 0 | 1 | 2 | 3 | 4 | 5 | 6 | 7 | 8 | 9 | NA |

**Question 30**

0 means that this treatment must never be used in this indication

1, 2, 3 means that this treatment must be used in third intention in this indication

4, 5, 6 means that this treatment must be used in second intention in this indication.

7, 8, 9 means that this treatment must be used in first intention in this indication.

NA means that you have no opinion/experience concerning this treatment in this indication.

| 30 | In an adult patient under the age of 65 suffering from a major depressive episode with a partial response after a treatment with tricyclic antidepressant (e.g. Amitriptyline), you choose to add a second antidepressant in combination.  What strategy(s) do you recommend?  *Circle the number corresponding to your choice* |
| --- | --- |

| 30A | Other tricyclic antidepressant | 0 | 1 | 2 | 3 | 4 | 5 | 6 | 7 | 8 | 9 | NA |
| --- | --- | --- | --- | --- | --- | --- | --- | --- | --- | --- | --- | --- |
| 30B | SSRI (e.g. Citalopram) | 0 | 1 | 2 | 3 | 4 | 5 | 6 | 7 | 8 | 9 | NA |
| 30C | SNRI (e.g. Venlafaxine) | 0 | 1 | 2 | 3 | 4 | 5 | 6 | 7 | 8 | 9 | NA |
| 30D | Mianserine | 0 | 1 | 2 | 3 | 4 | 5 | 6 | 7 | 8 | 9 | NA |
| 30E | Mirtazapine | 0 | 1 | 2 | 3 | 4 | 5 | 6 | 7 | 8 | 9 | NA |
| 30F | Agomelatine | 0 | 1 | 2 | 3 | 4 | 5 | 6 | 7 | 8 | 9 | NA |
| 30G | Tianeptine | 0 | 1 | 2 | 3 | 4 | 5 | 6 | 7 | 8 | 9 | NA |
| 30H | Bupropion | 0 | 1 | 2 | 3 | 4 | 5 | 6 | 7 | 8 | 9 | NA |
| 30I | Reversible, non-selectif MAOI | 0 | 1 | 2 | 3 | 4 | 5 | 6 | 7 | 8 | 9 | NA |
| 30J | Irreversible, non-selectif MAOI | 0 | 1 | 2 | 3 | 4 | 5 | 6 | 7 | 8 | 9 | NA |
| 30K | Other : ……………………………………………………………… | 0 | 1 | 2 | 3 | 4 | 5 | 6 | 7 | 8 | 9 | NA |

**Question 31**

0 means that this dosage is never indicated.

1, 2, 3 means that this dosage is rarely indicated.

4, 5, 6 means that this dosage is often indicated.

7, 8, 9 means that this dosage is always indicated.

NA means that you have no opinion/experience concerning this dosage.

| 31 | You have introduced a treatment with Mirtazapine to enhance antidepressant efficacy, in an adult patient under the age of 65 suffering from major depressive episode, with no significant organic or psychiatric history.  What is the targeted dose?  *Circle the number corresponding to your choice* |
| --- | --- |

| 31A | 7,5 mg/day | 0 | 1 | 2 | 3 | 4 | 5 | 6 | 7 | 8 | 9 | NA |
| --- | --- | --- | --- | --- | --- | --- | --- | --- | --- | --- | --- | --- |
| 31B | 15 mg/day | 0 | 1 | 2 | 3 | 4 | 5 | 6 | 7 | 8 | 9 | NA |
| 31C | 30 mgday | 0 | 1 | 2 | 3 | 4 | 5 | 6 | 7 | 8 | 9 | NA |
| 31D | 45 mg/day | 0 | 1 | 2 | 3 | 4 | 5 | 6 | 7 | 8 | 9 | NA |
| 31E | 150 mg/day | 0 | 1 | 2 | 3 | 4 | 5 | 6 | 7 | 8 | 9 | NA |
| 31F | Other : ……………………………………………………………… | 0 | 1 | 2 | 3 | 4 | 5 | 6 | 7 | 8 | 9 | NA |

**Question 32**

0 means that this duration is never recommended in this indication

1, 2, 3 means that this duration is rarely recommended in this indication.

4, 5, 6 means that this duration is often recommended in this indication

7, 8, 9 means that this duration is always recommended in this indication

NA means that you have no opinion/experience concerning the duration of this combination in this indication.

| 32 | You have introduced a combination of two antidepressants leading to clinical remission of a depressive unipolar episode, in an adult patient under 65 years.  How long do you maintain this combination?  *Circle the number corresponding to your choice* |
| --- | --- |

| 32A | 4 weeks | 0 | 1 | 2 | 3 | 4 | 5 | 6 | 7 | 8 | 9 | NA |
| --- | --- | --- | --- | --- | --- | --- | --- | --- | --- | --- | --- | --- |
| 32B | 8 weeks | 0 | 1 | 2 | 3 | 4 | 5 | 6 | 7 | 8 | 9 | NA |
| 32C | 3 months | 0 | 1 | 2 | 3 | 4 | 5 | 6 | 7 | 8 | 9 | NA |
| 32D | 6 months | 0 | 1 | 2 | 3 | 4 | 5 | 6 | 7 | 8 | 9 | NA |
| 32E | 1 year | 0 | 1 | 2 | 3 | 4 | 5 | 6 | 7 | 8 | 9 | NA |
| 32F | 2 years | 0 | 1 | 2 | 3 | 4 | 5 | 6 | 7 | 8 | 9 | NA |
| 32G | Other : …………………………………………………………… | 0 | 1 | 2 | 3 | 4 | 5 | 6 | 7 | 8 | 9 | NA |

**Question 33**

0 means that this treatment must never be used in this indication

1, 2, 3 means that this treatment must be used in third intention in this indication

4, 5, 6 means that this treatment must be used in second intention in this indication.

7, 8, 9 means that this treatment must be used in first intention in this indication.

NA means that you have no opinion/experience concerning this treatment in this indication.

### E. ADD-ON strategies

| 33 | Which of the following therapeutic strategies can be used to potentialize an antidepressant treatment in an adult patient under the age of 65, with no significant organic or psychiatric history, suffering from a major depressive episode?  *Circle the number corresponding to your choice* |
| --- | --- |

| 33A | Lithium | 0 | 1 | 2 | 3 | 4 | 5 | 6 | 7 | 8 | 9 | NA |
| --- | --- | --- | --- | --- | --- | --- | --- | --- | --- | --- | --- | --- |
| 33B1 | Thyroid hormons T3 | 0 | 1 | 2 | 3 | 4 | 5 | 6 | 7 | 8 | 9 | NA |
| 33B2 | Thyroid hormons T4 | 0 | 1 | 2 | 3 | 4 | 5 | 6 | 7 | 8 | 9 | NA |
| 33C1 | Amisulpride | 0 | 1 | 2 | 3 | 4 | 5 | 6 | 7 | 8 | 9 | NA |
| 33C2 | Risperidone | 0 | 1 | 2 | 3 | 4 | 5 | 6 | 7 | 8 | 9 | NA |
| 33C3 | Olanzapine | 0 | 1 | 2 | 3 | 4 | 5 | 6 | 7 | 8 | 9 | NA |
| 33C4 | Aripiprazole | 0 | 1 | 2 | 3 | 4 | 5 | 6 | 7 | 8 | 9 | NA |
| 33C5 | Quetiapine | 0 | 1 | 2 | 3 | 4 | 5 | 6 | 7 | 8 | 9 | NA |
| 33C6 | Clozapine | 0 | 1 | 2 | 3 | 4 | 5 | 6 | 7 | 8 | 9 | NA |
| 33C7 | Haloperidol | 0 | 1 | 2 | 3 | 4 | 5 | 6 | 7 | 8 | 9 | NA |
| 33D1 | Buspirone | 0 | 1 | 2 | 3 | 4 | 5 | 6 | 7 | 8 | 9 | NA |
| 33D2 | Benzodiazepine | 0 | 1 | 2 | 3 | 4 | 5 | 6 | 7 | 8 | 9 | NA |
| 33D3 | Hydroxyzine | 0 | 1 | 2 | 3 | 4 | 5 | 6 | 7 | 8 | 9 | NA |
| 33E1 | L-Dopa | 0 | 1 | 2 | 3 | 4 | 5 | 6 | 7 | 8 | 9 | NA |
| 33E2 | Pramipexole | 0 | 1 | 2 | 3 | 4 | 5 | 6 | 7 | 8 | 9 | NA |
| 33F | Modafinil | 0 | 1 | 2 | 3 | 4 | 5 | 6 | 7 | 8 | 9 | NA |
| 33G | Methylphenidate | 0 | 1 | 2 | 3 | 4 | 5 | 6 | 7 | 8 | 9 | NA |

| 33 (Continuated) | Which of the following therapeutic strategies can be used to potentialize an antidepressant treatment in an adult patient under the age of 65, with no significant organic or psychiatric history, suffering from a major depressive episode?  *Circle the number corresponding to your choice* |
| --- | --- |

| 33H1 | Lamotrigine | 0 | 1 | 2 | 3 | 4 | 5 | 6 | 7 | 8 | 9 | NA |
| --- | --- | --- | --- | --- | --- | --- | --- | --- | --- | --- | --- | --- |
| 33H2 | Valproic acide derivatives (e.g Divalproex sodium) | 0 | 1 | 2 | 3 | 4 | 5 | 6 | 7 | 8 | 9 | NA |
| 33H3 | Carbamazepine | 0 | 1 | 2 | 3 | 4 | 5 | 6 | 7 | 8 | 9 | NA |
| 33H4 | Oxcarbazepine | 0 | 1 | 2 | 3 | 4 | 5 | 6 | 7 | 8 | 9 | NA |
| 33H5 | Gabapentine | 0 | 1 | 2 | 3 | 4 | 5 | 6 | 7 | 8 | 9 | NA |
| 33H6 | Topiramate | 0 | 1 | 2 | 3 | 4 | 5 | 6 | 7 | 8 | 9 | NA |
| 33I | Pregabalin | 0 | 1 | 2 | 3 | 4 | 5 | 6 | 7 | 8 | 9 | NA |
| 33J1 | rTMS | 0 | 1 | 2 | 3 | 4 | 5 | 6 | 7 | 8 | 9 | NA |
| 33J2 | ECT | 0 | 1 | 2 | 3 | 4 | 5 | 6 | 7 | 8 | 9 | NA |

**Question 34**

0 means that this antidepressant or class of antidepressants must never be used in this indication

1, 2, 3 means that this antidepressant or class of antidepressants must be used in third intention in this indication

4, 5, 6 means that this antidepressant or class of antidepressants must be used in second intention in this indication.

7, 8, 9 means t that his antidepressant or class of antidepressants must be used in first intention in this indication.

NA means that you have no opinion/experience concerning this antidepressant or class of antidepressants in this indication.

| 34 | No clinical improvement was observed in a patient under the age of 65, with no significant organic or psychiatric history, suffering from a major depressive episode after two antidepressant treatments at adequate dosage and duration.  You decided to introduce a thyroid hormons therapy to potentialize the antidepressant treatment  With which class of antidepressant is this potentialization most effective?  *Circle the number corresponding to your choice* |
| --- | --- |

| 34A | SSRI (e.g Citalopram) | 0 | 1 | 2 | 3 | 4 | 5 | 6 | 7 | 8 | 9 | NA |
| --- | --- | --- | --- | --- | --- | --- | --- | --- | --- | --- | --- | --- |
| 34B | SNRI (e.g Venlafaxine) | 0 | 1 | 2 | 3 | 4 | 5 | 6 | 7 | 8 | 9 | NA |
| 34C | Tricyclic antidepressant (e.g Clomipramine) | 0 | 1 | 2 | 3 | 4 | 5 | 6 | 7 | 8 | 9 | NA |
| 34D | Mianserine / Mirtazapine | 0 | 1 | 2 | 3 | 4 | 5 | 6 | 7 | 8 | 9 | NA |
| 34E | Tianeptine | 0 | 1 | 2 | 3 | 4 | 5 | 6 | 7 | 8 | 9 | NA |
| 34F | Agomelatine | 0 | 1 | 2 | 3 | 4 | 5 | 6 | 7 | 8 | 9 | NA |
| 34G | MAOI A (Moclobemide) | 0 | 1 | 2 | 3 | 4 | 5 | 6 | 7 | 8 | 9 | NA |
| 34H | Non selectif irreversible MAOI (Iproniazide) | 0 | 1 | 2 | 3 | 4 | 5 | 6 | 7 | 8 | 9 | NA |
| 34I | Bupropion | 0 | 1 | 2 | 3 | 4 | 5 | 6 | 7 | 8 | 9 | NA |

**Question 35**

0 means that this dosage is never indicated.

1, 2, 3 means that this dosage is rarely indicated.

4, 5, 6 means that this dosage is often indicated.

7, 8, 9 means that this dosage is always indicated.

NA means that you have no opinion/experience concerning this dosage.

| 35 | You have introduced a treatment with thyroid hormons to enhance antidepressant efficacy, in an adult patient under the age of 65 suffering from major depressive episode, with no significant organic or psychiatric history.  What is the targeted dose?  *Circle the number corresponding to your choice* |
| --- | --- |

| 35A | 12,5 μg/day | 0 | 1 | 2 | 3 | 4 | 5 | 6 | 7 | 8 | 9 | NA |
| --- | --- | --- | --- | --- | --- | --- | --- | --- | --- | --- | --- | --- |
| 35B | 25 μg/day | 0 | 1 | 2 | 3 | 4 | 5 | 6 | 7 | 8 | 9 | NA |
| 35C | 37,5 μg/day | 0 | 1 | 2 | 3 | 4 | 5 | 6 | 7 | 8 | 9 | NA |
| 35D | 50 μg/day | 0 | 1 | 2 | 3 | 4 | 5 | 6 | 7 | 8 | 9 | NA |
| 35E | 75 μg/day | 0 | 1 | 2 | 3 | 4 | 5 | 6 | 7 | 8 | 9 | NA |
| 35F | 100 μg/day | 0 | 1 | 2 | 3 | 4 | 5 | 6 | 7 | 8 | 9 | NA |

**Question 36**

0 means that this paraclinical examination is never indicated.

1, 2, 3 means that this paraclinical examination is rarely indicated.

4, 5, 6 means that this paraclinical examination is often indicated.

7, 8, 9 means that this paraclinical examination is always indicated.

NA means that you have no opinion/experience concerning this paraclinical examination.

| 36 | You introduce a treatment with thyroid hormons to enhance antidepressant efficacy, in an adult patient under the age of 65 suffering from unipolar depressive episode, with no significant organic history.  What pre-therapeutic examination do you realize?  *Circle the number corresponding to your choice* |
| --- | --- |

| 36A | Clinical examination (pulse, blood pressure) | 0 | 1 | 2 | 3 | 4 | 5 | 6 | 7 | 8 | 9 | NA |
| --- | --- | --- | --- | --- | --- | --- | --- | --- | --- | --- | --- | --- |
| 36B | ECG | 0 | 1 | 2 | 3 | 4 | 5 | 6 | 7 | 8 | 9 | NA |
| 36C | TSH-us | 0 | 1 | 2 | 3 | 4 | 5 | 6 | 7 | 8 | 9 | NA |
| 36D | Free T3 and T4 | 0 | 1 | 2 | 3 | 4 | 5 | 6 | 7 | 8 | 9 | NA |
| 36E | Blood count | 0 | 1 | 2 | 3 | 4 | 5 | 6 | 7 | 8 | 9 | NA |
| 36F | Blood electrolytes | 0 | 1 | 2 | 3 | 4 | 5 | 6 | 7 | 8 | 9 | NA |
| 36G | Urea and creatinine | 0 | 1 | 2 | 3 | 4 | 5 | 6 | 7 | 8 | 9 | NA |
| 36H | Liver function | 0 | 1 | 2 | 3 | 4 | 5 | 6 | 7 | 8 | 9 | NA |
| 36I | Other ……………………………………………………………… | 0 | 1 | 2 | 3 | 4 | 5 | 6 | 7 | 8 | 9 | NA |

**Question 37**

0 means that this plasma concentration is never indicated.

1, 2, 3 means that this plasma concentration is rarely targeted in this indication.

4, 5, 6 means that this plasma concentration is often targeted in this indication.

7, 8, 9 means that this plasma concentration is always targeted in this indication.

NA means you have no opinion/experience concerning the plasma concentration in this indication.

| 37 | You introduce a treatment with thyroid hormons to enhance antidepressant efficacy, in an adult patient under the age of 65 suffering from a major depressive episode, with no significant organic history.  .  What is the targeted dose of TSH?  *Circle the number corresponding to your choice* |
| --- | --- |

| 37A | < 0,5 μg/L | 0 | 1 | 2 | 3 | 4 | 5 | 6 | 7 | 8 | 9 | NA |
| --- | --- | --- | --- | --- | --- | --- | --- | --- | --- | --- | --- | --- |
| 37B | Between 0,5 et 1,0 μg/L | 0 | 1 | 2 | 3 | 4 | 5 | 6 | 7 | 8 | 9 | NA |
| 37C | Between 1,0 et 1,5 μg/L | 0 | 1 | 2 | 3 | 4 | 5 | 6 | 7 | 8 | 9 | NA |
| 37D | Between 1,5 et 2,0 μg/L | 0 | 1 | 2 | 3 | 4 | 5 | 6 | 7 | 8 | 9 | NA |
| 37E | > 2,0 μg/L | 0 | 1 | 2 | 3 | 4 | 5 | 6 | 7 | 8 | 9 | NA |
| 37F | None | 0 | 1 | 2 | 3 | 4 | 5 | 6 | 7 | 8 | 9 | NA |

**Question 38**

0 means that this plasma concentration is never indicated.

1, 2, 3 means that this plasma concentration is rarely targeted in this indication.

4, 5, 6 means that this plasma concentration is often targeted in this indication.

7, 8, 9 means that this plasma concentration is always targeted in this indication.

NA means that you have no opinion/experience concerning the plasma concentration in this indication.

| 38 | You introduce a treatment with lithium to enhance antidepressant efficacy, in an adult patient under the age of 65 suffering from a major depressive episode, with no significant organic history.  What plasma concentration of Lithium are you targeting?  *Circle the number corresponding to your choice* |
| --- | --- |

| 38A | Between 0,1 and 0,2 mmol/L | 0 | 1 | 2 | 3 | 4 | 5 | 6 | 7 | 8 | 9 | NA |
| --- | --- | --- | --- | --- | --- | --- | --- | --- | --- | --- | --- | --- |
| 38B | Between 0,2 and 0,3 mmol/L | 0 | 1 | 2 | 3 | 4 | 5 | 6 | 7 | 8 | 9 | NA |
| 38C | Between 0,3 and 0,4 mmol/L | 0 | 1 | 2 | 3 | 4 | 5 | 6 | 7 | 8 | 9 | NA |
| 38D | Between 0,4 and 0,5 mmol/L | 0 | 1 | 2 | 3 | 4 | 5 | 6 | 7 | 8 | 9 | NA |
| 38E | Between 0,5 and 0,6 mmol/L | 0 | 1 | 2 | 3 | 4 | 5 | 6 | 7 | 8 | 9 | NA |
| 38F | Between 0,6 and 0,7 mmol/L | 0 | 1 | 2 | 3 | 4 | 5 | 6 | 7 | 8 | 9 | NA |
| 38G | Between 0,7 and 0,8 mmol/L | 0 | 1 | 2 | 3 | 4 | 5 | 6 | 7 | 8 | 9 | NA |

**Question 39**

0 means that this duration is never expected in this indication.

1, 2, 3 means that this duration is rarely expected in this indication.

4, 5, 6 means that this duration is often expected in this indication.

7, 8, 9 means that this duration is always expected in this indication.

NA means that you have no opinion/experience concerning this duration in this indication.

| 39 | You introduce a treatment with lithium to enhance antidepressant efficacy, in an adult patient under the age of 65 suffering from a major depressive episode, with no significant organic history.  How long do you wait to judge the clinical efficacy of this strategy?  *Circle the number corresponding to your choice* |
| --- | --- |

| 39A | Between 1 day and 1 week | 0 | 1 | 2 | 3 | 4 | 5 | 6 | 7 | 8 | 9 | NA |
| --- | --- | --- | --- | --- | --- | --- | --- | --- | --- | --- | --- | --- |
| 39B | Between 1 and 12 weeks | 0 | 1 | 2 | 3 | 4 | 5 | 6 | 7 | 8 | 9 | NA |
| 39C | Between 2 and 13 weeks | 0 | 1 | 2 | 3 | 4 | 5 | 6 | 7 | 8 | 9 | NA |
| 39D | Between 3 and 14 weeks | 0 | 1 | 2 | 3 | 4 | 5 | 6 | 7 | 8 | 9 | NA |
| 39E | Between 4 and 15 weeks | 0 | 1 | 2 | 3 | 4 | 5 | 6 | 7 | 8 | 9 | NA |
| 39F | Between 5 and 16 weeks | 0 | 1 | 2 | 3 | 4 | 5 | 6 | 7 | 8 | 9 | NA |

**Question 40**

0 means that this dose is never targeted in this indication.

1, 2, 3 means that this dose is rarely targeted in this indication.

4, 5, 6 means that this dose is often targeted in this indication.

7, 8, 9 means that this dose is always targeted in this indication.

NA means that you have no opinion/experience concerning the dose in this indication.

| 40 | You introduce a treatment with risperidone to enhance antidepressant efficacy, in an adult patient under the age of 65 suffering from a major depressive episode, with no significant organic history.  What is the targeted dose?  *Circle the number corresponding to your choice* |
| --- | --- |

| 40A | Lower or equal to 0,5 mg/day | 0 | 1 | 2 | 3 | 4 | 5 | 6 | 7 | 8 | 9 | NA |
| --- | --- | --- | --- | --- | --- | --- | --- | --- | --- | --- | --- | --- |
| 40B | Between 0,5 and 1 mg/day | 0 | 1 | 2 | 3 | 4 | 5 | 6 | 7 | 8 | 9 | NA |
| 40C | Between 1 and 1,5 mg/day | 0 | 1 | 2 | 3 | 4 | 5 | 6 | 7 | 8 | 9 | NA |
| 40D | Between 1,5 and 2 mg/day | 0 | 1 | 2 | 3 | 4 | 5 | 6 | 7 | 8 | 9 | NA |
| 40E | Between 2 and 3 mg/day | 0 | 1 | 2 | 3 | 4 | 5 | 6 | 7 | 8 | 9 | NA |
| 40F | Between 3 and 4 mg/day | 0 | 1 | 2 | 3 | 4 | 5 | 6 | 7 | 8 | 9 | NA |
| 40G | Between 4 and 6 mg/day | 0 | 1 | 2 | 3 | 4 | 5 | 6 | 7 | 8 | 9 | NA |
| 40H | Between 6 and 8 mg/day | 0 | 1 | 2 | 3 | 4 | 5 | 6 | 7 | 8 | 9 | NA |
| 40I | Higher than 8 mg/day | 0 | 1 | 2 | 3 | 4 | 5 | 6 | 7 | 8 | 9 | NA |

**Question 41**

0 means that this dose is never targeted in this indication.

1, 2, 3 means that this dose is rarely targeted in this indication.

4, 5, 6 means that this dose is often targeted in this indication.

7, 8, 9 means that this dose is always targeted in this indication.

NA means that you have no opinion/experience concerning the dose in this indication.

| 41 | You introduce a treatment with olanzapine to enhance antidepressant efficacy, in an adult patient under the age of 65 suffering from a major depressive episode, with no significant organic history.  What is the targeted dose?  *Circle the number corresponding to your choice* |
| --- | --- |

| 41A | 5 mg/day | 0 | 1 | 2 | 3 | 4 | 5 | 6 | 7 | 8 | 9 | NA |
| --- | --- | --- | --- | --- | --- | --- | --- | --- | --- | --- | --- | --- |
| 41B | 7,5 mg/day | 0 | 1 | 2 | 3 | 4 | 5 | 6 | 7 | 8 | 9 | NA |
| 41C | 10 mg/day | 0 | 1 | 2 | 3 | 4 | 5 | 6 | 7 | 8 | 9 | NA |
| 41D | 15 mg/day | 0 | 1 | 2 | 3 | 4 | 5 | 6 | 7 | 8 | 9 | NA |
| 41E | 20 mg/day | 0 | 1 | 2 | 3 | 4 | 5 | 6 | 7 | 8 | 9 | NA |
| 41F | 25 mg/day | 0 | 1 | 2 | 3 | 4 | 5 | 6 | 7 | 8 | 9 | NA |
| 41G | 30 mg/day | 0 | 1 | 2 | 3 | 4 | 5 | 6 | 7 | 8 | 9 | NA |
| 41H | Higher than 30 mg/ day | 0 | 1 | 2 | 3 | 4 | 5 | 6 | 7 | 8 | 9 | NA |

**Question 42**

0 means that this dose is never targeted in this indication.

1, 2, 3 means that this dose is rarely targeted in this indication.

4, 5, 6 means that this dose is often targeted in this indication.

7, 8, 9 means that this dose is always targeted in this indication.

NA means that you have no opinion/experience concerning the dose in this indication.

| 42 | You introduce a treatment with aripiprazole to enhance antidepressant efficacy, in an adult patient under the age of 65 suffering from a major depressive episode, with no significant organic history.  What is the targeted dose?  *Circle the number corresponding to your choice* |
| --- | --- |

| 42A | 5 mg/day | 0 | 1 | 2 | 3 | 4 | 5 | 6 | 7 | 8 | 9 | NA |
| --- | --- | --- | --- | --- | --- | --- | --- | --- | --- | --- | --- | --- |
| 42B | 10 mg/day | 0 | 1 | 2 | 3 | 4 | 5 | 6 | 7 | 8 | 9 | NA |
| 42C | 15 mg/day | 0 | 1 | 2 | 3 | 4 | 5 | 6 | 7 | 8 | 9 | NA |
| 42D | 20 mg/day | 0 | 1 | 2 | 3 | 4 | 5 | 6 | 7 | 8 | 9 | NA |
| 42E | 25 mg/day | 0 | 1 | 2 | 3 | 4 | 5 | 6 | 7 | 8 | 9 | NA |
| 42F | 30 mg/day | 0 | 1 | 2 | 3 | 4 | 5 | 6 | 7 | 8 | 9 | NA |
| 42G | Higher than 30 mg/day | 0 | 1 | 2 | 3 | 4 | 5 | 6 | 7 | 8 | 9 | NA |

**Question 43**

0 means that this dose is never targeted in this indication.

1, 2, 3 means that this dose is rarely targeted in this indication.

4, 5, 6 means that this dose is often targeted in this indication.

7, 8, 9 means that this dose is always targeted in this indication.

NA means that you have no opinion/experience concerning the dose in this indication.

| 43 | You introduce a treatment with quetiapine to enhance antidepressant efficacy, in an adult patient under the age of 65 suffering from a major depressive episode, with no significant organic history.  What is the targeted dose?  *Circle the number corresponding to your choice* |
| --- | --- |

| 43A | 50 mg/day | 0 | 1 | 2 | 3 | 4 | 5 | 6 | 7 | 8 | 9 | NA |
| --- | --- | --- | --- | --- | --- | --- | --- | --- | --- | --- | --- | --- |
| 43B | 100 mg/day | 0 | 1 | 2 | 3 | 4 | 5 | 6 | 7 | 8 | 9 | NA |
| 43C | 150 mg/day | 0 | 1 | 2 | 3 | 4 | 5 | 6 | 7 | 8 | 9 | NA |
| 43D | 200 mg/day | 0 | 1 | 2 | 3 | 4 | 5 | 6 | 7 | 8 | 9 | NA |
| 43E | 250 mg/day | 0 | 1 | 2 | 3 | 4 | 5 | 6 | 7 | 8 | 9 | NA |
| 43F | 300 mg/day | 0 | 1 | 2 | 3 | 4 | 5 | 6 | 7 | 8 | 9 | NA |
| 43G | 350 mg/day | 0 | 1 | 2 | 3 | 4 | 5 | 6 | 7 | 8 | 9 | NA |
| 43H | 400 mg/day | 0 | 1 | 2 | 3 | 4 | 5 | 6 | 7 | 8 | 9 | NA |
| 43I | 450 mg/day | 0 | 1 | 2 | 3 | 4 | 5 | 6 | 7 | 8 | 9 | NA |
| 43J | 500 mg/day | 0 | 1 | 2 | 3 | 4 | 5 | 6 | 7 | 8 | 9 | NA |
| 43K | 550 mg/day | 0 | 1 | 2 | 3 | 4 | 5 | 6 | 7 | 8 | 9 | NA |
| 43L | 600 mg/day | 0 | 1 | 2 | 3 | 4 | 5 | 6 | 7 | 8 | 9 | NA |
| 43M | Higher than 600 mg/day | 0 | 1 | 2 | 3 | 4 | 5 | 6 | 7 | 8 | 9 | NA |

### F. strategies to prevent relapse and recurrence

| 44 | From how many episodes of unipolar depression do you prescribe a treatment to prevent relapse?  *Circle the number corresponding to your choice (only one possible answer)* |
| --- | --- |

| 44A | From the first episode |  |
| --- | --- | --- |
| 44B | After the second episode |  |
| 44C | After the third episode |  |
| 44D | After the fourth episode |  |
| 44E | My decision is independent of the number of episodes |  |
| 44F | I never prescribe any treatment to prevent relapse in unipolar depression |  |
| 44G | I have no opinion |  |

| 45 | From how many episodes of unipolar depression do you prescribe a treatment to prevent recurrence*?*  *Circle the number corresponding to your choice (only one possible answer)* |
| --- | --- |

| 45A | From the first episode |  |
| --- | --- | --- |
| 45B | After the second episode |  |
| 45C | After the third episode |  |
| 45D | After the fourth episode |  |
| 45E | My decision is independent of the number of episodes |  |
| 45F | I never prescribe any treatment to prevent relapse in unipolar depression |  |
| 45G | I have no opinion |  |

**Question 46**

0 means that this feature never guides you toward the prescription of a preventive treatment of relapse

1, 2, 3 means that this feature guides you toward the prescription of a preventive treatment of relapse.

4, 5, 6 means that this feature often guides you toward the prescription of a preventive treatment of relapse.

7, 8, 9 means that this feature always guides you toward the prescription of a preventive treatment of relapse.

NA means that you have no opinion concerning this strategy.

| 46 | Apart from the number of episodes, among the following elements, which ones guides you toward the prescription of a preventive treatment of relapse after a first major depressive episode?  *Circle the number corresponding to your choice* |
| --- | --- |

| 46A | The severity of the episode | 0 | 1 | 2 | 3 | 4 | 5 | 6 | 7 | 8 | 9 | NA |
| --- | --- | --- | --- | --- | --- | --- | --- | --- | --- | --- | --- | --- |
| 46B | The presence of psychotic symptoms | 0 | 1 | 2 | 3 | 4 | 5 | 6 | 7 | 8 | 9 | NA |
| 46C | The presence of anxious symptoms | 0 | 1 | 2 | 3 | 4 | 5 | 6 | 7 | 8 | 9 | NA |
| 46D | The presence of sleep disorders | 0 | 1 | 2 | 3 | 4 | 5 | 6 | 7 | 8 | 9 | NA |
| 46E | The presence of atypical symptoms (hypersomnia, hyperphagia) | 0 | 1 | 2 | 3 | 4 | 5 | 6 | 7 | 8 | 9 | NA |
| 46F | The presence of catatonic symptoms | 0 | 1 | 2 | 3 | 4 | 5 | 6 | 7 | 8 | 9 | NA |
| 46G | The presence of residual depressive symptoms | 0 | 1 | 2 | 3 | 4 | 5 | 6 | 7 | 8 | 9 | NA |
| 46H | A high suicidal risk | 0 | 1 | 2 | 3 | 4 | 5 | 6 | 7 | 8 | 9 | NA |
| 46I | A family history of mood disorders | 0 | 1 | 2 | 3 | 4 | 5 | 6 | 7 | 8 | 9 | NA |
| 46J | A reactional depression | 0 | 1 | 2 | 3 | 4 | 5 | 6 | 7 | 8 | 9 | NA |
| 46K | An endogenous depression | 0 | 1 | 2 | 3 | 4 | 5 | 6 | 7 | 8 | 9 | NA |
| 46L | The occurrence in the peri-menopausal period | 0 | 1 | 2 | 3 | 4 | 5 | 6 | 7 | 8 | 9 | NA |
| 46M | The presence of dysthymic disorder | 0 | 1 | 2 | 3 | 4 | 5 | 6 | 7 | 8 | 9 | NA |
| 46N | The presence of anxiety disorder | 0 | 1 | 2 | 3 | 4 | 5 | 6 | 7 | 8 | 9 | NA |
| 46O | The presence of personality disorder | 0 | 1 | 2 | 3 | 4 | 5 | 6 | 7 | 8 | 9 | NA |
| 46P | A young age (under 30) | 0 | 1 | 2 | 3 | 4 | 5 | 6 | 7 | 8 | 9 | NA |
| 46Q | A significant functional impact | 0 | 1 | 2 | 3 | 4 | 5 | 6 | 7 | 8 | 9 | NA |
| 46R | Other : …………………………………………………………… | 0 | 1 | 2 | 3 | 4 | 5 | 6 | 7 | 8 | 9 | NA |

**Question 47**

0 means that this treatment is never effective in monotherapy or in combination to prevent relapse.

1, 2, 3 means that this treatment is effective in monotherapy or in combination to prevent relapse in rare case.

4, 5, 6 means that this treatment is often effective in monotherapy or in combination to prevent relapse.

7, 8, 9 means that this treatment is always effective in monotherapy or in combination to prevent relapse.

NA means that you have no opinion/experience concerning this in monotherapy or in combination to prevent relapse.

| 47 | Among the following treatment, which ones do you recommend in monotherapy or in combination to prevent relapse?  *Circle the number corresponding to your choice* |
| --- | --- |

| 47A | Lithium | 0 | 1 | 2 | 3 | 4 | 5 | 6 | 7 | 8 | 9 | NA |
| --- | --- | --- | --- | --- | --- | --- | --- | --- | --- | --- | --- | --- |
| 47B1 | Thyroid hormon T3 | 0 | 1 | 2 | 3 | 4 | 5 | 6 | 7 | 8 | 9 | NA |
| 47B2 | Thyroid hormon T4 | 0 | 1 | 2 | 3 | 4 | 5 | 6 | 7 | 8 | 9 | NA |
| 47C1 | Amisulpride | 0 | 1 | 2 | 3 | 4 | 5 | 6 | 7 | 8 | 9 | NA |
| 47C2 | Risperidone | 0 | 1 | 2 | 3 | 4 | 5 | 6 | 7 | 8 | 9 | NA |
| 47C3 | Olanzapine | 0 | 1 | 2 | 3 | 4 | 5 | 6 | 7 | 8 | 9 | NA |
| 47C4 | Aripiprazole | 0 | 1 | 2 | 3 | 4 | 5 | 6 | 7 | 8 | 9 | NA |
| 47C5 | Quetiapine | 0 | 1 | 2 | 3 | 4 | 5 | 6 | 7 | 8 | 9 | NA |
| 47C6 | Clozapine | 0 | 1 | 2 | 3 | 4 | 5 | 6 | 7 | 8 | 9 | NA |
| 47C7 | Haloperidol | 0 | 1 | 2 | 3 | 4 | 5 | 6 | 7 | 8 | 9 | NA |
| 47D1 | Buspirone ( | 0 | 1 | 2 | 3 | 4 | 5 | 6 | 7 | 8 | 9 | NA |
| 47D2 | Benzodiazepine | 0 | 1 | 2 | 3 | 4 | 5 | 6 | 7 | 8 | 9 | NA |
| 47D3 | Hydroxyzine | 0 | 1 | 2 | 3 | 4 | 5 | 6 | 7 | 8 | 9 | NA |
| 47E1 | L-Dopa | 0 | 1 | 2 | 3 | 4 | 5 | 6 | 7 | 8 | 9 | NA |
| 47E2 | Pramipexole | 0 | 1 | 2 | 3 | 4 | 5 | 6 | 7 | 8 | 9 | NA |
| 47F | Modafinil | 0 | 1 | 2 | 3 | 4 | 5 | 6 | 7 | 8 | 9 | NA |
| 47G | Methylphenidate | 0 | 1 | 2 | 3 | 4 | 5 | 6 | 7 | 8 | 9 | NA |
| 47H1 | Lamotrigine | 0 | 1 | 2 | 3 | 4 | 5 | 6 | 7 | 8 | 9 | NA |
| 47H2 | Valproic acide derivatives (e.g Divalproex sodium) | 0 | 1 | 2 | 3 | 4 | 5 | 6 | 7 | 8 | 9 | NA |
| 47H3 | Carbamazepine | 0 | 1 | 2 | 3 | 4 | 5 | 6 | 7 | 8 | 9 | NA |
| 47H4 | Oxcarbazepine | 0 | 1 | 2 | 3 | 4 | 5 | 6 | 7 | 8 | 9 | NA |
| 47H5 | Gabapentine | 0 | 1 | 2 | 3 | 4 | 5 | 6 | 7 | 8 | 9 | NA |
| 47H6 | Topiramate | 0 | 1 | 2 | 3 | 4 | 5 | 6 | 7 | 8 | 9 | NA |
| 47I | Pregabalin | 0 | 1 | 2 | 3 | 4 | 5 | 6 | 7 | 8 | 9 | NA |
| 47J1 | rTMS | 0 | 1 | 2 | 3 | 4 | 5 | 6 | 7 | 8 | 9 | NA |
| 47J2 | ECT | 0 | 1 | 2 | 3 | 4 | 5 | 6 | 7 | 8 | 9 | NA |

**Question 48**

0 means that this element is never interesting to discuss and or/to monitor to prevent relapse.

1, 2, 3 means that this element is rarely interesting to discuss and or/to monitor to prevent relapse.

4, 5, 6 means that this element is often interesting to discuss and or/to monitor to prevent relapse.

7, 8, 9 means that this element is always interesting to discuss and or/to monitor to prevent relapse.

NA means that you have no opinion/experience concerning this element to prevent relapse.

| 48 | As part of the usual follow-up, after obtaining the remission of a major depressive episode in an adult patient under the age of 65, what elements are interesting to discuss and or/to monitor to prevent relapse  *Circle the number corresponding to your choice* |
| --- | --- |

| 48A | Evaluation of adherence to treatment | 0 | 1 | 2 | 3 | 4 | 5 | 6 | 7 | 8 | 9 | NA |
| --- | --- | --- | --- | --- | --- | --- | --- | --- | --- | --- | --- | --- |
| 48B | Neuropsychological assessment of cognitive abilities | 0 | 1 | 2 | 3 | 4 | 5 | 6 | 7 | 8 | 9 | NA |
| 48C | Evaluation of social inclusion | 0 | 1 | 2 | 3 | 4 | 5 | 6 | 7 | 8 | 9 | NA |
| 48D | Evaluation of quality of life | 0 | 1 | 2 | 3 | 4 | 5 | 6 | 7 | 8 | 9 | NA |
| 48E | Search for drug intake | 0 | 1 | 2 | 3 | 4 | 5 | 6 | 7 | 8 | 9 | NA |
| 48F | Evaluation of physical activity | 0 | 1 | 2 | 3 | 4 | 5 | 6 | 7 | 8 | 9 | NA |
| 48G | Proposal for simple dietary advice (meals fixed-time, quantity and types of foods to be preferred) | 0 | 1 | 2 | 3 | 4 | 5 | 6 | 7 | 8 | 9 | NA |
| 48H | Completion of a specialized nutritional assessment | 0 | 1 | 2 | 3 | 4 | 5 | 6 | 7 | 8 | 9 | NA |
| 48I | Proposal for simple advice on sleep hygiene | 0 | 1 | 2 | 3 | 4 | 5 | 6 | 7 | 8 | 9 | NA |
| 48J | Other : ………………………………………………………………… | 0 | 1 | 2 | 3 | 4 | 5 | 6 | 7 | 8 | 9 | NA |

**II. THERAPEUTIC STRATEGIES**

**Section 2

psychotherapeutic strategies**

**Section n°2**

## *Stratégies psychothérapeutiques*

**Question 49**

0 means that this element is never an objective of the psychotherapeutic treatment in this indication.

1, 2, 3 means that this element is rarely an objective of the psychotherapeutic treatment in this indication.

4, 5, 6 means that this element is often an objective of the psychotherapeutic treatment in this indication.

7, 8, 9 means that this element is always an objective of the psychotherapeutic treatment in this indication.

NA NA means that you have no opinion/experience concerning this element as an objective of the psychotherapeutic treatment in this indication.

| 49 | In an adult patient under the age of 65 suffering from a major depressive episode, what are the objective of the psychotherapeutic treatment?  *Circle the number corresponding to your choice* |
| --- | --- |

| 49A | Provide psychological support | 0 | 1 | 2 | 3 | 4 | 5 | 6 | 7 | 8 | 9 | NA |
| --- | --- | --- | --- | --- | --- | --- | --- | --- | --- | --- | --- | --- |
| 49B | Inform about the disease and its management | 0 | 1 | 2 | 3 | 4 | 5 | 6 | 7 | 8 | 9 | NA |
| 49C | Enhance therapeutic alliance | 0 | 1 | 2 | 3 | 4 | 5 | 6 | 7 | 8 | 9 | NA |
| 49D | Enhance therapeutic aadherence | 0 | 1 | 2 | 3 | 4 | 5 | 6 | 7 | 8 | 9 | NA |
| 49E | Allow the patient to develop coping strategies | 0 | 1 | 2 | 3 | 4 | 5 | 6 | 7 | 8 | 9 | NA |
| 49F | Improve psychosocial functioning and quality of life | 0 | 1 | 2 | 3 | 4 | 5 | 6 | 7 | 8 | 9 | NA |
| 49G | Manage psychiatric comorbidities (including addictions, anxiety disorders) | 0 | 1 | 2 | 3 | 4 | 5 | 6 | 7 | 8 | 9 | NA |
| 49H | Teach the patient to assess his mood and to detect signs of clinical worsening | 0 | 1 | 2 | 3 | 4 | 5 | 6 | 7 | 8 | 9 | NA |
| 49I | Help the patient to apprehend his own psychopathology | 0 | 1 | 2 | 3 | 4 | 5 | 6 | 7 | 8 | 9 | NA |
| 49J | Other : ……………………………………………………………… | 0 | 1 | 2 | 3 | 4 | 5 | 6 | 7 | 8 | 9 | NA |

**Question 50**

0 means that this psychotherapeutic proposition is never interesting in this indication.

1, 2, 3 means that this psychotherapeutic proposition is rarely interesting in this indication.

4, 5, 6 means that this psychotherapeutic proposition is often interesting in this indication.

7, 8, 9 means that this psychotherapeutic proposition is always interesting in this indication.

NA means that you have no opinion/experience concerning this psychotherapeutic proposition.

| 50 | Assess the interest of each of the following psychotherapeutic intervention in the management of the acute phase of a major depressive episode of mild to moderate intensity in an adult patient under the age of 65?  *Circle the number corresponding to your choice* |
| --- | --- |

| 50A | Supportive therapy | 0 | 1 | 2 | 3 | 4 | 5 | 6 | 7 | 8 | 9 | NA |
| --- | --- | --- | --- | --- | --- | --- | --- | --- | --- | --- | --- | --- |
| 50B | Cognitive therapy | 0 | 1 | 2 | 3 | 4 | 5 | 6 | 7 | 8 | 9 | NA |
| 50C | Behavioral therapy | 0 | 1 | 2 | 3 | 4 | 5 | 6 | 7 | 8 | 9 | NA |
| 50D | Psychodynamic therapy | 0 | 1 | 2 | 3 | 4 | 5 | 6 | 7 | 8 | 9 | NA |
| 50E | Systemic therapy (family, couple ...) | 0 | 1 | 2 | 3 | 4 | 5 | 6 | 7 | 8 | 9 | NA |
| 50F | Interpersonal therapy and social rhythms | 0 | 1 | 2 | 3 | 4 | 5 | 6 | 7 | 8 | 9 | NA |
| 50G | Psychoeducation | 0 | 1 | 2 | 3 | 4 | 5 | 6 | 7 | 8 | 9 | NA |
| 50H | Body Mediation Therapy | 0 | 1 | 2 | 3 | 4 | 5 | 6 | 7 | 8 | 9 | NA |
| 50I | Mindfulness | 0 | 1 | 2 | 3 | 4 | 5 | 6 | 7 | 8 | 9 | NA |
| 50J | Acceptance and commitment therapy | 0 | 1 | 2 | 3 | 4 | 5 | 6 | 7 | 8 | 9 | NA |
| 50K | Other : ………………………………………………………………… | 0 | 1 | 2 | 3 | 4 | 5 | 6 | 7 | 8 | 9 | NA |

**Question 51**

0 means that this psychotherapeutic proposition is never interesting in this indication.

1, 2, 3 means that this psychotherapeutic proposition is rarely interesting in this indication.

4, 5, 6 means that this psychotherapeutic proposition is often interesting in this indication.

7, 8, 9 means that this psychotherapeutic proposition is always interesting in this indication.

NA means that you have no opinion/experience concerning this psychotherapeutic proposition.

| 51 | Assess the interest of each of the following psychotherapeutic intervention in the management of the acute phase of a major depressive episode of moderate to severe intensity in an adult patient under the age of 65?  *Circle the number corresponding to your choice* |
| --- | --- |

| 51A | Supportive therapy | 0 | 1 | 2 | 3 | 4 | 5 | 6 | 7 | 8 | 9 | NA |
| --- | --- | --- | --- | --- | --- | --- | --- | --- | --- | --- | --- | --- |
| 51B | Cognitive therapy | 0 | 1 | 2 | 3 | 4 | 5 | 6 | 7 | 8 | 9 | NA |
| 51C | Behavioral therapy | 0 | 1 | 2 | 3 | 4 | 5 | 6 | 7 | 8 | 9 | NA |
| 51D | Psychodynamic therapy | 0 | 1 | 2 | 3 | 4 | 5 | 6 | 7 | 8 | 9 | NA |
| 51E | Systemic therapy (family, couple ...) | 0 | 1 | 2 | 3 | 4 | 5 | 6 | 7 | 8 | 9 | NA |
| 51F | Interpersonal therapy and social rhythms | 0 | 1 | 2 | 3 | 4 | 5 | 6 | 7 | 8 | 9 | NA |
| 51G | Psychoeducation | 0 | 1 | 2 | 3 | 4 | 5 | 6 | 7 | 8 | 9 | NA |
| 51H | Body Mediation Therapy | 0 | 1 | 2 | 3 | 4 | 5 | 6 | 7 | 8 | 9 | NA |
| 51I | Mindfulness | 0 | 1 | 2 | 3 | 4 | 5 | 6 | 7 | 8 | 9 | NA |
| 51J | Acceptance and commitment therapy | 0 | 1 | 2 | 3 | 4 | 5 | 6 | 7 | 8 | 9 | NA |
| 51K | Other : ………………………………………………………………… | 0 | 1 | 2 | 3 | 4 | 5 | 6 | 7 | 8 | 9 | NA |

**Question 52**

0 means that this psychotherapeutic proposition is never interesting to maintain in this indication.

1, 2, 3 means that this psychotherapeutic proposition is rarely interesting to maintain in this indication.

4, 5, 6 means that this psychotherapeutic proposition is often interesting to maintain in this indication.

7, 8, 9 means that this psychotherapeutic proposition is always interesting to maintain in this indication.

NA means that you have no opinion/experience concerning the maintaining of this psychotherapeutic proposition.

| 52 | Assess the interest of each of the following psychotherapeutic intervention in the management of a major depressive episode in an adult patient under the age of 65, when clinical remission is reached?  *Circle the number corresponding to your choice* |
| --- | --- |

| 52A | Supportive therapy | 0 | 1 | 2 | 3 | 4 | 5 | 6 | 7 | 8 | 9 | NA |
| --- | --- | --- | --- | --- | --- | --- | --- | --- | --- | --- | --- | --- |
| 52B | Cognitive therapy | 0 | 1 | 2 | 3 | 4 | 5 | 6 | 7 | 8 | 9 | NA |
| 52C | Behavioral therapy | 0 | 1 | 2 | 3 | 4 | 5 | 6 | 7 | 8 | 9 | NA |
| 52D | Psychodynamic therapy | 0 | 1 | 2 | 3 | 4 | 5 | 6 | 7 | 8 | 9 | NA |
| 52E | Systemic therapy (family, couple ...) | 0 | 1 | 2 | 3 | 4 | 5 | 6 | 7 | 8 | 9 | NA |
| 52F | Interpersonal therapy and social rhythms | 0 | 1 | 2 | 3 | 4 | 5 | 6 | 7 | 8 | 9 | NA |
| 52G | Psychoeducation | 0 | 1 | 2 | 3 | 4 | 5 | 6 | 7 | 8 | 9 | NA |
| 52H | Body Mediation Therapy | 0 | 1 | 2 | 3 | 4 | 5 | 6 | 7 | 8 | 9 | NA |
| 52I | Mindfulness | 0 | 1 | 2 | 3 | 4 | 5 | 6 | 7 | 8 | 9 | NA |
| 52J | Acceptance and commitment therapy | 0 | 1 | 2 | 3 | 4 | 5 | 6 | 7 | 8 | 9 | NA |
| 52K | Other : ………………………………………………………………… | 0 | 1 | 2 | 3 | 4 | 5 | 6 | 7 | 8 | 9 | NA |

**II. THERAPEUTIC STRATEGIES**

**Section 3

Organisation
OF SEQUENCED TREATMENT**

**Section n°3**

## *Organisation des séquences thérapeutiques*

1. First line strategy
2. Second line strategy
3. Third line strategy
4. Fourth line strategy
5. Fifth ligne strategy
6. Ultra-resistance (from the sixth-line)

**Question 53**

0 means that this strategy is not recommended in first intention.

1, 2, 3 means that this strategy is recommended in third intention.

4, 5, 6 means that this strategy is recommended in second intention.

7, 8, 9 means that this strategy is recommended in first intention.

NA means that you have no opinion and/or experience with this strategy in this indication.

### A. 1^st^ line Stratégies

| 53 | An adult patient under the age of 65, with no significant organic or psychiatric history, has a depressive unipolar episode of mild to moderate intensity.  What treatment do you recommend?  *Circle the number corresponding to your choice* |
| --- | --- |

| 53A1 | SSRI in monotherapy (e.g Citalopram) | 0 | 1 | 2 | 3 | 4 | 5 | 6 | 7 | 8 | 9 | NA |
| --- | --- | --- | --- | --- | --- | --- | --- | --- | --- | --- | --- | --- |
| 53A2 | SNRI in monotherapy (e.g Venlafaxine) | 0 | 1 | 2 | 3 | 4 | 5 | 6 | 7 | 8 | 9 | NA |
| 53A3 | Tricyclic antidepressant in monotherapy  (e.g Clomipramine) | 0 | 1 | 2 | 3 | 4 | 5 | 6 | 7 | 8 | 9 | NA |
| 53A4 | Mianserine / Mirtazapine in monotherapy | 0 | 1 | 2 | 3 | 4 | 5 | 6 | 7 | 8 | 9 | NA |
| 53A5 | Tianeptine in monotherapy | 0 | 1 | 2 | 3 | 4 | 5 | 6 | 7 | 8 | 9 | NA |
| 53A6 | Agomelatine in monotherapy | 0 | 1 | 2 | 3 | 4 | 5 | 6 | 7 | 8 | 9 | NA |
| 53A7 | MAOI A (Moclobemide) in monotherapy | 0 | 1 | 2 | 3 | 4 | 5 | 6 | 7 | 8 | 9 | NA |
| 53A8 | Non selectif irreversible MAOI (Iproniazide) in monotherapy | 0 | 1 | 2 | 3 | 4 | 5 | 6 | 7 | 8 | 9 | NA |
| 53A9 | Bupropion in monotherapy | 0 | 1 | 2 | 3 | 4 | 5 | 6 | 7 | 8 | 9 | NA |
| 53A10 | Two antidepressants from the same pharmacological class in combination | 0 | 1 | 2 | 3 | 4 | 5 | 6 | 7 | 8 | 9 | NA |
| 53A11 | Two antidepressants from different pharmacological class in combination | 0 | 1 | 2 | 3 | 4 | 5 | 6 | 7 | 8 | 9 | NA |
| 53B | Adding lithium | 0 | 1 | 2 | 3 | 4 | 5 | 6 | 7 | 8 | 9 | NA |
| 53C | Adding thyroid hormones | 0 | 1 | 2 | 3 | 4 | 5 | 6 | 7 | 8 | 9 | NA |
| 53D1 | Adding first-generation antipsychotic (e.g Haloperidol) | 0 | 1 | 2 | 3 | 4 | 5 | 6 | 7 | 8 | 9 | NA |
| 53D2 | Adding second-generation antipsychotic (e.g Risperidone) | 0 | 1 | 2 | 3 | 4 | 5 | 6 | 7 | 8 | 9 | NA |
| 53E1 | Pramipexole in combination | 0 | 1 | 2 | 3 | 4 | 5 | 6 | 7 | 8 | 9 | NA |
| 53E2 | Modafinil in combination | 0 | 1 | 2 | 3 | 4 | 5 | 6 | 7 | 8 | 9 | NA |
| 53F | Methylphenidate in combination | 0 | 1 | 2 | 3 | 4 | 5 | 6 | 7 | 8 | 9 | NA |

| 53 (Continuated) | An adult patient under the age of 65, with no significant organic or psychiatric history, has a depressive unipolar episode of mild to moderate intensity.  What treatment do you recommend?  *Circle the number corresponding to your choice* |
| --- | --- |

| 53G1 | Lamotrigine in combination | 0 | 1 | 2 | 3 | 4 | 5 | 6 | 7 | 8 | 9 | NA |
| --- | --- | --- | --- | --- | --- | --- | --- | --- | --- | --- | --- | --- |
| 53G2 | Valproic acide derivatives (e.g Divalproex sodium) in combination | 0 | 1 | 2 | 3 | 4 | 5 | 6 | 7 | 8 | 9 | NA |
| 53G3 | Carbamazepine in combination | 0 | 1 | 2 | 3 | 4 | 5 | 6 | 7 | 8 | 9 | NA |
| 53H1 | Structured psychotherpy in monotherapy | 0 | 1 | 2 | 3 | 4 | 5 | 6 | 7 | 8 | 9 | NA |
| 53H2 | Structured psychotherpy in combination | 0 | 1 | 2 | 3 | 4 | 5 | 6 | 7 | 8 | 9 | NA |
| 53I1 | ECT in monotherapy | 0 | 1 | 2 | 3 | 4 | 5 | 6 | 7 | 8 | 9 | NA |
| 53I2 | ECT in combination | 0 | 1 | 2 | 3 | 4 | 5 | 6 | 7 | 8 | 9 | NA |
| 53I3 | rTMS in monotherapy | 0 | 1 | 2 | 3 | 4 | 5 | 6 | 7 | 8 | 9 | NA |
| 53I4 | rTMS in combination | 0 | 1 | 2 | 3 | 4 | 5 | 6 | 7 | 8 | 9 | NA |

**Question 54**

0 means that this strategy is not recommended in first intention.

1, 2, 3 means that this strategy is recommended in third intention.

4, 5, 6 means that this strategy is recommended in second intention.

7, 8, 9 means that this strategy is recommended in first intention.

NA means that you have no opinion and/or experience with this strategy in this indication.

| 54 | An adult patient under the age of 65, with no significant organic or psychiatric history, has a depressive unipolar episode of moderate to severe intensity.  What treatment do you recommend?  *Circle the number corresponding to your choice* |
| --- | --- |

| 54A1 | SSRI in monotherapy (e.g Citalopram) | 0 | 1 | 2 | 3 | 4 | 5 | 6 | 7 | 8 | 9 | NA |
| --- | --- | --- | --- | --- | --- | --- | --- | --- | --- | --- | --- | --- |
| 54A2 | SNRI in monotherapy (e.g Venlafaxine) | 0 | 1 | 2 | 3 | 4 | 5 | 6 | 7 | 8 | 9 | NA |
| 54A3 | Tricyclic antidepressant in monotherapy  (e.g Clomipramine) | 0 | 1 | 2 | 3 | 4 | 5 | 6 | 7 | 8 | 9 | NA |
| 54A4 | Mianserine / Mirtazapine in monotherapy | 0 | 1 | 2 | 3 | 4 | 5 | 6 | 7 | 8 | 9 | NA |
| 54A5 | Tianeptine in monotherapy | 0 | 1 | 2 | 3 | 4 | 5 | 6 | 7 | 8 | 9 | NA |
| 54A6 | Agomelatine in monotherapy | 0 | 1 | 2 | 3 | 4 | 5 | 6 | 7 | 8 | 9 | NA |
| 54A7 | MAOI A (Moclobemide) in monotherapy | 0 | 1 | 2 | 3 | 4 | 5 | 6 | 7 | 8 | 9 | NA |
| 54A8 | Non selectif irreversible MAOI (Iproniazide) in monotherapy | 0 | 1 | 2 | 3 | 4 | 5 | 6 | 7 | 8 | 9 | NA |
| 54A9 | Bupropion in monotherapy | 0 | 1 | 2 | 3 | 4 | 5 | 6 | 7 | 8 | 9 | NA |
| 54A10 | Two antidepressants from the same pharmacological class in combination | 0 | 1 | 2 | 3 | 4 | 5 | 6 | 7 | 8 | 9 | NA |
| 54A11 | Two antidepressants from different pharmacological class in combination | 0 | 1 | 2 | 3 | 4 | 5 | 6 | 7 | 8 | 9 | NA |
| 54B | Adding lithium | 0 | 1 | 2 | 3 | 4 | 5 | 6 | 7 | 8 | 9 | NA |
| 54C | Adding thyroid hormones | 0 | 1 | 2 | 3 | 4 | 5 | 6 | 7 | 8 | 9 | NA |
| 54D1 | Adding first-generation antipsychotic (e.g Haloperidol) | 0 | 1 | 2 | 3 | 4 | 5 | 6 | 7 | 8 | 9 | NA |
| 54D2 | Adding second-generation antipsychotic (e.g Risperidone) | 0 | 1 | 2 | 3 | 4 | 5 | 6 | 7 | 8 | 9 | NA |
| 54E1 | Pramipexole in combination | 0 | 1 | 2 | 3 | 4 | 5 | 6 | 7 | 8 | 9 | NA |
| 54E2 | Modafinil in combination | 0 | 1 | 2 | 3 | 4 | 5 | 6 | 7 | 8 | 9 | NA |
| 54F | Methylphenidate in combination | 0 | 1 | 2 | 3 | 4 | 5 | 6 | 7 | 8 | 9 | NA |

| 54 (Continuated) | An adult patient under the age of 65, with no significant organic or psychiatric history, has a depressive unipolar episode of moderate to severe intensity.  What treatment do you recommend?  *Circle the number corresponding to your choice* |
| --- | --- |

| 54G1 | Lamotrigine in combination | 0 | 1 | 2 | 3 | 4 | 5 | 6 | 7 | 8 | 9 | NA |
| --- | --- | --- | --- | --- | --- | --- | --- | --- | --- | --- | --- | --- |
| 54G2 | Valproic acide derivatives (e.g Divalproex sodium) in combination | 0 | 1 | 2 | 3 | 4 | 5 | 6 | 7 | 8 | 9 | NA |
| 54G3 | Carbamazepine in combination | 0 | 1 | 2 | 3 | 4 | 5 | 6 | 7 | 8 | 9 | NA |
| 54H1 | Structured psychotherpy in monotherapy | 0 | 1 | 2 | 3 | 4 | 5 | 6 | 7 | 8 | 9 | NA |
| 54H2 | Structured psychotherpy in combination | 0 | 1 | 2 | 3 | 4 | 5 | 6 | 7 | 8 | 9 | NA |
| 54I1 | ECT in monotherapy | 0 | 1 | 2 | 3 | 4 | 5 | 6 | 7 | 8 | 9 | NA |
| 54I2 | ECT in combination | 0 | 1 | 2 | 3 | 4 | 5 | 6 | 7 | 8 | 9 | NA |
| 54I3 | rTMS in monotherapy | 0 | 1 | 2 | 3 | 4 | 5 | 6 | 7 | 8 | 9 | NA |
| 54I4 | rTMS in combination | 0 | 1 | 2 | 3 | 4 | 5 | 6 | 7 | 8 | 9 | NA |

**Question 55**

0 means that this strategy is not recommended in first intention

1, 2, 3 means that this strategy is recommended in third intention

4, 5, 6 means that this strategy is recommended in second intention

7, 8, 9 means that this strategy is recommended in first intention.

NA means that you have no opinion and/or experience with this strategy in this indication.

| 55 | An adult patient under the age of 65, with no significant organic or psychiatric history, has a depressive unipolar episode with marked anhedonia  What treatment do you recommend?  *Circle the number corresponding to your choice* |
| --- | --- |

| 55A1 | SSRI in monotherapy (e.g Citalopram) | 0 | 1 | 2 | 3 | 4 | 5 | 6 | 7 | 8 | 9 | NA |
| --- | --- | --- | --- | --- | --- | --- | --- | --- | --- | --- | --- | --- |
| 55A2 | SNRI in monotherapy (e.g Venlafaxine) | 0 | 1 | 2 | 3 | 4 | 5 | 6 | 7 | 8 | 9 | NA |
| 55A3 | Tricyclic antidepressant in monotherapy  (e.g Clomipramine) | 0 | 1 | 2 | 3 | 4 | 5 | 6 | 7 | 8 | 9 | NA |
| 55A4 | Mianserine / Mirtazapine in monotherapy | 0 | 1 | 2 | 3 | 4 | 5 | 6 | 7 | 8 | 9 | NA |
| 55A5 | Tianeptine in monotherapy | 0 | 1 | 2 | 3 | 4 | 5 | 6 | 7 | 8 | 9 | NA |
| 55A6 | Agomelatine in monotherapy | 0 | 1 | 2 | 3 | 4 | 5 | 6 | 7 | 8 | 9 | NA |
| 55A7 | MAOI A (Moclobemide) in monotherapy | 0 | 1 | 2 | 3 | 4 | 5 | 6 | 7 | 8 | 9 | NA |
| 55A8 | Non selectif irreversible MAOI (Iproniazide) in monotherapy | 0 | 1 | 2 | 3 | 4 | 5 | 6 | 7 | 8 | 9 | NA |
| 55A9 | Bupropion in monotherapy | 0 | 1 | 2 | 3 | 4 | 5 | 6 | 7 | 8 | 9 | NA |
| 55A10 | Two antidepressants from the same pharmacological class in combination | 0 | 1 | 2 | 3 | 4 | 5 | 6 | 7 | 8 | 9 | NA |
| 55A11 | Two antidepressants from different pharmacological class in combination | 0 | 1 | 2 | 3 | 4 | 5 | 6 | 7 | 8 | 9 | NA |
| 55B | Adding lithium | 0 | 1 | 2 | 3 | 4 | 5 | 6 | 7 | 8 | 9 | NA |
| 55C | Adding thyroid hormones | 0 | 1 | 2 | 3 | 4 | 5 | 6 | 7 | 8 | 9 | NA |
| 55D1 | Adding first-generation antipsychotic (e.g Haloperidol) | 0 | 1 | 2 | 3 | 4 | 5 | 6 | 7 | 8 | 9 | NA |
| 55D2 | Adding second-generation antipsychotic (e.g Risperidone) | 0 | 1 | 2 | 3 | 4 | 5 | 6 | 7 | 8 | 9 | NA |
| 55E1 | Pramipexole in combination | 0 | 1 | 2 | 3 | 4 | 5 | 6 | 7 | 8 | 9 | NA |
| 55E2 | Modafinil in combination | 0 | 1 | 2 | 3 | 4 | 5 | 6 | 7 | 8 | 9 | NA |
| 55F | Methylphenidate in combination | 0 | 1 | 2 | 3 | 4 | 5 | 6 | 7 | 8 | 9 | NA |

| 55 (Continuated) | An adult patient under the age of 65, with no significant organic or psychiatric history, has a depressive unipolar episode with marked anhedonia  What treatment do you recommend?  *Circle the number corresponding to your choice* |
| --- | --- |

| 55G1 | Lamotrigine in combination | 0 | 1 | 2 | 3 | 4 | 5 | 6 | 7 | 8 | 9 | NA |
| --- | --- | --- | --- | --- | --- | --- | --- | --- | --- | --- | --- | --- |
| 55G2 | Valproic acide derivatives (e.g Divalproex sodium) in combination | 0 | 1 | 2 | 3 | 4 | 5 | 6 | 7 | 8 | 9 | NA |
| 55G3 | Carbamazepine in combination | 0 | 1 | 2 | 3 | 4 | 5 | 6 | 7 | 8 | 9 | NA |
| 55H1 | Structured psychotherpy in monotherapy | 0 | 1 | 2 | 3 | 4 | 5 | 6 | 7 | 8 | 9 | NA |
| 55H2 | Structured psychotherpy in combination | 0 | 1 | 2 | 3 | 4 | 5 | 6 | 7 | 8 | 9 | NA |
| 55I1 | ECT in monotherapy | 0 | 1 | 2 | 3 | 4 | 5 | 6 | 7 | 8 | 9 | NA |
| 55I2 | ECT in combination | 0 | 1 | 2 | 3 | 4 | 5 | 6 | 7 | 8 | 9 | NA |
| 55I3 | rTMS in monotherapy | 0 | 1 | 2 | 3 | 4 | 5 | 6 | 7 | 8 | 9 | NA |
| 55I4 | rTMS in combination | 0 | 1 | 2 | 3 | 4 | 5 | 6 | 7 | 8 | 9 | NA |

**Question 56**

0 means that this strategy is not recommended in first intention

1, 2, 3 means that this strategy is recommended in third intention

4, 5, 6 means that this strategy is recommended in second intention

7, 8, 9 means that this strategy is recommended in first intention.

NA means that you have no opinion and/or experience with this strategy in this indication.

| 56 | An adult patient under the age of 65, with no significant organic or psychiatric history, has a depressive unipolar episode with marked psychomotor retardation  What treatment do you recommend?  *Circle the number corresponding to your choice* |
| --- | --- |

| 56A1 | SSRI in monotherapy  (e.g Citalopram) | 0 | 1 | 2 | 3 | 4 | 5 | 6 | 7 | 8 | 9 | NA |
| --- | --- | --- | --- | --- | --- | --- | --- | --- | --- | --- | --- | --- |
| 56A2 | SNRI in monotherapy  (e.g Venlafaxine) | 0 | 1 | 2 | 3 | 4 | 5 | 6 | 7 | 8 | 9 | NA |
| 56A3 | Tricyclic antidepressant in monotherapy  (e.g Clomipramine) | 0 | 1 | 2 | 3 | 4 | 5 | 6 | 7 | 8 | 9 | NA |
| 56A4 | Mianserine / Mirtazapine in monotherapy | 0 | 1 | 2 | 3 | 4 | 5 | 6 | 7 | 8 | 9 | NA |
| 56A5 | Tianeptine in monotherapy | 0 | 1 | 2 | 3 | 4 | 5 | 6 | 7 | 8 | 9 | NA |
| 56A6 | Agomelatine in monotherapy | 0 | 1 | 2 | 3 | 4 | 5 | 6 | 7 | 8 | 9 | NA |
| 56A7 | MAOI A (Moclobemide) in monotherapy | 0 | 1 | 2 | 3 | 4 | 5 | 6 | 7 | 8 | 9 | NA |
| 56A8 | Non selectif irreversible MAOI (Iproniazide) in monotherapy | 0 | 1 | 2 | 3 | 4 | 5 | 6 | 7 | 8 | 9 | NA |
| 56A9 | Bupropion in monotherapy | 0 | 1 | 2 | 3 | 4 | 5 | 6 | 7 | 8 | 9 | NA |
| 56A10 | Two antidepressants from the same pharmacological class in combination | 0 | 1 | 2 | 3 | 4 | 5 | 6 | 7 | 8 | 9 | NA |
| 56A11 | Two antidepressants from different pharmacological class in combination | 0 | 1 | 2 | 3 | 4 | 5 | 6 | 7 | 8 | 9 | NA |
| 56B | Adding lithium | 0 | 1 | 2 | 3 | 4 | 5 | 6 | 7 | 8 | 9 | NA |
| 56C | Adding thyroid hormones | 0 | 1 | 2 | 3 | 4 | 5 | 6 | 7 | 8 | 9 | NA |
| 56D1 | Adding first-generation antipsychotic (e.g Haloperidol) | 0 | 1 | 2 | 3 | 4 | 5 | 6 | 7 | 8 | 9 | NA |
| 56D2 | Adding second-generation antipsychotic (e.g Risperidone) | 0 | 1 | 2 | 3 | 4 | 5 | 6 | 7 | 8 | 9 | NA |
| 56E1 | Pramipexole in combination | 0 | 1 | 2 | 3 | 4 | 5 | 6 | 7 | 8 | 9 | NA |
| 56E2 | Modafinil in combination | 0 | 1 | 2 | 3 | 4 | 5 | 6 | 7 | 8 | 9 | NA |
| 56F | Methylphenidate in combination | 0 | 1 | 2 | 3 | 4 | 5 | 6 | 7 | 8 | 9 | NA |

| 56 (Continuated) | An adult patient under the age of 65, with no significant organic or psychiatric history, has a depressive unipolar episode with marked psychomotor retardation  What treatment do you recommend?  *Circle the number corresponding to your choice* |
| --- | --- |

| 56G1 | Lamotrigine in combination | 0 | 1 | 2 | 3 | 4 | 5 | 6 | 7 | 8 | 9 | NA |
| --- | --- | --- | --- | --- | --- | --- | --- | --- | --- | --- | --- | --- |
| 56G2 | Valproic acide derivatives (e.g Divalproex sodium) in combination | 0 | 1 | 2 | 3 | 4 | 5 | 6 | 7 | 8 | 9 | NA |
| 56G3 | Carbamazepine in combination | 0 | 1 | 2 | 3 | 4 | 5 | 6 | 7 | 8 | 9 | NA |
| 56H1 | Structured psychotherpy in monotherapy | 0 | 1 | 2 | 3 | 4 | 5 | 6 | 7 | 8 | 9 | NA |
| 56H2 | Structured psychotherpy in combination | 0 | 1 | 2 | 3 | 4 | 5 | 6 | 7 | 8 | 9 | NA |
| 56I1 | ECT in monotherapy | 0 | 1 | 2 | 3 | 4 | 5 | 6 | 7 | 8 | 9 | NA |
| 56I2 | ECT in combination | 0 | 1 | 2 | 3 | 4 | 5 | 6 | 7 | 8 | 9 | NA |
| 56I3 | rTMS in monotherapy | 0 | 1 | 2 | 3 | 4 | 5 | 6 | 7 | 8 | 9 | NA |
| 56I4 | rTMS in combination | 0 | 1 | 2 | 3 | 4 | 5 | 6 | 7 | 8 | 9 | NA |

**Question 57**

0 means that this strategy is not recommended in first intention.

1, 2, 3 means that this strategy is recommended in third intention.

4, 5, 6 means that this strategy is recommended in second intention.

7, 8, 9 means that this strategy is recommended in first intention.

NA means that you have no opinion and/or experience with this strategy in this indication.

| 57 | An adult patient under the age of 65, with no significant organic or psychiatric history, has a depressive unipolar episode with marked sleep disturbances  What treatment do you recommend?  *Circle the number corresponding to your choice* |
| --- | --- |

| 57A1 | SSRI in monotherapy  (e.g Citalopram) | 0 | 1 | 2 | 3 | 4 | 5 | 6 | 7 | 8 | 9 | NA |
| --- | --- | --- | --- | --- | --- | --- | --- | --- | --- | --- | --- | --- |
| 57A2 | SNRI in monotherapy  (e.g Venlafaxine) | 0 | 1 | 2 | 3 | 4 | 5 | 6 | 7 | 8 | 9 | NA |
| 57A3 | Tricyclic antidepressant in monotherapy  (e.g Clomipramine) | 0 | 1 | 2 | 3 | 4 | 5 | 6 | 7 | 8 | 9 | NA |
| 57A4 | Mianserine / Mirtazapine in monotherapy | 0 | 1 | 2 | 3 | 4 | 5 | 6 | 7 | 8 | 9 | NA |
| 57A5 | Tianeptine in monotherapy | 0 | 1 | 2 | 3 | 4 | 5 | 6 | 7 | 8 | 9 | NA |
| 57A6 | Agomelatine in monotherapy | 0 | 1 | 2 | 3 | 4 | 5 | 6 | 7 | 8 | 9 | NA |
| 57A7 | MAOI A (Moclobemide) in monotherapy | 0 | 1 | 2 | 3 | 4 | 5 | 6 | 7 | 8 | 9 | NA |
| 57A8 | Non selectif irreversible MAOI (Iproniazide) in monotherapy | 0 | 1 | 2 | 3 | 4 | 5 | 6 | 7 | 8 | 9 | NA |
| 57A9 | Bupropion in monotherapy | 0 | 1 | 2 | 3 | 4 | 5 | 6 | 7 | 8 | 9 | NA |
| 57A10 | Two antidepressants from the same pharmacological class in combination | 0 | 1 | 2 | 3 | 4 | 5 | 6 | 7 | 8 | 9 | NA |
| 57A11 | Two antidepressants from different pharmacological class in combination | 0 | 1 | 2 | 3 | 4 | 5 | 6 | 7 | 8 | 9 | NA |
| 57B | Adding lithium | 0 | 1 | 2 | 3 | 4 | 5 | 6 | 7 | 8 | 9 | NA |
| 57C | Adding thyroid hormones | 0 | 1 | 2 | 3 | 4 | 5 | 6 | 7 | 8 | 9 | NA |
| 57D1 | Adding first-generation antipsychotic (e.g Haloperidol) | 0 | 1 | 2 | 3 | 4 | 5 | 6 | 7 | 8 | 9 | NA |
| 57D2 | Adding second-generation antipsychotic (e.g Risperidone) | 0 | 1 | 2 | 3 | 4 | 5 | 6 | 7 | 8 | 9 | NA |
| 57E1 | Pramipexole in combination | 0 | 1 | 2 | 3 | 4 | 5 | 6 | 7 | 8 | 9 | NA |
| 57E2 | Modafinil in combination | 0 | 1 | 2 | 3 | 4 | 5 | 6 | 7 | 8 | 9 | NA |
| 57F | Methylphenidate in combination | 0 | 1 | 2 | 3 | 4 | 5 | 6 | 7 | 8 | 9 | NA |

| 57 (Continuated) | An adult patient under the age of 65, with no significant organic or psychiatric history, has a depressive unipolar episode with marked sleep disturbances  What treatment do you recommend?  *Circle the number corresponding to your choice* |
| --- | --- |

| 57G1 | Lamotrigine in combination | 0 | 1 | 2 | 3 | 4 | 5 | 6 | 7 | 8 | 9 | NA |
| --- | --- | --- | --- | --- | --- | --- | --- | --- | --- | --- | --- | --- |
| 57G2 | Valproic acide derivatives (e.g Divalproex sodium) in combination | 0 | 1 | 2 | 3 | 4 | 5 | 6 | 7 | 8 | 9 | NA |
| 57G3 | Carbamazepine in combination | 0 | 1 | 2 | 3 | 4 | 5 | 6 | 7 | 8 | 9 | NA |
| 57H1 | Structured psychotherpy in monotherapy | 0 | 1 | 2 | 3 | 4 | 5 | 6 | 7 | 8 | 9 | NA |
| 57H2 | Structured psychotherpy in combination | 0 | 1 | 2 | 3 | 4 | 5 | 6 | 7 | 8 | 9 | NA |
| 57I1 | ECT in monotherapy | 0 | 1 | 2 | 3 | 4 | 5 | 6 | 7 | 8 | 9 | NA |
| 57I2 | ECT in combination | 0 | 1 | 2 | 3 | 4 | 5 | 6 | 7 | 8 | 9 | NA |
| 57I3 | rTMS in monotherapy | 0 | 1 | 2 | 3 | 4 | 5 | 6 | 7 | 8 | 9 | NA |
| 57I4 | rTMS in combination | 0 | 1 | 2 | 3 | 4 | 5 | 6 | 7 | 8 | 9 | NA |

**Question 58**

0 means that this strategy is not recommended in first intention

1, 2, 3 means that this strategy is recommended in third intention

4, 5, 6 means that this strategy is recommended in second intention

7, 8, 9 means that this strategy is recommended in first intention.

NA means that you have no opinion and/or experience with this strategy in this indication.

| 58 | An adult patient under the age of 65, with no significant organic or psychiatric history, has a depressive unipolar episode with marked atypical features (hyperphagia, hypersomnia)  What treatment do you recommend?  *Circle the number corresponding to your choice* |
| --- | --- |

| 58A1 | SSRI in monotherapy  (e.g Citalopram) | 0 | 1 | 2 | 3 | 4 | 5 | 6 | 7 | 8 | 9 | NA |
| --- | --- | --- | --- | --- | --- | --- | --- | --- | --- | --- | --- | --- |
| 58A2 | SNRI in monotherapy  (e.g Venlafaxine) | 0 | 1 | 2 | 3 | 4 | 5 | 6 | 7 | 8 | 9 | NA |
| 58A3 | Tricyclic antidepressant in monotherapy  (e.g Clomipramine) | 0 | 1 | 2 | 3 | 4 | 5 | 6 | 7 | 8 | 9 | NA |
| 58A4 | Mianserine / Mirtazapine in monotherapy | 0 | 1 | 2 | 3 | 4 | 5 | 6 | 7 | 8 | 9 | NA |
| 58A5 | Tianeptine in monotherapy | 0 | 1 | 2 | 3 | 4 | 5 | 6 | 7 | 8 | 9 | NA |
| 58A6 | Agomelatine in monotherapy | 0 | 1 | 2 | 3 | 4 | 5 | 6 | 7 | 8 | 9 | NA |
| 58A7 | MAOI A (Moclobemide) in monotherapy | 0 | 1 | 2 | 3 | 4 | 5 | 6 | 7 | 8 | 9 | NA |
| 58A8 | Non selectif irreversible MAOI (Iproniazide) in monotherapy | 0 | 1 | 2 | 3 | 4 | 5 | 6 | 7 | 8 | 9 | NA |
| 58A9 | Bupropion in monotherapy | 0 | 1 | 2 | 3 | 4 | 5 | 6 | 7 | 8 | 9 | NA |
| 58A10 | Two antidepressants from the same pharmacological class in combination | 0 | 1 | 2 | 3 | 4 | 5 | 6 | 7 | 8 | 9 | NA |
| 58A11 | Two antidepressants from different pharmacological class in combination | 0 | 1 | 2 | 3 | 4 | 5 | 6 | 7 | 8 | 9 | NA |
| 58B | Adding lithium | 0 | 1 | 2 | 3 | 4 | 5 | 6 | 7 | 8 | 9 | NA |
| 58C | Adding thyroid hormones | 0 | 1 | 2 | 3 | 4 | 5 | 6 | 7 | 8 | 9 | NA |
| 58D1 | Adding first-generation antipsychotic (e.g Haloperidol) | 0 | 1 | 2 | 3 | 4 | 5 | 6 | 7 | 8 | 9 | NA |
| 58D2 | Adding second-generation antipsychotic (e.g Risperidone) | 0 | 1 | 2 | 3 | 4 | 5 | 6 | 7 | 8 | 9 | NA |
| 58E1 | Pramipexole in combination | 0 | 1 | 2 | 3 | 4 | 5 | 6 | 7 | 8 | 9 | NA |
| 58E2 | Modafinil in combination | 0 | 1 | 2 | 3 | 4 | 5 | 6 | 7 | 8 | 9 | NA |
| 58F | Methylphenidate in combination | 0 | 1 | 2 | 3 | 4 | 5 | 6 | 7 | 8 | 9 | NA |

| 58 (Continuated) | An adult patient under the age of 65, with no significant organic or psychiatric history, has a depressive unipolar episode with marked atypical features (hyperphagia, hypersomnia)  What treatment do you recommend?  *Circle the number corresponding to your choice* |
| --- | --- |

| 58G1 | Lamotrigine in combination | 0 | 1 | 2 | 3 | 4 | 5 | 6 | 7 | 8 | 9 | NA |
| --- | --- | --- | --- | --- | --- | --- | --- | --- | --- | --- | --- | --- |
| 58G2 | Valproic acide derivatives (e.g Divalproex sodium) in combination | 0 | 1 | 2 | 3 | 4 | 5 | 6 | 7 | 8 | 9 | NA |
| 58G3 | Carbamazepine in combination | 0 | 1 | 2 | 3 | 4 | 5 | 6 | 7 | 8 | 9 | NA |
| 58H1 | Structured psychotherpy in monotherapy | 0 | 1 | 2 | 3 | 4 | 5 | 6 | 7 | 8 | 9 | NA |
| 58H2 | Structured psychotherpy in combination | 0 | 1 | 2 | 3 | 4 | 5 | 6 | 7 | 8 | 9 | NA |
| 58I1 | ECT in monotherapy | 0 | 1 | 2 | 3 | 4 | 5 | 6 | 7 | 8 | 9 | NA |
| 58I2 | ECT in combination | 0 | 1 | 2 | 3 | 4 | 5 | 6 | 7 | 8 | 9 | NA |
| 58I3 | rTMS in monotherapy | 0 | 1 | 2 | 3 | 4 | 5 | 6 | 7 | 8 | 9 | NA |
| 58I4 | rTMS in combination | 0 | 1 | 2 | 3 | 4 | 5 | 6 | 7 | 8 | 9 | NA |

**Question 59**

0 means that this strategy is not recommended in first intention.

1, 2, 3 means that this strategy is recommended in third intention.

4, 5, 6 means that this strategy is recommended in second intention.

7, 8, 9 means that this strategy is recommended in first intention.

NA means that you have no opinion and/or experience with this strategy in this indication.

| 59 | An adult patient under the age of 65, with no significant organic or psychiatric history, has a depressive unipolar episode with marked psychotics features  What treatment do you recommend?  *Circle the number corresponding to your choice* |
| --- | --- |

| 59A1 | SSRI in monotherapy  (e.g Citalopram) | 0 | 1 | 2 | 3 | 4 | 5 | 6 | 7 | 8 | 9 | NA |
| --- | --- | --- | --- | --- | --- | --- | --- | --- | --- | --- | --- | --- |
| 59A2 | SNRI in monotherapy  (e.g Venlafaxine) | 0 | 1 | 2 | 3 | 4 | 5 | 6 | 7 | 8 | 9 | NA |
| 59A3 | Tricyclic antidepressant in monotherapy  (e.g Clomipramine) | 0 | 1 | 2 | 3 | 4 | 5 | 6 | 7 | 8 | 9 | NA |
| 59A4 | Mianserine / Mirtazapine in monotherapy | 0 | 1 | 2 | 3 | 4 | 5 | 6 | 7 | 8 | 9 | NA |
| 59A5 | Tianeptine in monotherapy | 0 | 1 | 2 | 3 | 4 | 5 | 6 | 7 | 8 | 9 | NA |
| 59A6 | Agomelatine in monotherapy | 0 | 1 | 2 | 3 | 4 | 5 | 6 | 7 | 8 | 9 | NA |
| 59A7 | MAOI A (Moclobemide) in monotherapy | 0 | 1 | 2 | 3 | 4 | 5 | 6 | 7 | 8 | 9 | NA |
| 59A8 | Non selectif irreversible MAOI (Iproniazide) in monotherapy | 0 | 1 | 2 | 3 | 4 | 5 | 6 | 7 | 8 | 9 | NA |
| 59A9 | Bupropion in monotherapy | 0 | 1 | 2 | 3 | 4 | 5 | 6 | 7 | 8 | 9 | NA |
| 59A10 | Two antidepressants from the same pharmacological class in combination | 0 | 1 | 2 | 3 | 4 | 5 | 6 | 7 | 8 | 9 | NA |
| 59A11 | Two antidepressants from different pharmacological class in combination | 0 | 1 | 2 | 3 | 4 | 5 | 6 | 7 | 8 | 9 | NA |
| 59B | Adding lithium | 0 | 1 | 2 | 3 | 4 | 5 | 6 | 7 | 8 | 9 | NA |
| 59C | Adding thyroid hormones | 0 | 1 | 2 | 3 | 4 | 5 | 6 | 7 | 8 | 9 | NA |
| 59D1 | Adding first-generation antipsychotic (e.g Haloperidol) | 0 | 1 | 2 | 3 | 4 | 5 | 6 | 7 | 8 | 9 | NA |
| 59D2 | Adding second-generation antipsychotic (e.g Risperidone) | 0 | 1 | 2 | 3 | 4 | 5 | 6 | 7 | 8 | 9 | NA |
| 59E1 | Pramipexole in combination | 0 | 1 | 2 | 3 | 4 | 5 | 6 | 7 | 8 | 9 | NA |
| 59E2 | Modafinil in combination | 0 | 1 | 2 | 3 | 4 | 5 | 6 | 7 | 8 | 9 | NA |
| 59F | Methylphenidate in combination | 0 | 1 | 2 | 3 | 4 | 5 | 6 | 7 | 8 | 9 | NA |

| 59 (Continuated) | An adult patient under the age of 65, with no significant organic or psychiatric history, has a depressive unipolar episode with marked psychotics features  What treatment do you recommend?  *Circle the number corresponding to your choice* |
| --- | --- |

| 59G1 | Lamotrigine in combination | 0 | 1 | 2 | 3 | 4 | 5 | 6 | 7 | 8 | 9 | NA |
| --- | --- | --- | --- | --- | --- | --- | --- | --- | --- | --- | --- | --- |
| 59G2 | Valproic acide derivatives (e.g Divalproex sodium) in combination | 0 | 1 | 2 | 3 | 4 | 5 | 6 | 7 | 8 | 9 | NA |
| 59G3 | Carbamazepine in combination | 0 | 1 | 2 | 3 | 4 | 5 | 6 | 7 | 8 | 9 | NA |
| 59H1 | Structured psychotherpy in monotherapy | 0 | 1 | 2 | 3 | 4 | 5 | 6 | 7 | 8 | 9 | NA |
| 59H2 | Structured psychotherpy in combination | 0 | 1 | 2 | 3 | 4 | 5 | 6 | 7 | 8 | 9 | NA |
| 59I1 | ECT in monotherapy | 0 | 1 | 2 | 3 | 4 | 5 | 6 | 7 | 8 | 9 | NA |
| 59I2 | ECT in combination | 0 | 1 | 2 | 3 | 4 | 5 | 6 | 7 | 8 | 9 | NA |
| 59I3 | rTMS in monotherapy | 0 | 1 | 2 | 3 | 4 | 5 | 6 | 7 | 8 | 9 | NA |
| 59I4 | rTMS in combination | 0 | 1 | 2 | 3 | 4 | 5 | 6 | 7 | 8 | 9 | NA |

**Question 60**

0 means that this strategy is not recommended in first intention.

1, 2, 3 means that this strategy is recommended in third intention.

4, 5, 6 means that this strategy is recommended in second intention.

7, 8, 9 means that this strategy is recommended in first intention.

NA means that you have no opinion and/or experience with this strategy in this indication.

| 60 | An adult patient under the age of 65, with no significant organic or psychiatric history, has a depressive unipolar episode with high suicide risk  What treatment do you recommend?  *Circle the number corresponding to your choice* |
| --- | --- |

| 60A1 | SSRI in monotherapy  (e.g Citalopram) | 0 | 1 | 2 | 3 | 4 | 5 | 6 | 7 | 8 | 9 | NA |
| --- | --- | --- | --- | --- | --- | --- | --- | --- | --- | --- | --- | --- |
| 60A2 | SNRI in monotherapy  (e.g Venlafaxine) | 0 | 1 | 2 | 3 | 4 | 5 | 6 | 7 | 8 | 9 | NA |
| 60A3 | Tricyclic antidepressant in monotherapy  (e.g Clomipramine) | 0 | 1 | 2 | 3 | 4 | 5 | 6 | 7 | 8 | 9 | NA |
| 60A4 | Mianserine / Mirtazapine in monotherapy | 0 | 1 | 2 | 3 | 4 | 5 | 6 | 7 | 8 | 9 | NA |
| 60A5 | Tianeptine in monotherapy | 0 | 1 | 2 | 3 | 4 | 5 | 6 | 7 | 8 | 9 | NA |
| 60A6 | Agomelatine in monotherapy | 0 | 1 | 2 | 3 | 4 | 5 | 6 | 7 | 8 | 9 | NA |
| 60A7 | MAOI A (Moclobemide) in monotherapy | 0 | 1 | 2 | 3 | 4 | 5 | 6 | 7 | 8 | 9 | NA |
| 60A8 | Non selectif irreversible MAOI (Iproniazide) in monotherapy | 0 | 1 | 2 | 3 | 4 | 5 | 6 | 7 | 8 | 9 | NA |
| 60A9 | Bupropion in monotherapy | 0 | 1 | 2 | 3 | 4 | 5 | 6 | 7 | 8 | 9 | NA |
| 60A10 | Two antidepressants from the same pharmacological class in combination | 0 | 1 | 2 | 3 | 4 | 5 | 6 | 7 | 8 | 9 | NA |
| 60A11 | Two antidepressants from different pharmacological class in combination | 0 | 1 | 2 | 3 | 4 | 5 | 6 | 7 | 8 | 9 | NA |
| 60B | Adding lithium | 0 | 1 | 2 | 3 | 4 | 5 | 6 | 7 | 8 | 9 | NA |
| 60C | Adding thyroid hormones | 0 | 1 | 2 | 3 | 4 | 5 | 6 | 7 | 8 | 9 | NA |
| 60D1 | Adding first-generation antipsychotic (e.g Haloperidol) | 0 | 1 | 2 | 3 | 4 | 5 | 6 | 7 | 8 | 9 | NA |
| 60D2 | Adding second-generation antipsychotic (e.g Risperidone) | 0 | 1 | 2 | 3 | 4 | 5 | 6 | 7 | 8 | 9 | NA |
| 60E1 | Pramipexole in combination | 0 | 1 | 2 | 3 | 4 | 5 | 6 | 7 | 8 | 9 | NA |
| 60E2 | Modafinil in combination | 0 | 1 | 2 | 3 | 4 | 5 | 6 | 7 | 8 | 9 | NA |
| 60F | Methylphenidate in combination | 0 | 1 | 2 | 3 | 4 | 5 | 6 | 7 | 8 | 9 | NA |

| 60 (Continuated) | An adult patient under the age of 65, with no significant organic or psychiatric history, has a depressive unipolar episode with high suicide risk  What treatment do you recommend?  *Circle the number corresponding to your choice* |
| --- | --- |

| 60G1 | Lamotrigine in combination | 0 | 1 | 2 | 3 | 4 | 5 | 6 | 7 | 8 | 9 | NA |
| --- | --- | --- | --- | --- | --- | --- | --- | --- | --- | --- | --- | --- |
| 60G2 | Valproic acide derivatives (e.g Divalproex sodium) in combination | 0 | 1 | 2 | 3 | 4 | 5 | 6 | 7 | 8 | 9 | NA |
| 60G3 | Carbamazepine in combination | 0 | 1 | 2 | 3 | 4 | 5 | 6 | 7 | 8 | 9 | NA |
| 60H1 | Structured psychotherpy in monotherapy | 0 | 1 | 2 | 3 | 4 | 5 | 6 | 7 | 8 | 9 | NA |
| 60H2 | Structured psychotherpy in combination | 0 | 1 | 2 | 3 | 4 | 5 | 6 | 7 | 8 | 9 | NA |
| 60I1 | ECT in monotherapy | 0 | 1 | 2 | 3 | 4 | 5 | 6 | 7 | 8 | 9 | NA |
| 60I2 | ECT in combination | 0 | 1 | 2 | 3 | 4 | 5 | 6 | 7 | 8 | 9 | NA |
| 60I3 | rTMS in monotherapy | 0 | 1 | 2 | 3 | 4 | 5 | 6 | 7 | 8 | 9 | NA |
| 60I4 | rTMS in combination | 0 | 1 | 2 | 3 | 4 | 5 | 6 | 7 | 8 | 9 | NA |

**Question 61**

0 means that this strategy is not recommended in first intention

1, 2, 3 means that this strategy is recommended in third intention

4, 5, 6 means that this strategy is recommended in second intention

7, 8, 9 means that this strategy is recommended in first intention.

NA means that you have no opinion and/or experience with this strategy in this indication.

### B. 2^nd^ line Strategies

**a) in case of partial response to the first line treatment**

| 61 | An adult patient under the age of 65, with no significant organic or psychiatric history, has a depressive unipolar episode treated by SSRI in monotherapy of adequate dosage and duration. No response was obtained.  What strategy(s) do you recommend?  *Circle the number corresponding to your choice* |
| --- | --- |

| 61A | Optimising the dose of the initial antidepressant | 0 | 1 | 2 | 3 | 4 | 5 | 6 | 7 | 8 | 9 | NA |
| --- | --- | --- | --- | --- | --- | --- | --- | --- | --- | --- | --- | --- |
| 61B1 | Switch to another SSRI in monotherapy  (e.g Citalopram) | 0 | 1 | 2 | 3 | 4 | 5 | 6 | 7 | 8 | 9 | NA |
| 61B2 | Switch to a SNRI in monotherapy  (e.g Venlafaxine) | 0 | 1 | 2 | 3 | 4 | 5 | 6 | 7 | 8 | 9 | NA |
| 61B3 | Switch to a tricyclic antidepressant in monotherapy  (e.g Clomipramine) | 0 | 1 | 2 | 3 | 4 | 5 | 6 | 7 | 8 | 9 | NA |
| 61B4 | Switch to Mianserine / Mirtazapine in monotherapy | 0 | 1 | 2 | 3 | 4 | 5 | 6 | 7 | 8 | 9 | NA |
| 61B5 | Switch to Tianeptine in monotherapy | 0 | 1 | 2 | 3 | 4 | 5 | 6 | 7 | 8 | 9 | NA |
| 61B6 | Switch to Agomelatine in monotherapy | 0 | 1 | 2 | 3 | 4 | 5 | 6 | 7 | 8 | 9 | NA |
| 61B7 | Switch to MAOI A (Moclobemide) in monotherapy | 0 | 1 | 2 | 3 | 4 | 5 | 6 | 7 | 8 | 9 | NA |
| 61B8 | Switch to a non selectif irreversible MAOI (Iproniazide) in monotherapy | 0 | 1 | 2 | 3 | 4 | 5 | 6 | 7 | 8 | 9 | NA |
| 61B9 | Switch to Bupropion in monotherapy | 0 | 1 | 2 | 3 | 4 | 5 | 6 | 7 | 8 | 9 | NA |
| 61C1 | Adding a SSRI | 0 | 1 | 2 | 3 | 4 | 5 | 6 | 7 | 8 | 9 | NA |
| 61C2 | Adding a SNRI | 0 | 1 | 2 | 3 | 4 | 5 | 6 | 7 | 8 | 9 | NA |
| 61C3 | Adding a tricyclic antidepressant | 0 | 1 | 2 | 3 | 4 | 5 | 6 | 7 | 8 | 9 | NA |
| 61C4 | Adding Mianserine / Mirtazapine | 0 | 1 | 2 | 3 | 4 | 5 | 6 | 7 | 8 | 9 | NA |

| 61 (Continuated) | An adult patient under the age of 65, with no significant organic or psychiatric history, has a depressive unipolar episode treated by SSRI in monotherapy of adequate dosage and duration. No response was obtained.  What strategy(s) do you recommend?  *Circle the number corresponding to your choice* |
| --- | --- |

| 61C5 | Adding Tianeptine | 0 | 1 | 2 | 3 | 4 | 5 | 6 | 7 | 8 | 9 | NA |
| --- | --- | --- | --- | --- | --- | --- | --- | --- | --- | --- | --- | --- |
| 61C6 | Adding Agomelatine | 0 | 1 | 2 | 3 | 4 | 5 | 6 | 7 | 8 | 9 | NA |
| 61C7 | Adding MAOI A (Moclobemide) | 0 | 1 | 2 | 3 | 4 | 5 | 6 | 7 | 8 | 9 | NA |
| 61C8 | Adding Bupropion | 0 | 1 | 2 | 3 | 4 | 5 | 6 | 7 | 8 | 9 | NA |
| 61D1 | Adding lithium | 0 | 1 | 2 | 3 | 4 | 5 | 6 | 7 | 8 | 9 | NA |
| 61D2 | Adding thyroid hormones | 0 | 1 | 2 | 3 | 4 | 5 | 6 | 7 | 8 | 9 | NA |
| 61D3 | Adding first-generation antipsychotic (e.g Haloperidol) | 0 | 1 | 2 | 3 | 4 | 5 | 6 | 7 | 8 | 9 | NA |
| 61D4 | Adding second-generation antipsychotic (e.g Risperidone) | 0 | 1 | 2 | 3 | 4 | 5 | 6 | 7 | 8 | 9 | NA |
| 61E1 | Pramipexole in combination | 0 | 1 | 2 | 3 | 4 | 5 | 6 | 7 | 8 | 9 | NA |
| 61E2 | Modafinil in combination | 0 | 1 | 2 | 3 | 4 | 5 | 6 | 7 | 8 | 9 | NA |
| 61E3 | Methylphenidate in combination | 0 | 1 | 2 | 3 | 4 | 5 | 6 | 7 | 8 | 9 | NA |
| 61E4 | Lamotrigine in combination | 0 | 1 | 2 | 3 | 4 | 5 | 6 | 7 | 8 | 9 | NA |
| 61E5 | Valproic acide derivatives (e.g Divalproex sodium) in combination | 0 | 1 | 2 | 3 | 4 | 5 | 6 | 7 | 8 | 9 | NA |
| 61E6 | Carbamazepine in combination | 0 | 1 | 2 | 3 | 4 | 5 | 6 | 7 | 8 | 9 | NA |
| 61E7 | Pregabaline in combination | 0 | 1 | 2 | 3 | 4 | 5 | 6 | 7 | 8 | 9 | NA |
| 61F1 | Structured psychotherpy in monotherapy | 0 | 1 | 2 | 3 | 4 | 5 | 6 | 7 | 8 | 9 | NA |
| 61F2 | Structured psychotherpy in combination | 0 | 1 | 2 | 3 | 4 | 5 | 6 | 7 | 8 | 9 | NA |
| 61G1 | ECT in monotherapy | 0 | 1 | 2 | 3 | 4 | 5 | 6 | 7 | 8 | 9 | NA |
| 61G2 | ECT in combination | 0 | 1 | 2 | 3 | 4 | 5 | 6 | 7 | 8 | 9 | NA |
| 61G3 | rTMS in monotherapy | 0 | 1 | 2 | 3 | 4 | 5 | 6 | 7 | 8 | 9 | NA |
| 61G4 | rTMS in combination | 0 | 1 | 2 | 3 | 4 | 5 | 6 | 7 | 8 | 9 | NA |

**Question 62**

0 means that this strategy is not recommended in first intention.

1, 2, 3 means that this strategy is recommended in third intention.

4, 5, 6 means that this strategy is recommended in second intention.

7, 8, 9 means that this strategy is recommended in first intention.

NA means that you have no opinion and/or experience with this strategy in this indication.

| 62 | An adult patient under the age of 65, with no significant organic or psychiatric history, has a depressive unipolar episode treated by SNRI in monotherapy of adequate dosage and duration. No response was obtained.  What strategy(s) do you recommend?  *Circle the number corresponding to your choice* |
| --- | --- |

| 62A | Optimising the dose of the initial antidepressant | 0 | 1 | 2 | 3 | 4 | 5 | 6 | 7 | 8 | 9 | NA |
| --- | --- | --- | --- | --- | --- | --- | --- | --- | --- | --- | --- | --- |
| 62B1 | Switch to a SSRI in monotherapy  (e.g Citalopram) | 0 | 1 | 2 | 3 | 4 | 5 | 6 | 7 | 8 | 9 | NA |
| 62B2 | Switch to another SNRI in monotherapy  (e.g Venlafaxine) | 0 | 1 | 2 | 3 | 4 | 5 | 6 | 7 | 8 | 9 | NA |
| 62B3 | Switch to a tricyclic antidepressant in monotherapy  (e.g Clomipramine) | 0 | 1 | 2 | 3 | 4 | 5 | 6 | 7 | 8 | 9 | NA |
| 62B4 | Switch to Mianserine / Mirtazapine in monotherapy | 0 | 1 | 2 | 3 | 4 | 5 | 6 | 7 | 8 | 9 | NA |
| 62B5 | Switch to Tianeptine in monotherapy | 0 | 1 | 2 | 3 | 4 | 5 | 6 | 7 | 8 | 9 | NA |
| 62B6 | Switch to Agomelatine in monotherapy | 0 | 1 | 2 | 3 | 4 | 5 | 6 | 7 | 8 | 9 | NA |
| 62B7 | Switch to MAOI A (Moclobemide) in monotherapy | 0 | 1 | 2 | 3 | 4 | 5 | 6 | 7 | 8 | 9 | NA |
| 62B8 | Switch to a non selectif irreversible MAOI (Iproniazide) in monotherapy | 0 | 1 | 2 | 3 | 4 | 5 | 6 | 7 | 8 | 9 | NA |
| 62B9 | Switch to Bupropion in monotherapy | 0 | 1 | 2 | 3 | 4 | 5 | 6 | 7 | 8 | 9 | NA |
| 62C1 | Adding a SSRI | 0 | 1 | 2 | 3 | 4 | 5 | 6 | 7 | 8 | 9 | NA |
| 62C2 | Adding a SNRI | 0 | 1 | 2 | 3 | 4 | 5 | 6 | 7 | 8 | 9 | NA |
| 62C3 | Adding a tricyclic antidepressant | 0 | 1 | 2 | 3 | 4 | 5 | 6 | 7 | 8 | 9 | NA |
| 62C4 | Adding Mianserine / Mirtazapine | 0 | 1 | 2 | 3 | 4 | 5 | 6 | 7 | 8 | 9 | NA |
| 62C5 | Adding Tianeptine | 0 | 1 | 2 | 3 | 4 | 5 | 6 | 7 | 8 | 9 | NA |
| 62C6 | Adding Agomelatine | 0 | 1 | 2 | 3 | 4 | 5 | 6 | 7 | 8 | 9 | NA |
| 62C7 | Adding MAOI A (Moclobemide) | 0 | 1 | 2 | 3 | 4 | 5 | 6 | 7 | 8 | 9 | NA |
| 62C8 | Adding Bupropion | 0 | 1 | 2 | 3 | 4 | 5 | 6 | 7 | 8 | 9 | NA |

| 62 (Continuated) | An adult patient under the age of 65, with no significant organic or psychiatric history, has a depressive unipolar episode treated by SNRI in monotherapy of adequate dosage and duration. No response was obtained.  What strategy(s) do you recommend?  *Circle the number corresponding to your choice* |
| --- | --- |

| 62D1 | Adding lithium | 0 | 1 | 2 | 3 | 4 | 5 | 6 | 7 | 8 | 9 | NA |
| --- | --- | --- | --- | --- | --- | --- | --- | --- | --- | --- | --- | --- |
| 62D2 | Adding thyroid hormones | 0 | 1 | 2 | 3 | 4 | 5 | 6 | 7 | 8 | 9 | NA |
| 62D3 | Adding first-generation antipsychotic (e.g Haloperidol) | 0 | 1 | 2 | 3 | 4 | 5 | 6 | 7 | 8 | 9 | NA |
| 62D4 | Adding second-generation antipsychotic (e.g Risperidone) | 0 | 1 | 2 | 3 | 4 | 5 | 6 | 7 | 8 | 9 | NA |
| 62E1 | Pramipexole in combination | 0 | 1 | 2 | 3 | 4 | 5 | 6 | 7 | 8 | 9 | NA |
| 62E2 | Modafinil in combination | 0 | 1 | 2 | 3 | 4 | 5 | 6 | 7 | 8 | 9 | NA |
| 62E3 | Methylphenidate in combination | 0 | 1 | 2 | 3 | 4 | 5 | 6 | 7 | 8 | 9 | NA |
| 62E4 | Lamotrigine in combination | 0 | 1 | 2 | 3 | 4 | 5 | 6 | 7 | 8 | 9 | NA |
| 62E5 | Valproic acide derivatives (e.g Divalproex sodium) in combination | 0 | 1 | 2 | 3 | 4 | 5 | 6 | 7 | 8 | 9 | NA |
| 62E6 | Carbamazepine in combination | 0 | 1 | 2 | 3 | 4 | 5 | 6 | 7 | 8 | 9 | NA |
| 62E7 | Pregabaline in combination | 0 | 1 | 2 | 3 | 4 | 5 | 6 | 7 | 8 | 9 | NA |
| 62F1 | Structured psychotherpy in monotherapy | 0 | 1 | 2 | 3 | 4 | 5 | 6 | 7 | 8 | 9 | NA |
| 62F2 | Structured psychotherpy in combination | 0 | 1 | 2 | 3 | 4 | 5 | 6 | 7 | 8 | 9 | NA |
| 62G1 | ECT in monotherapy | 0 | 1 | 2 | 3 | 4 | 5 | 6 | 7 | 8 | 9 | NA |
| 62G2 | ECT in combination | 0 | 1 | 2 | 3 | 4 | 5 | 6 | 7 | 8 | 9 | NA |
| 62G3 | rTMS in monotherapy | 0 | 1 | 2 | 3 | 4 | 5 | 6 | 7 | 8 | 9 | NA |
| 62G4 | rTMS in combination | 0 | 1 | 2 | 3 | 4 | 5 | 6 | 7 | 8 | 9 | NA |

**Question 63**

0 means that this strategy is not recommended in first intention.

1, 2, 3 means that this strategy is recommended in third intention.

4, 5, 6 means that this strategy is recommended in second intention.

7, 8, 9 means that this strategy is recommended in first intention.

NA means that you have no opinion and/or experience with this strategy in this indication.

| 63 | An adult patient under the age of 65, with no significant organic or psychiatric history, has a depressive unipolar episode treated by tricyclic antidepressant in monotherapy of adequate dosage and duration. No response was obtained.  What strategy(s) do you recommend?  *Circle the number corresponding to your choice* |
| --- | --- |

| 63A | Optimising the dose of the initial antidepressant | 0 | 1 | 2 | 3 | 4 | 5 | 6 | 7 | 8 | 9 | NA |
| --- | --- | --- | --- | --- | --- | --- | --- | --- | --- | --- | --- | --- |
| 63B1 | Switch to a SSRI in monotherapy  (e.g Citalopram) | 0 | 1 | 2 | 3 | 4 | 5 | 6 | 7 | 8 | 9 | NA |
| 63B2 | Switch to a SNRI in monotherapy  (e.g Venlafaxine) | 0 | 1 | 2 | 3 | 4 | 5 | 6 | 7 | 8 | 9 | NA |
| 63B3 | Switch to a tricyclic antidepressant in monotherapy  (e.g Clomipramine) | 0 | 1 | 2 | 3 | 4 | 5 | 6 | 7 | 8 | 9 | NA |
| 63B4 | Switch to Mianserine / Mirtazapine in monotherapy | 0 | 1 | 2 | 3 | 4 | 5 | 6 | 7 | 8 | 9 | NA |
| 63B5 | Switch to Tianeptine in monotherapy | 0 | 1 | 2 | 3 | 4 | 5 | 6 | 7 | 8 | 9 | NA |
| 63B6 | Switch to Agomelatine in monotherapy | 0 | 1 | 2 | 3 | 4 | 5 | 6 | 7 | 8 | 9 | NA |
| 63B7 | Switch to MAOI A (Moclobemide) in monotherapy | 0 | 1 | 2 | 3 | 4 | 5 | 6 | 7 | 8 | 9 | NA |
| 63B8 | Switch to a non selectif irreversible MAOI (Iproniazide) in monotherapy | 0 | 1 | 2 | 3 | 4 | 5 | 6 | 7 | 8 | 9 | NA |
| 63B9 | Switch to Bupropion in monotherapy | 0 | 1 | 2 | 3 | 4 | 5 | 6 | 7 | 8 | 9 | NA |
| 63C1 | Adding a SSRI | 0 | 1 | 2 | 3 | 4 | 5 | 6 | 7 | 8 | 9 | NA |
| 63C2 | Adding a SNRI | 0 | 1 | 2 | 3 | 4 | 5 | 6 | 7 | 8 | 9 | NA |
| 63C3 | Adding a tricyclic antidepressant | 0 | 1 | 2 | 3 | 4 | 5 | 6 | 7 | 8 | 9 | NA |
| 63C4 | Adding Mianserine / Mirtazapine | 0 | 1 | 2 | 3 | 4 | 5 | 6 | 7 | 8 | 9 | NA |
| 63C5 | Adding Tianeptine | 0 | 1 | 2 | 3 | 4 | 5 | 6 | 7 | 8 | 9 | NA |
| 63C6 | Adding Agomelatine | 0 | 1 | 2 | 3 | 4 | 5 | 6 | 7 | 8 | 9 | NA |
| 63C7 | Adding MAOI A (Moclobemide) | 0 | 1 | 2 | 3 | 4 | 5 | 6 | 7 | 8 | 9 | NA |
| 63C8 | Adding Bupropion | 0 | 1 | 2 | 3 | 4 | 5 | 6 | 7 | 8 | 9 | NA |

| 63 (Continuated) | An adult patient under the age of 65, with no significant organic or psychiatric history, has a depressive unipolar episode treated by tricyclic antidepressant in monotherapy of adequate dosage and duration. No response was obtained  What strategy(s) do you recommend?  *Circle the number corresponding to your choice* |
| --- | --- |

| 63D1 | Adding lithium | 0 | 1 | 2 | 3 | 4 | 5 | 6 | 7 | 8 | 9 | NA |
| --- | --- | --- | --- | --- | --- | --- | --- | --- | --- | --- | --- | --- |
| 63D2 | Adding thyroid hormones | 0 | 1 | 2 | 3 | 4 | 5 | 6 | 7 | 8 | 9 | NA |
| 63D3 | Adding first-generation antipsychotic (e.g Haloperidol) | 0 | 1 | 2 | 3 | 4 | 5 | 6 | 7 | 8 | 9 | NA |
| 63D4 | Adding second-generation antipsychotic (e.g Risperidone) | 0 | 1 | 2 | 3 | 4 | 5 | 6 | 7 | 8 | 9 | NA |
| 63E1 | Pramipexole in combination | 0 | 1 | 2 | 3 | 4 | 5 | 6 | 7 | 8 | 9 | NA |
| 63E2 | Modafinil in combination | 0 | 1 | 2 | 3 | 4 | 5 | 6 | 7 | 8 | 9 | NA |
| 63E3 | Methylphenidate in combination | 0 | 1 | 2 | 3 | 4 | 5 | 6 | 7 | 8 | 9 | NA |
| 63E4 | Lamotrigine in combination | 0 | 1 | 2 | 3 | 4 | 5 | 6 | 7 | 8 | 9 | NA |
| 63E5 | Valproic acide derivatives (e.g Divalproex sodium) in combination | 0 | 1 | 2 | 3 | 4 | 5 | 6 | 7 | 8 | 9 | NA |
| 63E6 | Carbamazepine in combination | 0 | 1 | 2 | 3 | 4 | 5 | 6 | 7 | 8 | 9 | NA |
| 63E7 | Pregabaline in combination | 0 | 1 | 2 | 3 | 4 | 5 | 6 | 7 | 8 | 9 | NA |
| 63F1 | Structured psychotherpy in monotherapy | 0 | 1 | 2 | 3 | 4 | 5 | 6 | 7 | 8 | 9 | NA |
| 63F2 | Structured psychotherpy in combination | 0 | 1 | 2 | 3 | 4 | 5 | 6 | 7 | 8 | 9 | NA |
| 63G1 | ECT in monotherapy | 0 | 1 | 2 | 3 | 4 | 5 | 6 | 7 | 8 | 9 | NA |
| 63G2 | ECT in combination | 0 | 1 | 2 | 3 | 4 | 5 | 6 | 7 | 8 | 9 | NA |
| 63G3 | rTMS in monotherapy | 0 | 1 | 2 | 3 | 4 | 5 | 6 | 7 | 8 | 9 | NA |
| 63G4 | rTMS in combination | 0 | 1 | 2 | 3 | 4 | 5 | 6 | 7 | 8 | 9 | NA |

**Question 64**

0 means that this strategy is not recommended in first intention.

1, 2, 3 means that this strategy is recommended in third intention.

4, 5, 6 means that this strategy is recommended in second intention.

7, 8, 9 means that this strategy is recommended in first intention.

NA means that you have no opinion and/or experience with this strategy in this indication.

| 64 | An adult patient under the age of 65, with no significant organic or psychiatric history, has a depressive unipolar episode treated by Mirtazapine/Mianserine in monotherapy of adequate dosage and duration. No response was obtained.  What strategy(s) do you recommend?  *Circle the number corresponding to your choice* |
| --- | --- |

| 64A | Optimising the dose of the initial antidepressant | 0 | 1 | 2 | 3 | 4 | 5 | 6 | 7 | 8 | 9 | NA |
| --- | --- | --- | --- | --- | --- | --- | --- | --- | --- | --- | --- | --- |
| 64B1 | Switch to a SSRI in monotherapy  (e.g Citalopram) | 0 | 1 | 2 | 3 | 4 | 5 | 6 | 7 | 8 | 9 | NA |
| 64B2 | Switch to a SNRI in monotherapy  (e.g Venlafaxine) | 0 | 1 | 2 | 3 | 4 | 5 | 6 | 7 | 8 | 9 | NA |
| 64B3 | Switch to a tricyclic antidepressant in monotherapy  (e.g Clomipramine) | 0 | 1 | 2 | 3 | 4 | 5 | 6 | 7 | 8 | 9 | NA |
| 64B4 | Switch to Mianserine / Mirtazapine in monotherapy | 0 | 1 | 2 | 3 | 4 | 5 | 6 | 7 | 8 | 9 | NA |
| 64B5 | Switch to Tianeptine in monotherapy | 0 | 1 | 2 | 3 | 4 | 5 | 6 | 7 | 8 | 9 | NA |
| 64B6 | Switch to Agomelatine in monotherapy | 0 | 1 | 2 | 3 | 4 | 5 | 6 | 7 | 8 | 9 | NA |
| 64B7 | Switch to MAOI A (Moclobemide) in monotherapy | 0 | 1 | 2 | 3 | 4 | 5 | 6 | 7 | 8 | 9 | NA |
| 64B8 | Switch to a non selectif irreversible MAOI (Iproniazide) in monotherapy | 0 | 1 | 2 | 3 | 4 | 5 | 6 | 7 | 8 | 9 | NA |
| 64B9 | Switch to Bupropion in monotherapy | 0 | 1 | 2 | 3 | 4 | 5 | 6 | 7 | 8 | 9 | NA |
| 64C1 | Adding a SSRI | 0 | 1 | 2 | 3 | 4 | 5 | 6 | 7 | 8 | 9 | NA |
| 64C2 | Adding a SNRI | 0 | 1 | 2 | 3 | 4 | 5 | 6 | 7 | 8 | 9 | NA |
| 64C3 | Adding a tricyclic antidepressant | 0 | 1 | 2 | 3 | 4 | 5 | 6 | 7 | 8 | 9 | NA |
| 64C4 | Adding Mianserine / Mirtazapine | 0 | 1 | 2 | 3 | 4 | 5 | 6 | 7 | 8 | 9 | NA |
| 64C5 | Adding Tianeptine | 0 | 1 | 2 | 3 | 4 | 5 | 6 | 7 | 8 | 9 | NA |
| 64C6 | Adding Agomelatine | 0 | 1 | 2 | 3 | 4 | 5 | 6 | 7 | 8 | 9 | NA |
| 64C7 | Adding MAOI A (Moclobemide) | 0 | 1 | 2 | 3 | 4 | 5 | 6 | 7 | 8 | 9 | NA |
| 64C8 | Adding Bupropion | 0 | 1 | 2 | 3 | 4 | 5 | 6 | 7 | 8 | 9 | NA |

| 64 (Continuated) | An adult patient under the age of 65, with no significant organic or psychiatric history, has a depressive unipolar episode treated by Mirtazapine/Mianserine in monotherapy of adequate dosage and duration. No response was obtained  What strategy(s) do you recommend?  *Circle the number corresponding to your choice* |
| --- | --- |

| 64D1 | Adding lithium | 0 | 1 | 2 | 3 | 4 | 5 | 6 | 7 | 8 | 9 | NA |
| --- | --- | --- | --- | --- | --- | --- | --- | --- | --- | --- | --- | --- |
| 64D2 | Adding thyroid hormones | 0 | 1 | 2 | 3 | 4 | 5 | 6 | 7 | 8 | 9 | NA |
| 64D3 | Adding first-generation antipsychotic (e.g Haloperidol) | 0 | 1 | 2 | 3 | 4 | 5 | 6 | 7 | 8 | 9 | NA |
| 64D4 | Adding second-generation antipsychotic (e.g Risperidone) | 0 | 1 | 2 | 3 | 4 | 5 | 6 | 7 | 8 | 9 | NA |
| 64E1 | Pramipexole in combination | 0 | 1 | 2 | 3 | 4 | 5 | 6 | 7 | 8 | 9 | NA |
| 64E2 | Modafinil in combination | 0 | 1 | 2 | 3 | 4 | 5 | 6 | 7 | 8 | 9 | NA |
| 64E3 | Methylphenidate in combination | 0 | 1 | 2 | 3 | 4 | 5 | 6 | 7 | 8 | 9 | NA |
| 64E4 | Lamotrigine in combination | 0 | 1 | 2 | 3 | 4 | 5 | 6 | 7 | 8 | 9 | NA |
| 64E5 | Valproic acide derivatives (e.g Divalproex sodium) in combination | 0 | 1 | 2 | 3 | 4 | 5 | 6 | 7 | 8 | 9 | NA |
| 64E6 | Carbamazepine in combination | 0 | 1 | 2 | 3 | 4 | 5 | 6 | 7 | 8 | 9 | NA |
| 64E7 | Pregabaline in combination | 0 | 1 | 2 | 3 | 4 | 5 | 6 | 7 | 8 | 9 | NA |
| 64F1 | Structured psychotherpy in monotherapy | 0 | 1 | 2 | 3 | 4 | 5 | 6 | 7 | 8 | 9 | NA |
| 64F2 | Structured psychotherpy in combination | 0 | 1 | 2 | 3 | 4 | 5 | 6 | 7 | 8 | 9 | NA |
| 64G1 | ECT in monotherapy | 0 | 1 | 2 | 3 | 4 | 5 | 6 | 7 | 8 | 9 | NA |
| 64G2 | ECT in combination | 0 | 1 | 2 | 3 | 4 | 5 | 6 | 7 | 8 | 9 | NA |
| 64G3 | rTMS in monotherapy | 0 | 1 | 2 | 3 | 4 | 5 | 6 | 7 | 8 | 9 | NA |
| 64G4 | rTMS in combination | 0 | 1 | 2 | 3 | 4 | 5 | 6 | 7 | 8 | 9 | NA |

**Question 65**

0 means that this strategy is not recommended in first intention.

1, 2, 3 means that this strategy is recommended in third intention.

4, 5, 6 means that this strategy is recommended in second intention.

7, 8, 9 means that this strategy is recommended in first intention.

NA means that you have no opinion and/or experience with this strategy in this indication.

**b) IN CASE OF NON-RESPONSE TO THE FIRST LINE TREATMENT**

| 65 | An adult patient under the age of 65, with no significant organic or psychiatric history, has a depressive unipolar episode treated by SSRI in monotherapy of adequate dosage and duration. No response was obtained.  What strategy(s) do you recommend?  *Circle the number corresponding to your choice* |
| --- | --- |

| 65A | Optimising the dose of the initial antidepressant | 0 | 1 | 2 | 3 | 4 | 5 | 6 | 7 | 8 | 9 | NA |
| --- | --- | --- | --- | --- | --- | --- | --- | --- | --- | --- | --- | --- |
| 65B1 | Switch to a SSRI in monotherapy  (e.g Citalopram) | 0 | 1 | 2 | 3 | 4 | 5 | 6 | 7 | 8 | 9 | NA |
| 65B2 | Switch to a SNRI in monotherapy  (e.g Venlafaxine) | 0 | 1 | 2 | 3 | 4 | 5 | 6 | 7 | 8 | 9 | NA |
| 65B3 | Switch to a tricyclic antidepressant in monotherapy  (e.g Clomipramine) | 0 | 1 | 2 | 3 | 4 | 5 | 6 | 7 | 8 | 9 | NA |
| 65B4 | Switch to Mianserine / Mirtazapine in monotherapy | 0 | 1 | 2 | 3 | 4 | 5 | 6 | 7 | 8 | 9 | NA |
| 65B5 | Switch to Tianeptine in monotherapy | 0 | 1 | 2 | 3 | 4 | 5 | 6 | 7 | 8 | 9 | NA |
| 65B6 | Switch to Agomelatine in monotherapy | 0 | 1 | 2 | 3 | 4 | 5 | 6 | 7 | 8 | 9 | NA |
| 65B7 | Switch to MAOI A (Moclobemide) in monotherapy | 0 | 1 | 2 | 3 | 4 | 5 | 6 | 7 | 8 | 9 | NA |
| 65B8 | Switch to a non selectif irreversible MAOI (Iproniazide) in monotherapy | 0 | 1 | 2 | 3 | 4 | 5 | 6 | 7 | 8 | 9 | NA |
| 65B9 | Switch to Bupropion in monotherapy | 0 | 1 | 2 | 3 | 4 | 5 | 6 | 7 | 8 | 9 | NA |
| 65C1 | Adding a SSRI | 0 | 1 | 2 | 3 | 4 | 5 | 6 | 7 | 8 | 9 | NA |
| 65C2 | Adding a SNRI | 0 | 1 | 2 | 3 | 4 | 5 | 6 | 7 | 8 | 9 | NA |
| 65C3 | Adding a tricyclic antidepressant | 0 | 1 | 2 | 3 | 4 | 5 | 6 | 7 | 8 | 9 | NA |
| 65C4 | Adding Mianserine / Mirtazapine | 0 | 1 | 2 | 3 | 4 | 5 | 6 | 7 | 8 | 9 | NA |
| 65C5 | Adding Tianeptine | 0 | 1 | 2 | 3 | 4 | 5 | 6 | 7 | 8 | 9 | NA |
| 65C6 | Adding Agomelatine | 0 | 1 | 2 | 3 | 4 | 5 | 6 | 7 | 8 | 9 | NA |
| 65C7 | Adding MAOI A (Moclobemide) | 0 | 1 | 2 | 3 | 4 | 5 | 6 | 7 | 8 | 9 | NA |
| 65C8 | Adding Bupropion | 0 | 1 | 2 | 3 | 4 | 5 | 6 | 7 | 8 | 9 | NA |

| 65 (Continuated) | An adult patient under the age of 65, with no significant organic or psychiatric history, has a depressive unipolar episode treated by SSRI in monotherapy of adequate dosage and duration. No response was obtained.  What strategy(s) do you recommend?  *Circle the number corresponding to your choice* |
| --- | --- |

| 65D1 | Adding lithium | 0 | 1 | 2 | 3 | 4 | 5 | 6 | 7 | 8 | 9 | NA |
| --- | --- | --- | --- | --- | --- | --- | --- | --- | --- | --- | --- | --- |
| 65D2 | Adding thyroid hormones | 0 | 1 | 2 | 3 | 4 | 5 | 6 | 7 | 8 | 9 | NA |
| 65D3 | Adding first-generation antipsychotic (e.g Haloperidol) | 0 | 1 | 2 | 3 | 4 | 5 | 6 | 7 | 8 | 9 | NA |
| 65D4 | Adding second-generation antipsychotic (e.g Risperidone) | 0 | 1 | 2 | 3 | 4 | 5 | 6 | 7 | 8 | 9 | NA |
| 65E1 | Pramipexole in combination | 0 | 1 | 2 | 3 | 4 | 5 | 6 | 7 | 8 | 9 | NA |
| 65E2 | Modafinil in combination | 0 | 1 | 2 | 3 | 4 | 5 | 6 | 7 | 8 | 9 | NA |
| 65E3 | Methylphenidate in combination | 0 | 1 | 2 | 3 | 4 | 5 | 6 | 7 | 8 | 9 | NA |
| 65E4 | Lamotrigine in combination | 0 | 1 | 2 | 3 | 4 | 5 | 6 | 7 | 8 | 9 | NA |
| 65E5 | Valproic acide derivatives (e.g Divalproex sodium) in combination | 0 | 1 | 2 | 3 | 4 | 5 | 6 | 7 | 8 | 9 | NA |
| 65E6 | Carbamazepine in combination | 0 | 1 | 2 | 3 | 4 | 5 | 6 | 7 | 8 | 9 | NA |
| 65E7 | Pregabaline in combination | 0 | 1 | 2 | 3 | 4 | 5 | 6 | 7 | 8 | 9 | NA |
| 65F1 | Structured psychotherpy in monotherapy | 0 | 1 | 2 | 3 | 4 | 5 | 6 | 7 | 8 | 9 | NA |
| 65F2 | Structured psychotherpy in combination | 0 | 1 | 2 | 3 | 4 | 5 | 6 | 7 | 8 | 9 | NA |
| 65G1 | ECT in monotherapy | 0 | 1 | 2 | 3 | 4 | 5 | 6 | 7 | 8 | 9 | NA |
| 65G2 | ECT in combination | 0 | 1 | 2 | 3 | 4 | 5 | 6 | 7 | 8 | 9 | NA |
| 65G3 | rTMS in monotherapy | 0 | 1 | 2 | 3 | 4 | 5 | 6 | 7 | 8 | 9 | NA |
| 65G4 | rTMS in combination | 0 | 1 | 2 | 3 | 4 | 5 | 6 | 7 | 8 | 9 | NA |

**Question 66**

0 means that this strategy is not recommended in first intention.

1, 2, 3 means that this strategy is recommended in third intention.

4, 5, 6 means that this strategy is recommended in second intention.

7, 8, 9 means that this strategy is recommended in first intention.

NA means that you have no opinion and/or experience with this strategy in this indication.

| 66 | An adult patient under the age of 65, with no significant organic or psychiatric history, has a depressive unipolar episode treated by SNRI in monotherapy of adequate dosage and duration. No response was obtained.  What strategy(s) do you recommend?  *Circle the number corresponding to your choice* |
| --- | --- |

| 66A | Optimising the dose of the initial antidepressant | 0 | 1 | 2 | 3 | 4 | 5 | 6 | 7 | 8 | 9 | NA |
| --- | --- | --- | --- | --- | --- | --- | --- | --- | --- | --- | --- | --- |
| 66B1 | Switch to a SSRI in monotherapy  (e.g Citalopram) | 0 | 1 | 2 | 3 | 4 | 5 | 6 | 7 | 8 | 9 | NA |
| 66B2 | Switch to a SNRI in monotherapy  (e.g Venlafaxine) | 0 | 1 | 2 | 3 | 4 | 5 | 6 | 7 | 8 | 9 | NA |
| 66B3 | Switch to a tricyclic antidepressant in monotherapy  (e.g Clomipramine) | 0 | 1 | 2 | 3 | 4 | 5 | 6 | 7 | 8 | 9 | NA |
| 66B4 | Switch to Mianserine / Mirtazapine in monotherapy | 0 | 1 | 2 | 3 | 4 | 5 | 6 | 7 | 8 | 9 | NA |
| 66B5 | Switch to Tianeptine in monotherapy | 0 | 1 | 2 | 3 | 4 | 5 | 6 | 7 | 8 | 9 | NA |
| 66B6 | Switch to Agomelatine in monotherapy | 0 | 1 | 2 | 3 | 4 | 5 | 6 | 7 | 8 | 9 | NA |
| 66B7 | Switch to MAOI A (Moclobemide) in monotherapy | 0 | 1 | 2 | 3 | 4 | 5 | 6 | 7 | 8 | 9 | NA |
| 66B8 | Switch to a non selectif irreversible MAOI (Iproniazide) in monotherapy | 0 | 1 | 2 | 3 | 4 | 5 | 6 | 7 | 8 | 9 | NA |
| 66B9 | Switch to Bupropion in monotherapy | 0 | 1 | 2 | 3 | 4 | 5 | 6 | 7 | 8 | 9 | NA |
| 66C1 | Adding a SSRI | 0 | 1 | 2 | 3 | 4 | 5 | 6 | 7 | 8 | 9 | NA |
| 66C2 | Adding a SNRI | 0 | 1 | 2 | 3 | 4 | 5 | 6 | 7 | 8 | 9 | NA |
| 66C3 | Adding a tricyclic antidepressant | 0 | 1 | 2 | 3 | 4 | 5 | 6 | 7 | 8 | 9 | NA |
| 66C4 | Adding Mianserine / Mirtazapine | 0 | 1 | 2 | 3 | 4 | 5 | 6 | 7 | 8 | 9 | NA |
| 66C5 | Adding Tianeptine | 0 | 1 | 2 | 3 | 4 | 5 | 6 | 7 | 8 | 9 | NA |
| 66C6 | Adding Agomelatine | 0 | 1 | 2 | 3 | 4 | 5 | 6 | 7 | 8 | 9 | NA |
| 66C7 | Adding MAOI A (Moclobemide) | 0 | 1 | 2 | 3 | 4 | 5 | 6 | 7 | 8 | 9 | NA |
| 66C8 | Adding Bupropion | 0 | 1 | 2 | 3 | 4 | 5 | 6 | 7 | 8 | 9 | NA |

| 66 (Continuated) | An adult patient under the age of 65, with no significant organic or psychiatric history, has a depressive unipolar episode treated by SNRI in monotherapy of adequate dosage and duration. No response was obtained.  What strategy(s) do you recommend?  *Circle the number corresponding to your choice* |
| --- | --- |

| 66D1 | Adding lithium | 0 | 1 | 2 | 3 | 4 | 5 | 6 | 7 | 8 | 9 | NA |
| --- | --- | --- | --- | --- | --- | --- | --- | --- | --- | --- | --- | --- |
| 66D2 | Adding thyroid hormones | 0 | 1 | 2 | 3 | 4 | 5 | 6 | 7 | 8 | 9 | NA |
| 66D3 | Adding first-generation antipsychotic (e.g Haloperidol) | 0 | 1 | 2 | 3 | 4 | 5 | 6 | 7 | 8 | 9 | NA |
| 66D4 | Adding second-generation antipsychotic (e.g Risperidone) | 0 | 1 | 2 | 3 | 4 | 5 | 6 | 7 | 8 | 9 | NA |
| 66E1 | Pramipexole in combination | 0 | 1 | 2 | 3 | 4 | 5 | 6 | 7 | 8 | 9 | NA |
| 66E2 | Modafinil in combination | 0 | 1 | 2 | 3 | 4 | 5 | 6 | 7 | 8 | 9 | NA |
| 66E3 | Methylphenidate in combination | 0 | 1 | 2 | 3 | 4 | 5 | 6 | 7 | 8 | 9 | NA |
| 66E4 | Lamotrigine in combination | 0 | 1 | 2 | 3 | 4 | 5 | 6 | 7 | 8 | 9 | NA |
| 66E5 | Valproic acide derivatives (e.g Divalproex sodium) in combination | 0 | 1 | 2 | 3 | 4 | 5 | 6 | 7 | 8 | 9 | NA |
| 66E6 | Carbamazepine in combination | 0 | 1 | 2 | 3 | 4 | 5 | 6 | 7 | 8 | 9 | NA |
| 66E7 | Pregabaline in combination | 0 | 1 | 2 | 3 | 4 | 5 | 6 | 7 | 8 | 9 | NA |
| 66F1 | Structured psychotherpy in monotherapy | 0 | 1 | 2 | 3 | 4 | 5 | 6 | 7 | 8 | 9 | NA |
| 66F2 | Structured psychotherpy in combination | 0 | 1 | 2 | 3 | 4 | 5 | 6 | 7 | 8 | 9 | NA |
| 66G1 | ECT in monotherapy | 0 | 1 | 2 | 3 | 4 | 5 | 6 | 7 | 8 | 9 | NA |
| 66G2 | ECT in combination | 0 | 1 | 2 | 3 | 4 | 5 | 6 | 7 | 8 | 9 | NA |
| 66G3 | rTMS in monotherapy | 0 | 1 | 2 | 3 | 4 | 5 | 6 | 7 | 8 | 9 | NA |
| 66G4 | rTMS in combination | 0 | 1 | 2 | 3 | 4 | 5 | 6 | 7 | 8 | 9 | NA |

**Question 67**

0 means that this strategy is not recommended in first intention.

1, 2, 3 means that this strategy is recommended in third intention.

4, 5, 6 means that this strategy is recommended in second intention.

7, 8, 9 means that this strategy is recommended in first intention.

NA means that you have no opinion and/or experience with this strategy in this indication.

| 67 | An adult patient under the age of 65, with no significant organic or psychiatric history, has a depressive unipolar episode treated by a tricyclic antidepressant in monotherapy of adequate dosage and duration. No response was obtained*.*  What strategy(s) do you recommend?  *Circle the number corresponding to your choice* |
| --- | --- |

| 67A | Optimising the dose of the initial antidepressant | 0 | 1 | 2 | 3 | 4 | 5 | 6 | 7 | 8 | 9 | NA |
| --- | --- | --- | --- | --- | --- | --- | --- | --- | --- | --- | --- | --- |
| 67B1 | Switch to a SSRI in monotherapy  (e.g Citalopram) | 0 | 1 | 2 | 3 | 4 | 5 | 6 | 7 | 8 | 9 | NA |
| 67B2 | Switch to a SNRI in monotherapy  (e.g Venlafaxine) | 0 | 1 | 2 | 3 | 4 | 5 | 6 | 7 | 8 | 9 | NA |
| 67B3 | Switch to a tricyclic antidepressant in monotherapy  (e.g Clomipramine) | 0 | 1 | 2 | 3 | 4 | 5 | 6 | 7 | 8 | 9 | NA |
| 67B4 | Switch to Mianserine / Mirtazapine in monotherapy | 0 | 1 | 2 | 3 | 4 | 5 | 6 | 7 | 8 | 9 | NA |
| 67B5 | Switch to Tianeptine in monotherapy | 0 | 1 | 2 | 3 | 4 | 5 | 6 | 7 | 8 | 9 | NA |
| 67B6 | Switch to Agomelatine in monotherapy | 0 | 1 | 2 | 3 | 4 | 5 | 6 | 7 | 8 | 9 | NA |
| 67B7 | Switch to MAOI A (Moclobemide) in monotherapy | 0 | 1 | 2 | 3 | 4 | 5 | 6 | 7 | 8 | 9 | NA |
| 67B8 | Switch to a non selectif irreversible MAOI (Iproniazide) in monotherapy | 0 | 1 | 2 | 3 | 4 | 5 | 6 | 7 | 8 | 9 | NA |
| 67B9 | Switch to Bupropion in monotherapy | 0 | 1 | 2 | 3 | 4 | 5 | 6 | 7 | 8 | 9 | NA |
| 67C1 | Adding a SSRI | 0 | 1 | 2 | 3 | 4 | 5 | 6 | 7 | 8 | 9 | NA |
| 67C2 | Adding a SNRI | 0 | 1 | 2 | 3 | 4 | 5 | 6 | 7 | 8 | 9 | NA |
| 67C3 | Adding a tricyclic antidepressant | 0 | 1 | 2 | 3 | 4 | 5 | 6 | 7 | 8 | 9 | NA |
| 67C4 | Adding Mianserine / Mirtazapine | 0 | 1 | 2 | 3 | 4 | 5 | 6 | 7 | 8 | 9 | NA |
| 67C5 | Adding Tianeptine | 0 | 1 | 2 | 3 | 4 | 5 | 6 | 7 | 8 | 9 | NA |
| 67C6 | Adding Agomelatine | 0 | 1 | 2 | 3 | 4 | 5 | 6 | 7 | 8 | 9 | NA |
| 67C7 | Adding MAOI A (Moclobemide) | 0 | 1 | 2 | 3 | 4 | 5 | 6 | 7 | 8 | 9 | NA |
| 67C8 | Adding Bupropion | 0 | 1 | 2 | 3 | 4 | 5 | 6 | 7 | 8 | 9 | NA |

| 67 (Continuated) | An adult patient under the age of 65, with no significant organic or psychiatric history, has a depressive unipolar episode treated by a tricyclic antidepressant in monotherapy of adequate dosage and duration. No response was obtained*.*.  What strategy(s) do you recommend?  *Circle the number corresponding to your choice* |
| --- | --- |

| 67D1 | Adding lithium | 0 | 1 | 2 | 3 | 4 | 5 | 6 | 7 | 8 | 9 | NA |
| --- | --- | --- | --- | --- | --- | --- | --- | --- | --- | --- | --- | --- |
| 67D2 | Adding thyroid hormones | 0 | 1 | 2 | 3 | 4 | 5 | 6 | 7 | 8 | 9 | NA |
| 67D3 | Adding first-generation antipsychotic (e.g Haloperidol) | 0 | 1 | 2 | 3 | 4 | 5 | 6 | 7 | 8 | 9 | NA |
| 67D4 | Adding second-generation antipsychotic (e.g Risperidone) | 0 | 1 | 2 | 3 | 4 | 5 | 6 | 7 | 8 | 9 | NA |
| 67E1 | Pramipexole in combination | 0 | 1 | 2 | 3 | 4 | 5 | 6 | 7 | 8 | 9 | NA |
| 67E2 | Modafinil in combination | 0 | 1 | 2 | 3 | 4 | 5 | 6 | 7 | 8 | 9 | NA |
| 67E3 | Methylphenidate in combination | 0 | 1 | 2 | 3 | 4 | 5 | 6 | 7 | 8 | 9 | NA |
| 67E4 | Lamotrigine in combination | 0 | 1 | 2 | 3 | 4 | 5 | 6 | 7 | 8 | 9 | NA |
| 67E5 | Valproic acide derivatives (e.g Divalproex sodium) in combination | 0 | 1 | 2 | 3 | 4 | 5 | 6 | 7 | 8 | 9 | NA |
| 67E6 | Carbamazepine in combination | 0 | 1 | 2 | 3 | 4 | 5 | 6 | 7 | 8 | 9 | NA |
| 67E7 | Pregabaline in combination | 0 | 1 | 2 | 3 | 4 | 5 | 6 | 7 | 8 | 9 | NA |
| 67F1 | Structured psychotherpy in monotherapy | 0 | 1 | 2 | 3 | 4 | 5 | 6 | 7 | 8 | 9 | NA |
| 67F2 | Structured psychotherpy in combination | 0 | 1 | 2 | 3 | 4 | 5 | 6 | 7 | 8 | 9 | NA |
| 67G1 | ECT in monotherapy | 0 | 1 | 2 | 3 | 4 | 5 | 6 | 7 | 8 | 9 | NA |
| 67G2 | ECT in combination | 0 | 1 | 2 | 3 | 4 | 5 | 6 | 7 | 8 | 9 | NA |
| 67G3 | rTMS in monotherapy | 0 | 1 | 2 | 3 | 4 | 5 | 6 | 7 | 8 | 9 | NA |
| 67G4 | rTMS in combination | 0 | 1 | 2 | 3 | 4 | 5 | 6 | 7 | 8 | 9 | NA |

**Question 68**

0 means that this strategy is not recommended in first intention

1, 2, 3 means that this strategy is recommended in third intention

4, 5, 6 means that this strategy is recommended in second intention

7, 8, 9 means that this strategy is recommended in first intention.

NA means that you have no opinion and/or experience with this strategy in this indication.

| 68 | An adult patient under the age of 65, with no significant organic or psychiatric history, has a depressive unipolar episode treated by Mirtazapine/Mianserine in monotherapy of adequate dosage and duration. No response was obtained.  What strategy(s) do you recommend?  *Circle the number corresponding to your choice* |
| --- | --- |

| 68A | Optimising the dose of the initial antidepressant | 0 | 1 | 2 | 3 | 4 | 5 | 6 | 7 | 8 | 9 | NA |
| --- | --- | --- | --- | --- | --- | --- | --- | --- | --- | --- | --- | --- |
| 68B1 | Switch to a SSRI in monotherapy  (e.g Citalopram) | 0 | 1 | 2 | 3 | 4 | 5 | 6 | 7 | 8 | 9 | NA |
| 68B2 | Switch to a SNRI in monotherapy  (e.g Venlafaxine) | 0 | 1 | 2 | 3 | 4 | 5 | 6 | 7 | 8 | 9 | NA |
| 68B3 | Switch to a tricyclic antidepressant in monotherapy  (e.g Clomipramine) | 0 | 1 | 2 | 3 | 4 | 5 | 6 | 7 | 8 | 9 | NA |
| 68B4 | Switch to Mianserine / Mirtazapine in monotherapy | 0 | 1 | 2 | 3 | 4 | 5 | 6 | 7 | 8 | 9 | NA |
| 68B5 | Switch to Tianeptine in monotherapy | 0 | 1 | 2 | 3 | 4 | 5 | 6 | 7 | 8 | 9 | NA |
| 68B6 | Switch to Agomelatine in monotherapy | 0 | 1 | 2 | 3 | 4 | 5 | 6 | 7 | 8 | 9 | NA |
| 68B7 | Switch to MAOI A (Moclobemide) in monotherapy | 0 | 1 | 2 | 3 | 4 | 5 | 6 | 7 | 8 | 9 | NA |
| 68B8 | Switch to a non selectif irreversible MAOI (Iproniazide) in monotherapy | 0 | 1 | 2 | 3 | 4 | 5 | 6 | 7 | 8 | 9 | NA |
| 68B9 | Switch to Bupropion in monotherapy | 0 | 1 | 2 | 3 | 4 | 5 | 6 | 7 | 8 | 9 | NA |
| 68C1 | Adding a SSRI | 0 | 1 | 2 | 3 | 4 | 5 | 6 | 7 | 8 | 9 | NA |
| 68C2 | Adding a SNRI | 0 | 1 | 2 | 3 | 4 | 5 | 6 | 7 | 8 | 9 | NA |
| 68C3 | Adding a tricyclic antidepressant | 0 | 1 | 2 | 3 | 4 | 5 | 6 | 7 | 8 | 9 | NA |
| 68C4 | Adding Mianserine / Mirtazapine | 0 | 1 | 2 | 3 | 4 | 5 | 6 | 7 | 8 | 9 | NA |
| 68C5 | Adding Tianeptine | 0 | 1 | 2 | 3 | 4 | 5 | 6 | 7 | 8 | 9 | NA |
| 68C6 | Adding Agomelatine | 0 | 1 | 2 | 3 | 4 | 5 | 6 | 7 | 8 | 9 | NA |
| 68C7 | Adding MAOI A (Moclobemide) | 0 | 1 | 2 | 3 | 4 | 5 | 6 | 7 | 8 | 9 | NA |
| 68C8 | Adding Bupropion | 0 | 1 | 2 | 3 | 4 | 5 | 6 | 7 | 8 | 9 | NA |

| 68 (Continuated) | An adult patient under the age of 65, with no significant organic or psychiatric history, has a depressive unipolar episode treated by Mirtazapine/Mianserine in monotherapy of adequate dosage and duration. No response was obtained.  What strategy(s) do you recommend?  *Circle the number corresponding to your choice* |
| --- | --- |

| 68D1 | Adding lithium | 0 | 1 | 2 | 3 | 4 | 5 | 6 | 7 | 8 | 9 | NA |
| --- | --- | --- | --- | --- | --- | --- | --- | --- | --- | --- | --- | --- |
| 68D2 | Adding thyroid hormones | 0 | 1 | 2 | 3 | 4 | 5 | 6 | 7 | 8 | 9 | NA |
| 68D3 | Adding first-generation antipsychotic (e.g Haloperidol) | 0 | 1 | 2 | 3 | 4 | 5 | 6 | 7 | 8 | 9 | NA |
| 68D4 | Adding second-generation antipsychotic (e.g Risperidone) | 0 | 1 | 2 | 3 | 4 | 5 | 6 | 7 | 8 | 9 | NA |
| 68E1 | Pramipexole in combination | 0 | 1 | 2 | 3 | 4 | 5 | 6 | 7 | 8 | 9 | NA |
| 68E2 | Modafinil in combination | 0 | 1 | 2 | 3 | 4 | 5 | 6 | 7 | 8 | 9 | NA |
| 68E3 | Methylphenidate in combination | 0 | 1 | 2 | 3 | 4 | 5 | 6 | 7 | 8 | 9 | NA |
| 68E4 | Lamotrigine in combination | 0 | 1 | 2 | 3 | 4 | 5 | 6 | 7 | 8 | 9 | NA |
| 68E5 | Valproic acide derivatives (e.g Divalproex sodium) in combination | 0 | 1 | 2 | 3 | 4 | 5 | 6 | 7 | 8 | 9 | NA |
| 68E6 | Carbamazepine in combination | 0 | 1 | 2 | 3 | 4 | 5 | 6 | 7 | 8 | 9 | NA |
| 68E7 | Pregabaline in combination | 0 | 1 | 2 | 3 | 4 | 5 | 6 | 7 | 8 | 9 | NA |
| 68F1 | Structured psychotherpy in monotherapy | 0 | 1 | 2 | 3 | 4 | 5 | 6 | 7 | 8 | 9 | NA |
| 68F2 | Structured psychotherpy in combination | 0 | 1 | 2 | 3 | 4 | 5 | 6 | 7 | 8 | 9 | NA |
| 68G1 | ECT in monotherapy | 0 | 1 | 2 | 3 | 4 | 5 | 6 | 7 | 8 | 9 | NA |
| 68G2 | ECT in combination | 0 | 1 | 2 | 3 | 4 | 5 | 6 | 7 | 8 | 9 | NA |
| 68G3 | rTMS in monotherapy | 0 | 1 | 2 | 3 | 4 | 5 | 6 | 7 | 8 | 9 | NA |
| 68G4 | rTMS in combination | 0 | 1 | 2 | 3 | 4 | 5 | 6 | 7 | 8 | 9 | NA |

**Question 69**

0 means that this strategy is not recommended in first intention

1, 2, 3 means that this strategy is recommended in third intention

4, 5, 6 means that this strategy is recommended in second intention

7, 8, 9 means that this strategy is recommended in first intention.

NA means that you have no opinion and/or experience with this strategy in this indication.

### C. 3^rd^ line Strategies

**a) IN CASE OF PARTIAL RESPONSE to the 2^nd^ line treatment**

| 69 | An adult patient under the age of 65, with no significant organic or psychiatric history, has a depressive unipolar episode treated successively by two different SSRI in monotherapy of adequate dosage and duration. No response was obtained.  What strategy(s) do you recommend?  *Circle the number corresponding to your choice* |
| --- | --- |

| 69A1 | Switch to a SSRI in monotherapy  (e.g Citalopram) | 0 | 1 | 2 | 3 | 4 | 5 | 6 | 7 | 8 | 9 | NA |
| --- | --- | --- | --- | --- | --- | --- | --- | --- | --- | --- | --- | --- |
| 69A2 | Switch to a SNRI in monotherapy  (e.g Venlafaxine) | 0 | 1 | 2 | 3 | 4 | 5 | 6 | 7 | 8 | 9 | NA |
| 69A3 | Switch to a tricyclic antidepressant in monotherapy  (e.g Clomipramine) | 0 | 1 | 2 | 3 | 4 | 5 | 6 | 7 | 8 | 9 | NA |
| 69A4 | Switch to Mianserine / Mirtazapine in monotherapy | 0 | 1 | 2 | 3 | 4 | 5 | 6 | 7 | 8 | 9 | NA |
| 69A5 | Switch to Tianeptine in monotherapy | 0 | 1 | 2 | 3 | 4 | 5 | 6 | 7 | 8 | 9 | NA |
| 69A6 | Switch to Agomelatine in monotherapy | 0 | 1 | 2 | 3 | 4 | 5 | 6 | 7 | 8 | 9 | NA |
| 69A7 | Switch to MAOI A (Moclobemide) in monotherapy | 0 | 1 | 2 | 3 | 4 | 5 | 6 | 7 | 8 | 9 | NA |
| 69A8 | Switch to a non selectif irreversible MAOI (Iproniazide) in monotherapy | 0 | 1 | 2 | 3 | 4 | 5 | 6 | 7 | 8 | 9 | NA |
| 69A9 | Switch to Bupropion in monotherapy | 0 | 1 | 2 | 3 | 4 | 5 | 6 | 7 | 8 | 9 | NA |
| 69B1 | Adding a SSRI | 0 | 1 | 2 | 3 | 4 | 5 | 6 | 7 | 8 | 9 | NA |
| 69B2 | Adding a SNRI | 0 | 1 | 2 | 3 | 4 | 5 | 6 | 7 | 8 | 9 | NA |
| 69B3 | Adding a tricyclic antidepressant | 0 | 1 | 2 | 3 | 4 | 5 | 6 | 7 | 8 | 9 | NA |
| 69B4 | Adding Mianserine / Mirtazapine | 0 | 1 | 2 | 3 | 4 | 5 | 6 | 7 | 8 | 9 | NA |
| 69B5 | Adding Tianeptine | 0 | 1 | 2 | 3 | 4 | 5 | 6 | 7 | 8 | 9 | NA |

| 69 (Continuated) | An adult patient under the age of 65, with no significant organic or psychiatric history, has a depressive unipolar episode treated successively by two different SSRI in monotherapy of adequate dosage and duration. No response was obtained.  What strategy(s) do you recommend?  *Circle the number corresponding to your choice* |
| --- | --- |

| 69B6 | Adding Agomelatine | 0 | 1 | 2 | 3 | 4 | 5 | 6 | 7 | 8 | 9 | NA |
| --- | --- | --- | --- | --- | --- | --- | --- | --- | --- | --- | --- | --- |
| 69B7 | Adding MAOI A (Moclobemide) | 0 | 1 | 2 | 3 | 4 | 5 | 6 | 7 | 8 | 9 | NA |
| 69B8 | Adding Bupropion | 0 | 1 | 2 | 3 | 4 | 5 | 6 | 7 | 8 | 9 | NA |
| 69C1 | Adding lithium | 0 | 1 | 2 | 3 | 4 | 5 | 6 | 7 | 8 | 9 | NA |
| 69C2 | Adding thyroid hormones | 0 | 1 | 2 | 3 | 4 | 5 | 6 | 7 | 8 | 9 | NA |
| 69C3 | Adding first-generation antipsychotic (e.g Haloperidol) | 0 | 1 | 2 | 3 | 4 | 5 | 6 | 7 | 8 | 9 | NA |
| 69C4 | Adding second-generation antipsychotic (e.g Risperidone) | 0 | 1 | 2 | 3 | 4 | 5 | 6 | 7 | 8 | 9 | NA |
| 69D1 | Pramipexole in combination | 0 | 1 | 2 | 3 | 4 | 5 | 6 | 7 | 8 | 9 | NA |
| 69D2 | Modafinil in combination | 0 | 1 | 2 | 3 | 4 | 5 | 6 | 7 | 8 | 9 | NA |
| 69D3 | Methylphenidate in combination | 0 | 1 | 2 | 3 | 4 | 5 | 6 | 7 | 8 | 9 | NA |
| 69D4 | Lamotrigine in combination | 0 | 1 | 2 | 3 | 4 | 5 | 6 | 7 | 8 | 9 | NA |
| 69D5 | Valproic acide derivatives (e.g Divalproex sodium) in combination | 0 | 1 | 2 | 3 | 4 | 5 | 6 | 7 | 8 | 9 | NA |
| 69D6 | Carbamazepine in combination | 0 | 1 | 2 | 3 | 4 | 5 | 6 | 7 | 8 | 9 | NA |
| 69D7 | Pregabaline in combination | 0 | 1 | 2 | 3 | 4 | 5 | 6 | 7 | 8 | 9 | NA |
| 69E1 | Structured psychotherpy in monotherapy | 0 | 1 | 2 | 3 | 4 | 5 | 6 | 7 | 8 | 9 | NA |
| 69E2 | Structured psychotherpy in combination | 0 | 1 | 2 | 3 | 4 | 5 | 6 | 7 | 8 | 9 | NA |
| 69F1 | ECT in monotherapy | 0 | 1 | 2 | 3 | 4 | 5 | 6 | 7 | 8 | 9 | NA |
| 69F2 | ECT in combination | 0 | 1 | 2 | 3 | 4 | 5 | 6 | 7 | 8 | 9 | NA |
| 69F3 | rTMS in monotherapy | 0 | 1 | 2 | 3 | 4 | 5 | 6 | 7 | 8 | 9 | NA |
| 69F4 | rTMS in combination | 0 | 1 | 2 | 3 | 4 | 5 | 6 | 7 | 8 | 9 | NA |

**Question 70**

0 means that this strategy is not recommended in first intention.

1, 2, 3 means that this strategy is recommended in third intention.

4, 5, 6 means that this strategy is recommended in second intention.

7, 8, 9 means that this strategy is recommended in first intention.

NA means that you have no opinion and/or experience with this strategy in this indication.

| 70 | An adult patient under the age of 65, with no significant organic or psychiatric history, has a depressive unipolar episode treated successively by SSRI then by SNRI or reciprocally in monotherapy of adequate dosage and duration. No response was obtained.  What strategy(s) do you recommend?  *Circle the number corresponding to your choice* |
| --- | --- |

| 70A1 | Switch to a SSRI in monotherapy  (e.g Citalopram) | 0 | 1 | 2 | 3 | 4 | 5 | 6 | 7 | 8 | 9 | NA |
| --- | --- | --- | --- | --- | --- | --- | --- | --- | --- | --- | --- | --- |
| 70A2 | Switch to a SNRI in monotherapy  (e.g Venlafaxine) | 0 | 1 | 2 | 3 | 4 | 5 | 6 | 7 | 8 | 9 | NA |
| 70A3 | Switch to a tricyclic antidepressant in monotherapy  (e.g Clomipramine) | 0 | 1 | 2 | 3 | 4 | 5 | 6 | 7 | 8 | 9 | NA |
| 70A4 | Switch to Mianserine / Mirtazapine in monotherapy | 0 | 1 | 2 | 3 | 4 | 5 | 6 | 7 | 8 | 9 | NA |
| 70A5 | Switch to Tianeptine in monotherapy | 0 | 1 | 2 | 3 | 4 | 5 | 6 | 7 | 8 | 9 | NA |
| 70A6 | Switch to Agomelatine in monotherapy | 0 | 1 | 2 | 3 | 4 | 5 | 6 | 7 | 8 | 9 | NA |
| 70A7 | Switch to MAOI A (Moclobemide) in monotherapy | 0 | 1 | 2 | 3 | 4 | 5 | 6 | 7 | 8 | 9 | NA |
| 70A8 | Switch to a non selectif irreversible MAOI (Iproniazide) in monotherapy | 0 | 1 | 2 | 3 | 4 | 5 | 6 | 7 | 8 | 9 | NA |
| 70A9 | Switch to Bupropion in monotherapy | 0 | 1 | 2 | 3 | 4 | 5 | 6 | 7 | 8 | 9 | NA |
| 70B1 | Adding a SSRI | 0 | 1 | 2 | 3 | 4 | 5 | 6 | 7 | 8 | 9 | NA |
| 70B2 | Adding a SNRI | 0 | 1 | 2 | 3 | 4 | 5 | 6 | 7 | 8 | 9 | NA |
| 70B3 | Adding a tricyclic antidepressant | 0 | 1 | 2 | 3 | 4 | 5 | 6 | 7 | 8 | 9 | NA |
| 70B4 | Adding Mianserine / Mirtazapine | 0 | 1 | 2 | 3 | 4 | 5 | 6 | 7 | 8 | 9 | NA |
| 70B5 | Adding Tianeptine | 0 | 1 | 2 | 3 | 4 | 5 | 6 | 7 | 8 | 9 | NA |
| 70B6 | Adding Agomelatine | 0 | 1 | 2 | 3 | 4 | 5 | 6 | 7 | 8 | 9 | NA |
| 70B7 | Adding MAOI A (Moclobemide) | 0 | 1 | 2 | 3 | 4 | 5 | 6 | 7 | 8 | 9 | NA |
| 70B8 | Adding Bupropion | 0 | 1 | 2 | 3 | 4 | 5 | 6 | 7 | 8 | 9 | NA |

| 70 (Continuated) | An adult patient under the age of 65, with no significant organic or psychiatric history, has a depressive unipolar episode treated successively by SSRI then by SNRI or reciprocally in monotherapy of adequate dosage and duration. No response was obtained.  What strategy(s) do you recommend?  *Circle the number corresponding to your choice* |
| --- | --- |

| 70C1 | Adding lithium | 0 | 1 | 2 | 3 | 4 | 5 | 6 | 7 | 8 | 9 | NA |
| --- | --- | --- | --- | --- | --- | --- | --- | --- | --- | --- | --- | --- |
| 70C2 | Adding thyroid hormones | 0 | 1 | 2 | 3 | 4 | 5 | 6 | 7 | 8 | 9 | NA |
| 70C3 | Adding first-generation antipsychotic (e.g Haloperidol) | 0 | 1 | 2 | 3 | 4 | 5 | 6 | 7 | 8 | 9 | NA |
| 70C4 | Adding second-generation antipsychotic (e.g Risperidone) | 0 | 1 | 2 | 3 | 4 | 5 | 6 | 7 | 8 | 9 | NA |
| 70D1 | Pramipexole in combination | 0 | 1 | 2 | 3 | 4 | 5 | 6 | 7 | 8 | 9 | NA |
| 70D2 | Modafinil in combination | 0 | 1 | 2 | 3 | 4 | 5 | 6 | 7 | 8 | 9 | NA |
| 70D3 | Methylphenidate in combination | 0 | 1 | 2 | 3 | 4 | 5 | 6 | 7 | 8 | 9 | NA |
| 70D4 | Lamotrigine in combination | 0 | 1 | 2 | 3 | 4 | 5 | 6 | 7 | 8 | 9 | NA |
| 70D5 | Valproic acide derivatives (e.g Divalproex sodium) in combination | 0 | 1 | 2 | 3 | 4 | 5 | 6 | 7 | 8 | 9 | NA |
| 70D6 | Carbamazepine in combination | 0 | 1 | 2 | 3 | 4 | 5 | 6 | 7 | 8 | 9 | NA |
| 70D7 | Pregabaline in combination | 0 | 1 | 2 | 3 | 4 | 5 | 6 | 7 | 8 | 9 | NA |
| 70E1 | Structured psychotherpy in monotherapy | 0 | 1 | 2 | 3 | 4 | 5 | 6 | 7 | 8 | 9 | NA |
| 70E2 | Structured psychotherpy in combination | 0 | 1 | 2 | 3 | 4 | 5 | 6 | 7 | 8 | 9 | NA |
| 70F1 | ECT in monotherapy | 0 | 1 | 2 | 3 | 4 | 5 | 6 | 7 | 8 | 9 | NA |
| 70F2 | ECT in combination | 0 | 1 | 2 | 3 | 4 | 5 | 6 | 7 | 8 | 9 | NA |
| 70F3 | rTMS in monotherapy | 0 | 1 | 2 | 3 | 4 | 5 | 6 | 7 | 8 | 9 | NA |
| 70F4 | rTMS in combination | 0 | 1 | 2 | 3 | 4 | 5 | 6 | 7 | 8 | 9 | NA |

**Question 71**

0 means that this strategy is not recommended in first intention.

1, 2, 3 means that this strategy is recommended in third intention.

4, 5, 6 means that this strategy is recommended in second intention.

7, 8, 9 means that this strategy is recommended in first intention.

NA means that you have no opinion and/or experience with this strategy in this indication.

| 71 | An adult patient under the age of 65, with no significant organic or psychiatric history, has a depressive unipolar episode treated successively by SSRI then by Mirtazapine/Mianserine or reciprocally in monotherapy of adequate dosage and duration. No response was obtained.  What strategy(s) do you recommend?  *Circle the number corresponding to your choice* |
| --- | --- |

| 71A1 | Switch to a SSRI in monotherapy  (e.g Citalopram) | 0 | 1 | 2 | 3 | 4 | 5 | 6 | 7 | 8 | 9 | NA |
| --- | --- | --- | --- | --- | --- | --- | --- | --- | --- | --- | --- | --- |
| 71A2 | Switch to a SNRI in monotherapy  (e.g Venlafaxine) | 0 | 1 | 2 | 3 | 4 | 5 | 6 | 7 | 8 | 9 | NA |
| 71A3 | Switch to a tricyclic antidepressant in monotherapy  (e.g Clomipramine) | 0 | 1 | 2 | 3 | 4 | 5 | 6 | 7 | 8 | 9 | NA |
| 71A4 | Switch to Mianserine / Mirtazapine in monotherapy | 0 | 1 | 2 | 3 | 4 | 5 | 6 | 7 | 8 | 9 | NA |
| 71A5 | Switch to Tianeptine in monotherapy | 0 | 1 | 2 | 3 | 4 | 5 | 6 | 7 | 8 | 9 | NA |
| 71A6 | Switch to Agomelatine in monotherapy | 0 | 1 | 2 | 3 | 4 | 5 | 6 | 7 | 8 | 9 | NA |
| 71A7 | Switch to MAOI A (Moclobemide) in monotherapy | 0 | 1 | 2 | 3 | 4 | 5 | 6 | 7 | 8 | 9 | NA |
| 71A8 | Switch to a non selectif irreversible MAOI (Iproniazide) in monotherapy | 0 | 1 | 2 | 3 | 4 | 5 | 6 | 7 | 8 | 9 | NA |
| 71A9 | Switch to Bupropion in monotherapy | 0 | 1 | 2 | 3 | 4 | 5 | 6 | 7 | 8 | 9 | NA |
| 71B1 | Adding a SSRI | 0 | 1 | 2 | 3 | 4 | 5 | 6 | 7 | 8 | 9 | NA |
| 71B2 | Adding a SNRI | 0 | 1 | 2 | 3 | 4 | 5 | 6 | 7 | 8 | 9 | NA |
| 71B3 | Adding a tricyclic antidepressant | 0 | 1 | 2 | 3 | 4 | 5 | 6 | 7 | 8 | 9 | NA |
| 71B4 | Adding Mianserine / Mirtazapine | 0 | 1 | 2 | 3 | 4 | 5 | 6 | 7 | 8 | 9 | NA |
| 71B5 | Adding Tianeptine | 0 | 1 | 2 | 3 | 4 | 5 | 6 | 7 | 8 | 9 | NA |
| 71B6 | Adding Agomelatine | 0 | 1 | 2 | 3 | 4 | 5 | 6 | 7 | 8 | 9 | NA |
| 71B7 | Adding MAOI A (Moclobemide) | 0 | 1 | 2 | 3 | 4 | 5 | 6 | 7 | 8 | 9 | NA |
| 71B8 | Adding Bupropion | 0 | 1 | 2 | 3 | 4 | 5 | 6 | 7 | 8 | 9 | NA |

| 71 (Continuated) | An adult patient under the age of 65, with no significant organic or psychiatric history, has a depressive unipolar episode treated successively by SSRI then by Mirtazapine/Mianserine or reciprocally in monotherapy of adequate dosage and duration. No response was obtained.  What strategy(s) do you recommend?  *Circle the number corresponding to your choice* |
| --- | --- |

| 71C1 | Adding lithium | 0 | 1 | 2 | 3 | 4 | 5 | 6 | 7 | 8 | 9 | NA |
| --- | --- | --- | --- | --- | --- | --- | --- | --- | --- | --- | --- | --- |
| 71C2 | Adding thyroid hormones | 0 | 1 | 2 | 3 | 4 | 5 | 6 | 7 | 8 | 9 | NA |
| 71C3 | Adding first-generation antipsychotic (e.g Haloperidol) | 0 | 1 | 2 | 3 | 4 | 5 | 6 | 7 | 8 | 9 | NA |
| 71C4 | Adding second-generation antipsychotic (e.g Risperidone) | 0 | 1 | 2 | 3 | 4 | 5 | 6 | 7 | 8 | 9 | NA |
| 71D1 | Pramipexole in combination | 0 | 1 | 2 | 3 | 4 | 5 | 6 | 7 | 8 | 9 | NA |
| 71D2 | Modafinil in combination | 0 | 1 | 2 | 3 | 4 | 5 | 6 | 7 | 8 | 9 | NA |
| 71D3 | Methylphenidate in combination | 0 | 1 | 2 | 3 | 4 | 5 | 6 | 7 | 8 | 9 | NA |
| 71D4 | Lamotrigine in combination | 0 | 1 | 2 | 3 | 4 | 5 | 6 | 7 | 8 | 9 | NA |
| 71D5 | Valproic acide derivatives (e.g Divalproex sodium) in combination | 0 | 1 | 2 | 3 | 4 | 5 | 6 | 7 | 8 | 9 | NA |
| 71D6 | Carbamazepine in combination | 0 | 1 | 2 | 3 | 4 | 5 | 6 | 7 | 8 | 9 | NA |
| 71D7 | Pregabaline in combination | 0 | 1 | 2 | 3 | 4 | 5 | 6 | 7 | 8 | 9 | NA |
| 71E1 | Structured psychotherpy in monotherapy | 0 | 1 | 2 | 3 | 4 | 5 | 6 | 7 | 8 | 9 | NA |
| 71E2 | Structured psychotherpy in combination | 0 | 1 | 2 | 3 | 4 | 5 | 6 | 7 | 8 | 9 | NA |
| 71F1 | ECT in monotherapy | 0 | 1 | 2 | 3 | 4 | 5 | 6 | 7 | 8 | 9 | NA |
| 71F2 | ECT in combination | 0 | 1 | 2 | 3 | 4 | 5 | 6 | 7 | 8 | 9 | NA |
| 71F3 | rTMS in monotherapy | 0 | 1 | 2 | 3 | 4 | 5 | 6 | 7 | 8 | 9 | NA |
| 71F4 | rTMS in combination | 0 | 1 | 2 | 3 | 4 | 5 | 6 | 7 | 8 | 9 | NA |

**Question 72**

0 means that this strategy is not recommended in first intention

1, 2, 3 means that this strategy is recommended in third intention

4, 5, 6 means that this strategy is recommended in second intention

7, 8, 9 means that this strategy is recommended in first intention.

NA means that you have no opinion and/or experience with this strategy in this indication.

| 72 | An adult patient under the age of 65, with no significant organic or psychiatric history, has a depressive unipolar episode treated successively by SSRI then by tricyclic antidepressant or reciprocally in monotherapy of adequate dosage and duration. No response was obtained.  What strategy(s) do you recommend?  *Circle the number corresponding to your choice* |
| --- | --- |

| 72A1 | Switch to a SSRI in monotherapy  (e.g Citalopram) | 0 | 1 | 2 | 3 | 4 | 5 | 6 | 7 | 8 | 9 | NA |
| --- | --- | --- | --- | --- | --- | --- | --- | --- | --- | --- | --- | --- |
| 72A2 | Switch to a SNRI in monotherapy  (e.g Venlafaxine) | 0 | 1 | 2 | 3 | 4 | 5 | 6 | 7 | 8 | 9 | NA |
| 72A3 | Switch to a tricyclic antidepressant in monotherapy  (e.g Clomipramine) | 0 | 1 | 2 | 3 | 4 | 5 | 6 | 7 | 8 | 9 | NA |
| 72A4 | Switch to Mianserine / Mirtazapine in monotherapy | 0 | 1 | 2 | 3 | 4 | 5 | 6 | 7 | 8 | 9 | NA |
| 72A5 | Switch to Tianeptine in monotherapy | 0 | 1 | 2 | 3 | 4 | 5 | 6 | 7 | 8 | 9 | NA |
| 72A6 | Switch to Agomelatine in monotherapy | 0 | 1 | 2 | 3 | 4 | 5 | 6 | 7 | 8 | 9 | NA |
| 72A7 | Switch to MAOI A (Moclobemide) in monotherapy | 0 | 1 | 2 | 3 | 4 | 5 | 6 | 7 | 8 | 9 | NA |
| 72A8 | Switch to a non selectif irreversible MAOI (Iproniazide) in monotherapy | 0 | 1 | 2 | 3 | 4 | 5 | 6 | 7 | 8 | 9 | NA |
| 72A9 | Switch to Bupropion in monotherapy | 0 | 1 | 2 | 3 | 4 | 5 | 6 | 7 | 8 | 9 | NA |
| 72B1 | Adding a SSRI | 0 | 1 | 2 | 3 | 4 | 5 | 6 | 7 | 8 | 9 | NA |
| 72B2 | Adding a SNRI | 0 | 1 | 2 | 3 | 4 | 5 | 6 | 7 | 8 | 9 | NA |
| 72B3 | Adding a tricyclic antidepressant | 0 | 1 | 2 | 3 | 4 | 5 | 6 | 7 | 8 | 9 | NA |
| 72B4 | Adding Mianserine / Mirtazapine | 0 | 1 | 2 | 3 | 4 | 5 | 6 | 7 | 8 | 9 | NA |
| 72B5 | Adding Tianeptine | 0 | 1 | 2 | 3 | 4 | 5 | 6 | 7 | 8 | 9 | NA |
| 72B6 | Adding Agomelatine | 0 | 1 | 2 | 3 | 4 | 5 | 6 | 7 | 8 | 9 | NA |
| 72B7 | Adding MAOI A (Moclobemide) | 0 | 1 | 2 | 3 | 4 | 5 | 6 | 7 | 8 | 9 | NA |
| 72B8 | Adding Bupropion | 0 | 1 | 2 | 3 | 4 | 5 | 6 | 7 | 8 | 9 | NA |

| 72 (Continuated) | An adult patient under the age of 65, with no significant organic or psychiatric history, has a depressive unipolar episode treated successively by SSRI then by tricyclic antidepressant or reciprocally in monotherapy of adequate dosage and duration. No response was obtained.  What strategy(s) do you recommend?  *Circle the number corresponding to your choice* |
| --- | --- |

| 72C1 | Adding lithium | 0 | 1 | 2 | 3 | 4 | 5 | 6 | 7 | 8 | 9 | NA |
| --- | --- | --- | --- | --- | --- | --- | --- | --- | --- | --- | --- | --- |
| 72C2 | Adding thyroid hormones | 0 | 1 | 2 | 3 | 4 | 5 | 6 | 7 | 8 | 9 | NA |
| 72C3 | Adding first-generation antipsychotic (e.g Haloperidol) | 0 | 1 | 2 | 3 | 4 | 5 | 6 | 7 | 8 | 9 | NA |
| 72C4 | Adding second-generation antipsychotic (e.g Risperidone) | 0 | 1 | 2 | 3 | 4 | 5 | 6 | 7 | 8 | 9 | NA |
| 72D1 | Pramipexole in combination | 0 | 1 | 2 | 3 | 4 | 5 | 6 | 7 | 8 | 9 | NA |
| 72D2 | Modafinil in combination | 0 | 1 | 2 | 3 | 4 | 5 | 6 | 7 | 8 | 9 | NA |
| 72D3 | Methylphenidate in combination | 0 | 1 | 2 | 3 | 4 | 5 | 6 | 7 | 8 | 9 | NA |
| 72D4 | Lamotrigine in combination | 0 | 1 | 2 | 3 | 4 | 5 | 6 | 7 | 8 | 9 | NA |
| 72D5 | Valproic acide derivatives (e.g Divalproex sodium) in combination | 0 | 1 | 2 | 3 | 4 | 5 | 6 | 7 | 8 | 9 | NA |
| 72D6 | Carbamazepine in combination | 0 | 1 | 2 | 3 | 4 | 5 | 6 | 7 | 8 | 9 | NA |
| 72D7 | Pregabaline in combination | 0 | 1 | 2 | 3 | 4 | 5 | 6 | 7 | 8 | 9 | NA |
| 72E1 | Structured psychotherpy in monotherapy | 0 | 1 | 2 | 3 | 4 | 5 | 6 | 7 | 8 | 9 | NA |
| 72E2 | Structured psychotherpy in combination | 0 | 1 | 2 | 3 | 4 | 5 | 6 | 7 | 8 | 9 | NA |
| 72F1 | ECT in monotherapy | 0 | 1 | 2 | 3 | 4 | 5 | 6 | 7 | 8 | 9 | NA |
| 72F2 | ECT in combination | 0 | 1 | 2 | 3 | 4 | 5 | 6 | 7 | 8 | 9 | NA |
| 72F3 | rTMS in monotherapy | 0 | 1 | 2 | 3 | 4 | 5 | 6 | 7 | 8 | 9 | NA |
| 72F4 | rTMS in combination | 0 | 1 | 2 | 3 | 4 | 5 | 6 | 7 | 8 | 9 | NA |

**Question 73**

0 means that this strategy is not recommended in first intention

1, 2, 3 means that this strategy is recommended in third intention

4, 5, 6 means that this strategy is recommended in second intention

7, 8, 9 means that this strategy is recommended in first intention.

NA means that you have no opinion and/or experience with this strategy in this indication.

| 73 | An adult patient under the age of 65, with no significant organic or psychiatric history, has a depressive unipolar episode treated successively by SNRI then by Mirtazapine/Mianserine or reciprocally in monotherapy of adequate dosage and duration. No response was obtained.  What strategy(s) do you recommend?  *Circle the number corresponding to your choice* |
| --- | --- |

| 73A1 | Switch to a SSRI in monotherapy  (e.g Citalopram) | 0 | 1 | 2 | 3 | 4 | 5 | 6 | 7 | 8 | 9 | NA |
| --- | --- | --- | --- | --- | --- | --- | --- | --- | --- | --- | --- | --- |
| 73A2 | Switch to a SNRI in monotherapy  (e.g Venlafaxine) | 0 | 1 | 2 | 3 | 4 | 5 | 6 | 7 | 8 | 9 | NA |
| 73A3 | Switch to a tricyclic antidepressant in monotherapy  (e.g Clomipramine) | 0 | 1 | 2 | 3 | 4 | 5 | 6 | 7 | 8 | 9 | NA |
| 73A4 | Switch to Mianserine / Mirtazapine in monotherapy | 0 | 1 | 2 | 3 | 4 | 5 | 6 | 7 | 8 | 9 | NA |
| 73A5 | Switch to Tianeptine in monotherapy | 0 | 1 | 2 | 3 | 4 | 5 | 6 | 7 | 8 | 9 | NA |
| 73A6 | Switch to Agomelatine in monotherapy | 0 | 1 | 2 | 3 | 4 | 5 | 6 | 7 | 8 | 9 | NA |
| 73A7 | Switch to MAOI A (Moclobemide) in monotherapy | 0 | 1 | 2 | 3 | 4 | 5 | 6 | 7 | 8 | 9 | NA |
| 73A8 | Switch to a non selectif irreversible MAOI (Iproniazide) in monotherapy | 0 | 1 | 2 | 3 | 4 | 5 | 6 | 7 | 8 | 9 | NA |
| 73A9 | Switch to Bupropion in monotherapy | 0 | 1 | 2 | 3 | 4 | 5 | 6 | 7 | 8 | 9 | NA |
| 73B1 | Adding a SSRI | 0 | 1 | 2 | 3 | 4 | 5 | 6 | 7 | 8 | 9 | NA |
| 73B2 | Adding a SNRI | 0 | 1 | 2 | 3 | 4 | 5 | 6 | 7 | 8 | 9 | NA |
| 73B3 | Adding a tricyclic antidepressant | 0 | 1 | 2 | 3 | 4 | 5 | 6 | 7 | 8 | 9 | NA |
| 73B4 | Adding Mianserine / Mirtazapine | 0 | 1 | 2 | 3 | 4 | 5 | 6 | 7 | 8 | 9 | NA |
| 73B5 | Adding Tianeptine | 0 | 1 | 2 | 3 | 4 | 5 | 6 | 7 | 8 | 9 | NA |
| 73B6 | Adding Agomelatine | 0 | 1 | 2 | 3 | 4 | 5 | 6 | 7 | 8 | 9 | NA |
| 73B7 | Adding MAOI A (Moclobemide) | 0 | 1 | 2 | 3 | 4 | 5 | 6 | 7 | 8 | 9 | NA |
| 73B8 | Adding Bupropion | 0 | 1 | 2 | 3 | 4 | 5 | 6 | 7 | 8 | 9 | NA |

| 73 (Continuated) | An adult patient under the age of 65, with no significant organic or psychiatric history, has a depressive unipolar episode treated successively by SNRI then by Mirtazapine/Mianserine or reciprocally in monotherapy of adequate dosage and duration. No response was obtained.  What strategy(s) do you recommend?  *Circle the number corresponding to your choice* |
| --- | --- |

| 73C1 | Adding lithium | 0 | 1 | 2 | 3 | 4 | 5 | 6 | 7 | 8 | 9 | NA |
| --- | --- | --- | --- | --- | --- | --- | --- | --- | --- | --- | --- | --- |
| 73C2 | Adding thyroid hormones | 0 | 1 | 2 | 3 | 4 | 5 | 6 | 7 | 8 | 9 | NA |
| 73C3 | Adding first-generation antipsychotic (e.g Haloperidol) | 0 | 1 | 2 | 3 | 4 | 5 | 6 | 7 | 8 | 9 | NA |
| 73C4 | Adding second-generation antipsychotic (e.g Risperidone) | 0 | 1 | 2 | 3 | 4 | 5 | 6 | 7 | 8 | 9 | NA |
| 73D1 | Pramipexole in combination | 0 | 1 | 2 | 3 | 4 | 5 | 6 | 7 | 8 | 9 | NA |
| 73D2 | Modafinil in combination | 0 | 1 | 2 | 3 | 4 | 5 | 6 | 7 | 8 | 9 | NA |
| 73D3 | Methylphenidate in combination | 0 | 1 | 2 | 3 | 4 | 5 | 6 | 7 | 8 | 9 | NA |
| 73D4 | Lamotrigine in combination | 0 | 1 | 2 | 3 | 4 | 5 | 6 | 7 | 8 | 9 | NA |
| 73D5 | Valproic acide derivatives (e.g Divalproex sodium) in combination | 0 | 1 | 2 | 3 | 4 | 5 | 6 | 7 | 8 | 9 | NA |
| 73D6 | Carbamazepine in combination | 0 | 1 | 2 | 3 | 4 | 5 | 6 | 7 | 8 | 9 | NA |
| 73D7 | Pregabaline in combination | 0 | 1 | 2 | 3 | 4 | 5 | 6 | 7 | 8 | 9 | NA |
| 73E1 | Structured psychotherpy in monotherapy | 0 | 1 | 2 | 3 | 4 | 5 | 6 | 7 | 8 | 9 | NA |
| 73E2 | Structured psychotherpy in combination | 0 | 1 | 2 | 3 | 4 | 5 | 6 | 7 | 8 | 9 | NA |
| 73F1 | ECT in monotherapy | 0 | 1 | 2 | 3 | 4 | 5 | 6 | 7 | 8 | 9 | NA |
| 73F2 | ECT in combination | 0 | 1 | 2 | 3 | 4 | 5 | 6 | 7 | 8 | 9 | NA |
| 73F3 | rTMS in monotherapy | 0 | 1 | 2 | 3 | 4 | 5 | 6 | 7 | 8 | 9 | NA |
| 73F4 | rTMS in combination | 0 | 1 | 2 | 3 | 4 | 5 | 6 | 7 | 8 | 9 | NA |

**Question 74**

0 means that this strategy is not recommended in first intention

1, 2, 3 means that this strategy is recommended in third intention

4, 5, 6 means that this strategy is recommended in second intention

7, 8, 9 means that this strategy is recommended in first intention.

NA means that you have no opinion and/or experience with this strategy in this indication.

| 74 | An adult patient under the age of 65, with no significant organic or psychiatric history, has a depressive unipolar episode treated successively by SNRI then by tricyclic antidepressant or reciprocally in monotherapy of adequate dosage and duration. No response was obtained.  What strategy(s) do you recommend?  *Circle the number corresponding to your choice* |
| --- | --- |

| 74A1 | Switch to a SSRI in monotherapy  (e.g Citalopram) | 0 | 1 | 2 | 3 | 4 | 5 | 6 | 7 | 8 | 9 | NA |
| --- | --- | --- | --- | --- | --- | --- | --- | --- | --- | --- | --- | --- |
| 74A2 | Switch to a SNRI in monotherapy  (e.g Venlafaxine) | 0 | 1 | 2 | 3 | 4 | 5 | 6 | 7 | 8 | 9 | NA |
| 74A3 | Switch to a tricyclic antidepressant in monotherapy  (e.g Clomipramine) | 0 | 1 | 2 | 3 | 4 | 5 | 6 | 7 | 8 | 9 | NA |
| 74A4 | Switch to Mianserine / Mirtazapine in monotherapy | 0 | 1 | 2 | 3 | 4 | 5 | 6 | 7 | 8 | 9 | NA |
| 74A5 | Switch to Tianeptine in monotherapy | 0 | 1 | 2 | 3 | 4 | 5 | 6 | 7 | 8 | 9 | NA |
| 74A6 | Switch to Agomelatine in monotherapy | 0 | 1 | 2 | 3 | 4 | 5 | 6 | 7 | 8 | 9 | NA |
| 74A7 | Switch to MAOI A (Moclobemide) in monotherapy | 0 | 1 | 2 | 3 | 4 | 5 | 6 | 7 | 8 | 9 | NA |
| 74A8 | Switch to a non selectif irreversible MAOI (Iproniazide) in monotherapy | 0 | 1 | 2 | 3 | 4 | 5 | 6 | 7 | 8 | 9 | NA |
| 74A9 | Switch to Bupropion in monotherapy | 0 | 1 | 2 | 3 | 4 | 5 | 6 | 7 | 8 | 9 | NA |
| 74B1 | Adding a SSRI | 0 | 1 | 2 | 3 | 4 | 5 | 6 | 7 | 8 | 9 | NA |
| 74B2 | Adding a SNRI | 0 | 1 | 2 | 3 | 4 | 5 | 6 | 7 | 8 | 9 | NA |
| 74B3 | Adding a tricyclic antidepressant | 0 | 1 | 2 | 3 | 4 | 5 | 6 | 7 | 8 | 9 | NA |
| 74B4 | Adding Mianserine / Mirtazapine | 0 | 1 | 2 | 3 | 4 | 5 | 6 | 7 | 8 | 9 | NA |
| 74B5 | Adding Tianeptine | 0 | 1 | 2 | 3 | 4 | 5 | 6 | 7 | 8 | 9 | NA |
| 74B6 | Adding Agomelatine | 0 | 1 | 2 | 3 | 4 | 5 | 6 | 7 | 8 | 9 | NA |
| 74B7 | Adding MAOI A (Moclobemide) | 0 | 1 | 2 | 3 | 4 | 5 | 6 | 7 | 8 | 9 | NA |
| 74B8 | Adding Bupropion | 0 | 1 | 2 | 3 | 4 | 5 | 6 | 7 | 8 | 9 | NA |

| 74 (Continuated) | An adult patient under the age of 65, with no significant organic or psychiatric history, has a depressive unipolar episode treated successively by SNRI then by tricyclic antidepressant or reciprocally in monotherapy of adequate dosage and duration. No response was obtained.  What strategy(s) do you recommend?  *Circle the number corresponding to your choice* |
| --- | --- |

| 74C1 | Adding lithium | 0 | 1 | 2 | 3 | 4 | 5 | 6 | 7 | 8 | 9 | NA |
| --- | --- | --- | --- | --- | --- | --- | --- | --- | --- | --- | --- | --- |
| 74C2 | Adding thyroid hormones | 0 | 1 | 2 | 3 | 4 | 5 | 6 | 7 | 8 | 9 | NA |
| 74C3 | Adding first-generation antipsychotic (e.g Haloperidol) | 0 | 1 | 2 | 3 | 4 | 5 | 6 | 7 | 8 | 9 | NA |
| 74C4 | Adding second-generation antipsychotic (e.g Risperidone) | 0 | 1 | 2 | 3 | 4 | 5 | 6 | 7 | 8 | 9 | NA |
| 74D1 | Pramipexole in combination | 0 | 1 | 2 | 3 | 4 | 5 | 6 | 7 | 8 | 9 | NA |
| 74D2 | Modafinil in combination | 0 | 1 | 2 | 3 | 4 | 5 | 6 | 7 | 8 | 9 | NA |
| 74D3 | Methylphenidate in combination | 0 | 1 | 2 | 3 | 4 | 5 | 6 | 7 | 8 | 9 | NA |
| 74D4 | Lamotrigine in combination | 0 | 1 | 2 | 3 | 4 | 5 | 6 | 7 | 8 | 9 | NA |
| 74D5 | Valproic acide derivatives (e.g Divalproex sodium) in combination | 0 | 1 | 2 | 3 | 4 | 5 | 6 | 7 | 8 | 9 | NA |
| 74D6 | Carbamazepine in combination | 0 | 1 | 2 | 3 | 4 | 5 | 6 | 7 | 8 | 9 | NA |
| 74D7 | Pregabaline in combination | 0 | 1 | 2 | 3 | 4 | 5 | 6 | 7 | 8 | 9 | NA |
| 74E1 | Structured psychotherpy in monotherapy | 0 | 1 | 2 | 3 | 4 | 5 | 6 | 7 | 8 | 9 | NA |
| 74E2 | Structured psychotherpy in combination | 0 | 1 | 2 | 3 | 4 | 5 | 6 | 7 | 8 | 9 | NA |
| 74F1 | ECT in monotherapy | 0 | 1 | 2 | 3 | 4 | 5 | 6 | 7 | 8 | 9 | NA |
| 74F2 | ECT in combination | 0 | 1 | 2 | 3 | 4 | 5 | 6 | 7 | 8 | 9 | NA |
| 74F3 | rTMS in monotherapy | 0 | 1 | 2 | 3 | 4 | 5 | 6 | 7 | 8 | 9 | NA |
| 74F4 | rTMS in combination | 0 | 1 | 2 | 3 | 4 | 5 | 6 | 7 | 8 | 9 | NA |

**Question 75**

0 means that this strategy is not recommended in first intention

1, 2, 3 means that this strategy is recommended in third intention

4, 5, 6 means that this strategy is recommended in second intention

7, 8, 9 means that this strategy is recommended in first intention.

NA means that you have no opinion and/or experience with this strategy in this indication.

**b) IN CASE OF non-RESPONSE to the 2^nd^ line treatment**

| 75 | An adult patient under the age of 65, with no significant organic or psychiatric history, has a depressive unipolar episode treated successively by two different SSRI of adequate dosage and duration in monotherapy. No response was obtained.  What strategy(s) do you recommend?  *Circle the number corresponding to your choice* |
| --- | --- |

| 75A1 | Switch to a SSRI in monotherapy  (e.g Citalopram) | 0 | 1 | 2 | 3 | 4 | 5 | 6 | 7 | 8 | 9 | NA |
| --- | --- | --- | --- | --- | --- | --- | --- | --- | --- | --- | --- | --- |
| 75A2 | Switch to a SNRI in monotherapy  (e.g Venlafaxine) | 0 | 1 | 2 | 3 | 4 | 5 | 6 | 7 | 8 | 9 | NA |
| 75A3 | Switch to a tricyclic antidepressant in monotherapy  (e.g Clomipramine) | 0 | 1 | 2 | 3 | 4 | 5 | 6 | 7 | 8 | 9 | NA |
| 75A4 | Switch to Mianserine / Mirtazapine in monotherapy | 0 | 1 | 2 | 3 | 4 | 5 | 6 | 7 | 8 | 9 | NA |
| 75A5 | Switch to Tianeptine in monotherapy | 0 | 1 | 2 | 3 | 4 | 5 | 6 | 7 | 8 | 9 | NA |
| 75A6 | Switch to Agomelatine in monotherapy | 0 | 1 | 2 | 3 | 4 | 5 | 6 | 7 | 8 | 9 | NA |
| 75A7 | Switch to MAOI A (Moclobemide) in monotherapy | 0 | 1 | 2 | 3 | 4 | 5 | 6 | 7 | 8 | 9 | NA |
| 75A8 | Switch to a non selectif irreversible MAOI (Iproniazide) in monotherapy | 0 | 1 | 2 | 3 | 4 | 5 | 6 | 7 | 8 | 9 | NA |
| 75A9 | Switch to Bupropion in monotherapy | 0 | 1 | 2 | 3 | 4 | 5 | 6 | 7 | 8 | 9 | NA |
| 75B1 | Adding a SSRI | 0 | 1 | 2 | 3 | 4 | 5 | 6 | 7 | 8 | 9 | NA |
| 75B2 | Adding a SNRI | 0 | 1 | 2 | 3 | 4 | 5 | 6 | 7 | 8 | 9 | NA |
| 75B3 | Adding a tricyclic antidepressant | 0 | 1 | 2 | 3 | 4 | 5 | 6 | 7 | 8 | 9 | NA |
| 75B4 | Adding Mianserine / Mirtazapine | 0 | 1 | 2 | 3 | 4 | 5 | 6 | 7 | 8 | 9 | NA |
| 75B5 | Adding Tianeptine | 0 | 1 | 2 | 3 | 4 | 5 | 6 | 7 | 8 | 9 | NA |
| 75B6 | Adding Agomelatine | 0 | 1 | 2 | 3 | 4 | 5 | 6 | 7 | 8 | 9 | NA |
| 75B7 | Adding MAOI A (Moclobemide) | 0 | 1 | 2 | 3 | 4 | 5 | 6 | 7 | 8 | 9 | NA |
| 75B8 | Adding Bupropion | 0 | 1 | 2 | 3 | 4 | 5 | 6 | 7 | 8 | 9 | NA |

| 75 (Continuated) | An adult patient under the age of 65, with no significant organic or psychiatric history, has a depressive unipolar episode treated successively by two different SSRI of adequate dosage and duration in monotherapy. No response was obtained.  What strategy(s) do you recommend?  *Circle the number corresponding to your choice* |
| --- | --- |

| 75C1 | Adding lithium | 0 | 1 | 2 | 3 | 4 | 5 | 6 | 7 | 8 | 9 | NA |
| --- | --- | --- | --- | --- | --- | --- | --- | --- | --- | --- | --- | --- |
| 75C2 | Adding thyroid hormones | 0 | 1 | 2 | 3 | 4 | 5 | 6 | 7 | 8 | 9 | NA |
| 75C3 | Adding first-generation antipsychotic (e.g Haloperidol) | 0 | 1 | 2 | 3 | 4 | 5 | 6 | 7 | 8 | 9 | NA |
| 75C4 | Adding second-generation antipsychotic (e.g Risperidone) | 0 | 1 | 2 | 3 | 4 | 5 | 6 | 7 | 8 | 9 | NA |
| 75D1 | Pramipexole in combination | 0 | 1 | 2 | 3 | 4 | 5 | 6 | 7 | 8 | 9 | NA |
| 75D2 | Modafinil in combination | 0 | 1 | 2 | 3 | 4 | 5 | 6 | 7 | 8 | 9 | NA |
| 75D3 | Methylphenidate in combination | 0 | 1 | 2 | 3 | 4 | 5 | 6 | 7 | 8 | 9 | NA |
| 75D4 | Lamotrigine in combination | 0 | 1 | 2 | 3 | 4 | 5 | 6 | 7 | 8 | 9 | NA |
| 75D5 | Valproic acide derivatives (e.g Divalproex sodium) in combination | 0 | 1 | 2 | 3 | 4 | 5 | 6 | 7 | 8 | 9 | NA |
| 75D6 | Carbamazepine in combination | 0 | 1 | 2 | 3 | 4 | 5 | 6 | 7 | 8 | 9 | NA |
| 75D7 | Pregabaline in combination | 0 | 1 | 2 | 3 | 4 | 5 | 6 | 7 | 8 | 9 | NA |
| 75E1 | Structured psychotherpy in monotherapy | 0 | 1 | 2 | 3 | 4 | 5 | 6 | 7 | 8 | 9 | NA |
| 75E2 | Structured psychotherpy in combination | 0 | 1 | 2 | 3 | 4 | 5 | 6 | 7 | 8 | 9 | NA |
| 75F1 | ECT in monotherapy | 0 | 1 | 2 | 3 | 4 | 5 | 6 | 7 | 8 | 9 | NA |
| 75F2 | ECT in combination | 0 | 1 | 2 | 3 | 4 | 5 | 6 | 7 | 8 | 9 | NA |
| 75F3 | rTMS in monotherapy | 0 | 1 | 2 | 3 | 4 | 5 | 6 | 7 | 8 | 9 | NA |
| 75F4 | rTMS in combination | 0 | 1 | 2 | 3 | 4 | 5 | 6 | 7 | 8 | 9 | NA |

**Question 76**

0 means that this strategy is not recommended in first intention.

1, 2, 3 means that this strategy is recommended in third intention.

4, 5, 6 means that this strategy is recommended in second intention.

7, 8, 9 means that this strategy is recommended in first intention.

NA means that you have no opinion and/or experience with this strategy in this indication.

| 76 | An adult patient under the age of 65, with no significant organic or psychiatric history, has a depressive unipolar episode treated successively by SSRI then by SNRI or reciprocally in monotherapy of adequate dosage and duration. No response was obtained.  What strategy(s) do you recommend?  *Circle the number corresponding to your choice* |
| --- | --- |

| 76A1 | Switch to a SSRI in monotherapy  (e.g Citalopram) | 0 | 1 | 2 | 3 | 4 | 5 | 6 | 7 | 8 | 9 | NA |
| --- | --- | --- | --- | --- | --- | --- | --- | --- | --- | --- | --- | --- |
| 76A2 | Switch to a SNRI in monotherapy  (e.g Venlafaxine) | 0 | 1 | 2 | 3 | 4 | 5 | 6 | 7 | 8 | 9 | NA |
| 76A3 | Switch to a tricyclic antidepressant in monotherapy  (e.g Clomipramine) | 0 | 1 | 2 | 3 | 4 | 5 | 6 | 7 | 8 | 9 | NA |
| 76A4 | Switch to Mianserine / Mirtazapine in monotherapy | 0 | 1 | 2 | 3 | 4 | 5 | 6 | 7 | 8 | 9 | NA |
| 76A5 | Switch to Tianeptine in monotherapy | 0 | 1 | 2 | 3 | 4 | 5 | 6 | 7 | 8 | 9 | NA |
| 76A6 | Switch to Agomelatine in monotherapy | 0 | 1 | 2 | 3 | 4 | 5 | 6 | 7 | 8 | 9 | NA |
| 76A7 | Switch to MAOI A (Moclobemide) in monotherapy | 0 | 1 | 2 | 3 | 4 | 5 | 6 | 7 | 8 | 9 | NA |
| 76A8 | Switch to a non selectif irreversible MAOI (Iproniazide) in monotherapy | 0 | 1 | 2 | 3 | 4 | 5 | 6 | 7 | 8 | 9 | NA |
| 76A9 | Switch to Bupropion in monotherapy | 0 | 1 | 2 | 3 | 4 | 5 | 6 | 7 | 8 | 9 | NA |
| 76B1 | Adding a SSRI | 0 | 1 | 2 | 3 | 4 | 5 | 6 | 7 | 8 | 9 | NA |
| 76B2 | Adding a SNRI | 0 | 1 | 2 | 3 | 4 | 5 | 6 | 7 | 8 | 9 | NA |
| 76B3 | Adding a tricyclic antidepressant | 0 | 1 | 2 | 3 | 4 | 5 | 6 | 7 | 8 | 9 | NA |
| 76B4 | Adding Mianserine / Mirtazapine | 0 | 1 | 2 | 3 | 4 | 5 | 6 | 7 | 8 | 9 | NA |
| 76B5 | Adding Tianeptine | 0 | 1 | 2 | 3 | 4 | 5 | 6 | 7 | 8 | 9 | NA |
| 76B6 | Adding Agomelatine | 0 | 1 | 2 | 3 | 4 | 5 | 6 | 7 | 8 | 9 | NA |
| 76B7 | Adding MAOI A (Moclobemide) | 0 | 1 | 2 | 3 | 4 | 5 | 6 | 7 | 8 | 9 | NA |
| 76B8 | Adding Bupropion | 0 | 1 | 2 | 3 | 4 | 5 | 6 | 7 | 8 | 9 | NA |

| 76 (Continuated) | An adult patient under the age of 65, with no significant organic or psychiatric history, has a depressive unipolar episode treated successively by SSRI then by SNRI or reciprocally in monotherapy of adequate dosage and duration. No response was obtained.  What strategy(s) do you recommend?  *Circle the number corresponding to your choice* |
| --- | --- |

| 76C1 | Adding lithium | 0 | 1 | 2 | 3 | 4 | 5 | 6 | 7 | 8 | 9 | NA |
| --- | --- | --- | --- | --- | --- | --- | --- | --- | --- | --- | --- | --- |
| 76C2 | Adding thyroid hormones | 0 | 1 | 2 | 3 | 4 | 5 | 6 | 7 | 8 | 9 | NA |
| 76C3 | Adding first-generation antipsychotic (e.g Haloperidol) | 0 | 1 | 2 | 3 | 4 | 5 | 6 | 7 | 8 | 9 | NA |
| 76C4 | Adding second-generation antipsychotic (e.g Risperidone) | 0 | 1 | 2 | 3 | 4 | 5 | 6 | 7 | 8 | 9 | NA |
| 76D1 | Pramipexole in combination | 0 | 1 | 2 | 3 | 4 | 5 | 6 | 7 | 8 | 9 | NA |
| 76D2 | Modafinil in combination | 0 | 1 | 2 | 3 | 4 | 5 | 6 | 7 | 8 | 9 | NA |
| 76D3 | Methylphenidate in combination | 0 | 1 | 2 | 3 | 4 | 5 | 6 | 7 | 8 | 9 | NA |
| 76D4 | Lamotrigine in combination | 0 | 1 | 2 | 3 | 4 | 5 | 6 | 7 | 8 | 9 | NA |
| 76D5 | Valproic acide derivatives (e.g Divalproex sodium) in combination | 0 | 1 | 2 | 3 | 4 | 5 | 6 | 7 | 8 | 9 | NA |
| 76D6 | Carbamazepine in combination | 0 | 1 | 2 | 3 | 4 | 5 | 6 | 7 | 8 | 9 | NA |
| 76D7 | Pregabaline in combination | 0 | 1 | 2 | 3 | 4 | 5 | 6 | 7 | 8 | 9 | NA |
| 76E1 | Structured psychotherpy in monotherapy | 0 | 1 | 2 | 3 | 4 | 5 | 6 | 7 | 8 | 9 | NA |
| 76E2 | Structured psychotherpy in combination | 0 | 1 | 2 | 3 | 4 | 5 | 6 | 7 | 8 | 9 | NA |
| 76F1 | ECT in monotherapy | 0 | 1 | 2 | 3 | 4 | 5 | 6 | 7 | 8 | 9 | NA |
| 76F2 | ECT in combination | 0 | 1 | 2 | 3 | 4 | 5 | 6 | 7 | 8 | 9 | NA |
| 76F3 | rTMS in monotherapy | 0 | 1 | 2 | 3 | 4 | 5 | 6 | 7 | 8 | 9 | NA |
| 76F4 | rTMS in combination | 0 | 1 | 2 | 3 | 4 | 5 | 6 | 7 | 8 | 9 | NA |

**Question 77**

0 means that this strategy is not recommended in first intention

1, 2, 3 means that this strategy is recommended in third intention

4, 5, 6 means that this strategy is recommended in second intention

7, 8, 9 means that this strategy is recommended in first intention.

NA means that you have no opinion and/or experience with this strategy in this indication.

| 77 | An adult patient under the age of 65, with no significant organic or psychiatric history, has a depressive unipolar episode treated successively by SSRI then by Mianserine/Mirtazapine or reciprocally in monotherapy of adequate dosage and duration. No response was obtained.  What strategy(s) do you recommend?  *Circle the number corresponding to your choice* |
| --- | --- |

| 77A1 | Switch to a SSRI in monotherapy  (e.g Citalopram) | 0 | 1 | 2 | 3 | 4 | 5 | 6 | 7 | 8 | 9 | NA |
| --- | --- | --- | --- | --- | --- | --- | --- | --- | --- | --- | --- | --- |
| 77A2 | Switch to a SNRI in monotherapy  (e.g Venlafaxine) | 0 | 1 | 2 | 3 | 4 | 5 | 6 | 7 | 8 | 9 | NA |
| 77A3 | Switch to a tricyclic antidepressant in monotherapy  (e.g Clomipramine) | 0 | 1 | 2 | 3 | 4 | 5 | 6 | 7 | 8 | 9 | NA |
| 77A4 | Switch to Mianserine / Mirtazapine in monotherapy | 0 | 1 | 2 | 3 | 4 | 5 | 6 | 7 | 8 | 9 | NA |
| 77A5 | Switch to Tianeptine in monotherapy | 0 | 1 | 2 | 3 | 4 | 5 | 6 | 7 | 8 | 9 | NA |
| 77A6 | Switch to Agomelatine in monotherapy | 0 | 1 | 2 | 3 | 4 | 5 | 6 | 7 | 8 | 9 | NA |
| 77A7 | Switch to MAOI A (Moclobemide) in monotherapy | 0 | 1 | 2 | 3 | 4 | 5 | 6 | 7 | 8 | 9 | NA |
| 77A8 | Switch to a non selectif irreversible MAOI (Iproniazide) in monotherapy | 0 | 1 | 2 | 3 | 4 | 5 | 6 | 7 | 8 | 9 | NA |
| 77A9 | Switch to Bupropion in monotherapy | 0 | 1 | 2 | 3 | 4 | 5 | 6 | 7 | 8 | 9 | NA |
| 77B1 | Adding a SSRI | 0 | 1 | 2 | 3 | 4 | 5 | 6 | 7 | 8 | 9 | NA |
| 77B2 | Adding a SNRI | 0 | 1 | 2 | 3 | 4 | 5 | 6 | 7 | 8 | 9 | NA |
| 77B3 | Adding a tricyclic antidepressant | 0 | 1 | 2 | 3 | 4 | 5 | 6 | 7 | 8 | 9 | NA |
| 77B4 | Adding Mianserine / Mirtazapine | 0 | 1 | 2 | 3 | 4 | 5 | 6 | 7 | 8 | 9 | NA |
| 77B5 | Adding Tianeptine | 0 | 1 | 2 | 3 | 4 | 5 | 6 | 7 | 8 | 9 | NA |
| 77B6 | Adding Agomelatine | 0 | 1 | 2 | 3 | 4 | 5 | 6 | 7 | 8 | 9 | NA |
| 77B7 | Adding MAOI A (Moclobemide) | 0 | 1 | 2 | 3 | 4 | 5 | 6 | 7 | 8 | 9 | NA |
| 77B8 | Adding Bupropion | 0 | 1 | 2 | 3 | 4 | 5 | 6 | 7 | 8 | 9 | NA |

| 77 (Continuated) | An adult patient under the age of 65, with no significant organic or psychiatric history, has a depressive unipolar episode treated successively by SSRI then by Mianserine/Mirtazapine or reciprocally in monotherapy of adequate dosage and duration. No response was obtained.  What strategy(s) do you recommend?  What strategy(s) do you recommend?  *Circle the number corresponding to your choice* |
| --- | --- |

| 77C1 | Adding lithium | 0 | 1 | 2 | 3 | 4 | 5 | 6 | 7 | 8 | 9 | NA |
| --- | --- | --- | --- | --- | --- | --- | --- | --- | --- | --- | --- | --- |
| 77C2 | Adding thyroid hormones | 0 | 1 | 2 | 3 | 4 | 5 | 6 | 7 | 8 | 9 | NA |
| 77C3 | Adding first-generation antipsychotic (e.g Haloperidol) | 0 | 1 | 2 | 3 | 4 | 5 | 6 | 7 | 8 | 9 | NA |
| 77C4 | Adding second-generation antipsychotic (e.g Risperidone) | 0 | 1 | 2 | 3 | 4 | 5 | 6 | 7 | 8 | 9 | NA |
| 77D1 | Pramipexole in combination | 0 | 1 | 2 | 3 | 4 | 5 | 6 | 7 | 8 | 9 | NA |
| 77D2 | Modafinil in combination | 0 | 1 | 2 | 3 | 4 | 5 | 6 | 7 | 8 | 9 | NA |
| 77D3 | Methylphenidate in combination | 0 | 1 | 2 | 3 | 4 | 5 | 6 | 7 | 8 | 9 | NA |
| 77D4 | Lamotrigine in combination | 0 | 1 | 2 | 3 | 4 | 5 | 6 | 7 | 8 | 9 | NA |
| 77D5 | Valproic acide derivatives (e.g Divalproex sodium) in combination | 0 | 1 | 2 | 3 | 4 | 5 | 6 | 7 | 8 | 9 | NA |
| 77D6 | Carbamazepine in combination | 0 | 1 | 2 | 3 | 4 | 5 | 6 | 7 | 8 | 9 | NA |
| 77D7 | Pregabaline in combination | 0 | 1 | 2 | 3 | 4 | 5 | 6 | 7 | 8 | 9 | NA |
| 77E1 | Structured psychotherpy in monotherapy | 0 | 1 | 2 | 3 | 4 | 5 | 6 | 7 | 8 | 9 | NA |
| 77E2 | Structured psychotherpy in combination | 0 | 1 | 2 | 3 | 4 | 5 | 6 | 7 | 8 | 9 | NA |
| 77F1 | ECT in monotherapy | 0 | 1 | 2 | 3 | 4 | 5 | 6 | 7 | 8 | 9 | NA |
| 77F2 | ECT in combination | 0 | 1 | 2 | 3 | 4 | 5 | 6 | 7 | 8 | 9 | NA |
| 77F3 | rTMS in monotherapy | 0 | 1 | 2 | 3 | 4 | 5 | 6 | 7 | 8 | 9 | NA |
| 77F4 | rTMS in combination | 0 | 1 | 2 | 3 | 4 | 5 | 6 | 7 | 8 | 9 | NA |

**Question 78**

0 means that this strategy is not recommended in first intention.

1, 2, 3 means that this strategy is recommended in third intention.

4, 5, 6 means that this strategy is recommended in second intention.

7, 8, 9 means that this strategy is recommended in first intention.

NA means that you have no opinion and/or experience with this strategy in this indication.

| 78 | An adult patient under the age of 65, with no significant organic or psychiatric history, has a depressive unipolar episode treated successively by SSRI then by tricyclic antidepressant or reciprocally in monotherapy of adequate dosage and duration. No response was obtained.  What strategy(s) do you recommend?  *Circle the number corresponding to your choice* |
| --- | --- |

| 78A1 | Switch to a SSRI in monotherapy  (e.g Citalopram) | 0 | 1 | 2 | 3 | 4 | 5 | 6 | 7 | 8 | 9 | NA |
| --- | --- | --- | --- | --- | --- | --- | --- | --- | --- | --- | --- | --- |
| 78A2 | Switch to a SNRI in monotherapy  (e.g Venlafaxine) | 0 | 1 | 2 | 3 | 4 | 5 | 6 | 7 | 8 | 9 | NA |
| 78A3 | Switch to a tricyclic antidepressant in monotherapy  (e.g Clomipramine) | 0 | 1 | 2 | 3 | 4 | 5 | 6 | 7 | 8 | 9 | NA |
| 78A4 | Switch to Mianserine / Mirtazapine in monotherapy | 0 | 1 | 2 | 3 | 4 | 5 | 6 | 7 | 8 | 9 | NA |
| 78A5 | Switch to Tianeptine in monotherapy | 0 | 1 | 2 | 3 | 4 | 5 | 6 | 7 | 8 | 9 | NA |
| 78A6 | Switch to Agomelatine in monotherapy | 0 | 1 | 2 | 3 | 4 | 5 | 6 | 7 | 8 | 9 | NA |
| 78A7 | Switch to MAOI A (Moclobemide) in monotherapy | 0 | 1 | 2 | 3 | 4 | 5 | 6 | 7 | 8 | 9 | NA |
| 78A8 | Switch to a non selectif irreversible MAOI (Iproniazide) in monotherapy | 0 | 1 | 2 | 3 | 4 | 5 | 6 | 7 | 8 | 9 | NA |
| 78A9 | Switch to Bupropion in monotherapy | 0 | 1 | 2 | 3 | 4 | 5 | 6 | 7 | 8 | 9 | NA |
| 78B1 | Adding a SSRI | 0 | 1 | 2 | 3 | 4 | 5 | 6 | 7 | 8 | 9 | NA |
| 78B2 | Adding a SNRI | 0 | 1 | 2 | 3 | 4 | 5 | 6 | 7 | 8 | 9 | NA |
| 78B3 | Adding a tricyclic antidepressant | 0 | 1 | 2 | 3 | 4 | 5 | 6 | 7 | 8 | 9 | NA |
| 78B4 | Adding Mianserine / Mirtazapine | 0 | 1 | 2 | 3 | 4 | 5 | 6 | 7 | 8 | 9 | NA |
| 78B5 | Adding Tianeptine | 0 | 1 | 2 | 3 | 4 | 5 | 6 | 7 | 8 | 9 | NA |
| 78B6 | Adding Agomelatine | 0 | 1 | 2 | 3 | 4 | 5 | 6 | 7 | 8 | 9 | NA |
| 78B7 | Adding MAOI A (Moclobemide) | 0 | 1 | 2 | 3 | 4 | 5 | 6 | 7 | 8 | 9 | NA |
| 78B8 | Adding Bupropion | 0 | 1 | 2 | 3 | 4 | 5 | 6 | 7 | 8 | 9 | NA |

| 78 (Continuated) | An adult patient under the age of 65, with no significant organic or psychiatric history, has a depressive unipolar episode treated successively by SSRI then by tricyclic antidepressant or reciprocally in monotherapy of adequate dosage and duration. No response was obtained.  What strategy(s) do you recommend?  *Circle the number corresponding to your choice* |
| --- | --- |

| 78C1 | Adding lithium | 0 | 1 | 2 | 3 | 4 | 5 | 6 | 7 | 8 | 9 | NA |
| --- | --- | --- | --- | --- | --- | --- | --- | --- | --- | --- | --- | --- |
| 78C2 | Adding thyroid hormones | 0 | 1 | 2 | 3 | 4 | 5 | 6 | 7 | 8 | 9 | NA |
| 78C3 | Adding first-generation antipsychotic (e.g Haloperidol) | 0 | 1 | 2 | 3 | 4 | 5 | 6 | 7 | 8 | 9 | NA |
| 78C4 | Adding second-generation antipsychotic (e.g Risperidone) | 0 | 1 | 2 | 3 | 4 | 5 | 6 | 7 | 8 | 9 | NA |
| 78D1 | Pramipexole in combination | 0 | 1 | 2 | 3 | 4 | 5 | 6 | 7 | 8 | 9 | NA |
| 78D2 | Modafinil in combination | 0 | 1 | 2 | 3 | 4 | 5 | 6 | 7 | 8 | 9 | NA |
| 78D3 | Methylphenidate in combination | 0 | 1 | 2 | 3 | 4 | 5 | 6 | 7 | 8 | 9 | NA |
| 78D4 | Lamotrigine in combination | 0 | 1 | 2 | 3 | 4 | 5 | 6 | 7 | 8 | 9 | NA |
| 78D5 | Valproic acide derivatives (e.g Divalproex sodium) in combination | 0 | 1 | 2 | 3 | 4 | 5 | 6 | 7 | 8 | 9 | NA |
| 78D6 | Carbamazepine in combination | 0 | 1 | 2 | 3 | 4 | 5 | 6 | 7 | 8 | 9 | NA |
| 78D7 | Pregabaline in combination | 0 | 1 | 2 | 3 | 4 | 5 | 6 | 7 | 8 | 9 | NA |
| 78E1 | Structured psychotherpy in monotherapy | 0 | 1 | 2 | 3 | 4 | 5 | 6 | 7 | 8 | 9 | NA |
| 78E2 | Structured psychotherpy in combination | 0 | 1 | 2 | 3 | 4 | 5 | 6 | 7 | 8 | 9 | NA |
| 78F1 | ECT in monotherapy | 0 | 1 | 2 | 3 | 4 | 5 | 6 | 7 | 8 | 9 | NA |
| 78F2 | ECT in combination | 0 | 1 | 2 | 3 | 4 | 5 | 6 | 7 | 8 | 9 | NA |
| 78F3 | rTMS in monotherapy | 0 | 1 | 2 | 3 | 4 | 5 | 6 | 7 | 8 | 9 | NA |
| 78F4 | rTMS in combination | 0 | 1 | 2 | 3 | 4 | 5 | 6 | 7 | 8 | 9 | NA |

**Question 79**

0 means that this strategy is not recommended in first intention

1, 2, 3 means that this strategy is recommended in third intention

4, 5, 6 means that this strategy is recommended in second intention

7, 8, 9 means that this strategy is recommended in first intention.

NA means that you have no opinion and/or experience with this strategy in this indication.

| 79 | An adult patient under the age of 65, with no significant organic or psychiatric history, has a depressive unipolar episode treated successively by SNRI then by Mirtazapine/Mianserine or reciprocally in monotherapy of adequate dosage and duration. No response was obtained.  What strategy(s) do you recommend?  *Circle the number corresponding to your choice* |
| --- | --- |

| 79A1 | Switch to a SSRI in monotherapy  (e.g Citalopram) | 0 | 1 | 2 | 3 | 4 | 5 | 6 | 7 | 8 | 9 | NA |
| --- | --- | --- | --- | --- | --- | --- | --- | --- | --- | --- | --- | --- |
| 79A2 | Switch to a SNRI in monotherapy  (e.g Venlafaxine) | 0 | 1 | 2 | 3 | 4 | 5 | 6 | 7 | 8 | 9 | NA |
| 79A3 | Switch to a tricyclic antidepressant in monotherapy  (e.g Clomipramine) | 0 | 1 | 2 | 3 | 4 | 5 | 6 | 7 | 8 | 9 | NA |
| 79A4 | Switch to Mianserine / Mirtazapine in monotherapy | 0 | 1 | 2 | 3 | 4 | 5 | 6 | 7 | 8 | 9 | NA |
| 79A5 | Switch to Tianeptine in monotherapy | 0 | 1 | 2 | 3 | 4 | 5 | 6 | 7 | 8 | 9 | NA |
| 79A6 | Switch to Agomelatine in monotherapy | 0 | 1 | 2 | 3 | 4 | 5 | 6 | 7 | 8 | 9 | NA |
| 79A7 | Switch to MAOI A (Moclobemide) in monotherapy | 0 | 1 | 2 | 3 | 4 | 5 | 6 | 7 | 8 | 9 | NA |
| 79A8 | Switch to a non selectif irreversible MAOI (Iproniazide) in monotherapy | 0 | 1 | 2 | 3 | 4 | 5 | 6 | 7 | 8 | 9 | NA |
| 79A9 | Switch to Bupropion in monotherapy | 0 | 1 | 2 | 3 | 4 | 5 | 6 | 7 | 8 | 9 | NA |
| 79B1 | Adding a SSRI | 0 | 1 | 2 | 3 | 4 | 5 | 6 | 7 | 8 | 9 | NA |
| 79B2 | Adding a SNRI | 0 | 1 | 2 | 3 | 4 | 5 | 6 | 7 | 8 | 9 | NA |
| 79B3 | Adding a tricyclic antidepressant | 0 | 1 | 2 | 3 | 4 | 5 | 6 | 7 | 8 | 9 | NA |
| 79B4 | Adding Mianserine / Mirtazapine | 0 | 1 | 2 | 3 | 4 | 5 | 6 | 7 | 8 | 9 | NA |
| 79B5 | Adding Tianeptine | 0 | 1 | 2 | 3 | 4 | 5 | 6 | 7 | 8 | 9 | NA |
| 79B6 | Adding Agomelatine | 0 | 1 | 2 | 3 | 4 | 5 | 6 | 7 | 8 | 9 | NA |
| 79B7 | Adding MAOI A (Moclobemide) | 0 | 1 | 2 | 3 | 4 | 5 | 6 | 7 | 8 | 9 | NA |
| 79B8 | Adding Bupropion | 0 | 1 | 2 | 3 | 4 | 5 | 6 | 7 | 8 | 9 | NA |

| 79 (Continuated) | An adult patient under the age of 65, with no significant organic or psychiatric history, has a depressive unipolar episode treated successively by SNRI then by Mirtazapine/Mianserine or reciprocally in monotherapy of adequate dosage and duration. No response was obtained.  What strategy(s) do you recommend?  *Circle the number corresponding to your choice* |
| --- | --- |

| 79C1 | Adding lithium | 0 | 1 | 2 | 3 | 4 | 5 | 6 | 7 | 8 | 9 | NA |
| --- | --- | --- | --- | --- | --- | --- | --- | --- | --- | --- | --- | --- |
| 79C2 | Adding thyroid hormones | 0 | 1 | 2 | 3 | 4 | 5 | 6 | 7 | 8 | 9 | NA |
| 79C3 | Adding first-generation antipsychotic (e.g Haloperidol) | 0 | 1 | 2 | 3 | 4 | 5 | 6 | 7 | 8 | 9 | NA |
| 79C4 | Adding second-generation antipsychotic (e.g Risperidone) | 0 | 1 | 2 | 3 | 4 | 5 | 6 | 7 | 8 | 9 | NA |
| 79D1 | Pramipexole in combination | 0 | 1 | 2 | 3 | 4 | 5 | 6 | 7 | 8 | 9 | NA |
| 79D2 | Modafinil in combination | 0 | 1 | 2 | 3 | 4 | 5 | 6 | 7 | 8 | 9 | NA |
| 79D3 | Methylphenidate in combination | 0 | 1 | 2 | 3 | 4 | 5 | 6 | 7 | 8 | 9 | NA |
| 79D4 | Lamotrigine in combination | 0 | 1 | 2 | 3 | 4 | 5 | 6 | 7 | 8 | 9 | NA |
| 79D5 | Valproic acide derivatives (e.g Divalproex sodium) in combination | 0 | 1 | 2 | 3 | 4 | 5 | 6 | 7 | 8 | 9 | NA |
| 79D6 | Carbamazepine in combination | 0 | 1 | 2 | 3 | 4 | 5 | 6 | 7 | 8 | 9 | NA |
| 79D7 | Pregabaline in combination | 0 | 1 | 2 | 3 | 4 | 5 | 6 | 7 | 8 | 9 | NA |
| 79E1 | Structured psychotherpy in monotherapy | 0 | 1 | 2 | 3 | 4 | 5 | 6 | 7 | 8 | 9 | NA |
| 79E2 | Structured psychotherpy in combination | 0 | 1 | 2 | 3 | 4 | 5 | 6 | 7 | 8 | 9 | NA |
| 79F1 | ECT in monotherapy | 0 | 1 | 2 | 3 | 4 | 5 | 6 | 7 | 8 | 9 | NA |
| 79F2 | ECT in combination | 0 | 1 | 2 | 3 | 4 | 5 | 6 | 7 | 8 | 9 | NA |
| 79F3 | rTMS in monotherapy | 0 | 1 | 2 | 3 | 4 | 5 | 6 | 7 | 8 | 9 | NA |
| 79F4 | rTMS in combination | 0 | 1 | 2 | 3 | 4 | 5 | 6 | 7 | 8 | 9 | NA |

**Question 80**

0 means that this strategy is not recommended in first intention.

1, 2, 3 means that this strategy is recommended in third intention.

4, 5, 6 means that this strategy is recommended in second intention.

7, 8, 9 means that this strategy is recommended in first intention.

NA means that you have no opinion and/or experience with this strategy in this indication.

| 80 | An adult patient under the age of 65, with no significant organic or psychiatric history, has a depressive unipolar episode treated successively by SNRI then by tricyclic antidepressant or reciprocally in monotherapy of adequate dosage and duration. No response was obtained.  What strategy(s) do you recommend?  *Circle the number corresponding to your choice* |
| --- | --- |

| 80A1 | Switch to a SSRI in monotherapy  (e.g Citalopram) | 0 | 1 | 2 | 3 | 4 | 5 | 6 | 7 | 8 | 9 | NA |
| --- | --- | --- | --- | --- | --- | --- | --- | --- | --- | --- | --- | --- |
| 80A2 | Switch to a SNRI in monotherapy  (e.g Venlafaxine) | 0 | 1 | 2 | 3 | 4 | 5 | 6 | 7 | 8 | 9 | NA |
| 80A3 | Switch to a tricyclic antidepressant in monotherapy  (e.g Clomipramine) | 0 | 1 | 2 | 3 | 4 | 5 | 6 | 7 | 8 | 9 | NA |
| 80A4 | Switch to Mianserine / Mirtazapine in monotherapy | 0 | 1 | 2 | 3 | 4 | 5 | 6 | 7 | 8 | 9 | NA |
| 80A5 | Switch to Tianeptine in monotherapy | 0 | 1 | 2 | 3 | 4 | 5 | 6 | 7 | 8 | 9 | NA |
| 80A6 | Switch to Agomelatine in monotherapy | 0 | 1 | 2 | 3 | 4 | 5 | 6 | 7 | 8 | 9 | NA |
| 80A7 | Switch to MAOI A (Moclobemide) in monotherapy | 0 | 1 | 2 | 3 | 4 | 5 | 6 | 7 | 8 | 9 | NA |
| 80A8 | Switch to a non selectif irreversible MAOI (Iproniazide) in monotherapy | 0 | 1 | 2 | 3 | 4 | 5 | 6 | 7 | 8 | 9 | NA |
| 80A9 | Switch to Bupropion in monotherapy | 0 | 1 | 2 | 3 | 4 | 5 | 6 | 7 | 8 | 9 | NA |
| 80B1 | Adding a SSRI | 0 | 1 | 2 | 3 | 4 | 5 | 6 | 7 | 8 | 9 | NA |
| 80B2 | Adding a SNRI | 0 | 1 | 2 | 3 | 4 | 5 | 6 | 7 | 8 | 9 | NA |
| 80B3 | Adding a tricyclic antidepressant | 0 | 1 | 2 | 3 | 4 | 5 | 6 | 7 | 8 | 9 | NA |
| 80B4 | Adding Mianserine / Mirtazapine | 0 | 1 | 2 | 3 | 4 | 5 | 6 | 7 | 8 | 9 | NA |
| 80B5 | Adding Tianeptine | 0 | 1 | 2 | 3 | 4 | 5 | 6 | 7 | 8 | 9 | NA |
| 80B6 | Adding Agomelatine | 0 | 1 | 2 | 3 | 4 | 5 | 6 | 7 | 8 | 9 | NA |
| 80B7 | Adding MAOI A (Moclobemide) | 0 | 1 | 2 | 3 | 4 | 5 | 6 | 7 | 8 | 9 | NA |
| 80B8 | Adding Bupropion | 0 | 1 | 2 | 3 | 4 | 5 | 6 | 7 | 8 | 9 | NA |

| 80 (Continuated) | An adult patient under the age of 65, with no significant organic or psychiatric history, has a depressive unipolar episode treated successively by SNRI then by tricyclic antidepressant or reciprocally in monotherapy of adequate dosage and duration. No response was obtained.  What strategy(s) do you recommend?  *Circle the number corresponding to your choice* |
| --- | --- |

| 80C1 | Adding lithium | 0 | 1 | 2 | 3 | 4 | 5 | 6 | 7 | 8 | 9 | NA |
| --- | --- | --- | --- | --- | --- | --- | --- | --- | --- | --- | --- | --- |
| 80C2 | Adding thyroid hormones | 0 | 1 | 2 | 3 | 4 | 5 | 6 | 7 | 8 | 9 | NA |
| 80C3 | Adding first-generation antipsychotic (e.g Haloperidol) | 0 | 1 | 2 | 3 | 4 | 5 | 6 | 7 | 8 | 9 | NA |
| 80C4 | Adding second-generation antipsychotic (e.g Risperidone) | 0 | 1 | 2 | 3 | 4 | 5 | 6 | 7 | 8 | 9 | NA |
| 80D1 | Pramipexole in combination | 0 | 1 | 2 | 3 | 4 | 5 | 6 | 7 | 8 | 9 | NA |
| 80D2 | Modafinil in combination | 0 | 1 | 2 | 3 | 4 | 5 | 6 | 7 | 8 | 9 | NA |
| 80D3 | Methylphenidate in combination | 0 | 1 | 2 | 3 | 4 | 5 | 6 | 7 | 8 | 9 | NA |
| 80D4 | Lamotrigine in combination | 0 | 1 | 2 | 3 | 4 | 5 | 6 | 7 | 8 | 9 | NA |
| 80D5 | Valproic acide derivatives (e.g Divalproex sodium) in combination | 0 | 1 | 2 | 3 | 4 | 5 | 6 | 7 | 8 | 9 | NA |
| 80D6 | Carbamazepine in combination | 0 | 1 | 2 | 3 | 4 | 5 | 6 | 7 | 8 | 9 | NA |
| 80D7 | Pregabaline in combination | 0 | 1 | 2 | 3 | 4 | 5 | 6 | 7 | 8 | 9 | NA |
| 80E1 | Structured psychotherpy in monotherapy | 0 | 1 | 2 | 3 | 4 | 5 | 6 | 7 | 8 | 9 | NA |
| 80E2 | Structured psychotherpy in combination | 0 | 1 | 2 | 3 | 4 | 5 | 6 | 7 | 8 | 9 | NA |
| 80F1 | ECT in monotherapy | 0 | 1 | 2 | 3 | 4 | 5 | 6 | 7 | 8 | 9 | NA |
| 80F2 | ECT in combination | 0 | 1 | 2 | 3 | 4 | 5 | 6 | 7 | 8 | 9 | NA |
| 80F3 | rTMS in monotherapy | 0 | 1 | 2 | 3 | 4 | 5 | 6 | 7 | 8 | 9 | NA |
| 80F4 | rTMS in combination | 0 | 1 | 2 | 3 | 4 | 5 | 6 | 7 | 8 | 9 | NA |

**Question 81**

0 means that this strategy is not recommended in first intention.

1, 2, 3 means that this strategy is recommended in third intention.

4, 5, 6 means that this strategy is recommended in second intention.

7, 8, 9 means that this strategy is recommended in first intention.

NA means that you have no opinion and/or experience with this strategy in this indication.

### D. 4^th^ line Stratégies

| 81 | An adult patient under the age of 65, with no significant organic or psychiatric history, has a depressive unipolar episode treated successively by four antidepressants of different pharmacological classes (SSRI, SNRI, tricyclic antidepressant) in monotherapy of adequate dosage and duration. No response was obtained.  What strategy(s) do you recommend?  *Circle the number corresponding to your choice* |
| --- | --- |

| 81A1 | Switch to a SSRI in monotherapy  (e.g Citalopram) | 0 | 1 | 2 | 3 | 4 | 5 | 6 | 7 | 8 | 9 | NA |
| --- | --- | --- | --- | --- | --- | --- | --- | --- | --- | --- | --- | --- |
| 81A2 | Switch to a SNRI in monotherapy  (e.g Venlafaxine) | 0 | 1 | 2 | 3 | 4 | 5 | 6 | 7 | 8 | 9 | NA |
| 81A3 | Switch to a tricyclic antidepressant in monotherapy  (e.g Clomipramine) | 0 | 1 | 2 | 3 | 4 | 5 | 6 | 7 | 8 | 9 | NA |
| 81A4 | Switch to Mianserine / Mirtazapine in monotherapy | 0 | 1 | 2 | 3 | 4 | 5 | 6 | 7 | 8 | 9 | NA |
| 81A5 | Switch to Tianeptine in monotherapy | 0 | 1 | 2 | 3 | 4 | 5 | 6 | 7 | 8 | 9 | NA |
| 81A6 | Switch to Agomelatine in monotherapy | 0 | 1 | 2 | 3 | 4 | 5 | 6 | 7 | 8 | 9 | NA |
| 81A7 | Switch to MAOI A (Moclobemide) in monotherapy | 0 | 1 | 2 | 3 | 4 | 5 | 6 | 7 | 8 | 9 | NA |
| 81A8 | Switch to a non selectif irreversible MAOI (Iproniazide) in monotherapy | 0 | 1 | 2 | 3 | 4 | 5 | 6 | 7 | 8 | 9 | NA |
| 81A9 | Switch to Bupropion in monotherapy | 0 | 1 | 2 | 3 | 4 | 5 | 6 | 7 | 8 | 9 | NA |
| 81B1 | Adding a SSRI | 0 | 1 | 2 | 3 | 4 | 5 | 6 | 7 | 8 | 9 | NA |
| 81B2 | Adding a SNRI | 0 | 1 | 2 | 3 | 4 | 5 | 6 | 7 | 8 | 9 | NA |
| 81B3 | Adding a tricyclic antidepressant | 0 | 1 | 2 | 3 | 4 | 5 | 6 | 7 | 8 | 9 | NA |
| 81B4 | Adding Mianserine / Mirtazapine | 0 | 1 | 2 | 3 | 4 | 5 | 6 | 7 | 8 | 9 | NA |
| 81B5 | Adding Tianeptine | 0 | 1 | 2 | 3 | 4 | 5 | 6 | 7 | 8 | 9 | NA |

| 81 (Continuated) | An adult patient under the age of 65, with no significant organic or psychiatric history, has a depressive unipolar episode treated successively by four antidepressants of different pharmacological classes (SSRI, SNRI, tricyclic antidepressant) in monotherapy of adequate dosage and duration. No response was obtained.  What strategy(s) do you recommend?  *Circle the number corresponding to your choice* |
| --- | --- |

| 81B6 | Adding Agomelatine | 0 | 1 | 2 | 3 | 4 | 5 | 6 | 7 | 8 | 9 | NA |
| --- | --- | --- | --- | --- | --- | --- | --- | --- | --- | --- | --- | --- |
| 81B7 | Adding MAOI A (Moclobemide) | 0 | 1 | 2 | 3 | 4 | 5 | 6 | 7 | 8 | 9 | NA |
| 81B8 | Adding Bupropion | 0 | 1 | 2 | 3 | 4 | 5 | 6 | 7 | 8 | 9 | NA |
| 81C1 | Adding lithium | 0 | 1 | 2 | 3 | 4 | 5 | 6 | 7 | 8 | 9 | NA |
| 81C2 | Adding thyroid hormones | 0 | 1 | 2 | 3 | 4 | 5 | 6 | 7 | 8 | 9 | NA |
| 81C3 | Adding first-generation antipsychotic (e.g Haloperidol) | 0 | 1 | 2 | 3 | 4 | 5 | 6 | 7 | 8 | 9 | NA |
| 81C4 | Adding second-generation antipsychotic (e.g Risperidone) | 0 | 1 | 2 | 3 | 4 | 5 | 6 | 7 | 8 | 9 | NA |
| 81D1 | Pramipexole in combination | 0 | 1 | 2 | 3 | 4 | 5 | 6 | 7 | 8 | 9 | NA |
| 81D2 | Modafinil in combination | 0 | 1 | 2 | 3 | 4 | 5 | 6 | 7 | 8 | 9 | NA |
| 81D3 | Methylphenidate in combination | 0 | 1 | 2 | 3 | 4 | 5 | 6 | 7 | 8 | 9 | NA |
| 81D4 | Lamotrigine in combination | 0 | 1 | 2 | 3 | 4 | 5 | 6 | 7 | 8 | 9 | NA |
| 81D5 | Valproic acide derivatives (e.g Divalproex sodium) in combination | 0 | 1 | 2 | 3 | 4 | 5 | 6 | 7 | 8 | 9 | NA |
| 81D6 | Carbamazepine in combination | 0 | 1 | 2 | 3 | 4 | 5 | 6 | 7 | 8 | 9 | NA |
| 81D7 | Pregabaline in combination | 0 | 1 | 2 | 3 | 4 | 5 | 6 | 7 | 8 | 9 | NA |
| 81E1 | Structured psychotherpy in monotherapy | 0 | 1 | 2 | 3 | 4 | 5 | 6 | 7 | 8 | 9 | NA |
| 81E2 | Structured psychotherpy in combination | 0 | 1 | 2 | 3 | 4 | 5 | 6 | 7 | 8 | 9 | NA |
| 81F1 | ECT in monotherapy | 0 | 1 | 2 | 3 | 4 | 5 | 6 | 7 | 8 | 9 | NA |
| 81F2 | ECT in combination | 0 | 1 | 2 | 3 | 4 | 5 | 6 | 7 | 8 | 9 | NA |
| 81F3 | rTMS in monotherapy | 0 | 1 | 2 | 3 | 4 | 5 | 6 | 7 | 8 | 9 | NA |
| 81F4 | rTMS in combination | 0 | 1 | 2 | 3 | 4 | 5 | 6 | 7 | 8 | 9 | NA |

**Question 82**

0 means that this strategy is not recommended in first intention.

1, 2, 3 means that this strategy is recommended in third intention.

4, 5, 6 means that this strategy is recommended in second intention.

7, 8, 9 means that this strategy is recommended in first intention.

NA means that you have no opinion and/or experience with this strategy in this indication.

| 82 | An adult patient under the age of 65, with no significant organic or psychiatric history, has a depressive unipolar episode treated successively by four antidepressants of different pharmacological classes (SSRI, SNRI, Mianserine/Mirtazapine) in monotherapy of adequate dosage and duration. No response was obtained.  What strategy(s) do you recommend?  *Circle the number corresponding to your choice* |
| --- | --- |

| 82A1 | Switch to a SSRI in monotherapy  (e.g Citalopram) | 0 | 1 | 2 | 3 | 4 | 5 | 6 | 7 | 8 | 9 | NA |
| --- | --- | --- | --- | --- | --- | --- | --- | --- | --- | --- | --- | --- |
| 82A2 | Switch to a SNRI in monotherapy  (e.g Venlafaxine) | 0 | 1 | 2 | 3 | 4 | 5 | 6 | 7 | 8 | 9 | NA |
| 82A3 | Switch to a tricyclic antidepressant in monotherapy  (e.g Clomipramine) | 0 | 1 | 2 | 3 | 4 | 5 | 6 | 7 | 8 | 9 | NA |
| 82A4 | Switch to Mianserine / Mirtazapine in monotherapy | 0 | 1 | 2 | 3 | 4 | 5 | 6 | 7 | 8 | 9 | NA |
| 82A5 | Switch to Tianeptine in monotherapy | 0 | 1 | 2 | 3 | 4 | 5 | 6 | 7 | 8 | 9 | NA |
| 82A6 | Switch to Agomelatine in monotherapy | 0 | 1 | 2 | 3 | 4 | 5 | 6 | 7 | 8 | 9 | NA |
| 82A7 | Switch to MAOI A (Moclobemide) in monotherapy | 0 | 1 | 2 | 3 | 4 | 5 | 6 | 7 | 8 | 9 | NA |
| 82A8 | Switch to a non selectif irreversible MAOI (Iproniazide) in monotherapy | 0 | 1 | 2 | 3 | 4 | 5 | 6 | 7 | 8 | 9 | NA |
| 82A9 | Switch to Bupropion in monotherapy | 0 | 1 | 2 | 3 | 4 | 5 | 6 | 7 | 8 | 9 | NA |
| 82B1 | Adding a SSRI | 0 | 1 | 2 | 3 | 4 | 5 | 6 | 7 | 8 | 9 | NA |
| 82B2 | Adding a SNRI | 0 | 1 | 2 | 3 | 4 | 5 | 6 | 7 | 8 | 9 | NA |
| 82B3 | Adding a tricyclic antidepressant | 0 | 1 | 2 | 3 | 4 | 5 | 6 | 7 | 8 | 9 | NA |
| 82B4 | Adding Mianserine / Mirtazapine | 0 | 1 | 2 | 3 | 4 | 5 | 6 | 7 | 8 | 9 | NA |
| 82B5 | Adding Tianeptine | 0 | 1 | 2 | 3 | 4 | 5 | 6 | 7 | 8 | 9 | NA |
| 82B6 | Adding Agomelatine | 0 | 1 | 2 | 3 | 4 | 5 | 6 | 7 | 8 | 9 | NA |
| 82B7 | Adding MAOI A (Moclobemide) | 0 | 1 | 2 | 3 | 4 | 5 | 6 | 7 | 8 | 9 | NA |
| 82B8 | Adding Bupropion | 0 | 1 | 2 | 3 | 4 | 5 | 6 | 7 | 8 | 9 | NA |

| 82 (Continuated) | An adult patient under the age of 65, with no significant organic or psychiatric history, has a depressive unipolar episode treated successively by four antidepressants of different pharmacological classes (SSRI, SNRI, Mianserine/Mirtazapine) in monotherapy of adequate dosage and duration. No response was obtained.  What strategy(s) do you recommend?  *Circle the number corresponding to your choice* |
| --- | --- |

| 82C1 | Adding lithium | 0 | 1 | 2 | 3 | 4 | 5 | 6 | 7 | 8 | 9 | NA |
| --- | --- | --- | --- | --- | --- | --- | --- | --- | --- | --- | --- | --- |
| 82C2 | Adding thyroid hormones | 0 | 1 | 2 | 3 | 4 | 5 | 6 | 7 | 8 | 9 | NA |
| 82C3 | Adding first-generation antipsychotic (e.g Haloperidol) | 0 | 1 | 2 | 3 | 4 | 5 | 6 | 7 | 8 | 9 | NA |
| 82C4 | Adding second-generation antipsychotic (e.g Risperidone) | 0 | 1 | 2 | 3 | 4 | 5 | 6 | 7 | 8 | 9 | NA |
| 82D1 | Pramipexole in combination | 0 | 1 | 2 | 3 | 4 | 5 | 6 | 7 | 8 | 9 | NA |
| 82D2 | Modafinil in combination | 0 | 1 | 2 | 3 | 4 | 5 | 6 | 7 | 8 | 9 | NA |
| 82D3 | Methylphenidate in combination | 0 | 1 | 2 | 3 | 4 | 5 | 6 | 7 | 8 | 9 | NA |
| 82D4 | Lamotrigine in combination | 0 | 1 | 2 | 3 | 4 | 5 | 6 | 7 | 8 | 9 | NA |
| 82D5 | Valproic acide derivatives (e.g Divalproex sodium) in combination | 0 | 1 | 2 | 3 | 4 | 5 | 6 | 7 | 8 | 9 | NA |
| 82D6 | Carbamazepine in combination | 0 | 1 | 2 | 3 | 4 | 5 | 6 | 7 | 8 | 9 | NA |
| 82D7 | Pregabaline in combination | 0 | 1 | 2 | 3 | 4 | 5 | 6 | 7 | 8 | 9 | NA |
| 82E1 | Structured psychotherpy in monotherapy | 0 | 1 | 2 | 3 | 4 | 5 | 6 | 7 | 8 | 9 | NA |
| 82E2 | Structured psychotherpy in combination | 0 | 1 | 2 | 3 | 4 | 5 | 6 | 7 | 8 | 9 | NA |
| 82F1 | ECT in monotherapy | 0 | 1 | 2 | 3 | 4 | 5 | 6 | 7 | 8 | 9 | NA |
| 82F2 | ECT in combination | 0 | 1 | 2 | 3 | 4 | 5 | 6 | 7 | 8 | 9 | NA |
| 82F3 | rTMS in monotherapy | 0 | 1 | 2 | 3 | 4 | 5 | 6 | 7 | 8 | 9 | NA |
| 82F4 | rTMS in combination | 0 | 1 | 2 | 3 | 4 | 5 | 6 | 7 | 8 | 9 | NA |

**Question 83**

0 means that this strategy is not recommended in first intention.

1, 2, 3 means that this strategy is recommended in third intention.

4, 5, 6 means that this strategy is recommended in second intention.

7, 8, 9 means that this strategy is recommended in first intention.

NA means that you have no opinion and/or experience with this strategy in this indication.

### E. 5^th^ line STRATEGIES

| 83 | An adult patient under the age of 65, with no significant organic or psychiatric history, has a depressive unipolar episode treated successively by four antidepressants of different pharmacological classes (SSRI, SNRI, Mianserine/Mirtazapine, Tricyclic antidepressant) in monotherapy of adequate dosage and duration. No response was obtained.  What strategy(s) do you recommend?  *Circle the number corresponding to your choice* |
| --- | --- |

| 83A1 | Switch to a SSRI in monotherapy  (e.g Citalopram) | 0 | 1 | 2 | 3 | 4 | 5 | 6 | 7 | 8 | 9 | NA |
| --- | --- | --- | --- | --- | --- | --- | --- | --- | --- | --- | --- | --- |
| 83A2 | Switch to a SNRI in monotherapy  (e.g Venlafaxine) | 0 | 1 | 2 | 3 | 4 | 5 | 6 | 7 | 8 | 9 | NA |
| 83A3 | Switch to a tricyclic antidepressant in monotherapy  (e.g Clomipramine) | 0 | 1 | 2 | 3 | 4 | 5 | 6 | 7 | 8 | 9 | NA |
| 83A4 | Switch to Mianserine / Mirtazapine in monotherapy | 0 | 1 | 2 | 3 | 4 | 5 | 6 | 7 | 8 | 9 | NA |
| 83A5 | Switch to Tianeptine in monotherapy | 0 | 1 | 2 | 3 | 4 | 5 | 6 | 7 | 8 | 9 | NA |
| 83A6 | Switch to Agomelatine in monotherapy | 0 | 1 | 2 | 3 | 4 | 5 | 6 | 7 | 8 | 9 | NA |
| 83A7 | Switch to MAOI A (Moclobemide) in monotherapy | 0 | 1 | 2 | 3 | 4 | 5 | 6 | 7 | 8 | 9 | NA |
| 83A8 | Switch to a non selectif irreversible MAOI (Iproniazide) in monotherapy | 0 | 1 | 2 | 3 | 4 | 5 | 6 | 7 | 8 | 9 | NA |
| 83A9 | Switch to Bupropion in monotherapy | 0 | 1 | 2 | 3 | 4 | 5 | 6 | 7 | 8 | 9 | NA |
| 83B1 | Adding a SSRI | 0 | 1 | 2 | 3 | 4 | 5 | 6 | 7 | 8 | 9 | NA |
| 83B2 | Adding a SNRI | 0 | 1 | 2 | 3 | 4 | 5 | 6 | 7 | 8 | 9 | NA |
| 83B3 | Adding a tricyclic antidepressant | 0 | 1 | 2 | 3 | 4 | 5 | 6 | 7 | 8 | 9 | NA |
| 83B4 | Adding Mianserine / Mirtazapine | 0 | 1 | 2 | 3 | 4 | 5 | 6 | 7 | 8 | 9 | NA |
| 83B5 | Adding Tianeptine | 0 | 1 | 2 | 3 | 4 | 5 | 6 | 7 | 8 | 9 | NA |

| 83 (Continuated) | An adult patient under the age of 65, with no significant organic or psychiatric history, has a depressive unipolar episode treated successively by four antidepressants of different pharmacological classes (SSRI, SNRI, Mianserine/Mirtazapine, Tricyclic antidepressant) in monotherapy of adequate dosage and duration. No response was obtained.  What strategy(s) do you recommend?  *Circle the number corresponding to your choice* |
| --- | --- |

| 83B6 | Adding Agomelatine | 0 | 1 | 2 | 3 | 4 | 5 | 6 | 7 | 8 | 9 | NA |
| --- | --- | --- | --- | --- | --- | --- | --- | --- | --- | --- | --- | --- |
| 83B7 | Adding MAOI A (Moclobemide) | 0 | 1 | 2 | 3 | 4 | 5 | 6 | 7 | 8 | 9 | NA |
| 83B8 | Adding Bupropion | 0 | 1 | 2 | 3 | 4 | 5 | 6 | 7 | 8 | 9 | NA |
| 83C1 | Adding lithium | 0 | 1 | 2 | 3 | 4 | 5 | 6 | 7 | 8 | 9 | NA |
| 83C2 | Adding thyroid hormones | 0 | 1 | 2 | 3 | 4 | 5 | 6 | 7 | 8 | 9 | NA |
| 83C3 | Adding first-generation antipsychotic (e.g Haloperidol) | 0 | 1 | 2 | 3 | 4 | 5 | 6 | 7 | 8 | 9 | NA |
| 83C4 | Adding second-generation antipsychotic (e.g Risperidone) | 0 | 1 | 2 | 3 | 4 | 5 | 6 | 7 | 8 | 9 | NA |
| 83D1 | Pramipexole in combination | 0 | 1 | 2 | 3 | 4 | 5 | 6 | 7 | 8 | 9 | NA |
| 83D2 | Modafinil in combination | 0 | 1 | 2 | 3 | 4 | 5 | 6 | 7 | 8 | 9 | NA |
| 83D3 | Methylphenidate in combination | 0 | 1 | 2 | 3 | 4 | 5 | 6 | 7 | 8 | 9 | NA |
| 83D4 | Lamotrigine in combination | 0 | 1 | 2 | 3 | 4 | 5 | 6 | 7 | 8 | 9 | NA |
| 83D5 | Valproic acide derivatives (e.g Divalproex sodium) in combination | 0 | 1 | 2 | 3 | 4 | 5 | 6 | 7 | 8 | 9 | NA |
| 83D6 | Carbamazepine in combination | 0 | 1 | 2 | 3 | 4 | 5 | 6 | 7 | 8 | 9 | NA |
| 83D7 | Pregabaline in combination | 0 | 1 | 2 | 3 | 4 | 5 | 6 | 7 | 8 | 9 | NA |
| 83E1 | Structured psychotherpy in monotherapy | 0 | 1 | 2 | 3 | 4 | 5 | 6 | 7 | 8 | 9 | NA |
| 83E2 | Structured psychotherpy in combination | 0 | 1 | 2 | 3 | 4 | 5 | 6 | 7 | 8 | 9 | NA |
| 83F1 | ECT in monotherapy | 0 | 1 | 2 | 3 | 4 | 5 | 6 | 7 | 8 | 9 | NA |
| 83F2 | ECT in combination | 0 | 1 | 2 | 3 | 4 | 5 | 6 | 7 | 8 | 9 | NA |
| 83F3 | rTMS in monotherapy | 0 | 1 | 2 | 3 | 4 | 5 | 6 | 7 | 8 | 9 | NA |
| 83F4 | rTMS in combination | 0 | 1 | 2 | 3 | 4 | 5 | 6 | 7 | 8 | 9 | NA |

**Question 84**

0 means that this strategy is not recommended in first intention.

1, 2, 3 means that this strategy is recommended in third intention.

4, 5, 6 means that this strategy is recommended in second intention.

7, 8, 9 means that this strategy is recommended in first intention.

NA means that you have no opinion and/or experience with this strategy in this indication.

### F. Strategies in ultra-resistanT DEPRESSION (FROM THE 6^TH^ LINE)

| 84 | An adult patient under the age of 65, with no significant organic or psychiatric history, has a depressive unipolar episode treated successively by several antidepressants of different pharmacological classes, in monotherapy or in combination. No response was obtained.  What strategy(s) do you recommend?  *Circle the number corresponding to your choice* |
| --- | --- |

| 84A1 | Switch to a SSRI in monotherapy  (e.g Citalopram) | 0 | 1 | 2 | 3 | 4 | 5 | 6 | 7 | 8 | 9 | NA |
| --- | --- | --- | --- | --- | --- | --- | --- | --- | --- | --- | --- | --- |
| 84A2 | Switch to a SNRI in monotherapy  (e.g Venlafaxine) | 0 | 1 | 2 | 3 | 4 | 5 | 6 | 7 | 8 | 9 | NA |
| 84A3 | Switch to a tricyclic antidepressant in monotherapy  (e.g Clomipramine) | 0 | 1 | 2 | 3 | 4 | 5 | 6 | 7 | 8 | 9 | NA |
| 84A4 | Switch to Mianserine / Mirtazapine in monotherapy | 0 | 1 | 2 | 3 | 4 | 5 | 6 | 7 | 8 | 9 | NA |
| 84A5 | Switch to Tianeptine in monotherapy | 0 | 1 | 2 | 3 | 4 | 5 | 6 | 7 | 8 | 9 | NA |
| 84A6 | Switch to Agomelatine in monotherapy | 0 | 1 | 2 | 3 | 4 | 5 | 6 | 7 | 8 | 9 | NA |
| 84A7 | Switch to MAOI A (Moclobemide) in monotherapy | 0 | 1 | 2 | 3 | 4 | 5 | 6 | 7 | 8 | 9 | NA |
| 84A8 | Switch to a non selectif irreversible MAOI (Iproniazide) in monotherapy | 0 | 1 | 2 | 3 | 4 | 5 | 6 | 7 | 8 | 9 | NA |
| 84A9 | Switch to Bupropion in monotherapy | 0 | 1 | 2 | 3 | 4 | 5 | 6 | 7 | 8 | 9 | NA |
| 84B1 | Adding a SSRI | 0 | 1 | 2 | 3 | 4 | 5 | 6 | 7 | 8 | 9 | NA |
| 84B2 | Adding a SNRI | 0 | 1 | 2 | 3 | 4 | 5 | 6 | 7 | 8 | 9 | NA |
| 84B3 | Adding a tricyclic antidepressant | 0 | 1 | 2 | 3 | 4 | 5 | 6 | 7 | 8 | 9 | NA |
| 84B4 | Adding Mianserine / Mirtazapine | 0 | 1 | 2 | 3 | 4 | 5 | 6 | 7 | 8 | 9 | NA |
| 84B5 | Adding Tianeptine | 0 | 1 | 2 | 3 | 4 | 5 | 6 | 7 | 8 | 9 | NA |
| 84B6 | Adding Agomelatine |  |  |  |  |  |  |  |  |  |  |  |
| 84B7 | Adding MAOI A (Moclobemide) |  |  |  |  |  |  |  |  |  |  |  |
| 84B8 | Adding Bupropion |  |  |  |  |  |  |  |  |  |  |  |

| 84 (Continuated) | An adult patient under the age of 65, with no significant organic or psychiatric history, has a depressive unipolar episode treated successively by several antidepressants of different pharmacological classes, in monotherapy or in combination. No response was obtained.  What strategy(s) do you recommend?  *Circle the number corresponding to your choice* |
| --- | --- |

| 84C1 | Adding lithium | 0 | 1 | 2 | 3 | 4 | 5 | 6 | 7 | 8 | 9 | NA |
| --- | --- | --- | --- | --- | --- | --- | --- | --- | --- | --- | --- | --- |
| 84C2 | Adding thyroid hormones | 0 | 1 | 2 | 3 | 4 | 5 | 6 | 7 | 8 | 9 | NA |
| 84C3 | Adding first-generation antipsychotic (e.g Haloperidol) | 0 | 1 | 2 | 3 | 4 | 5 | 6 | 7 | 8 | 9 | NA |
| 84C4 | Adding second-generation antipsychotic (e.g Risperidone) | 0 | 1 | 2 | 3 | 4 | 5 | 6 | 7 | 8 | 9 | NA |
| 84D1 | Pramipexole in combination | 0 | 1 | 2 | 3 | 4 | 5 | 6 | 7 | 8 | 9 | NA |
| 84D2 | Modafinil in combination | 0 | 1 | 2 | 3 | 4 | 5 | 6 | 7 | 8 | 9 | NA |
| 84D3 | Methylphenidate in combination | 0 | 1 | 2 | 3 | 4 | 5 | 6 | 7 | 8 | 9 | NA |
| 84D4 | Lamotrigine in combination | 0 | 1 | 2 | 3 | 4 | 5 | 6 | 7 | 8 | 9 | NA |
| 84D5 | Valproic acide derivatives (e.g Divalproex sodium) in combination | 0 | 1 | 2 | 3 | 4 | 5 | 6 | 7 | 8 | 9 | NA |
| 84D6 | Carbamazepine in combination | 0 | 1 | 2 | 3 | 4 | 5 | 6 | 7 | 8 | 9 | NA |
| 84D7 | Pregabaline in combination | 0 | 1 | 2 | 3 | 4 | 5 | 6 | 7 | 8 | 9 | NA |
| 84E1 | Structured psychotherpy in monotherapy | 0 | 1 | 2 | 3 | 4 | 5 | 6 | 7 | 8 | 9 | NA |
| 84E2 | Structured psychotherpy in combination | 0 | 1 | 2 | 3 | 4 | 5 | 6 | 7 | 8 | 9 | NA |
| 84F1 | ECT in monotherapy | 0 | 1 | 2 | 3 | 4 | 5 | 6 | 7 | 8 | 9 | NA |
| 84F2 | ECT in combination | 0 | 1 | 2 | 3 | 4 | 5 | 6 | 7 | 8 | 9 | NA |
| 84F3 | rTMS in monotherapy | 0 | 1 | 2 | 3 | 4 | 5 | 6 | 7 | 8 | 9 | NA |
| 84F4 | rTMS in combination | 0 | 1 | 2 | 3 | 4 | 5 | 6 | 7 | 8 | 9 | NA |

**Question 85**

0 means that this strategy is not recommended in first intention.

1, 2, 3 means that this strategy is recommended in third intention.

4, 5, 6 means that this strategy is recommended in second intention.

7, 8, 9 means that this strategy is recommended in first intention.

NA means that you have no opinion and/or experience with this strategy in this indication.

| 85 | An adult patient under the age of 65, with no significant organic or psychiatric history, has a depressive unipolar episode treated successively by several antidepressants of different pharmacological classes, in monotherapy, in combination or in potentialization by lithium. No response was obtained.  What strategy(s) do you recommend?  *Circle the number corresponding to your choice* |
| --- | --- |

| 85A1 | Switch to a SSRI in monotherapy  (e.g Citalopram) | 0 | 1 | 2 | 3 | 4 | 5 | 6 | 7 | 8 | 9 | NA |
| --- | --- | --- | --- | --- | --- | --- | --- | --- | --- | --- | --- | --- |
| 85A2 | Switch to a SNRI in monotherapy  (e.g Venlafaxine) | 0 | 1 | 2 | 3 | 4 | 5 | 6 | 7 | 8 | 9 | NA |
| 85A3 | Switch to a tricyclic antidepressant in monotherapy  (e.g Clomipramine) | 0 | 1 | 2 | 3 | 4 | 5 | 6 | 7 | 8 | 9 | NA |
| 85A4 | Switch to Mianserine / Mirtazapine in monotherapy | 0 | 1 | 2 | 3 | 4 | 5 | 6 | 7 | 8 | 9 | NA |
| 85A5 | Switch to Tianeptine in monotherapy | 0 | 1 | 2 | 3 | 4 | 5 | 6 | 7 | 8 | 9 | NA |
| 85A6 | Switch to Agomelatine in monotherapy | 0 | 1 | 2 | 3 | 4 | 5 | 6 | 7 | 8 | 9 | NA |
| 85A7 | Switch to MAOI A (Moclobemide) in monotherapy | 0 | 1 | 2 | 3 | 4 | 5 | 6 | 7 | 8 | 9 | NA |
| 85A8 | Switch to a non selectif irreversible MAOI (Iproniazide) in monotherapy | 0 | 1 | 2 | 3 | 4 | 5 | 6 | 7 | 8 | 9 | NA |
| 85A9 | Switch to Bupropion in monotherapy | 0 | 1 | 2 | 3 | 4 | 5 | 6 | 7 | 8 | 9 | NA |
| 85B1 | Adding a SSRI | 0 | 1 | 2 | 3 | 4 | 5 | 6 | 7 | 8 | 9 | NA |
| 85B2 | Adding a SNRI | 0 | 1 | 2 | 3 | 4 | 5 | 6 | 7 | 8 | 9 | NA |
| 85B3 | Adding a tricyclic antidepressant | 0 | 1 | 2 | 3 | 4 | 5 | 6 | 7 | 8 | 9 | NA |
| 85B4 | Adding Mianserine / Mirtazapine | 0 | 1 | 2 | 3 | 4 | 5 | 6 | 7 | 8 | 9 | NA |
| 85B5 | Adding Tianeptine | 0 | 1 | 2 | 3 | 4 | 5 | 6 | 7 | 8 | 9 | NA |
| 85B6 | Adding Agomelatine | 0 | 1 | 2 | 3 | 4 | 5 | 6 | 7 | 8 | 9 | NA |
| 85B7 | Adding MAOI A (Moclobemide) | 0 | 1 | 2 | 3 | 4 | 5 | 6 | 7 | 8 | 9 | NA |
| 85B8 | Adding Bupropion | 0 | 1 | 2 | 3 | 4 | 5 | 6 | 7 | 8 | 9 | NA |

| 85 (Continuated) | An adult patient under the age of 65, with no significant organic or psychiatric history, has a depressive unipolar episode treated successively by several antidepressants of different pharmacological classes, in monotherapy, in combination or in potentialization by lithium. No response was obtained.  What strategy(s) do you recommend?  *Circle the number corresponding to your choice* |
| --- | --- |

| 85C1 | Adding lithium | 0 | 1 | 2 | 3 | 4 | 5 | 6 | 7 | 8 | 9 | NA |
| --- | --- | --- | --- | --- | --- | --- | --- | --- | --- | --- | --- | --- |
| 85C2 | Adding thyroid hormones | 0 | 1 | 2 | 3 | 4 | 5 | 6 | 7 | 8 | 9 | NA |
| 85C3 | Adding first-generation antipsychotic (e.g Haloperidol) | 0 | 1 | 2 | 3 | 4 | 5 | 6 | 7 | 8 | 9 | NA |
| 85C4 | Adding second-generation antipsychotic (e.g Risperidone) | 0 | 1 | 2 | 3 | 4 | 5 | 6 | 7 | 8 | 9 | NA |
| 85D1 | Pramipexole in combination | 0 | 1 | 2 | 3 | 4 | 5 | 6 | 7 | 8 | 9 | NA |
| 85D2 | Modafinil in combination | 0 | 1 | 2 | 3 | 4 | 5 | 6 | 7 | 8 | 9 | NA |
| 85D3 | Methylphenidate in combination | 0 | 1 | 2 | 3 | 4 | 5 | 6 | 7 | 8 | 9 | NA |
| 85D4 | Lamotrigine in combination | 0 | 1 | 2 | 3 | 4 | 5 | 6 | 7 | 8 | 9 | NA |
| 85D5 | Valproic acide derivatives (e.g Divalproex sodium) in combination | 0 | 1 | 2 | 3 | 4 | 5 | 6 | 7 | 8 | 9 | NA |
| 85D6 | Carbamazepine in combination | 0 | 1 | 2 | 3 | 4 | 5 | 6 | 7 | 8 | 9 | NA |
| 85D7 | Pregabaline in combination | 0 | 1 | 2 | 3 | 4 | 5 | 6 | 7 | 8 | 9 | NA |
| 85E1 | Structured psychotherpy in monotherapy | 0 | 1 | 2 | 3 | 4 | 5 | 6 | 7 | 8 | 9 | NA |
| 85E2 | Structured psychotherpy in combination | 0 | 1 | 2 | 3 | 4 | 5 | 6 | 7 | 8 | 9 | NA |
| 85F1 | ECT in monotherapy | 0 | 1 | 2 | 3 | 4 | 5 | 6 | 7 | 8 | 9 | NA |
| 85F2 | ECT in combination | 0 | 1 | 2 | 3 | 4 | 5 | 6 | 7 | 8 | 9 | NA |
| 85F3 | rTMS in monotherapy | 0 | 1 | 2 | 3 | 4 | 5 | 6 | 7 | 8 | 9 | NA |
| 85F4 | rTMS in combination | 0 | 1 | 2 | 3 | 4 | 5 | 6 | 7 | 8 | 9 | NA |

**Question 86**

0 means that this strategy is not recommended in first intention.

1, 2, 3 means that this strategy is recommended in third intention.

4, 5, 6 means that this strategy is recommended in second intention.

7, 8, 9 means that this strategy is recommended in first intention.

NA means that you have no opinion and/or experience with this strategy in this indication.

| 86 | An adult patient under the age of 65, with no significant organic or psychiatric history, has a depressive unipolar episode treated successively by several antidepressants of different pharmacological classes, in monotherapy, in combination or in potentialization by thyroid hormons. No response was obtained.  What strategy(s) do you recommend?  *Circle the number corresponding to your choice* |
| --- | --- |

| 86A1 | Switch to a SSRI in monotherapy  (e.g Citalopram) | 0 | 1 | 2 | 3 | 4 | 5 | 6 | 7 | 8 | 9 | NA |
| --- | --- | --- | --- | --- | --- | --- | --- | --- | --- | --- | --- | --- |
| 86A2 | Switch to a SNRI in monotherapy  (e.g Venlafaxine) | 0 | 1 | 2 | 3 | 4 | 5 | 6 | 7 | 8 | 9 | NA |
| 86A3 | Switch to a tricyclic antidepressant in monotherapy  (e.g Clomipramine) | 0 | 1 | 2 | 3 | 4 | 5 | 6 | 7 | 8 | 9 | NA |
| 86A4 | Switch to Mianserine / Mirtazapine in monotherapy | 0 | 1 | 2 | 3 | 4 | 5 | 6 | 7 | 8 | 9 | NA |
| 86A5 | Switch to Tianeptine in monotherapy | 0 | 1 | 2 | 3 | 4 | 5 | 6 | 7 | 8 | 9 | NA |
| 86A6 | Switch to Agomelatine in monotherapy | 0 | 1 | 2 | 3 | 4 | 5 | 6 | 7 | 8 | 9 | NA |
| 86A7 | Switch to MAOI A (Moclobemide) in monotherapy | 0 | 1 | 2 | 3 | 4 | 5 | 6 | 7 | 8 | 9 | NA |
| 86A8 | Switch to a non selectif irreversible MAOI (Iproniazide) in monotherapy | 0 | 1 | 2 | 3 | 4 | 5 | 6 | 7 | 8 | 9 | NA |
| 86A9 | Switch to Bupropion in monotherapy | 0 | 1 | 2 | 3 | 4 | 5 | 6 | 7 | 8 | 9 | NA |
| 86B1 | Adding a SSRI | 0 | 1 | 2 | 3 | 4 | 5 | 6 | 7 | 8 | 9 | NA |
| 86B2 | Adding a SNRI | 0 | 1 | 2 | 3 | 4 | 5 | 6 | 7 | 8 | 9 | NA |
| 86B3 | Adding a tricyclic antidepressant | 0 | 1 | 2 | 3 | 4 | 5 | 6 | 7 | 8 | 9 | NA |
| 86B4 | Adding Mianserine / Mirtazapine | 0 | 1 | 2 | 3 | 4 | 5 | 6 | 7 | 8 | 9 | NA |
| 86B5 | Adding Tianeptine | 0 | 1 | 2 | 3 | 4 | 5 | 6 | 7 | 8 | 9 | NA |
| 86B6 | Adding Agomelatine | 0 | 1 | 2 | 3 | 4 | 5 | 6 | 7 | 8 | 9 | NA |
| 86B7 | Adding MAOI A (Moclobemide) | 0 | 1 | 2 | 3 | 4 | 5 | 6 | 7 | 8 | 9 | NA |
| 86B8 | Adding Bupropion | 0 | 1 | 2 | 3 | 4 | 5 | 6 | 7 | 8 | 9 | NA |

⮱

| 86 (Continuated) | An adult patient under the age of 65, with no significant organic or psychiatric history, has a depressive unipolar episode treated successively by several antidepressants of different pharmacological classes, in monotherapy, in combination or in potentialization by thyroid hormons. No response was obtained.  What strategy(s) do you recommend?  *Circle the number corresponding to your choice* |
| --- | --- |

| 86C1 | Adding lithium | 0 | 1 | 2 | 3 | 4 | 5 | 6 | 7 | 8 | 9 | NA |
| --- | --- | --- | --- | --- | --- | --- | --- | --- | --- | --- | --- | --- |
| 86C2 | Adding thyroid hormones | 0 | 1 | 2 | 3 | 4 | 5 | 6 | 7 | 8 | 9 | NA |
| 86C3 | Adding first-generation antipsychotic (e.g Haloperidol) | 0 | 1 | 2 | 3 | 4 | 5 | 6 | 7 | 8 | 9 | NA |
| 86C4 | Adding second-generation antipsychotic (e.g Risperidone) | 0 | 1 | 2 | 3 | 4 | 5 | 6 | 7 | 8 | 9 | NA |
| 86D1 | Pramipexole in combination | 0 | 1 | 2 | 3 | 4 | 5 | 6 | 7 | 8 | 9 | NA |
| 86D2 | Modafinil in combination | 0 | 1 | 2 | 3 | 4 | 5 | 6 | 7 | 8 | 9 | NA |
| 86D3 | Methylphenidate in combination | 0 | 1 | 2 | 3 | 4 | 5 | 6 | 7 | 8 | 9 | NA |
| 86D4 | Lamotrigine in combination | 0 | 1 | 2 | 3 | 4 | 5 | 6 | 7 | 8 | 9 | NA |
| 86D5 | Valproic acide derivatives (e.g Divalproex sodium) in combination | 0 | 1 | 2 | 3 | 4 | 5 | 6 | 7 | 8 | 9 | NA |
| 86D6 | Carbamazepine in combination | 0 | 1 | 2 | 3 | 4 | 5 | 6 | 7 | 8 | 9 | NA |
| 86D7 | Pregabaline in combination | 0 | 1 | 2 | 3 | 4 | 5 | 6 | 7 | 8 | 9 | NA |
| 86E1 | Structured psychotherpy in monotherapy | 0 | 1 | 2 | 3 | 4 | 5 | 6 | 7 | 8 | 9 | NA |
| 86E2 | Structured psychotherpy in combination | 0 | 1 | 2 | 3 | 4 | 5 | 6 | 7 | 8 | 9 | NA |
| 86F1 | ECT in monotherapy | 0 | 1 | 2 | 3 | 4 | 5 | 6 | 7 | 8 | 9 | NA |
| 86F2 | ECT in combination | 0 | 1 | 2 | 3 | 4 | 5 | 6 | 7 | 8 | 9 | NA |
| 86F3 | rTMS in monotherapy | 0 | 1 | 2 | 3 | 4 | 5 | 6 | 7 | 8 | 9 | NA |
| 86F4 | rTMS in combination | 0 | 1 | 2 | 3 | 4 | 5 | 6 | 7 | 8 | 9 | NA |

**Question 87**

0 means that this strategy is not recommended in first intention.

1, 2, 3 means that this strategy is recommended in third intention.

4, 5, 6 means that this strategy is recommended in second intention.

7, 8, 9 means that this strategy is recommended in first intention.

NA means that you have no opinion and/or experience with this strategy in this indication.

| 87 | An adult patient under the age of 65, with no significant organic or psychiatric history, has a depressive unipolar episode treated successively by several antidepressants of different pharmacological classes, in monotherapy, in combination or in potentialization by second-generation antipsychotic. No response was obtained.  What strategy(s) do you recommend?  *Circle the number corresponding to your choice* |
| --- | --- |

| 87A1 | Switch to a SSRI in monotherapy  (e.g Citalopram) | 0 | 1 | 2 | 3 | 4 | 5 | 6 | 7 | 8 | 9 | NA |
| --- | --- | --- | --- | --- | --- | --- | --- | --- | --- | --- | --- | --- |
| 87A2 | Switch to a SNRI in monotherapy  (e.g Venlafaxine) | 0 | 1 | 2 | 3 | 4 | 5 | 6 | 7 | 8 | 9 | NA |
| 87A3 | Switch to a tricyclic antidepressant in monotherapy  (e.g Clomipramine) | 0 | 1 | 2 | 3 | 4 | 5 | 6 | 7 | 8 | 9 | NA |
| 87A4 | Switch to Mianserine / Mirtazapine in monotherapy | 0 | 1 | 2 | 3 | 4 | 5 | 6 | 7 | 8 | 9 | NA |
| 87A5 | Switch to Tianeptine in monotherapy | 0 | 1 | 2 | 3 | 4 | 5 | 6 | 7 | 8 | 9 | NA |
| 87A6 | Switch to Agomelatine in monotherapy | 0 | 1 | 2 | 3 | 4 | 5 | 6 | 7 | 8 | 9 | NA |
| 87A7 | Switch to MAOI A (Moclobemide) in monotherapy | 0 | 1 | 2 | 3 | 4 | 5 | 6 | 7 | 8 | 9 | NA |
| 87A8 | Switch to a non selectif irreversible MAOI (Iproniazide) in monotherapy | 0 | 1 | 2 | 3 | 4 | 5 | 6 | 7 | 8 | 9 | NA |
| 87A9 | Switch to Bupropion in monotherapy | 0 | 1 | 2 | 3 | 4 | 5 | 6 | 7 | 8 | 9 | NA |
| 87B1 | Adding a SSRI | 0 | 1 | 2 | 3 | 4 | 5 | 6 | 7 | 8 | 9 | NA |
| 87B2 | Adding a SNRI | 0 | 1 | 2 | 3 | 4 | 5 | 6 | 7 | 8 | 9 | NA |
| 87B3 | Adding a tricyclic antidepressant | 0 | 1 | 2 | 3 | 4 | 5 | 6 | 7 | 8 | 9 | NA |
| 87B4 | Adding Mianserine / Mirtazapine | 0 | 1 | 2 | 3 | 4 | 5 | 6 | 7 | 8 | 9 | NA |
| 87B5 | Adding Tianeptine | 0 | 1 | 2 | 3 | 4 | 5 | 6 | 7 | 8 | 9 | NA |
| 87B6 | Adding Agomelatine | 0 | 1 | 2 | 3 | 4 | 5 | 6 | 7 | 8 | 9 | NA |
| 87B7 | Adding MAOI A (Moclobemide) | 0 | 1 | 2 | 3 | 4 | 5 | 6 | 7 | 8 | 9 | NA |
| 87B8 | Adding Bupropion | 0 | 1 | 2 | 3 | 4 | 5 | 6 | 7 | 8 | 9 | NA |

| 87 (Continuated) | An adult patient under the age of 65, with no significant organic or psychiatric history, has a depressive unipolar episode treated successively by several antidepressants of different pharmacological classes, in monotherapy, in combination or in potentialization by second-generation antipsychotic. No response was obtained.  What strategy(s) do you recommend?  *Circle the number corresponding to your choice* |
| --- | --- |

| 87C1 | Adding lithium | 0 | 1 | 2 | 3 | 4 | 5 | 6 | 7 | 8 | 9 | NA |
| --- | --- | --- | --- | --- | --- | --- | --- | --- | --- | --- | --- | --- |
| 87C2 | Adding thyroid hormones | 0 | 1 | 2 | 3 | 4 | 5 | 6 | 7 | 8 | 9 | NA |
| 87C3 | Adding first-generation antipsychotic (e.g Haloperidol) | 0 | 1 | 2 | 3 | 4 | 5 | 6 | 7 | 8 | 9 | NA |
| 87C4 | Adding second-generation antipsychotic (e.g Risperidone) | 0 | 1 | 2 | 3 | 4 | 5 | 6 | 7 | 8 | 9 | NA |
| 87D1 | Pramipexole in combination | 0 | 1 | 2 | 3 | 4 | 5 | 6 | 7 | 8 | 9 | NA |
| 87D2 | Modafinil in combination | 0 | 1 | 2 | 3 | 4 | 5 | 6 | 7 | 8 | 9 | NA |
| 87D3 | Methylphenidate in combination | 0 | 1 | 2 | 3 | 4 | 5 | 6 | 7 | 8 | 9 | NA |
| 87D4 | Lamotrigine in combination | 0 | 1 | 2 | 3 | 4 | 5 | 6 | 7 | 8 | 9 | NA |
| 87D5 | Valproic acide derivatives (e.g Divalproex sodium) in combination | 0 | 1 | 2 | 3 | 4 | 5 | 6 | 7 | 8 | 9 | NA |
| 87D6 | Carbamazepine in combination | 0 | 1 | 2 | 3 | 4 | 5 | 6 | 7 | 8 | 9 | NA |
| 87D7 | Pregabaline in combination | 0 | 1 | 2 | 3 | 4 | 5 | 6 | 7 | 8 | 9 | NA |
| 87E1 | Structured psychotherpy in monotherapy | 0 | 1 | 2 | 3 | 4 | 5 | 6 | 7 | 8 | 9 | NA |
| 87E2 | Structured psychotherpy in combination | 0 | 1 | 2 | 3 | 4 | 5 | 6 | 7 | 8 | 9 | NA |
| 87F1 | ECT in monotherapy | 0 | 1 | 2 | 3 | 4 | 5 | 6 | 7 | 8 | 9 | NA |
| 87F2 | ECT in combination | 0 | 1 | 2 | 3 | 4 | 5 | 6 | 7 | 8 | 9 | NA |
| 87F3 | rTMS in monotherapy | 0 | 1 | 2 | 3 | 4 | 5 | 6 | 7 | 8 | 9 | NA |
| 87F4 | rTMS in combination | 0 | 1 | 2 | 3 | 4 | 5 | 6 | 7 | 8 | 9 | NA |
