# Supplementary material for: Clinical guidelines for the management of treatment-resistant depression: French recommendations from experts, the French Association for Biological Psychiatry and Neuropsychopharmacology and the fondation FondaMental
Source: BMC Psychiatry. 2019 Aug 28;19:262. doi: 10.1186/s12888-019-2237-x (PMC6712810; doi:10.1186/s12888-019-2237-x)
Supplement: Supplementary file 3 — Definitions (According to the American College of Neuropsychopharmacology) [47]. (DOCX 14 kb) [file 12888_2019_2237_MOESM3_ESM.docx]

**Additional file 3:** **Definitions (*According to the American College of Neuropsychopharmacology*)** ^44^

| **Response** | Response requires a clinically significant reduction of symptoms of depression after treatment initiation. Response is defined as of ≥50% decrease in scores on the Hamilton Depression Rating scale_17_ (HDRS) or on the Montgomery and Asberg Depression Rating Scale (MADRS) from baseline to 6 to 8 weeks of treatment. |
| --- | --- |
| **Complete Remission** | Complete Remission implies that the signs and symptoms of the illness be absent or close to it (1 or 2 signs of low intensity). Complete remission is defined as an HDRS_17_ score ≤7 or an MADRS score ≤ 10 during at least 2 consecutive weeks. |
| **Partial Remission** | Partial Remission is defined as the presence of 2 to 4 residuals symptoms of MDD. In clinical trials, partial remission is defined as an HDRS_17_ score between 8 and 14. |
| **Relapse and Recurrence** | Relapse is defined as the re-appearance of symptoms of MDD after remission and before complete recovery. Recurrence is defined as the re-appearance of a depressive episode after complete recovery |
| **Chronic Depression** | Chronic Depression are depression with a duration of at least 2 years |
