# Supplementary material for: Clinical guidelines for the management of treatment-resistant depression: French recommendations from experts, the French Association for Biological Psychiatry and Neuropsychopharmacology and the fondation FondaMental
Source: BMC Psychiatry. 2019 Aug 28;19:262. doi: 10.1186/s12888-019-2237-x (PMC6712810; doi:10.1186/s12888-019-2237-x)
Supplement: Supplementary file 4 — Antidepressant treatments classified according to the pharmacological profile. (DOCX 14 kb) [file 12888_2019_2237_MOESM4_ESM.docx]

**Additional file 4: Antidepressant treatments classified according to the pharmacological profile**

| **Selective serotonin reuptake inhibitors (SSRI)** |
| --- |
| Citalopram, Escitalopram, Fluoxetine, Fluvoxamine, Paroxetine, Sertraline |
| **Dual Serotonin and norepinephrine reuptake inhibitors (SNRI)** |
| Duloxetine, Milnacipran, Venlafaxine |
| **Imipraminic** |
| Amitriptyline, Amoxapine, Clomipramine, Dosulepine, Doxepine, Imipramine, Maprotiline, Trimipramine |
| **Alpha2-receptor antagonists** |
| Mianserine, Mirtazapine |
| **Irreversible, nonselective Monoamine oxidase inhibitors,** |
| Iproniazid |
| **Reversible and selective inhibitor of MAO-A** |
| Moclobemide |
| **Others** |
| Agomelatine, Bupropion ^1^, Tianeptine |

*^1^* *Bupropion is not authorized for treating depression in France*
