# Supplementary material for: Clinical guidelines for the management of treatment-resistant depression: French recommendations from experts, the French Association for Biological Psychiatry and Neuropsychopharmacology and the fondation FondaMental
Source: BMC Psychiatry. 2019 Aug 28;19:262. doi: 10.1186/s12888-019-2237-x (PMC6712810; doi:10.1186/s12888-019-2237-x)
Supplement: Supplementary file 5 — Expert Panel: description. (DOCX 14 kb) [file 12888_2019_2237_MOESM5_ESM.docx]

**Additional file 5: Expert Panel: description**

| **Age (years)** | N  Mean SD ± | 36  48 ± 8,6 |
| --- | --- | --- |
| **Years of practice** | N  < 10  10 – 20  > 20 | 36  16,7%  47,2%  36,1% |
| **Places of practice** | N  Office Practice  Private Clinic  General Hospital  Psychiatric Hospital  Others | 36  44,4%  27,8%  30,6%  38,9%  13,9% |
| **Type of activity** | N  Clinical activity  Teaching  Research projects | 36  97,2%  16,6%  11,1% |
| **Participations in Research Projects** | N  Participation  Participation in the field of unipolar depression or TRD  Other research projects | 36  86,1%  74,2%  25,8% |
| **Publications** | N  Participation  National Journal  International Journal | 36  55,6%  45%  75% |
| **Communications** | N  Conferences  National Congress  International Congress  Teaching | 36  63,9%  38,9%  19,4%  86,1% |
| **Brain Stimulation techniques** | N  ECT (Prescription and Practice)  ECT (Prescription only)  rTMS (Prescription and Practice)  rTMS (Prescription only)  tDCS (Prescription and Practice)  tDCS (Prescription only) | 36  33,3%  66,7%  11,1%  75%  11,1%  22,2% |

**Socio-demographic data and professional activities of the experts’ panel (N = 36 experts)**

**ECT:** Electroconvulsive therapy **; N :** Number of expert **; rTMS** repetitive Transcranial Magnetic Stimulation **; tDCS :** Transcranial Direct Current Stimulation ; **TRD :** Treatment Resistant Depression
