# Supplementary material for: Clinical guidelines for the management of treatment-resistant depression: French recommendations from experts, the French Association for Biological Psychiatry and Neuropsychopharmacology and the fondation FondaMental
Source: BMC Psychiatry. 2019 Aug 28;19:262. doi: 10.1186/s12888-019-2237-x (PMC6712810; doi:10.1186/s12888-019-2237-x)
Supplement: Supplementary file 6 — Tolerance profile of the antidepressants. (DOCX 147 kb) [file 12888_2019_2237_MOESM6_ESM.docx]

**Additional file 6: Tolerance profile of the antidepressants**

|  | **Cardiac** **tolerability** | **Metabolic**  **tolerability** | **Sexual**  **tolerability** | **Orthostatic hypotension** | **Weight Gain** | **Hepatic**  **tolerability** | **Digestive tolerability** | **Neurological tolerability** | **Ocular tolerability** | **Global**  **tolerability** |
| --- | --- | --- | --- | --- | --- | --- | --- | --- | --- | --- |
| **SSRI** |  |  |  |  |  |  |  |  |  |  |
| Citalopram | 5.0 (2.2) | 7.31 (1.04) | 5.0 (1.54) | 7.3 (1.32) | 6.5 (1.3) | 7.0 | 5.8 | 7.1 | 7.5 | 6.5 |
| Escitalopram | 4.61 (2.17) | 7.31 (1.12) | 4.8 (1.26) | 7.3 (1.46) | 6.5 (1.44) | 7.0 | 5.7 | 7.0 | 7.6 | 6.4 |
| Fluoxetine | 6.92 (1.73) | 7.72 (0.97) | 5.1 (1.51) | 7.4 (1.42) | 7.5 (1.58) | 7.0 | 5.7 | 6.8 | 7.4 | 6.8 |
| Fluvoxamine | 6.58 (1.58) | 6.88 (1.39) | 4.8 (1.58) | 6.8 (1.72) | 6.2 (1.67) | 6.6 | 5.4 | 6.4 | 7.0 | 6.3 |
| Paroxetine | 6.8 (1.58) | 6.75 (1.46) | 4.0 (1.74) | 6.8 (1.4) | 5.4 (1.55) | 6.9 | 5.5 | 6.3 | 6.9 | 6.2 |
| Sertraline | 7.3 (1.65) | 7.36 (1.27) | 5.0 (1.42) | 7.3 (1.49) | 6.6 (1.29) | 7.1 | 6.1 | 7.0 | 7.3 | 6.8 |
| **SNRI** |  |  |  |  |  |  |  |  |  |  |
| Duloxetine | 6.28 (1.72) | 7.0 (1.24) | 5.4 (1.27) | 6.3 (1.67) | 6.7 (1.3) | 6.2 | 5.6 | 6.4 | 6.9 | 6.3 |
| Milnacipran | 6.4 (1.67) | 6.9’ (1.37) | 5.8 (1.6) | 6.8 (1.48) | 6.6 (1.44) | 6.9 | 6.2 | 6.4 | 6.5 | 6.5 |
| Venlafaxine | 5.86 (1.91) | 6.78 (1.24) | 5.2 (1.35) | 6.3 (1.8) | 6.1 (1.58) | 6.7 | 5.8 | 6.1 | 6.5 | 6.2 |
| **OTHERS ATD** |  |  |  |  |  |  |  |  |  |  |
| Mianserine | 6.67 (1.8) | 5.19 (1.95) | 5.6 (1.98) | 5.8 (1.8) | 3.6 (1.67) | 6.6 | 6.3 | 7.0 | 6.3 | 5.9 |
| Mirtazapine | 6.83 (1.68) | 4.86 (1.95) | 5.8 (1.71) | 6.0 (1.81) | 3.4 (1.55) | 6.5 | 6.4 | 6.8 | 6.7 | 5.9 |
| Tianeptine | 7.29 (1.45) | 7.49 (1.31) | 7.1 (1.31) | 7.4 (1.56) | 7.5 (1.29) | 6.9 | 7.1 | 7.2 | 7.5 | 7.3 |
| Agomelatine | 7.75 (1.05) | 7.67 (1.17) | 7.5 (1.16) | 7.6 (1.42) | 7.6 (1.35) | 3.7 | 6.7 | 7.4 | 7.5 | 7.1 |
| Bupropion | 6.04 (1.68) | 7.0 (1.47) | 6.3 (1.69) | 6.4 (1.72) | 7.0 | 5.8 | 6.0 | 5.7 | 6.5 | 6.3 |
| **IMIPRAMINIC** |  |  |  |  |  |  |  |  |  |  |
| Amoxapine | 4.52 (1.45) | 5.0 (1.55) | 4.2 (1.31) | 4.1 (1.55) | 4.2 | 5.4 | 4.8 | 4.6 | 4.2 | 4.6 |
| Clomipramine | 4.42 (1.44) | 5.2 (1.4) | 3.9 (1.44) | 3.4 (1.46) | 4.2 | 5.9 | 4.5 | 4.5 | 3.8 | 4.4 |
| Dosulepine | 4.67 (1.58) | 5.2 (1.6) | 4.2 (1.53) | 3.7 (1.54) | 4.3 | 5.4 | 4.6 | 4.7 | 3.8 | 4.5 |
| Doxepine | 4.62 (1.5) | 5.1 (1.46) | 4.2 (1.32) | 3.9 (1.39) | 4.3 | 5.7 | 4.7 | 4.8 | 4.3 | 4.7 |
| Imipramine | 4.44 (1.66) | 5.1 (1.3) | 4.0 (1.56) | 3.7 (1.38) | 4.3 | 5.7 | 4.8 | 4.8 | 3.8 | 4.5 |
| Maprotiline | 4.44 (1.78) | 4.7 (1.32) | 4.2 (1.49) | 3.7 (1.46) | 3.9 | 5.7 | 4.8 | 4.7 | 4.0 | 4.5 |
| Trimipramine | 4.67 (1.49) | 4.6 (1.36) | 4.1 (1.37) | 3.8 (1.5) | 3.6 | 5.6 | 4.7 | 4.8 | 3.9 | 4.5 |
| **Reversible, selective** **MAOI-A** |  |  |  |  |  |  |  |  |  |  |
| Moclobemide | 6.5 (1.42) | 6.4 (1.55) | 6.3 (1.49) | 6.2 (1.76) | 6.6 | 6.2 | 6.4 | 6.3 | 6.6 | 6.4 |
| **Irreversible, nonselective** **MAOI** |  |  |  |  |  |  |  |  |  |  |
| Iproniazid | 5.0 (1.9) | 5.6 (1.76) | 5.4 (1.62) | 4.0 (1.99) | 5.7 | 5.0 | 5.8 | 5.5 | 5.4 | 5.3 |

**Tolerability of the antidepressants used in the treatment of unipolar depression in adults under 65 years of age. Values given as mean of scores on a scale ranging from 0 (extremely bad) to 9 (optimal) according to the level of tolerability**

**Initial treatment**

SSRI

SNRI

Imipraminic

α2 antagonist

Optimize Antidepressant

- Switch to SNRI

- Switch to Imipraminic AD

- Switch to SSRI

- Switch to SNRI

- Switch to SSRI

- Switch to another Imipraminic

- Association with α2 antagonist

- Potentiation with lithium

Switch to Imipraminic AD

- Switch to SNRI

- Switch to Imipraminc AD

- Switch to SNRI

- Switch to SSRI

- Switch to SNRI

**2nd Intention**

**1st Intention**

Switch to α2 antagonist

**Figure S1: Second-Strategies in case of partial response to the first-line treatment**

***AAP:*** *Atypical Antipsychotic****; AD:*** *Antidepressant****; SNRI:*** *Serotonin and Norepinephrine Reuptake Inhibitors;* ***SSRI****: Selective Serotonin Reuptake Inhibitors*

**Figure S2: Second line Strategies in case of non-response to the first-line treatment**

***AAP:*** *Atypical Antipsychotic****; AD:*** *Antidepressant****; SNRI****: Serotonin and Norepinephrine Reuptake Inhibitors;* ***SSRI****: Selective Serotonin Reuptake Inhibitors*

**Initial treatment**

SSRI

SNRI

Imipraminic

α2 antagonist

Optimize Antidepressant

- Association with α2 antagonist

- Switch to SNRI

- Switch to Imipraminic AD

- Switch to α2 antagonist

- Potentiation with lithium

- Switch to SSRI

- Switch to Imipraminic AD

- Switch to SNRI

- Potentiation with lithium

- Potentiation with lithium

- Potentiation with AAP

- Switch to Imipraminic AD

- Switch to SSRI

- Switch to SNRI

- Potentiation with Imipraminic AD

Association with α2 antagonist

- Association with α2 antagonist

- Switch to SNRI

- Switch to SSRI

- Switch to SNRI

**2nd Intention**

**1st Intention**

Two consecutives SSRI

SSRI then SNRI or vice versa

SSRI then

α2 antagonist or vice

versa

SSRI then Imipraminic or vice versa

SNRI then

α2 antagonist or vice

versa

SSRI then Imipraminic or vice versa

Optimize Antidepressant by increasing dose

- Switch to SNRI

- Switch to Imipraminic AD

- Association with α2 antagonist

- Switch to Imipraminic AD

- Switch to α2 antagonist

Switch to SNRI

- Switch to SNRI

- Association with α2 antagonist

Switch to Imipraminic AD

Association with α2 antagonist

- Switch to α2 antagonist

- Potentiation with lithium

- Switch to α2 antagonist

- Potentiation with lithium or AAP

Potentiation with lithium or Antipsychotic

Potentiation with lithium or AAP

- Association with ECT or rTMS

2nd Intention

1rst Intention

**Figure S3: Third-line Strategies in case of partial response to the Second-line strategy**

***AAP :*** *Atypical Antipsychotic****; AD :*** *Antidepressant****; ECT :*** *electroconvuslive therapy****; SNRI****: Serotonin and Norepinephrine Reuptake Inhibitors ;* ***SSRI*** *: Selective Serotonin Reuptake Inhibitors ;* ***rTMS****: repetitive transcranial magnetic stimulation*

**Figure S4: Third-line Strategies in case of non-response to the Second-line strategy**

***AD:*** *Antidepressant****; SNRI****: Serotonin and Norepinephrine Reuptake Inhibitors;* ***SSRI****: Selective Serotonin Reuptake Inhibitors;* ***rTMS****: repetitive transcranial magnetic stimulation*

Two consecutives SSRI

SSRI then SNRI or vice versa

SSRI then

α2 antagonist or vice

versa

SSRI then Imipraminic or vice versa

SNRI then

α2 antagonist or vice

versa

SSRI then Imipraminic or vice versa

- Switch to SNRI

- Switch to Imipraminic AD

Switch to Imipraminic AD

- Switch to SNRI

- Switch to Imipraminic AD

Switch to SNRI

Switch to Imipraminic AD

Association with α2 antagonist

Switch to α2 antagonist

- Switch to α2 antagonist

- Potentiation with lithium

- Association with rTMS

**2nd** **Intention**

**1rst Intention**

Association with rTMS

- Switch to α2 antagonist

- Potentiation with lithium

- Association with rTMS

- Switch to SNRI

- Potentiation with lithium

- Association with rTMS

**Figure S5: Fourth-Line Strategies**

***** The expert’s answers have not made it possible to adequately differentiated first and second intention strategies, in this situation.

****** *In monotherapy or in combination*

***AAP :*** *Atypical Antipsychotic****; AD :*** *Antidepressant****; ECT :*** *electroconvulsive therapy****; SNRI****: Serotonin and Norepinephrine Reuptake Inhibitors ;* ***SSRI*** *: Selective Serotonin Reuptake Inhibitors ;* ***rTMS****: repetitive transcranial magnetic stimulation*

Previous Strategies:

SSRI

SNRI

Imipraminic ATD*

- MAOI

- Association with α2 antagonist

- Potentiation with lithium or AAP

- ECT*

- rTMS*

- MAOI

- Association with α2 antagonist

- Potentiation with lithium or AAP

- ECT**

- rTMS**

Switch to Imipraminic AD

Previous Strategies :

SSRI

SNRI

α2 antagonist

1rs Intention

2nd Intention

Previous Strategies :

SSRI

SNRI

α2 antagonist

Imipraminic

Association with ECT

Potentiation with lithium

1rst line

Association with rTMS

ECT in monotherapy

Irreversible MAOI

Potentiation with AAP or tri-iodothyronine

2nd line

**Figure S6: Fifth Line strategy**

***AAP :*** *Atypical Antipsychotic****; AD :*** *Antidepressant****; ECT :*** *electroconvulsive therapy****; MAOI :*** *Monoamine oxydase inhibitor****; SNRI****: Serotonin and Norepinephrine Reuptake Inhibitors ;* ***SSRI*** *: Selective Serotonin Reuptake Inhibitors ;* ***rTMS****: repetitive transcranial magnetic stimulation*

**Figure S7: Strategies from the sixth line of treatment**

***AAP :*** *Atypical Antipsychotic****; AD :*** *Antidepressant****; ECT :*** *electroconvulsive therapy****; MAOI :*** *Monoamine oxidase inhibitor****; SNRI****: Serotonin and Norepinephrine Reuptake Inhibitors ;* ***SSRI*** *: Selective Serotonin Reuptake Inhibitors ;* ***rTMS****: repetitive transcranial magnetic stimulation*

Previous Strategies:

Monotherapy or Combination of ATD from different pharmacological class

Potentiation with AAP

Potentiation with Lithium

Potentiation with tri-iodothyronine

ECT*

ECT*

Potentiation with Lithium

Potentiation with AAP or

tri-iodothyronine

Combine with rTMS

Add Lamictal or tri-iodothyronine

Association with rTMS

Potentiation with AAP or Lamotrigine or tri-iodothyronine

ECT*

Association with rTMS

Potentiation with Lithium

Association with rTMS

Potentiation with AAP or Lamotrigine

1rst Intention

2nd intention

Association with rTMS

Potentiation with Lamictal or tri-iodothyronine
